# Supplementary material for: Quantum advantage for learning shallow neural networks with natural data distributions
Source: Nat Commun. 2025 Dec 31;17:1341. doi: 10.1038/s41467-025-68097-2 (PMC12873301; doi:10.1038/s41467-025-68097-2)
Supplement: Supplementary file 1 — Supplementary Information [file 41467_2025_68097_MOESM1_ESM.pdf]

Quantum advantage for learning shallow neural networks with natural data distributions

Laura Lewis,[1,2,3,*] Dar Gilboa,[1] and Jarrod R. McClean[1]

[1] *Google Quantum AI, Venice, CA, USA*
[2] *University of Cambridge, Cambridge, UK*
[3] *University of Edinburgh, Edinburgh, UK*

Supplementary Information

CONTENTS

I. Supplementary Note 1 - Preliminaries 1
A. Quantum learning theory 1
B. Hallgren's irrational period finding algorithm 3

II. Supplementary Note 2 - Detailed problem statement 4

III. Supplementary Note 3 - Classical hardness 5
A. Classical hardness for gradient-based methods 5
B. Correlational SQ lower bound 10

IV. Supplementary Note 4 - Uniform data distribution 13
A. Learning the linear function 14
1. Warmup 14
2. General Case 16
B. Learning the outer function via gradient methods 31
C. Integral bounds 43

V. Supplementary Note 5 - Non-uniform distributions 50
A. Learning the linear function 55
1. Warmup 56
2. General case 59
B. Learning the outer function via gradient methods 73
C. Integral bounds 74

I. SUPPLEMENTARY NOTE 1 - PRELIMINARIES

A. Quantum learning theory

In classical learning theory, the goal is to learn a collection of functions $\mathcal{C} \subseteq \{c : \mathcal{X} \to \mathcal{Y}\}$ with input space $\mathcal{X}$ and output space $\mathcal{Y}$. Typically, for Boolean functions, $\mathcal{X} = \{0,1\}^d, \mathcal{Y} = \{0,1\}$, where d is the input dimension, but in general, one could have any $\mathcal{X} \subseteq \mathbb{R}^d, \mathcal{Y} \subseteq \mathbb{R}$. This collection $\mathcal{C}$ is called a *concept class*. Two common models used in classical learning theory are the *probably approximately correct (PAC) model* [1] and the *statistical query (SQ) model* [2]. In classical PAC learning, a learning algorithm is given labeled random examples $\{(x_i, c^\star(x_i))\}_{i=1}^N$, where the x_i are sampled i.i.d. according to an unknown distribution $\mathcal{D}$ over the input space $\mathcal{X}$. The goal is to learn the unknown target function $c^\star$ up to some error with high probability. More precisely, an (ϵ, δ)-*PAC learner* for $c^\star$ outputs a hypothesis function $h : \mathcal{X} \to \mathcal{Y}$ such that

$$\mathcal{L}(h) \leq \epsilon \tag{1}$$

* llewis@alumni.caltech.edu

with probability at least $1-\delta$ for some loss function $\mathcal{L}$. Typically, the loss function is chosen as the squared loss $\mathbb{E}_{x\sim\mathcal{D}}(h(x)-c^\star(x))^2$ or the misclassification error $\Pr_{x\sim\mathcal{D}}(h(x)\neq c^\star(x))$. One often wants to minimize the amount of training data N, or the *sample complexity*, needed to learn any unknown target function $c^\star$ from the concept class $\mathcal{C}$ for any unknown distribution $\mathcal{D}$. Meanwhile, in classical SQ learning, rather than having direct access to the examples, a learning algorithm only has access to noisy expectation values of functions of the data. In particular, an SQ learner has access to a statistical query oracle, which takes as input a tolerance parameter $\tau \geq 0$ and a function $\phi : \mathcal{X}\times\mathcal{Y}\to\mathcal{Y}$ and outputs a number α such that

$$\left|\alpha - \mathop{\mathbb{E}}_{x\sim\mathcal{D}}[\phi(x, c^\star(x))]\right| \leq \tau. \tag{2}$$

Then, an (ϵ,δ)-*SQ learner* outputs a hypothesis function satisfying Equation (1) with probability $1-\delta$. In the some definitions of statistical query learning, the parameter δ is not present. Here, we include it to allow for a probability of failure in randomized learning algorithms, as noted in [2]. In this case, the measure of complexity is the number of queries, or the *query complexity*, needed to learn any unknown target function $c^\star$ from the concept class $\mathcal{C}$ for any unknown distribution $\mathcal{D}$.

Both PAC and SQ learning have been extended to the quantum setting in the quantum PAC model [3] and quantum statistical query (QSQ) model [4], respectively. Here, the only difference is the access model, in which quantum learning algorithms are given access to quantum data instead. Specifically, in quantum PAC learning [3], a quantum learner is given copies of the quantum example state

$$|c^\star\rangle \triangleq \sum_{x\in\mathcal{X}} \sqrt{\mathcal{D}(x)}\,|x, c^\star(x)\rangle\,. \tag{3}$$

The learning algorithm is allowed to perform (potentially entangled) measurements on the example states, and in this case, one wants to minimize the number of copies of the example states used to learn the concept class. Finally, in the QSQ model [4], a learner has access to a QSQ oracle, which takes as input a tolerance parameter $\tau \geq 0$ and an observable O such that $\|O\|\leq 1$ and outputs a number α such that

$$|\alpha - \langle c^\star|O|c^\star\rangle| \leq \tau. \tag{4}$$

The goal is again to minimize the number of queries to the QSQ oracle needed to learn the concept class $\mathcal{C}$. A key difference between the quantum PAC setting and the QSQ setting is that in the PAC setting, the learner may perform entangled measurements across multiple copies of the quantum example state [5, 6].

In this work, we focus on the QSQ access model with noise tolerance $\tau \geq 0$ for learning a particular concept class (defined in Section II) in the distribution-specific setting, where $\mathcal{D}$ is known to be either uniform or a discrete Gaussian with a diagonal covariance matrix. Moreover, we consider functions with real inputs and outputs, so we redefine QSQ access for real functions.

Definition 1 (Quantum statistical query access for real functions)**.** *Let $\mathcal{C}\subseteq\{c:\mathbb{R}^d\to\mathbb{R}\}$ be a concept class, where $d\geq 1$ is the input dimension. Let $\mathcal{D}$ be a probability distribution over $\mathbb{R}^d$. A quantum statistical query oracle for some $c^\star\in\mathcal{C}$ receives as input a tolerance parameter $\tau\geq 0$, discretization/truncation parameters $M, R\geq 1$, respectively, and an observable O such that $\|O\|\leq 1$, and outputs a number α such that*

$$|\alpha - \langle h_M^*|O|h_M^*\rangle\,| \leq \tau, \tag{5}$$

where $|h_M^*\rangle$ is the quantum example state

$$|h_M^*\rangle = \sum_{x_1,\ldots,x_d=-R}^{R-1} \sqrt{\mathcal{D}(x)}\,|x\rangle\,|h_M^*(x)\rangle \tag{6}$$

and h_M^* is a suitable discretization of the target $c^\star$ and $\mathcal{D}$ must be suitably renormalized.

Without loss of generality, beyond $\tau > 0$, we consider the QSQ model in which the output α is a rational number. We can do this because the rational numbers are dense in $\mathbb{R}$. Then, if a QSQ outputs an irrational number, we can find a rational number close to it and consider the error in this approximation as a part of the tolerance of the QSQ.

One may also consider multiple discretization parameters if necessary. We remark that allowing one to specify the discretization/truncation parameters rather than fixing them throughout should not be too powerful. Notably, classical SQ access can approximate expectation values of a real target function itself, without needing the intermediary step of discretization at all.

B. Hallgren's irrational period finding algorithm

In this section, we give an overview of Hallgren's irrational period finding algorithm [7]. For more detailed presentations, we refer the reader to [8, 9]. This algorithm was originally a subroutine for a quantum algorithm for solving Pell's equation in number theory. However, we will only focus on this subroutine, which is sufficient for our purposes.

One of the most well-known quantum algorithms is Shor's period finding algorithm [10]. Given access to a function $f : \mathbb{Z}_N \to \mathbb{Z}_M$ which is periodic with period $S \in \mathbb{N}$, this algorithm can identify S up to some precision. However, the algorithm crucially relies on the fact that the period is an integer. Namely, recall that Shor's algorithm utilizes the continued fractions algorithm to recover the period from the quantum measurement outcomes. Without the assumption that $S \in \mathbb{N}$, directly using continued fractions is not guaranteed to recover an approximation of S. Thus, if one hopes to generalize Shor's algorithm to real functions with real periods, one must do something more complicated. This is exactly what Hallgren's algorithm does.

Consider a function $f : \mathbb{R} \to X$ which is periodic with period $S \in \mathbb{R}$. Here, X is some output space, which may be continuous-valued. In order to access f on a quantum computer, we must suitably discretize it. However, this must be done with some care, as "bad" discretizations can cause us to lose all information about the period in the new discretized function. The notion of pseudoperiodicity defined below excludes this possibility.

Definition 2 (Pseudoperiodic [7]). *A function $f : \mathbb{Z} \to X$ for some output space X is* pseudoperiodic *with period $S \in \mathbb{R}$ if for each $0 \leq k \leq \lfloor S \rfloor$ and each $\ell \in \mathbb{Z}$, either $f(k + \lfloor \ell S \rfloor)$ or $f(k + \lceil \ell S \rceil)$ equals $f(k)$. f is* η-pseudoperiodic *with period S if this condition holds for at least an η-fraction of inputs $0 \leq k \leq \lfloor S \rfloor$.*

This ensures that the discretization still encodes sufficient information about the period of the original function. Thus, from here, we consider a pseudoperiodic discretization of the real function we want to learn the period of. Hallgren's algorithm provides a guarantee for recovering the period of a pseudoperiodic function, which we restate below. We also present the algorithm in Algorithm 1.

Theorem 1 (Lemma 3.1 in [7]). *Let f be an η-pseudoperiodic function with period $S \in \mathbb{R}$. Suppose that, given an integer T, we can efficiently check (in time* polylog(S)*) whether or not $|\ell S - T| < 1$ for some $\ell \in \mathbb{Z}$. Additionally, suppose that we have an upper bound A on S. Then, there exists a quantum algorithm that outputs an integer a such that $|S - a| \leq 1$ with probability $\Omega(\eta^2/(\log A)^4)$. Moreover, the algorithm runs in time* polylog(A).

Algorithm 1: Hallgren's Algorithm

1: Choose an integer $q \geq 3S^2$ (this can be satisfied by choosing $q \geq 3A^2$).
2: Apply quantum Fourier sampling to the function f over $\mathbb{Z}_q$ twice. Let $b, c \in \mathbb{Z}$ be the outputs.
3: Compute the continued fraction expansion of b/c.
4: For each convergent b_i/c_i in the continued fraction expansion, use the verification procedure to check whether $\lfloor b_i q/b \rfloor$ or $\lceil b_i q/b \rceil$ is an integer multiple of the period S.
5: **return** the smallest value that passed the test from the previous step.

We note that there are two key subroutines in Hallgren's algorithm: quantum Fourier sampling (as in the standard period finding algorithm) and the verification procedure to check if a given guess is indeed close to the period. For a periodic function f, checking if a given guess is a multiple of the period is simple with query access to f. However, for η-pseudoperiodic functions, this is nontrivial. Hence, in order to apply Theorem 1, one must ensure that this condition is satisfied.

We give a brief sketch the proof of Theorem 1, as our proofs in Sections IV A 2 and V A 2 rely on similar ideas.

Proof Sketch of Theorem 1. We consider f to be pseudoperiodic on the whole domain for simplicity, as this only affects the success probability, which we will incorporate later. Querying the pseudoperiodic function f in superposition and measuring the last register, we get

$$\frac{1}{\sqrt{p}} \sum_{k=0}^{p-1} |x_0 + [kS]\rangle \,, \tag{7}$$

where $[kS]$ denotes one of $\lfloor kS \rfloor$ or $\lceil kS \rceil$, $0 \leq x_0 \leq \lfloor S \rfloor$, and $p = \lfloor q/S \rfloor$. By the shift invariance property of the Fourier transform, we can assume without loss of generality that $x_0 = 0$. Then, applying the quantum Fourier transform mod q, we have

$$\frac{1}{\sqrt{pq}} \sum_{k=0}^{p-1} \sum_{y=0}^{q-1} e^{2\pi i y [kS]/q} |y\rangle . \tag{8}$$

Thus, the probability of measuring some y is $(1/pq) \left| \sum_{k=0}^{p-1} e^{2\pi i y [kS]/q} \right|^2$. Using this, [7] lower bounds the probability of measuring some $y = \lfloor aq/S \rceil$ such that $y < q/\log A$, where a is an integer and $\lfloor \cdot \rceil$ denotes rounding to the closest integer. In particular, they show that one can lower bound this probability by $\Omega(1/S)$. In total, the probability that quantum Fourier sampling produces two such values (as in Step 2 of Algorithm 1) that are also relatively prime is then $\Omega(\eta^2/\log^4(A))$.

Now, consider obtaining two values $b \triangleq \lfloor kq/S \rceil$ and $c \triangleq \lfloor \ell q/S \rceil$ from this quantum Fourier sampling. [7] shows that k/ℓ is a convergent in the continued fraction expansion of b/c. This is shown by proving that $|b/c - k/\ell| \leq 1/(2\ell^2)$, as this implies the desired result [11]. Finally, the proof concludes by showing that $\lfloor kq/S \rceil$ is close to an integer multiple of the period S. This justifies Steps 3-5 of Algorithm 1, which iterates through all convergents in the continued fractions expansion of b/c and checks which one is close to an integer multiple of the period. The proof guarantees that at least one such convergent will indeed be close to the period. □

II. SUPPLEMENTARY NOTE 2 - DETAILED PROBLEM STATEMENT

In this section, we define the concept class we wish to learn precisely. We want to learn functions that are a composition of a periodic function and a linear function, as these are classically hard to learn via gradient methods [12, 13]. Moreover, previous works have shown that this class is hard to learn classically even for SQ algorithms and efficient classical algorithms learning under small amounts of noise [14, 15]. We consider a slightly restricted setting, which we show is still hard for classical gradient methods in Section III A. [14, 15] do not directly apply to our parameter regimes, but nevertheless, these works constitute strong evidence that the problem is hard for broader classes of classical algorithms.

Let $d \geq 1$ denote the input dimension, and define the set of vectors with fixed norm $R_w > 0$ satisfying $w_j \geq R_w/d^2$:

$$\mathcal{S}_w \triangleq \left\{ w \in R_w \mathbb{S}^{d-1} : w_j \geq \frac{R_w}{d^2}, \ \forall j \in [d] \right\}. \tag{9}$$

Here, $\mathbb{S}^{d-1}$ denotes the $(d-1)$-dimensional unit sphere, which lives in $\mathbb{R}^d$. Let $\tilde{\mathcal{S}}_w$ be a 0.51-packing net of the set $\mathcal{S}_w$, i.e., $\tilde{\mathcal{S}}_w \subseteq \mathcal{S}_w$ such that each point in $\tilde{\mathcal{S}}_w$ is separated by a geodesic angle of at least 0.51. Let $w^\star \in \tilde{\mathcal{S}}_w$ be a vector in $\tilde{\mathcal{S}}_w$. We remark that [12] considers $w^\star$ in $R_w \mathbb{S}^{d-1}$ directly, without requiring that $w_j \geq R_w/d^2$ or that $w^\star$ is taken from a packing net over this set. We extend their proof of classical hardness to our setting in Section III A[1].

Let $\tilde{g} : \mathbb{R} \to [-1, 1]$ be a periodic function of period 1 which has bounded variation on every finite interval. In particular, we assume that $\tilde{g}$ can be written as

$$\tilde{g}(y) = \sum_{j=1}^{D} \beta_j^\star \cos(2\pi j y), \quad \|\beta^\star\|_1 = 1, \tag{10}$$

for some constant $D > 0$. It is clear that a function of this form has period 1 and has bounded variation on every finite interval[2]. Here, the condition on the norm of the $\beta^\star$ coefficients ensures that the range of $\tilde{g}$ is in $[-1, 1]$. This is an additional assumption to those considered in [12, 13], but we do not expect this to affect the classical hardness. Namely, the hardness stems from $\tilde{g}$ preserving the Fourier sparsity of the input distribution, and this property is still preserved when taking $\tilde{g}$ to have this specific form.

[1] We note that our classical hardness in fact holds when considering $w_j \geq R_w/d^v$ where v is any constant greater than 3/2, but we choose $v = 2$ for simplicity.

[2] One could also choose to write $\tilde{g}$ as a linear combination of sines and cosines to resemble a Fourier series with a finite number of nonzero terms, but adding sines makes the analysis more cumbersome than instructive and does not affect the classical hardness.

Concretely, [12] also considers an example where $\tilde{g}$ takes this form (in particular, where $\tilde{g}$ is simply a cosine, i.e., $D = 1$), and the hardness result still holds. Our concept class consists of these functions

$$\mathcal{C} \triangleq \{g_{w^\star} : \mathbb{R}^d \to [-1,1] : g_{w^\star}(x) = \tilde{g}(x^\intercal w^\star),\ w^\star \in \tilde{\mathcal{S}}_w\}, \tag{11}$$

with $\tilde{g}$ defined in Equation (10). Hence, to learn a target function $g_{w^\star}$ in the concept class, it would be sufficient, but perhaps not necessary, to identify $w^\star$ and $\beta^\star$.

We devise a quantum learning algorithm given QSQ access (see Definition 1) to functions in this concept class when the distribution $\mathcal{D}$ is fixed to be either uniform or a discrete Gaussian with a diagonal covariance matrix $\Sigma = \mathrm{diag}(\sigma_1^2, \ldots, \sigma_d^2)$ for sufficiently large σ_j. In particular, for QSQ access with respect to a truncation parameter R, we require $\sigma_j = \Omega(R)$. We specify the discretization and truncation parameters in more detail in later sections.

To learn a target concept $g_{w^\star}$ with respect to a distribution $\mathcal{D}$, we want to find a good predictor $f_\theta(x)$ which minimizes the objective function

$$\min_{\theta\in\Theta} \mathcal{L}_{w^\star}(\theta) \triangleq \min_{\theta\in\Theta} \mathop{\mathbb{E}}_{x\sim\mathcal{D}}[(f_\theta(x) - g_{w^\star}(x))^2], \tag{12}$$

where θ are some parameters that we want to learn. Here, we use the squared loss to align with the classical hardness results [12, 13]. As in the classical case, we assume that we have access to this loss function and can compute it for a given choice of parameters θ. Here, SQ access [2] is more general than only having access to (gradients of) the loss function, as it allows the learning algorithm to access expectations of arbitrary functions of the data. Nonetheless, the SQ setting is a natural generalization of the gradient access model due to the similarities of the arguments used to prove hardness in [12] with those of [2, 16]. This is discussed in [12]. Thus, we find that the most natural quantum analogue for learning is the QSQ model with noise tolerance $\tau \geq 0$. For a given precision $\epsilon > 0$, our quantum algorithm will find parameters θ such that $\mathcal{L}_{w^\star}(\theta) \leq \epsilon$.

To quantify the performance of our quantum algorithm, we count any accesses to the unknown function $g_{w^\star}$. Namely, we consider both the number of QSQs and the number of (classical) queries to the gradient of the objective function $\mathcal{L}_{w^\star}$. This is the most fair comparison to the classical lower bound from [12], which is also in terms of the number of queries to the gradient of the objective function.

III. SUPPLEMENTARY NOTE 3 - CLASSICAL HARDNESS

A. Classical hardness for gradient-based methods

In this section, we discuss the hardness of the task detailed in Section II for classical gradient-based methods. This hardness result was already proven in Ref. [12] under a different setting. Notably, the classical hardness results [12, 13] hold for any distribution whose density is Fourier-concentrated, in the sense of the following definition.

Definition 3 (Fourier-concentrated [12]). *Let $\epsilon(r)$ be some function from $[0,\infty) \to [0,1]$. A density function $\varphi^2 : \mathbb{R}^d \to \mathbb{R}$ is $\epsilon(r)$-*Fourier-concentrated *if its square root φ belongs to $L^2(\mathbb{R}^d)$ (square integrable) and satisfies*

$$\|\hat{\varphi} \cdot \mathbf{1}_{\geq r}\|_2 \leq \|\hat{\varphi}\|_2 \epsilon(r), \tag{13}$$

where $\mathbf{1}_{\geq r}$ is the indicator function of $\{x : \|x\|_2 \geq r\}$.

Several common distributions are Fourier concentrated. For instance, $\epsilon(r)$ will decay subexponentially when φ is a member of various classes of smooth functions such as Gaussians.

For the task detailed in Section II, we have two additional assumptions compared to [12], designed to facilitate error analysis under finite precision, which we argue here do not affect the classical hardness. First, we sample the vector $w^\star$ from a 0.51-packing net $\tilde{\mathcal{S}}_w$ of the set $\mathcal{S}_w$ defined by

$$\mathcal{S}_w \triangleq \left\{ w \in R_w \mathbb{S}^{d-1} : w_j \geq \frac{R_w}{d^2},\ \forall j \in [d] \right\}, \tag{14}$$

where $\mathbb{S}^{d-1} \subseteq \mathbb{R}^d$ is the $(d-1)$-dimensional unit sphere. Second, we consider the function $\tilde{g}$ to be of a specific form given in Equation (10).

Instead, Ref. [12] considers $w^\star$ sampled from $R_w\mathbb{S}^{d-1}$ and $\tilde{g}$ as an arbitrary function with period 1 and with bounded variation on every finite interval. Note that the latter should not affect classical hardness,

as our choice of $\tilde{g}$ still preserves the crucial property of Fourier-concentration. Moreover, [12] considers an example where $\tilde{g}$ takes this form (namely when $\tilde{g}$ is simply a cosine), and the classical hardness still holds. Thus, we do not concern ourselves with the form of $\tilde{g}$ and mainly focus on the former case.

The key result in [12] that proves classical hardness is their Theorem 3. Examining the proof, we notice that the only part that relies on $w^\star$ being sampled from $R_w\mathbb{S}^{d-1}$ is Lemma 5 in [12], which we restate below. Informally, Lemma 5 tells us that for any function h, for a random choice of $w^\star$, the Fourier transform of the target function does not correlate well with h. Thus, no matter what our hypothesis function is, obtaining information about $w^\star$ should be difficult. The crux of the classical hardness says that, in particular, the gradient of the loss function does not contain much information about $w^\star$.

Lemma 1 (Lemma 5 in [12])**.** *Let φ^2 be a density function on $\mathbb{R}^d$ that is $\epsilon(r)$-Fourier-concentrated. For any square integrable function $h : \mathbb{R}^d \to \mathbb{R}$, if $d \geq c'$ (for some universal constant c') and we sample $w^\star$ uniformly at random from $R_w\mathbb{S}^{d-1}$, then*

$$\mathbb{E}\left[\left(\langle h, \widehat{g_{w^\star}\varphi}\rangle - a_0\langle h, \hat{\varphi}\rangle\right)^2\right] \leq 10\|h\|^2\left(\exp(-cd) + \sum_{n=1}^{\infty}\epsilon\left(\frac{nR_w}{2}\right)\right), \tag{15}$$

where a_0, c are constants and $\widehat{g_{w^\star}\varphi}$ denotes the Fourier transform of the pointwise product of $g_{w^\star}$ and φ.

Here, the inner product is defined as

$$\langle f, h\rangle = \int_x f(x)\overline{h(x)}\,dx \tag{16}$$

and the norm is $\|f\| = \sqrt{\langle f, f\rangle}$. Also, the hat denotes the Fourier transform defined via

$$\hat{f}(y) = \int \exp(-2\pi i x^\intercal y) f(x)\,dx. \tag{17}$$

Instead, we prove the following similar result.

Lemma 2. *Let φ^2 be a density function on $\mathbb{R}^d$ that is $\epsilon(r)$-Fourier-concentrated. For any square integrable function $h : \mathbb{R}^d \to \mathbb{R}$, if $d \geq c'$ (for some universal constant c') and we sample $w^\star$ uniformly at random from $\tilde{\mathcal{S}}_w$, then*

$$\mathop{\mathbb{E}}_{w^\star\sim\tilde{\mathcal{S}}_w}\left[\left(\langle h, \widehat{g_{w^\star}\varphi}\rangle - a_0\langle h, \hat{\varphi}\rangle\right)^2\right] \leq 10\|h\|^2\left(\exp(-cd) + \sum_{n=1}^{\infty}\epsilon\left(\frac{nR_w}{4}\right)\right), \tag{18}$$

where a_0, c are constants.

Note that the difference from Lemma 1 resulting from sampling from the packing net instead of the continuous space is that $R_w/2$ is replaced by $R_w/4$. Before proving Lemma 2, we need to show the following lemma, which says that there exists a large 0.51-packing net of $\mathcal{S}_w$. The choice of 0.51 is made for convenience, and other choices are possible.

Lemma 3. *For $v > 3/2$ and d sufficiently large, there exists a 0.51-packing net $\tilde{\mathcal{S}}_w$ of the set $\mathcal{S}_w$ such that $|\tilde{\mathcal{S}}_w| > e^{cd}$, where is c is an absolute constant.*

Proof. We first prove a lower bound on the volume of $\mathcal{S}_w$, and then show that this implies that a large packing net exists. Define the annulus of width R_w/d^v around the equator as

$$\mathrm{Ann}(d-1, R_w, R_w/d^v) \triangleq \left\{w \in R_w\mathbb{S}^{d-1} : |w_1| \leq R_w/d^v\right\}. \tag{19}$$

The complement of this annulus on the hypersphere is the union of two antipodal spherical caps, where a spherical cap is a portion of a sphere cut off by a plane. Note that spherical caps can be defined via the angle between the rays from the center of the sphere to the pole and to the edge of the base of the cap, called the half angle. The half angle θ subtended by each of these antipodal spherical caps satisfies $\cos\theta = 1/d^v$. Moreover, it is known [17] that the volume of a hyperspherical cap with half angle θ can be computed as

$$\mathrm{Vol}(\mathrm{Cap}(d, R_w, \theta)) = \frac{1}{2}\mathrm{Vol}(R_w\mathbb{S}^{d-1}) I_{\sin^2\theta}\left(\frac{d}{2}, \frac{1}{2}\right), \tag{20}$$

where $\text{Cap}(d, R_w, \theta)$ denotes a hyperspherical cap with half angle θ of a sphere in $\mathbb{R}^d$ with radius R_w. Also, $I_x(a, b)$ denotes the normalized incomplete Beta function

$$I_x(a,b) \triangleq \frac{B_x(a,b)}{B_1(a,b)}, \quad B_x(a,b) \triangleq \int_0^x t^{a-1}(1-t)^{b-1}\,dt. \tag{21}$$

Using that the annulus defined is the complement of the union of two antipodal spherical caps, we can compute its volume as

$$\begin{aligned}
\text{Vol}\left(\text{Ann}\left(d-1, R_w, R_w/d^v\right)\right) &= \text{Vol}(R_w\mathbb{S}^{d-1}) - 2\text{Vol}\left(\text{Cap}\left(d, R_w, \arccos\left(1/d^v\right)\right)\right) &(22)\\
&= \text{Vol}(R_w\mathbb{S}^{d-1})\left(1 - I_{\sin^2(\arccos(1/d^v))}\left(d/2, 1/2\right)\right) &(23)\\
&= \text{Vol}(R_w\mathbb{S}^{d-1})\left(1 - I_{1-1/d^{2v}}(d/2, 1/2)\right). &(24)
\end{aligned}$$

We can bound the second term above. First, expanding in terms of the definition, we have:

$$1 - I_{1-1/d^{2v}}(d/2,1/2) = 1 - \frac{1}{B_1(d/2,1/2)}\left(\int_0^t t^{d/2-1}(1-t)^{-1/2}\,dt - \int_{1-1/d^{2v}}^1 t^{d/2-1}(1-t)^{-1/2}\,dt\right) \tag{25}$$

$$= \frac{1}{B_1(d/2,1/2)}\int_{1-1/d^{2v}}^1 t^{d/2-1}(1-t)^{-1/2}\,dt. \tag{26}$$

We can bound the integral as

$$\int_{1-1/d^{2v}}^1 t^{d/2-1}(1-t)^{-1/2}\,dt \le \int_{1-1/d^{2v}}^1 (1-t)^{-1/2}\,dt = \frac{2}{d^v}. \tag{27}$$

Moreover, we can lower bound the beta function. Recall that the Beta function can be written in terms of Gamma functions:

$$B(d/2,1/2) = \frac{\Gamma(d/2)\Gamma(1/2)}{\Gamma(d/2+1/2)}. \tag{28}$$

Standard bounds on ratios of Gamma functions [18] give

$$B(d/2,1/2) \ge cd^{-1/2} \tag{29}$$

for some absolute constant c. Putting everything together, we see that

$$\text{Vol}(\text{Ann}(d-1, R_w, R_w/d^v)) \le C\,\text{Vol}(R_w\mathbb{S}^{d-1})d^{1/2-s} \tag{30}$$

for some absolute constant C. Denote

$$\mathcal{S}_{w,\pm} \triangleq \left\{w \in R_w\mathbb{S}^{d-1} : |w_j| \ge R_w/d^v,\ \forall j \in [d]\right\}. \tag{31}$$

Using our previous work, we can lower bound the volume of this set:

$$\begin{aligned}
\text{Vol}(\mathcal{S}_{w,\pm}) &\ge \text{Vol}(R_w\mathbb{S}^{d-1})\left(1 - d\,\text{Vol}(\text{Ann}(d-1, R_w, R_w/d^v))\right) &(32)\\
&\ge \text{Vol}(R_w\mathbb{S}^{d-1})\left(1 - Cd^{1/2-s}\right) &(33)\\
&\ge \frac{1}{2}\text{Vol}(R_w\mathbb{S}^{d-1}), &(34)
\end{aligned}$$

where in the last line we used $s > 3/2$ and d sufficiently large. Thus, it follows that

$$\text{Vol}(\mathcal{S}_w) \ge \frac{\text{Vol}(R_w\mathbb{S}^{d-1})}{2^{d+1}}. \tag{35}$$

In order to lower bound $|\tilde{\mathcal{S}}_w|$, we use a lower bound in terms of the ratio of $\text{Vol}(\mathcal{S}_w)$ and the volume of a spherical cap with angle $0.51/2 = 0.255$ (see, e.g., Proposition 4.2.12 of [19]). This gives

$$|\tilde{\mathcal{S}}_w| \ge \frac{\text{Vol}(\mathcal{S}_w)}{\text{Vol}(\text{Cap}(d, R_w, 0.255))} \tag{36}$$

$$\geq \frac{\mathrm{Vol}(R_w \mathbb{S}^{d-1})}{2^{d+1}\mathrm{Vol}(\mathrm{Cap}(d, R_w, 0.255))} \tag{37}$$

$$= \frac{1}{2^d I_{\sin^2(0.255)}(d/2, 1/2)} \tag{38}$$

$$= \frac{B(d/2, 1/2)}{2^d \int_0^{\sin^2(0.255)} t^{d/2-1}(1-t)^{-1/2}\, dt} \tag{39}$$

$$\geq \frac{cd^{-1/2}}{2^d \int_0^{\sin^2(0.255)} t^{d/2-1}(1-t)^{-1/2}\, dt} \tag{40}$$

$$\geq \frac{c'd^{-1/2}}{2^d \int_0^{\sin^2(0.255)} t^{d/2-1}\, dt} \tag{41}$$

$$\geq \frac{c'\sqrt{d}}{2} \frac{1}{(2\sin(0.255))^d} \tag{42}$$

$$\geq e^{c''d}. \tag{43}$$

In the second line, we use Equation (35). In the third line, we use Equation (20). In the fourth line, we use the definition of $I_x(a, b)$. In the fifth line, we use Equation (29). In the sixth line, we redefine the constant by absorbing a factor of $1/(1 - \sin^2(0.255))^{-1/2}$. Finally, in the last line, we assume that d is sufficiently large in order to absorb the polynomial factor in d and use that $2\sin(0.255) < 1$. □

With this result, we can prove Lemma 2.

Proof of Lemma 2. We follow the proof of Lemma 5 in [12] but make appropriate changes. Note that Lemma 2 from [12] proves that for any w,

$$\widehat{g_{w^\star}\varphi}(x) = \sum_{z\in\mathbb{Z}} a_z \cdot \hat{\varphi}(x - zw^\star), \tag{44}$$

where a_z are complex coefficients corresponding to the Fourier series expansion of $\tilde{g}$. Using this, we can write

$$\mathop{\mathbb{E}}_{w^\star\sim\tilde{\mathcal{S}}_w}\left[\left(\langle h, \widehat{g_{w^\star}\varphi}\rangle - a_0\langle h, \hat{\varphi}\rangle\right)^2\right] = \mathop{\mathbb{E}}_{w^\star\sim\tilde{\mathcal{S}}_w}\left[\left(\left\langle h, \sum_{z\in\mathbb{Z}} a_z\hat{\varphi}(\cdot - zw^\star)\right\rangle - a_0\langle h, \hat{\varphi}\rangle\right)^2\right] \tag{45}$$

$$= \mathop{\mathbb{E}}_{w^\star\sim\tilde{\mathcal{S}}_w}\left[\left\langle h, \sum_{z\in\mathbb{Z}\setminus\{0\}} a_z\hat{\varphi}(\cdot - zw^\star)\right\rangle^2\right]. \tag{46}$$

For any $w \in \tilde{\mathcal{S}}_w$, define

$$A_{w,r} \triangleq \{x \in \mathbb{R}^d : \exists z \in \mathbb{Z}\setminus\{0\} \text{ s.t. } \|x - zw\|_2 < r\}. \tag{47}$$

Let $\mathbb{1}_{A_{w,r}}$ denote the indicator function to the set $A_{w,r}$ and $\mathbb{1}_{A^C_{w,r}}$ denote the indicator of its complement. Using that $(a+b)^2 \leq 2(a^2+b^2)$, we can upper bound our previous expression by

$$\mathop{\mathbb{E}}_{w^\star\sim\tilde{\mathcal{S}}_w}\left[\left(\langle h, \widehat{g_{w^\star}\varphi}\rangle - a_0\langle h, \hat{\varphi}\rangle\right)^2\right] \tag{48}$$

$$\leq 2\mathop{\mathbb{E}}_{w^\star\sim\tilde{\mathcal{S}}_w}\left[\left\langle h, \mathbb{1}_{A_{w^\star,R_w/4}} \sum_{z\in\mathbb{Z}\setminus\{0\}} a_z\hat{\varphi}(\cdot - zw^\star)\right\rangle^2\right] + 2\mathop{\mathbb{E}}_{w^\star\sim\tilde{\mathcal{S}}_w}\left[\left\langle h, \mathbb{1}_{A^C_{w^\star,R_w/4}} \sum_{z\in\mathbb{Z}\setminus\{0\}} a_z\hat{\varphi}(\cdot - zw^\star)\right\rangle^2\right]. \tag{49}$$

Note that this is slightly different from the proof in [12], where we use the set $A_{w,R_w/4}$ instead of $A_{w,R_w/2}$. This is because, as we show shortly, for $w \in \tilde{\mathcal{S}}_w$, the sets $A_{w,R_w/4}$ are disjoint. In contrast, for the set $\mathcal{W}$ chosen in [12], $A_{w,R_w/2}$ are disjoint instead.

First, let us show that $A_{w,R_w/4}$ are disjoint for $w \in \tilde{\mathcal{S}}_w$. Suppose for the sake of contradiction that the $A_{w,R_w/4}$ are not disjoint, i.e., there exists some $x \in \mathbb{R}^d$ such that $\|x - zw\|_2 < R_w/4$ and $\|x - z'w'\|_2 < R_w/4$ for $z, z' \in \mathbb{Z}\setminus\{0\}$ and $w, w' \in \tilde{\mathcal{S}}_w$. By triangle inequality, we have

$$\|zw - z'w'\|_2 \leq \|x - zw\|_2 + \|x - z'w'\|_2 \leq R_w/2. \tag{50}$$

Since w, w' are both in $R_w\mathbb{S}^{d-1} \cap \mathbb{R}^d_+$, if the signs of z and z' are different, then the angle between the segments wz and wz' is greater than $\pi/2$. This implies that the cosine of this angle θ is negative. Since $\|zw\|_2 \geq R_w$ and $\|z'w'\|_2 \geq R_w$, this implies

$$\|zw - z'w'\|_2^2 = \|zw\|_2^2 + \|z'w'\|_2^2 - 2\|z'w\|_2\|z'w'\|_2 \cos\theta \tag{51}$$

$$\geq \|zw\|_2^2 + \|z'w'\|_2^2 \tag{52}$$

$$\geq R_w^2, \tag{53}$$

which contradicts Equation (50). Thus, we can henceforth assume that the signs of z, z' are the same.

Note that $\|x - zw\|_2 < R_w/4$ implies that x lies on a spherical shell of width $R_w/2$, centered at radius zR_w. Similarly, $\|x - z'w'\|_2 < R_w/4$ implies that x lies on a spherical shell of width $R_w/2$ centered at radius $z'R_w$. Since these do not intersect when $z \neq z'$, there can be no such x in the intersection of these two sets.

Finally, it remains to consider the case of $z = z'$. Note that

$$\|zw - zw'\|_2^2 \geq \|w - w'\|_2^2. \tag{54}$$

From the definition of $\tilde{\mathcal{S}}_w$ as a 0.51-packing net of $\mathcal{S}_w$, we then have

$$\sin\frac{0.51}{2} = \frac{\|w - w'\|_2}{2R_w}. \tag{55}$$

This implies that

$$\|w - w'\|_2 \geq 2R_w \sin\frac{0.51}{2} > \frac{R_w}{2}, \tag{56}$$

contradicting Equation (50). It follows that no such x can exist, and thus $A_{w,R_w/4}$ are disjoint for all $w \in \tilde{\mathcal{S}}_w$.

Now, using this, we want to bound the expression in Equation (49). For the first term in Equation (49), the same argument as in [12] holds for our case. We reproduce the argument here.

$$\mathop{\mathbb{E}}_{w^\star \sim \tilde{\mathcal{S}}_w}\left[\left\langle h, \mathbb{1}_{A_{w^\star,R_w/4}} \sum_{z\in\mathbb{Z}\setminus\{0\}} a_z\hat{\varphi}(\cdot - zw^\star)\right\rangle^2\right] \tag{57}$$

$$= \mathop{\mathbb{E}}_{w^\star \sim \tilde{\mathcal{S}}_w}\left[\left\langle h, \mathbb{1}_{A_{w^\star,R_w/4}}\left(\widehat{g_{w^\star}\varphi} - a_0\hat{\varphi}\right)\right\rangle^2\right] \tag{58}$$

$$= \mathop{\mathbb{E}}_{w^\star \sim \tilde{\mathcal{S}}_w}\left[\left\langle \mathbb{1}_{A_{w,R_w/4}} h, \widehat{g_{w^\star}\varphi} - a_0\hat{\varphi}\right\rangle^2\right] \tag{59}$$

$$\leq \mathop{\mathbb{E}}_{w^\star \sim \tilde{\mathcal{S}}_w}\left[\left\|\mathbb{1}_{A_{w^\star,R_w/4}} h\right\|_2^2 \|\widehat{g_{w^\star}\varphi} - a_0\hat{\varphi}\|_2^2\right] \tag{60}$$

$$\leq 2 \mathop{\mathbb{E}}_{w^\star \sim \tilde{\mathcal{S}}_w}\left[\left\|\mathbb{1}_{A_{w^\star,R_w/4}} h\right\|_2^2 \left(\|\widehat{g_{w^\star}\varphi}\|_2^2 + \|a_0\hat{\varphi}\|_2^2\right)\right] \tag{61}$$

$$= 2 \mathop{\mathbb{E}}_{w^\star \sim \tilde{\mathcal{S}}_w}\left[\left\|\mathbb{1}_{A_{w^\star,R_w/4}} h\right\|_2^2 \left(\|g_{w^\star}\varphi\|_2^2 + |a_0|^2\|\hat{\varphi}\|_2^2\right)\right] \tag{62}$$

$$\leq 4 \mathop{\mathbb{E}}_{w^\star \sim \tilde{\mathcal{S}}_w}\left[\left\|\mathbb{1}_{A_{w^\star,R_w/4}} h\right\|_2^2\right] \tag{63}$$

$$\leq \frac{4}{|\tilde{\mathcal{S}}_w|} \sum_{w^\star \in \tilde{\mathcal{S}}_w} \int \mathbb{1}_{A_{w^\star,R_w/4}} |h(x)|^2\, dx \tag{64}$$

$$= \frac{4}{|\tilde{\mathcal{S}}_w|} \int \left(\sum_{w^\star \in \tilde{\mathcal{S}}_w} \mathbb{1}_{A_{w^\star,R_w/4}}\right) |h(x)|^2\, dx \tag{65}$$

$$\leq \frac{4}{|\tilde{\mathcal{S}}_w|} \int |h(x)|^2\, dx \tag{66}$$

$$\leq 4e^{-cd}\|h\|_2^2, \tag{67}$$

where in the second line, we use Equation (44). In the fourth line, we use the Cauchy-Schwarz inequality. In the seventh line, we use that $\|\hat{\varphi}\|_2 = \|\varphi\|_2 = 1$, $|a_0|^2 \leq \sum_z |a_z|^2 \leq 1$, and

$$\|g_{w^\star}\varphi\|_2^2 = \int g_{w^\star}^2(x)\varphi^2(x)\, dx \leq \int \varphi^2(x)\, dx = 1. \tag{68}$$

In the second to last line, we use that $A_{w,R_w/4}$ are disjoint sets for $w \in \tilde{\mathcal{S}}_w$, as previously argued, so that $\sum_{w\in\tilde{\mathcal{S}}_w} \mathbb{1}_{A_{w,R_w/4}}(x) \le 1$ for any x. The last line follows by Lemma 3.

Finally, it remains to bound the second term in Equation (49). We will upper bound the expression deterministically for any $w^\star$, so we may drop the expectation By Cauchy-Schwarz,

$$\left\langle h, \mathbb{1}_{A^C_{w^\star,R_w/4}} \sum_{z\in\mathbb{Z}\setminus\{0\}} a_z \hat{\varphi}(\cdot - zw^\star) \right\rangle^2 \le \|h\|_2^2 \cdot \left\| \mathbb{1}_{A^C_{w^\star,R_w/4}} \sum_{z\in\mathbb{Z}\setminus\{0\}} a_z \hat{\varphi}(\cdot - zw^\star) \right\|_2^2 \tag{69}$$

$$= \|h\|_2^2 \left(\sum_{z_1,z_2\in\mathbb{Z}\setminus\{0\}} a_{z_1} a^*_{z_2} \langle \mathbb{1}_{A^C_{w^\star,R_w/4}} \hat{\varphi}(\cdot - z_1 w^\star), \hat{\varphi}(\cdot - z_2 w^\star) \rangle \right). \tag{70}$$

First, consider the terms in the above sum with $z_1 = z_2$. Then, we have

$$\langle \mathbb{1}_{A^C_{w^\star,R_w/4}} \hat{\varphi}(\cdot - z_1 w^\star), \hat{\varphi}(\cdot - z_2 w^\star) \rangle = \int \mathbb{1}_{A^C_{w^\star,R_w/4}} |\hat{\varphi}(x - z_1 w^\star)|^2 \, dx \tag{71}$$

$$= \int \mathbb{1}_{A^C_{w^\star,R_w/4}} (x + z_1 w^\star) |\hat{\varphi}(x)|^2 \, dx \tag{72}$$

$$\le \int_{x:\|x\|_2 \ge R_w/4} |\hat{\varphi}(x)|^2 \, dx \tag{73}$$

$$\le \epsilon^2(R_w/4). \tag{74}$$

Here, the third line follows by definition of $A^C_{w^\star,R_w/4}$ and the assumption that $z_1 \neq 0$ so that $\mathbb{1}_{A^C_{w^\star,R_w/4}}(x+z_1 w^\star) = 1$ only if $\|x\|_2 \ge R_w/4$. The last line follows since φ is $\epsilon(r)$-Fourier-concentrated.

For terms such that $z_1 \neq z_2$, the exact same argument as in [12] holds, so we do not reproduce it here. This gives a bound of

$$\sum_{\substack{z_1,z_2\in\mathbb{Z}\setminus\{0\} \\ z_1 \neq z_2}} a_{z_1} a^*_{z_2} \langle \mathbb{1}_{A^C_{w^\star,R_w/4}} \hat{\varphi}(\cdot - z_1 w^\star), \hat{\varphi}(\cdot - z_2 w^\star) \rangle \le 4 \sum_{n=1}^{\infty} \epsilon(nR_w/2). \tag{75}$$

Thus, putting everything together, we have

$$\left\langle h, \mathbb{1}_{A^C_{w^\star,R_w/4}} \sum_{z\in\mathbb{Z}\setminus\{0\}} a_z \hat{\varphi}(\cdot - zw^\star) \right\rangle^2 \le \|h\|_2^2 \left(\epsilon^2(R_w/4) + 4 \sum_{n=1}^{\infty} \epsilon(nR_w/2) \right) \le 5 \|h\|_2^2 \sum_{n=1}^{\infty} \epsilon(nR_w/4), \tag{76}$$

where we used the fact that $\epsilon^2(R_w/4) \le \epsilon^2(R_w/4) \le \sum_{n=1}^{\infty} \epsilon(nR_w/4)$ and ϵ is a non-increasing function for distributions of interest. Together with Equation (67), plugging into Equation (49), we obtain the claim. □

B. Correlational SQ lower bound

We extend the classical hardness argument from the previous section to hold against any classical algorithm utilizing correlational SQs. Recall from Section I A that in the correlational SQ model [20, 21], queries are restricted to acting only on the input space, i.e., for a query ϕ, algorithms receive estimates of $\mathbb{E}_{x\sim\mathcal{D}}[\phi(x) g_{w^\star}(x)]$. In this section, we focus on Gaussian distributions and prove hardness for the simplest case of when $\tilde{g}$ consists of a single cosine. This clearly implies hardness for the more general $\tilde{g}$ considered in the remainder of the paper. We prove the following theorem.

Theorem 2 (Correlational SQ Hardness)**.** *Let $\epsilon \in (0,1)$. Let $\mathcal{C} = \{x \mapsto \cos(2\pi x^\intercal w^\star) : w^\star \in \tilde{\mathcal{S}}_w \subseteq \mathbb{R}^d\}$ be a concept class, where $\tilde{\mathcal{S}}_w$ is a 0.51-packing net of the set $\mathcal{S}_w$ given in Equation (9). Consider a Gaussian distribution $\mathcal{D}$ with a diagonal covariance matrix $\Sigma = \sigma^2 I$, where $\sigma \ge \tilde{\Omega}(d^2)$. Then, any classical algorithm using correlational SQs to learn an unknown $c^\star \in \mathcal{C}$ with respect to the distribution $\mathcal{D}$ requires at least $2^{\Omega(d)}$ queries of tolerance $\mathcal{O}(1/d^4)$ to learn $\mathcal{C}$ to error ϵ.*

First, we note that while some of the parameters, e.g., the variance and tolerance, appear arbitrary, we have in fact carefully chosen these to align with the parameters of our quantum algorithm. Namely,

$\sigma \geq \tilde{\Omega}(d^2)$ is also satisfied for our efficient quantum algorithm solving this problem via Corollary 9. Also, as seen in Theorem 8, a QSQ tolerance of $\mathcal{O}(1/d^4)$ is also sufficient for our quantum algorithm to learn successfully[3].

Moreover, this lower bound only holds against classical algorithms using *correlational* SQs, rather than general SQs. In the case of Boolean functions, these two models are in fact equivalent [20]. However, for real functions, there exist separations between correlational SQs and general SQs [22–24]. Nevertheless, Theorem 2 is a strengthening of the classical hardness proven in Section III A, and we view it as an important step towards general SQ hardness. Additionally, it is interesting to observe that only one type of query made by our quantum algorithm is not a "correlational QSQ." Correlational QSQs have not been studied before in the literature, but a clear natural analogue is that queried observables O can only act on the input register, i.e., $O = O_1 \otimes I$, where the identity acts on the output register. Then, the only queries that our quantum algorithms in Sections IV and V make that are not correlational in this sense are of the form given in Equation (140). As all other QSQs are correlational, considering classical algorithms with access only to correlational SQs is arguably not significantly restrictive.

Previously, classical learning theorists have shown similar correlational SQ lower bounds for learning single-layer neural networks [25, 26]. However, the functions for which they show hardness of learning are not the quite same as those considered here. In particular, they differ in the activation function (cosine vs. ReLu), and also do not restrict the valid affine functions to $w^\star \in \tilde{\mathcal{S}_w}$. Thus, the above theorem does not follow immediately from existing results and needs to be analyzed separately.

One way to prove correlational SQ lower bounds is via the *statistical dimension* [16, 27, 28], which captures the difficulty of learning a concept class, similarly to the more commonly known VC dimension. Informally, the statistical dimension quantifies the size of the largest subset of the concept class whose elements have "low correlation." More precisely, we state the following definition, following the presentation of [25].

Definition 4 (Statistical dimension)**.** *Let $\mathcal{C}$ be a concept class, and let $\mathcal{D}$ be a distribution on the same domain as functions in $\mathcal{C}$. Define the* average correlation *of $\mathcal{C}$ as*

$$\rho_{\mathcal{D}}(\mathcal{C}) \triangleq \frac{1}{|\mathcal{C}|^2} \sum_{c,c' \in \mathcal{C}} \left| \mathop{\mathbb{E}}_{x\sim\mathcal{D}}[c(x)c'(x)] \right|. \tag{77}$$

Then, the statistical dimension *of $\mathcal{C}$ at threshold γ, denoted* $\mathrm{SDA}_{\mathcal{D}}(\mathcal{C},\gamma)$ *is the largest A such that for all $\mathcal{C}' \subseteq \mathcal{C}$ with $|\mathcal{C}'| \geq |\mathcal{C}|/A$, then $\rho_{\mathcal{D}}(\mathcal{C}') \leq \gamma$.*

Intuitively, functions in the subset $\mathcal{C}'$ have low correlation and thus should be hard to distinguish and hard to learn. This fundamental relationship between the statistical dimension and SQ lower bounds has been formalized [28–30]. We state a version of this result, following the presentation of [25].

Theorem 3 (Theorem 4.1 in [25])**.** *Let $\epsilon \in (0,1)$, and let $\gamma > 0$. Let $\mathcal{C}$ be a concept class, and let $\mathcal{D}$ be a distribution on the same domain as functions in $\mathcal{C}$. Suppose that $\mathbb{E}_{x\sim\mathcal{D}}[c^2(x)] > \epsilon^2$ for all $c \in \mathcal{C}$. Let $A = \mathrm{SDA}_{\mathcal{D}}(\mathcal{C},\gamma)$. Then, any SQ learning making only correlational SQs to some unknown $c \in \mathcal{C}$ requires at least $\Omega(A)$ queries of tolerance $\sqrt{\gamma}$ to learn $\mathcal{C}$ up to error ϵ.*

Theorem 2 follows as a consequence of this theorem. In order to apply it, we first need to lower bound the statistical dimension of our concept class.

Lemma 4. *Let $\mathcal{C} = \{x \mapsto \cos(2\pi x^\intercal w^\star) : w^\star \in \tilde{\mathcal{S}}_w \subseteq \mathbb{R}^d\}$ be a concept class, where $\tilde{\mathcal{S}}_w$ is a 0.51 packing net of the set $\mathcal{S}_w$ given in Equation (9). Let $\mathcal{D}$ be a Gaussian distribution with a digaonal covariance matrix $\Sigma = \sigma^2 I$, where $\sigma \geq \tilde{\Omega}(d^2)$. Then, $\mathrm{SDA}_{\mathcal{D}}(\mathcal{C},\gamma) \geq 2^{\Omega(d)}$ for $\gamma = \mathcal{O}(1/d^8)$.*

Proof. By definition of the statistical dimension, we need to consider the average correlation. In particular, we need to control expectations of the form

$$\mathop{\mathbb{E}}_{x\sim\mathcal{D}}[\cos(2\pi x^\intercal w^\star)\cos(2\pi x^\intercal v^\star)]. \tag{78}$$

Using the sum-product formula for cosines, we have

$$\mathop{\mathbb{E}}_{x\sim\mathcal{D}}[\cos(2\pi x^\intercal w^\star)\cos(2\pi x^\intercal v^\star)] = \frac{1}{2}\mathop{\mathbb{E}}_{x\sim\mathcal{D}}[\cos(2\pi x^\intercal (w^\star + v^\star))] + \frac{1}{2}\mathop{\mathbb{E}}_{x\sim\mathcal{D}}[\cos(2\pi x^\intercal (w^\star - v^\star))]. \tag{79}$$

---

[3] The scaling for the variance can be seen by $\sigma \geq \tilde{\Omega}(\tau M_1^2 d^4/R_w^2)$, $M_1 \geq R_w^2/\epsilon_1 \geq R_w^2 d$ and taking $\tau = \mathcal{O}(1/(d^4 R_w^2))$. These conditions are all satisfied by the choices of parameters in Theorem 8 and Corollary 9. To be precise, as stated, the tolerance of the classical hardness only matches the QSQ tolerance with respect to the d scaling. The proof can be extended in the same way such that the tolerances match precisely, but we focus on d scaling for simplicity.

Now, to evaluate these expectations, recall the definition of a characteristic function $\varphi(t) = \mathbb{E}[e^{-it^\intercal X}]$, where X is a random vector. When the distribution of X is a multivariate normal distribution with mean vector μ and covariance matrix Σ, it is well known that the characteristic function is $\varphi(t) = e^{it^\intercal \mu - t^\intercal \Sigma t/2}$. In our case, this simplifies to $\varphi(t) = e^{-\sigma^2 \|t\|_2^2/2}$. Meanwhile, expanding the definiton of $\varphi(t)$, we have

$$\varphi(t) = \mathbb{E}[e^{-it^\intercal X}] = \mathbb{E}[\cos(t^\intercal X) - i\sin(t^\intercal X)]. \tag{80}$$

Setting this equal to the known expression for Gaussian distributions, i.e., $\varphi(t) = e^{-\sigma^2\|t\|_2^2/2}$, then notice that this expression is only real. Thus, one can conclude that the imaginary part of the characteristic function is zero and hence

$$\mathbb{E}[\cos(t^\intercal X)] = e^{-\sigma^2\|t\|_2^2/2} \tag{81}$$

for a random vector X distributed according to a multivariate Gaussian. Plugging this into our previous expression, we have

$$\left|\mathop{\mathbb{E}}_{x\sim\mathcal{D}}[\cos(2\pi x^\intercal w^\star)\cos(2\pi x^\intercal v^\star)]\right| \leq \frac{1}{2}e^{-\sigma^2\|2\pi(w^\star+v^\star)\|_2^2/2} + \frac{1}{2}e^{-\sigma^2\|2\pi(w^\star-v^\star)\|_2^2/2}. \tag{82}$$

It remains to lower bound these two norms. This can be done via a simple calculation while using the fact that $w^\star, v^\star \in \tilde{\mathcal{S}}_w$, which is a 0.51-packing net of $\mathcal{S}_w$. In particular, this means that each vector in $\tilde{\mathcal{S}}_w$ is separated by a geodesic angle of at least 0.51, which implies that $|(w^\star)^\intercal v^\star| \leq R_w^2\cos(0.51)$. Hence, we have

$$\begin{aligned}
\|2\pi(w^\star+v^\star)\|_2^2 &= (2\pi)^2(\|w^\star\|_2^2 + \|v^\star\|_2^2 + 2(w^\star)^\intercal v^\star) && (83)\\
&\geq (2\pi)^2(2R_w^2 - 2R_w^2\cos(0.51)) && (84)\\
&= 2(2\pi R_w)^2(1-\cos(0.51)) && (85)\\
&= (4\pi R_w)^2\sin^2(0.255) && (86)
\end{aligned}$$

and similarly

$$\begin{aligned}
\|2\pi(w^\star-v^\star)\|_2^2 &= (2\pi)^2(\|w^\star\|_2^2 + \|v^\star\|_2^2 - 2(w^\star)^\intercal v^\star) && (87)\\
&\geq (2\pi)^2(2R_w^2 - 2R_w^2\cos(0.51)) && (88)\\
&= (4\pi R_w)^2\sin^2(0.255). && (89)
\end{aligned}$$

Thus, we have

$$\left|\mathop{\mathbb{E}}_{x\sim\mathcal{D}}[\cos(2\pi x^\intercal w^\star)\cos(2\pi x^\intercal v^\star)]\right| \leq e^{-\frac{1}{2}\sigma^2(4\pi R_w)^2\sin^2(0.255)} \leq e^{-\tilde{O}(d^4R_w^2)}, \tag{90}$$

where in the last inequality, we use our condition that $\sigma \geq \tilde{\Omega}(d^2)$. Using this, we can bound the average correlation. Here, we write $g_{w^\star}(x) = \cos(2\pi x^\intercal w^\star)$.

$$\rho_{\mathcal{D}}(\mathcal{C}') = \frac{1}{|\mathcal{C}'|^2}\sum_{g_{w^\star},g_{v^\star}\in\mathcal{C}'}\left|\mathop{\mathbb{E}}_{x\sim\mathcal{D}}[g_{w^\star}(x)g_{v^\star}(x)]\right| \leq \frac{1}{|\mathcal{C}'|^2}\left(|\mathcal{C}'| + \sum_{\substack{g_{w^\star},g_{v^\star}\in\mathcal{C}'\\ w^\star\neq v^\star}} e^{-\tilde{O}(d^4R_w^2)}\right) \leq \frac{1}{|\mathcal{C}'|} + e^{-\tilde{O}(d^4R_w^2)}. \tag{91}$$

This is less than $\mathcal{O}(1/d^8)$ if $|\mathcal{C}'| \geq d^8$. The size of $\mathcal{C}'$ is in turn greater than $|\mathcal{C}|/A = e^{cd}/A$, where A is the statistical dimension when $A \geq e^{cd}/d^8 = 2^{\Omega(d)}$. This completes the proof of the lemma. □

With this, we can prove Theorem 2 by applying Theorem 3.

Proof of Theorem 2. Now that we have a lower bound on the statistical dimension, it remains to check the conditions of Theorem 3 and apply the theorem. We only need to check the condition that $\mathbb{E}_{x\sim\mathcal{D}}[\cos^2(2\pi x^\intercal w^\star)] > \epsilon^2$ for all $w^\star \in \tilde{\mathcal{S}}_w$. This follows easily by the same manipulations as in the proof of Lemma 4.

$$\mathop{\mathbb{E}}_{x\sim\mathcal{D}}[\cos^2(2\pi x^\intercal w^\star)] = \mathop{\mathbb{E}}_{x\sim\mathcal{D}}[1+\cos(4\pi x^\intercal w^\star)] = 1 + e^{-\sigma^2\|4\pi w^\star\|_2^2/2} = 1 + e^{-(4\pi\sigma)^2R_w^2/2}. \tag{92}$$

This is clearly greater than ϵ^2 for any $\epsilon \in (0,1)$. Thus, by Theorem 3 and Lemma 4, then the theorem clearly follows. □

IV. SUPPLEMENTARY NOTE 4 - UNIFORM DATA DISTRIBUTION

In this section, we consider learning our concept class defined in Section II when given QSQ access to quantum example states with respect to the uniform distribution.

Theorem 4 (Guarantee; Uniform Case)**.** *Let $\epsilon, \delta > 0, \tau \geq 0$. Let φ^2 be the uniform distribution. Let $w^\star \in \mathbb{R}^d$ be unknown with norm $R_w > 0$ and $w_j^\star \geq R_w/d^2$, for all $j \in [d]$. Let $g_{w^\star} : \mathbb{R}^d \to [-1,1]$ be defined as $g_{w^\star}(x) = \tilde{g}(x^\intercal w^\star)$, where $\tilde{g} : \mathbb{R} \to [-1,1]$ is a function defined in Equation (10). Consider parameters $M_1 = \max(70\pi d D^3 R_w, R_w^2/\epsilon_1)$, $M_2 = cM_1$, where c is any constant such that M_2 is an integer and $c < 1/(8\pi D R_w)$, and*

$$\tilde{R} = \tilde{\Omega}\left(\max\left(\frac{\tau M_1^2 d^4}{R_w^2}, \frac{D^2}{\epsilon}, \frac{D^2\sqrt{d}}{R_w \epsilon}, \frac{D^{5/2}}{\sqrt{\epsilon}}, \frac{D^{3/2}\sqrt{d}}{R_w\sqrt{\epsilon}}\right)\right), \quad \epsilon_1 = \tilde{\mathcal{O}}\left(\min\left(\frac{\epsilon^3}{D^6 d}, \frac{\epsilon^{3/2}}{D^{13/2} d}, \frac{R_w}{D\sqrt{d}}\right)\right) \tag{93}$$

Suppose we have QSQ access (see Definition 1) with respect to discretization parameters $M_{1,m} \triangleq mM_1$, $M_{2,m} \triangleq mM_2$ and a truncation parameter $R \geq \tilde{R}$, for $m \in \{1, \ldots, D\}$. Then, there exists a quantum algorithm with this QSQ access that can efficiently find parameters $\hat{\beta} \in \mathbb{R}^D$ such that $\mathcal{L}_{w^\star}(\hat{\beta}) \leq \epsilon$ with probability at least $1 - \delta$. Moreover, this algorithm uses

$$N = \mathcal{O}\left(dD \log\left(\frac{1}{\delta}\right) \log^5\left(\frac{M_1 d^2}{R_w}\right)\right) \tag{94}$$

quantum statistical queries with tolerance $\tau \leq \min\left(\frac{1}{M_2^2}\left(\frac{7}{40D} - \frac{1}{M_2}\right), \frac{1}{2D^2 M_2^2}\left(\frac{2}{15} - \frac{1}{8}\left(\frac{2\pi R_w}{M_1}\right)^2 + \frac{2D^2}{M_2}\right)\right)$ *and*

$$t = \Theta\left(\log\left(\sqrt{\frac{D}{\epsilon}}\right)\right) \tag{95}$$

iterations of gradient descent.

In particular, our algorithm uses QSQs with different choices of discretization/truncation parameters for the different parts of Hallgren's algorithm (Section I B). Recall that Hallgren's algorithm has two subroutines: quantum Fourier sampling and a verification procedure. In the quantum Fourier sampling part, we use QSQs with respect to discretization parameters M_1, M_2 and truncation parameter $R = \tilde{R}$. For verification, we use discretization parameters $M_{1,m} \triangleq mM_1, M_{2,m} \triangleq mM_2$ and truncation parameter $R = \tilde{R}M_{1,m}$ for $m \in \{1, \ldots, D\}$.

Recall that our target functions $g_{w^\star}(x)$ have the nice property that they are periodic with period $e_j/|w_j^\star| = e_j/w_j^\star$ (since $w_j > 0$), where e_j is the unit vector for coordinate $j \in [d]$:

$$g_{w^\star}\left(x + \frac{e_j}{w_j^\star}\right) = \tilde{g}\left(\left(x + \frac{e_j}{w_j^\star}\right)^\intercal w^\star\right) = \tilde{g}\left(x^\intercal w^\star + 1\right) = \tilde{g}(x^\intercal w^\star) = g_{w^\star}(x). \tag{96}$$

The second to last equality holds because $\tilde{g}$ is periodic with period 1. In other words, each individual coordinate of $g_{w^\star}$ is periodic with period $1/w_j^\star$. To quantumly learn $g_{w^\star}$, then we can first perform period finding to find $w^\star$ one component at a time. Then, given the specific form of $\tilde{g}$ (Equation (10)), we can find the parameters $\beta_j^\star$, which can be done via gradient methods, as this is effectively a regression problem.

Despite the simplicity of this algorithm, there are several nontrivial issues that arise. First, recall that the quantum example states are given by Equation (3), which we reproduce below for convenience taking the distribution $\mathcal{D}$ to be the uniform distribution

$$|c^\star\rangle = \frac{1}{\sqrt{|\mathcal{X}|}} \sum_{x \in \mathcal{X}} |x\rangle |c^\star(x)\rangle . \tag{97}$$

We are given access to expectations with respect to these states. In our case, notice that the target function $g_{w^\star} : \mathbb{R}^d \to [-1,1]$ takes inputs and outputs in a continuous and uncountably infinite space. As we should not have a superposition over this large space, we must truncate and discretize our target function. However, discretization can cause a loss of information about the period of the function, which is problematic. Thus, it is important to choose the correct discretization in such a way that information about the period is sufficiently preserved.

In Section IV A, we discuss in detail how to apply period finding to our problem and mitigate these discretization issues. In Section IV B, we show how one can use gradient descent to learn the outer function $\tilde{g}$ given knowledge of $w^\star$.

A. Learning the linear function

In this section, we discuss how to use period finding to learn the inner linear function, i.e., how to learn the vector of coefficients $w^\star$. First, we need to suitably discretize $g_{w^\star}$ such that this discretization satisfies pseudoperiodicity (Definition 2). In Section IV A 1, we consider a simple special case to illustrate the idea behind the discretization and application of period finding for pedagogical purposes. In Section IV A 2, we prove the general case. Throughout, we will assume uniform discretizations of the intervals in the sense that they will be equal size and not adaptively refined in any way. The size of the discretization will be defined to be the number of bins in which the function is represented.

1. *Warmup*

First, let us consider the case of $d = 1$, i.e., the input x to the function and the unknown vector $w^\star$ are both real numbers instead of vectors. We will later generalize this to the case of general $d \geq 1$. The main simplifying assumption made in this section is that $1/w^\star \in \mathbb{Z}$ is an integer. This will allow us to present this step of the algorithm without being hindered by too many approximations in the first instance. If $1/w^\star \in \mathbb{Z}$, we have the following lemma, which tells us the correct discretization that satisfies pseudoperiodicity.

Lemma 5 (Discretization; Simple Case). *Let $w^\star \in \mathbb{R}$ be unknown with $1/w^\star \in \mathbb{Z}$ and $w^\star > 0$. Let $g_{w^\star} : \mathbb{R} \to [-1, 1]$ be defined as $g_{w^\star}(x) = \tilde{g}(xw^\star)$, where $\tilde{g} : \mathbb{R} \to [-1, 1]$ is a function with period 1 which has bounded variation on every finite interval. Let $M \in \mathbb{Z}$ be the size of the discretization. Consider the discretized function $h_{w^\star,M} : \mathbb{Z} \to \frac{1}{M}\mathbb{Z}$ defined by*

$$h_{w^\star,M}(k) = \left\lfloor g_{w^\star}\left(\frac{k}{M}\right)\right\rfloor_M, \tag{98}$$

where $\lfloor\cdot\rfloor_M$ denotes rounding down to the nearest multiple of $1/M$. Then, $h_{w^\star,M}$ is pseudoperiodic (in fact, periodic) with period $M/w^\star$.

Note that for our choice of $\tilde{g}$ as a linear combination of cosines (Equation (10)), the conditions in the lemma are clearly satisfied.

Proof. We need to show that $h_{w^\star,M}(k + [\ell M/w^\star]) = h_{w^\star,M}(k)$ for all $\ell \in \mathbb{Z}$, where we use $[\cdot]$ to denote one of either $\lfloor\cdot\rfloor$ or $\lceil\cdot\rceil$. In fact, because $1/w^\star \in \mathbb{Z}$ and $\ell, N \in \mathbb{Z}$, then $[\ell M/w^\star] = \ell M/w^\star$. Since $\tilde{g}$ is a bounded variation function, it is equal everywhere to its Fourier series expansion:

$$\tilde{g}(x) = \sum_{z\in\mathbb{Z}} a_z e^{2\pi i z x}. \tag{99}$$

Using this to expand out $h_{w^\star,M}(k + \ell M/w^\star)$, we have

$$h_{w^\star,M}(k + \ell M/w^\star) = \left\lfloor g_{w^\star}\left(\frac{k}{M} + \frac{\ell M/w^\star}{M}\right)\right\rfloor_M \tag{100}$$

$$= \left\lfloor \tilde{g}\left(\left(\frac{k}{M} + \frac{\ell}{w^\star}\right) w^\star\right)\right\rfloor_M \tag{101}$$

$$= \left\lfloor \sum_{z\in\mathbb{Z}} a_z \exp\left(2\pi i z\left(\frac{kw^\star}{M} + \ell\right)\right)\right\rfloor_M \tag{102}$$

$$= \left\lfloor \sum_{z\in\mathbb{Z}} a_z \exp\left(\frac{2\pi i z k w^\star}{M}\right) \exp\left(2\pi i z \ell\right)\right\rfloor_M \tag{103}$$

$$= \left\lfloor \sum_{z\in\mathbb{Z}} a_z \exp\left(\frac{2\pi i z k w^\star}{M}\right)\right\rfloor_M \tag{104}$$

$$= \left\lfloor \tilde{g}\left(\frac{kw^\star}{M}\right)\right\rfloor_M \tag{105}$$

$$= \left\lfloor g_{w^\star}\left(\frac{k}{M}\right)\right\rfloor_M \tag{106}$$

$$= h_{w^\star,M}(k). \tag{107}$$

Here, in the fifth line, we use that $z, \ell \in \mathbb{Z}$ so that $\exp(2\pi i z\ell) = 1$. This gives the claim. $\square$

Now, we have a suitable discretization, but we also need to truncate the domain of the function so that it is not all of $\mathbb{Z}$. Let R be this truncation parameter. Then, as defined in Definition 1, we should have QSQ access to the quantum example state with respect to the truncated and discretized target function, i.e.,

$$|h_{w^\star}\rangle = \frac{1}{(2R)^{d/2}} \sum_{x_1,\ldots,x_d=-R}^{R-1} |x\rangle \, |h_{w^\star,M}(x)\rangle \,. \tag{108}$$

Before proving our guarantee on learning $w^\star$, first notice that by the definition of $\mathcal{S}_w$ (Equation (9)), we have an upper bound on the period of $h_{w^\star,M}$. In particular, $w_j^\star \geq R_w/d^2$. Then, the period satisfies

$$\frac{Me_j}{w_j^\star} \leq \frac{Me_j d^2}{R_w}. \tag{109}$$

This is useful for choosing our truncation parameter R in the following result.

Proposition 1 (Linear Function Guarantee; Simple Uniform Case)**.** *Let φ^2 be the uniform distribution. Let $\tau \geq 0$. Let $w^\star \in \mathbb{R}^d$ be unknown with $1/w_j^\star \in \mathbb{Z}$ for all $j \in [d]$. Also suppose that $w_j^\star \geq R_w/d^2$, for all $j \in [d]$. Let $g_{w^\star} : \mathbb{R}^d \to [-1,1]$ be defined as $g_{w^\star}(x) = \tilde{g}(x^\intercal w^\star)$, where $\tilde{g} : \mathbb{R} \to [-1,1]$ is a function with period 1 which has bounded variation on every finite interval. Then, for any choice of discretization parameter $M \geq 1$ and truncation parameter $R \geq (1+2\tau)M^2 d^4/R_w^2$, there exists a quantum algorithm that learns $w^\star$ exactly with constant probability using*

$$N = d \tag{110}$$

quantum statistical queries with tolerance τ (with respect to the discretized and truncated example state).

Proof. We first consider the case of $d = 1$. Consider the state in Equation (108), with respect to which we have access to expectations. By Lemma 5, we know that $h_{w^\star,M}$ is periodic with period $M/w^\star$ via our choice of discretization. With the truncation, $h_{w^\star,M}$ is still periodic with period $M/w^\star$. Moreover, since $1/w^\star \in \mathbb{Z}$ in this simple case, then the period is an integer. Thus, we can simply apply standard period finding [10] to solve for $M/w^\star$, i.e., apply the quantum Fourier transform (QFT) and measure. We can encode this algorithm into an observable O with $\|O\| \leq 1$ as follows

$$O = \left(\mathsf{QFT}_q^{-1} \sum_{\ell \in [M]} \frac{\ell}{M} |\ell\rangle\langle\ell| \, \mathsf{QFT}_q \right) \otimes I. \tag{111}$$

Here, QFT_q denotes the QFT in a dimension of size $q = 2R$ (since the input x is between $-R$ and R due to truncation), and I is the identity operator acting on the qubits encoding the output $h_{w^\star,M}(x)$. O is simply applying a QFT on the first register and measuring these qubits with proper normalization factors to ensure that $\|O\| \leq 1$. By a standard analysis of the period finding algorithm (see, e.g., [31]), if our QSQs were noiseless ($\tau = 0$), this allows us to recover the period $M/w^\star$ exactly with constant success probability using only one (noiseless) QSQ.

However, we consider the case of a general noise tolerance $\tau \geq 0$ for our QSQs. By the standard analysis of period finding, with constant probability, the output of the QSQ is some number α such that $|\alpha - y| \leq \tau$, where $|y - kRw^\star/M| \leq 1/2$ for some integer $k \geq 0$. By the reverse triangle inequality, this implies that $|\alpha - kRw^\star/M| \leq \tau + 1/2$. Then, dividing by R, we see that

$$\left| \frac{\alpha}{R} - \frac{kw^\star}{M} \right| \leq \frac{\tau + 1/2}{R}. \tag{112}$$

Notice that $kw^\star/M$ can be thought of as a fraction with denominator $M/w^\star$ (since $1/w^\star \in \mathbb{Z}$ in this case), which is the period of our target function. Let $A \triangleq Md^2/R_w$ be an upper bound on the period $M/w^\star$. Then, $kw^\star/M$ is a fraction with denominator less than A. Two distinct rational numbers with denominator less than A must be at least a distance of $1/A^2 \geq (1+2\tau)/R$ apart, where the inequality

comes from our choice of $R \geq (1+2\tau)A^2$. Thus, Equation (112) implies that there exists a unique fraction $kw^\star/M$ that is determined by α/R. Moreover, by our choice of R again,

$$\left|\frac{\alpha}{R} - \frac{kw^\star}{M}\right| \leq \frac{\tau + 1/2}{R} \leq \frac{1}{2A^2}. \tag{113}$$

Thus, by standard results for the continued fractions expansion [11], we can recover the unique $kw^\star/M$ from α/R. The rest of the analysis follows in the same way as the usual period finding algorithm. This tells us that we can recover the period $M/w^\star$ exactly with constant probability. Moreover, from $M/w^\star$, we can recover $w^\star$ exactly as well since M is known.

Thus far in this section, we have only considered the case of $d = 1$. Our above discussion is easily generalized to arbitrary $d \geq 1$. In particular, our simplifying assumption is now that $1/w_j^\star \in \mathbb{Z}$ for all $j \in [d]$. Recall that for general d, our target function is $g_{w^\star}(x) = \tilde{g}(x^\intercal w^\star)$, where $\tilde{g} : \mathbb{R} \to [-1,1]$ is again a function with period 1 and now $w^\star \in \mathbb{R}^d$. Then, we can define the discretized function as before but this time $h_{w^\star,M} : \mathbb{Z}^d \to \frac{1}{M}\mathbb{Z}$. By essentially the same proof as Lemma 5, one can show that $h_{w^\star,M}$ is periodic with period $Me_j/w_j^\star$, where e_j is the unit vector for coordinate $j \in [d]$. Then, we can perform period finding one coordinate at a time, encoding in the QSQ operator

$$O_j = \left(\mathsf{QFT}_{j,q}^{-1} \sum_{\ell \in [M]} \frac{\ell}{M} |\ell\rangle\langle\ell| \, \mathsf{QFT}_{j,q}\right) \otimes I_{-j} \otimes I, \tag{114}$$

where $\mathsf{QFT}_{j,q}$ denotes the QFT in a dimension $q = 2R$ acting only on the qubits that encode the jth coordinate of the input x_j. Also, I_{-j} is the identity operator acting on all qubits that do not encode x_j and I is the identity operator acting on the qubits encoding the output of the function. By the same argument as before, applying O_j for each coordinate $j \in [d]$, we can recover the whole vector $w^\star$ exactly. Moreover, this uses $N = d$ QSQs to learn $w^\star$ exactly with constant probability. □

From this learned $w^\star$, we can use classical gradient methods to learn the $\tilde{g}$, assuming it has the form given in Equation (10). This is discussed in Section IV B.

2. General Case

In the previous section, we proved that $w^\star$ can be recovered exactly in a simple case. We presented this first to give an overview of the algorithm without becoming overwhelmed by the technicalities involved for the general case. In this section, we prove the general case, with the formal statement given below.

Theorem 5 (Linear Function Guarantee; Uniform Case)**.** *Let φ^2 be the uniform distribution. Let $1 > \epsilon_1 > 0, \delta > 0, \tau \geq 0$. Let $w^\star \in \mathbb{R}^d$ be unknown with norm $R_w > 0$ and $w_j^\star \geq R_w/d^2$, for all $j \in [d]$. Let $g_{w^\star} : \mathbb{R}^d \to [-1,1]$ be defined as $g_{w^\star}(x) = \tilde{g}(x^\intercal w^\star)$, where $\tilde{g} : \mathbb{R} \to [-1,1]$ is given in Equation (10). Consider parameters $M_1 = \lceil \max(70\pi d D^3 R_w, R_w^2/\epsilon_1)\rceil$, $M_2 = cM_1$, where c is any constant such that M_2 is an integer and $c < 1/(8\pi D R_w)$, and*

$$\tilde{R} = \tilde{\Omega}\left(\max\left(\frac{\tau M_1^2 d^4}{R_w^2}, \frac{D^2}{\epsilon}, \frac{D^2\sqrt{d}}{R_w \epsilon}, \frac{D^{5/2}}{\sqrt{\epsilon}}, \frac{D^{3/2}\sqrt{d}}{R_w\sqrt{\epsilon}}\right)\right). \tag{115}$$

Suppose we have QSQ access (Definition 1) with respect to discretization parameters $M_{1,m} \triangleq mM_1$, $M_{2,m} \triangleq mM_2$, and a truncation parameter $R \geq \tilde{R}$, for $m \in \{1, \ldots, D\}$. Then, there exists a quantum algorithm with this QSQ access that can learn an approximation $\hat{w}$ of $w^\star$ such that $\|\hat{w} - w^\star\|_\infty \leq \epsilon_1$ with probability at least $1 - \delta$ using

$$N = \mathcal{O}\left(dD \log\left(\frac{1}{\delta}\right) \log^5\left(\frac{M_1 d^2}{R_w}\right)\right) \tag{116}$$

quantum statistical queries with tolerance $\tau \leq \min\left(\frac{1}{M_2^2}\left(\frac{7}{40D} - \frac{1}{M_2}\right), \frac{1}{2D^2M_2^2}\left(\frac{2}{15} - \frac{1}{8}\left(\frac{2\pi R_w}{M_1}\right)^2 + \frac{2D^2}{M_2}\right)\right)$.

As stated before, our algorithm has two subroutines as in Hallgren's algorithm: quantum Fourier sampling and the verification procedure. For quantum Fourier sampling, we use QSQs with respect to discretization parameters M_1, M_2 and truncation parameter $R = \tilde{R}$. For verification, we use discretization parameters $M_{1,m} \triangleq mM_1, M_{2,m} \triangleq mM_2$ and truncation parameter $R = \tilde{R}M_{1,m}$ for $m \in \{1, \ldots, D\}$.

There are two main differences with the presentation in Section IV A 1. First, in Lemma 5, it was fortuitous that the pseudoperiodicity property (Definition 2) required of the discretization turned out to simply be periodicity under our simplifying assumption. However, in general, this is not the case, so we will need to prove a new version of Lemma 5. Second, even with the discretization, the period of the discretized function may not be an integer in general. Thus, the standard period finding algorithm [10] does not apply. Instead, we turn to a subroutine of Hallgren's algorithm [7] which performs irrational period finding for pseudoperiodic functions. This has some additional conditions that we must fulfill, as discussed in Section I B.

To address the first point, we have the following lemma, which is a generalization of Lemma 5. Again, we first consider $d = 1$ and later generalize to $d \geq 1$. In this case, we need two discretization parameters: one to control the fineness of the discretization of the input to $g_{w^\star}$ and another to control the outer rounding. We consider the case when the latter is more coarse than the former to obtain pseudoperiodicity.

Lemma 6 (Discretization; General Case)**.** *Let $w^\star \in \mathbb{R}$ be unknown with $|w^\star| \leq R_w$ for some $R_w > 0$. Let $g_{w^\star} : \mathbb{R} \to [-1, 1]$ be defined as $g_{w^\star}(x) = \tilde{g}(xw^\star)$, where $\tilde{g} : \mathbb{R} \to [-1, 1]$ is a function with period 1 which has bounded variation on every finite interval, is given by a trigonometric polynomial of degree at most D, and is λ-Lipschitz. Let $M \geq 1$ and consider discretization parameters $M_1 = M$, $M_2 = cM$, where c is any constant such that M_2 is an integer and $c < 1/(4\lambda R_w)$. Consider the discretized function $h_{w^\star,M_1,M_2} : \mathbb{Z} \to \frac{1}{M_2}\mathbb{Z}$ defined by*

$$h_{w^\star,M_1,M_2}(k) = \left\lfloor g_{w^\star}\left(\frac{k}{M_1}\right)\right\rfloor_{M_2}, \tag{117}$$

where $\lfloor\cdot\rfloor_{M_2}$ denotes rounding down to the nearest multiple of $1/M_2$. Then, $h_{w^\star,M_1,M_2}$ is $(1-4DR_w/M)$-pseudoperiodic with period $M_1/w^\star$.

In particular, when $\tilde{g}$ is given by Equation (10) and $M \geq 70\pi dD^3 R_w$, then for discretization parameters $M_1 = M$, $M_2 = cM$ with $c < 1/(8\pi DR_w)$, then $h_{w^\star,M_1,M_2}$ is $(33/35)$-pseudoperiodic with period $M_1/w^\star$.

Proof. We prove the first statement first, so we want to show that $h_{w^\star,M_1,M_2}$ is $(1 - 4DR_w/M)$-pseudoperiodic. In other words, we want to show that $h_{w^\star,M_1,M_2}(k + \lfloor \ell M_1/w^\star\rfloor)$ or $h_{w^\star,M_1,M_2}(k + \lceil \ell M_1/w^\star\rceil)$ equals $h_{w^\star,M_1,M_2}(k)$ for at least a $(1 - 4DR_w/M)$-fraction of the inputs k, for all $\ell \in \mathbb{Z}$. Fixing some $\ell \in \mathbb{Z}$, denote

$$h_+(k) \triangleq h_{w^\star,M_1,M_2}\left(k + \left\lceil\frac{\ell M_1}{w^\star}\right\rceil\right), \quad h_-(k) \triangleq h_{w^\star,M_1,M_2}\left(k + \left\lfloor\frac{\ell M_1}{w^\star}\right\rfloor\right). \tag{118}$$

Also fix some input k such that $0 \leq k \leq \lfloor M/w^\star\rfloor$. Suppose for now that $g_{w^\star}$ is monotonically increasing in the interval $((k-1)M_1, (k+1)M_1)$. We will show that in this case, either $h_+(k) = h_{w^\star,M_1,M_2}(k)$ or $h_-(k) = h_{w^\star,M_1,M_2}(k)$. We have the following upper bound on $h_+(k)$:

$$\begin{aligned}
h_+(k) &= h_{w^\star,M_1,M_2}\left(k + \left\lceil\frac{\ell M_1}{w^\star}\right\rceil\right) && (119)\\
&= \left\lfloor g_{w^\star}\left(\frac{k + \lceil\frac{\ell M_1}{w^\star}\rceil}{M_1}\right) M_2\right\rfloor / M_2 && (120)\\
&= \left\lfloor g_{w^\star}\left(\frac{k + \frac{\ell M_1}{w^\star} + \Delta}{M_1}\right) cM_1\right\rfloor / (cM_1) && (121)\\
&= \left\lfloor g_{w^\star}\left(\frac{k + \Delta}{M_1}\right) cM_1\right\rfloor / (cM_1) && (122)\\
&\leq \left\lfloor g_{w^\star}\left(\frac{k}{M_1}\right) cM_1 + \Delta\lambda w^\star c\right\rfloor / (cM_1) && (123)\\
&\leq \left\lfloor g_{w^\star}\left(\frac{k}{M_1}\right) cM_1 + \lambda w^\star c\right\rfloor / (cM_1). && (124)
\end{aligned}$$

Here, the first line follows by the definition of $h_+(k)$. The second line follows by the definition of $h_{w^\star,M_1,M_2}$. In the third line, we define Δ such that $0 \leq \Delta < 1$ and use $M_2 = cM_1$. In the fourth line, we use that $g_{w^\star}$ has period $1/w^\star$. In the fifth line, because $\tilde{g}$ is λ-Lipschitz, then $g_{w^\star}$ is $(\lambda w^\star)$-Lipschitz. Finally, in the last line, we use that $\Delta < 1$. We can also lower bound $h_+(k)$ using the fourth line of the

above calculation and our assumption that $g_{w^\star}$ is monotonically increasing.

$$h_+(k) = \left\lfloor g_{w^\star}\left(\frac{k+\Delta}{M_1}\right) cM_1 \right\rfloor /(cM_1) \geq \left\lfloor g\left(\frac{k}{M_1}\right) cM_1 \right\rfloor /(cM_1) = h_{w^\star,M_1,M_2}(k). \tag{125}$$

Similarly, one can show that

$$\left\lfloor g_{w^\star}\left(\frac{k}{M_1}\right) cM_1 - \lambda w^\star c \right\rfloor /(cM_1) \leq h_-(k) \leq h_{w^\star,M_1,M_2}(k). \tag{126}$$

Using that $c < 1/(4\lambda R_w)$, then

$$2\lambda w^\star c \leq 2\lambda R_w c \leq \frac{1}{2}. \tag{127}$$

Then,

$$|h_+(k) - h_-(k)| \leq \left| \frac{\lfloor g_{w^\star}(k/M_1)cM_1 + \lambda w^\star c \rfloor}{cM_1} - \frac{\lfloor g_{w^\star}(k/M_1)cM_1 - \lambda w^\star c \rfloor}{cM_1} \right| \tag{128}$$

$$= \left| \frac{g_{w^\star}(k/M_1)cM_1 + \lambda w^\star c - \Delta_+ - g_{w^\star}(k/M_1)cM_1 + \lambda w^\star c + \Delta_-}{cM_1} \right| \tag{129}$$

$$= \left| \frac{2\lambda w^\star c + \Delta_- + \Delta_+}{cM_1} \right| \tag{130}$$

$$\leq \frac{3}{2cM_1}, \tag{131}$$

where in the last line we use that $0 \leq \Delta_-, \Delta_+ < 1$. Because the outputs of $h_+(k)$ and $h_-(k)$ are discretized in steps of $1/(cM_1)$, this implies that

$$|h_+(k) - h_-(k)| \leq \frac{1}{cM_1} = \frac{1}{M_2}. \tag{132}$$

Moreover, by the above work, we know that $h_-(k) \leq h_{w^\star,M_1,M_2}(k) \leq h_+(k)$. Thus, because all three functions have outputs discretized in units of $1/(cM_2)$, it follows that either $h_-(k) = h_{w^\star,M_1,M_2}(k)$ or $h_+(k) = h_{w^\star,M_1,M_2}(k)$. A similar argument holds when $g_{w^\star}$ is instead assumed to be monotonically decreasing in the interval $((k-1)/M_1, (k+1)/M_1)$.

Thus, we have shown that if $g_{w^\star}$ is monotone, then $h_{w^\star,M_1,M_2}$ satisfies the property required for pseudoperiodicity. It suffices to show that $g_{w^\star}$ is monotone in regions $((k-1)/M_1, (k+1)/M_1)$ for all except a $4DR_w/M$-fraction of the inputs k within a single period $0 \leq k \leq \lfloor M_1/w^\star \rfloor$. Note that these intervals are just neighborhoods of size $2/M_1$ centered around some k/M_1 with $0 \leq k \leq \lfloor M_1/w^\star \rfloor$. Thus, we can instead consider neighborhoods of size $2/M_1$ around points k with $0 \leq k \leq 1/w^\star$, i.e., within a single period of $g_{w^\star}$. Note that $g_{w^\star}$ will be monotone in the interval unless it contains a critical point. Thus, it remains to consider neighborhoods of the critical points of $g_{w^\star}$.

By assumption, $\tilde{g}$ is a trigonometric polynomial of degree at most D. Moreover, it is known that trigonometric polynomials with degree at most D have at most $2D$ zeroes in a single period (see, e.g., Chapter 13 of [32]). The derivative of a trigonometric polynomial with degree at most D is clearly still a trigonometric polynomial of degree at most D. Thus, $\tilde{g}$ must have at most $2D$ critical points in a single period. The same holds for $g_{w^\star}$ since $w^\star \neq 0$.

Recall that the period of $g_{w^\star}$ is $1/w^\star$, so there are at most $1/w^\star$ integer values to consider within one period of $g_{w^\star}$. Thus, there are at most $M_1/(2w^\star)$ intervals of size $2/M_1$ around these $1/w^\star$ values. Now, since there are at most $2D$ critical points, at most $2D$ of these intervals contain a critical point. Hence, the proportion of intervals (and hence inputs) for which $g_{w^\star}$ will not be monotone is at most

$$\frac{2D}{M_1/(2w^\star)} = \frac{4Dw^\star}{M_1} \leq \frac{4DR_w}{M_1}. \tag{133}$$

Outside of this proportion, we have already shown that $h_{w^\star,M_1,M_2}$ is pseudoperiodic. Thus, we can conclude that $h_{w^\star,M_1,M_2}$ is $(1 - 4DR_w/M_1)$-pseudoperiodic.

In the specific case where $\tilde{g}$ is given by Equation (10), $\tilde{g}$ clearly has bounded variation on every finite interval and is a trigonometric polynomial of degree at least D. Moreover, it is λ-Lipschitz with $\lambda = 2\pi D$:

$$\tilde{g}'(y) = -\sum_{j=1}^{D} \beta_j^\star \sin(2\pi j y) \cdot 2\pi j \tag{134}$$

$$|\tilde{g}'(y)| \le 2\pi \left| \sum_{j=1} j\beta_j^\star \sin(2\pi j y) \right| \le 2\pi D \sum_{j=1}^{D} |\beta_j^\star| = 2\pi D, \tag{135}$$

where we used that $j \le D$ and $\|\beta^\star\|_1 = 1$. Thus, we can apply the result we just proved for the case of $\lambda = 2\pi D$. Consider $M \ge 70\pi d D^3 R_w$, and take $M_1 = M$ and $M_2 = cM$ for $c < 1/(8\pi D R_w)$. Then,

$$\frac{4DR_w}{M_1} \le \frac{4DR_w}{70DR_w} = \frac{2}{35}. \tag{136}$$

Hence, $h_{w^\star,M_1,M_2}$ is (33/35)-pseudoperiodic. □

Thus, we see that this discretization still contains information about the period of the original function. We also truncate the domain of the function as well with truncation parameter R. Then, for $d = 1$, we require QSQ access to

$$|h_{w^\star}\rangle = \frac{1}{\sqrt{2R}} \sum_{x=-R}^{+R-1} |x\rangle\, |h_{w^\star,M_1,M_2}(x)\rangle\,. \tag{137}$$

With this truncation, $h_{w^\star,M_1,M_2}$ is still (33/35)-pseudoperiodic with period $M_1/w^\star$. Moreover, Lemma 6 also implies that for $d \ge 1$, $h_{w^\star,M_1,M_2}$ is (33/35)-pseudoperiodic in each coordinate with period $M_1/w_j^\star$. We discuss this in more detail later. However, note that because $1/w_j^\star$ is not necessarily an integer, the period $M_1/w_j^\star$ may also not be an integer. Hence, the standard period finding algorithm [10] does not apply. Instead, we want use an irrational period finding algorithm [7], which works even if $M_1/w_j^\star$ is irrational. We review Hallgren's algorithm in Section I B.

Lemma 6 guarantees that $h_{w^\star,M_1,M_2}$ is η-pseudoperiodic with $\eta = 33/35$. Moreover, note that Theorem 1 requires an upper bound on the period, which we have because $w_j \ge R_w/d^2$ by the definition of $\mathcal{S}_w$. The final condition of Theorem 1 that we need is this verification procedure to check if a given T is close to an integer multiple of the true period. We design such a verification procedure in Algorithm 2 and analyze it in Theorem 6. Note that in Algorithm 2, we must restrict the noise tolerance of our QSQs to be inverse polynomial in some of our parameters. Classically, the hardness results have access to gradients that are exponentially accurate, so requiring the tolerance parameter to scale inverse polynomially is not particularly strong.

Algorithm 2: Verification Procedure; Uniform Case

1: Choose parameters $M_1 = \max(70\pi d D^3 R_w, R_w^2/\epsilon_1)$, $M_2 = cM_1$ for some c such that $M_2 \in \mathbb{Z}$ and $c < 1/(8\pi D R_w)$, and $\tilde{R} = \tilde{\Omega}\left(\max\left(\frac{D^2}{\epsilon}, \frac{D^2\sqrt{d}}{R_w \epsilon}, \frac{D^{5/2}}{\sqrt{\epsilon}}, \frac{D^{3/2}\sqrt{d}}{R_w\sqrt{\epsilon}}\right)\right)$.

2: For $m \in \{1, \dots, D\}$, query the QSQ oracle with observable $O_{k,m}$ (defined in Equation (142)), discretization parameters $M_{1,m} \triangleq mM_1$, $M_{2,m} \triangleq mM_2$, truncation parameter $R \triangleq \tilde{R}M_{1,m}$, and tolerance $\tau \le \min\left(\frac{1}{M_2^2}\left(\frac{7}{40D} - \frac{1}{M_2}\right), \frac{1}{2D^2M_2^2}\left(\frac{2}{15} - \frac{1}{8}\left(\frac{2\pi R_w}{M_1}\right)^2 + \frac{2D^2}{M_2}\right)\right)$ to obtain values α_m.

3: Check if $\alpha_1 \ge \frac{1}{M_2^2}\left(\frac{21}{40D} - \frac{3}{M_2}\right)$.

4: Check if $\sum_{m=1}^{D} \alpha_m \le \frac{1}{M_2^2}\left(\frac{20}{39}D + \frac{1}{2D}\left(\frac{2}{15} - \frac{1}{8}\left(\frac{2\pi R_w}{M_1}\right)^2 + \frac{2D^2}{M_2}\right)\right)$.

5: **return** "yes" iff both conditions in Steps 3 and 4 are satisfied.

Theorem 6 (Verification Procedure; Uniform Case)**.** *Let φ^2 be the uniform distribution. Let $1 > \epsilon_1 > 0$. Let $w^\star \in \mathbb{R}^d$ be unknown with norm $R_w > 0$ and $w_j^\star \ge R_w/d^2$ for all $j \in [d]$. Let $g_{w^\star} : \mathbb{R}^d \to [-1,1]$ be defined as $g_{w^\star}(x) = \tilde{g}(x^\intercal w^\star)$ for $\tilde{g}$ given in Equation (10). Consider parameters $M_1 = \max(70\pi d D^3 R_w, R_w^2/\epsilon_1)$, $M_2 = cM_1$ for some constant c such that $c < 1/(8\pi D R_w)$ and $M_2 \in \mathbb{Z}$, and*

$$\tilde{R} = \tilde{\Omega}\left(\max\left(\frac{D^2}{\epsilon}, \frac{D^2\sqrt{d}}{R_w\epsilon}, \frac{D^{5/2}}{\sqrt{\epsilon}}, \frac{D^{3/2}\sqrt{d}}{R_w\sqrt{\epsilon}}\right)\right). \tag{138}$$

Suppose we have QSQ access (see Definition 1) with respect to discretization parameters $M_{1,m} \triangleq mM_1$, $M_{2,m} \triangleq mM_2$ and truncation parameter $R \triangleq \tilde{R}M_{1,m}$ for $m \in \{1, \dots, D\}$. Then, given an integer T and $k \in [d]$, Algorithm 2 can check whether or not $|T - \frac{\ell M_1}{w_k^\star}| \le 1$ for some integer ℓ using D QSQs with tolerance $\tau \le \min\left(\frac{1}{M_2^2}\left(\frac{7}{40D} - \frac{1}{M_2}\right), \frac{1}{2D^2M_2^2}\left(\frac{2}{15} - \frac{1}{8}\left(\frac{2\pi R_w}{M_1}\right)^2 + \frac{2D^2}{M_2}\right)\right)$.

Proof. Explicitly, the example state for our QSQ access is

$$\left|h_{w^\star,M_{1,m},M_{2,m}}\right\rangle = \frac{1}{\sqrt{(2\tilde{R}M_{1,m})^d}} \sum_{x_1,\ldots,x_d=-\tilde{R}M_{1,m}}^{\tilde{R}M_{1,m}-1} |x\rangle \left|h_{w^\star,M_{1,m},M_{2,m}}(x)\right\rangle, \tag{139}$$

where $h_{w^\star,M_{1,m},M_{2,m}}$ is a discretization of $g_{w^\star}$ from Lemma 6. We query D QSQs, each with the different parameters indexed by m as specified previously.

The main idea behind our verification procedure is to compute the inner product between $h_{w^\star,M_{1,m},M_{2,m}}$ and this function with its input shifted by the guess T for the period. This inner product should be large for a good guess. The technical work behind this theorem goes into defining an observable to approximate this inner product and finding a suitable threshold for the inner product to surpass such that T is close to the true period.

Consider defining the observable

$$A_m \triangleq I \otimes 2\,|-\rangle\!\langle-| \otimes \left(\frac{1}{M_{2,m}^2} \sum_{i,j=0}^{M_{2,m}-1} ij\,|i\rangle\!\langle j|\right), \tag{140}$$

where the identity is on the first $\log\left(\tilde{R}M_{1,m}\right) + d$ qubits (the extra d qubits are to represent the sign of each entry of $x_1,\ldots,x_d$). Also define an operator $S_{k,a}$ that cyclically shifts the kth entry of the input register by a. In particular, this acts as

$$S_{k,a} : |x\rangle \left|h_{w^\star,M_{1,m},M_{2,m}}(x)\right\rangle \mapsto |x + ae_k\rangle \left|h_{w^\star,M_{1,m},M_{2,m}}(x)\right\rangle, \tag{141}$$

where we use e_k to denote the unit vector with a one in the kth coordinate and zeros elsewhere. Then, we query the following observable as our QSQ to verify the period of the kth coordinate:

$$O_{k,m} \triangleq A_m S_{k,-T}. \tag{142}$$

First, we claim that this observable does indeed reflect our idea about computing the inner product between $h_{w^\star,M_{1,m},M_{2,m}}$ and this function with its input shifted by T.

Claim 1 (Approximating inner product)**.** *For $m \in \{1,\ldots,D\}$, consider parameters $M_{1,m}, M_{2,m}$ as defined above. Also consider a parameter $\tilde{R}$ and an observable $O_{k,m}$ as defined above. Then, the expectation value of O_m with respect to the example state in Equation (139) is given by*

$$\left\langle h_{w^\star,M_{1,m},M_{2,m}}\right|O_{k,m}\left|h_{w^\star,M_{1,m},M_{2,m}}\right\rangle \tag{143}$$

$$= \frac{1}{(2\tilde{R}M_{1,m})^d M_{2,m}^2} \sum_{x_1,\ldots,x_d=-\tilde{R}M_{1,m}}^{\tilde{R}M_{1,m}-1} h_{w^\star,M_{1,m},M_{2,m}}(x) h_{w^\star,M_{1,m},M_{2,m}}(x+Te_k), \tag{144}$$

where e_k denotes the unit vector with a single one in the kth coordinate.

Proof of Claim 1. This follows by a simple calculation.

$$\left\langle h_{w^\star,M_{1,m},M_{2,m}}\right|O_{k,m}\left|h_{w^\star,M_{1,m},M_{2,m}}\right\rangle \tag{145}$$

$$= \left\langle h_{w^\star,M_{1,m},M_{2,m}}\right|A_m S_{k,-T}\left|h_{w^\star,M_{1,m},M_{2,m}}\right\rangle \tag{146}$$

$$= \frac{1}{(2\tilde{R}M_{1,m})^d} \left(\sum_{x_1,\ldots,x_d=-\tilde{R}M_{1,m}}^{\tilde{R}M_{1,m}-1} \langle x| \left\langle h_{w^\star,M_{1,m},M_{2,m}}(x)\right|\right) A_m \left(\sum_{x'_1,\ldots,x'_d=-\tilde{R}M_{1,m}}^{\tilde{R}M_{1,m}-1} |x' - Te_k\rangle \left|h_{w^\star,M_{1,m},M_{2,m}}(x')\right\rangle\right) \tag{147}$$

$$= \frac{1}{(2\tilde{R}M_{1,m})^d} \sum_{\substack{x_1,\ldots,x_d=-\tilde{R}M_{1,m} \\ x'_1,\ldots,x'_d=-\tilde{R}M_{1,m}}}^{\tilde{R}M_{1,m}-1} \langle x| \left\langle h_{w^\star,M_{1,m},M_{2,m}}(x)\right| A_m |x'\rangle \left|h_{w^\star,M_{1,m},M_{2,m}}(x'+Te_k)\right\rangle \tag{148}$$

$$= \frac{1}{(2\tilde{R}M_{1,m})^d} \sum_{x_1,\ldots,x_d=-\tilde{R}M_{1,m}}^{\tilde{R}M_{1,m}-1} \left\langle h_{w^\star,M_{1,m},M_{2,m}}(x)\right| \left(2\,|-\rangle\!\langle-| \otimes \frac{1}{M_{2,m}^2} \sum_{i,j=0}^{M_{2,m}-1} ij\,|i\rangle\!\langle j|\right) \left|h_{w^\star,M_{1,m},M_{2,m}}(x+Te_k)\right\rangle \tag{149}$$

$$= \frac{1}{(2\tilde{R}M_{1,m})^d} \sum_{x_1,\ldots,x_d=-\tilde{R}M_{1,m}}^{\tilde{R}M_{1,m}-1} h_{w^\star,M_{1,m},M_{2,m}}(x) h_{w^\star,M_{1,m},M_{2,m}}(x+Te_k). \tag{150}$$

In the second line, we use the definition of $O_{k,m}$. In the third line, we use the definition of $S_{k,-T}$. In the fourth line, we relabel the x' indices in the summation $x' \mapsto x' - Te_k$. This still results in summing over the same values because $S_{k,-T}$ is defined to be a cyclical shift. In the fifth line, we use the definition of A_m and collapse the second summation by evaluating $\langle x|x'\rangle$. In the last line, we use the following calculation. For any two computational basis states $|a\rangle, |b\rangle$, where $a, b \in \{0, \ldots, M_{2,m}-1\}$, it is clear that

$$\langle a| \left(\frac{1}{M_{2,m}^2} \sum_{i,j=0}^{M_{2,m}-1} ij \, |i\rangle\!\langle j| \right) |b\rangle = \frac{1}{M_{2,m}^2} ab. \tag{151}$$

Similarly, if $|a\rangle, |b\rangle$ are instead representations of numbers in $[-1,1]$ using $\log(M_{2,m})+1$ bits, where the first qubit encodes the sign, then

$$\langle a| \left(2\,|-\rangle\!\langle -| \otimes \frac{1}{M_{2,m}^2} \sum_{i,j=0}^{M_{2,m}-1} ij \, |i\rangle\!\langle j| \right) |b\rangle = \frac{1}{M_{2,m}^2} ab. \tag{152}$$

If the sign qubits are the same for both $|a\rangle$ and $|b\rangle$, then the $2\,|-\rangle\!\langle -|$ term does not affect the overall sign. However, if the sign qubits are different, then the $2\,|-\rangle\!\langle -|$ term gives an extra minus sign, as required. Thus, we have proven the claim. □

Now, we want to show that the conditions checked in Steps 3 and 4 in Algorithm 2 are satisfied if and only if $|T - \ell M_1 / w_k^\star| \leq 1$. To do so, we first simplify our approximate inner product from Claim 1 further using the particular form of $h_{w^\star,M_{1,m},M_{2,m}}$ from Lemma 6 and $\tilde{g}$ from Equation (10).

$$\langle h_{w^\star,M_{1,m},M_{2,m}} | O_{k,m} | h_{w^\star,M_{1,m},M_{2,m}} \rangle \tag{153}$$

$$= \frac{1}{(2\tilde{R}M_{1,m})^d M_{2,m}^2} \sum_{x_1,\ldots,x_d=-\tilde{R}M_{1,m}}^{\tilde{R}M_{1,m}-1} h_{w^\star,M_{1,m},M_{2,m}}(x) h_{w^\star,M_{1,m},M_{2,m}}(x+Te_k) \tag{154}$$

$$= \frac{1}{(2\tilde{R}M_{1,m})^d M_{2,m}^2} \sum_{x_1,\ldots,x_d=-\tilde{R}M_{1,m}}^{\tilde{R}M_{1,m}-1} \sum_{j,j'=1}^{D} \beta_j^\star \beta_{j'}^\star \left\lfloor \cos\left(\frac{2\pi j x^\intercal w^\star}{M_{1,m}} \right) \right\rfloor_{M_{2,m}} \left\lfloor \cos\left(\frac{2\pi j' (x+Te_k)^\intercal w^\star}{M_{1,m}} \right) \right\rfloor_{M_{2,m}} \tag{155}$$

$$= \frac{1}{(2\tilde{R}M_{1,m})^d M_{2,m}^2} \sum_{x_1,\ldots,x_d=-\tilde{R}M_{1,m}}^{\tilde{R}M_{1,m}-1} \sum_{j,j'=1}^{D} \beta_j^\star \beta_{j'}^\star \cos\left(\frac{2\pi j x^\intercal w^\star}{M_{1,m}} \right) \cos\left(\frac{2\pi j' x^\intercal w^\star}{M_{1,m}} + \frac{2\pi j' T w_k^\star}{M_{1,m}} \right) + \epsilon_d \tag{156}$$

$$\begin{aligned} = \frac{1}{(2\tilde{R}M_{1,m})^d M_{2,m}^2} \sum_{x_1,\ldots,x_d=-\tilde{R}M_{1,m}}^{\tilde{R}M_{1,m}-1} \sum_{j,j'=1}^{D} \beta_j^\star \beta_{j'}^\star \cos\left(\frac{2\pi j x^\intercal w^\star}{M_{1,m}} \right) \Bigg(\cos\left(\frac{2\pi j' x^\intercal w^\star}{M_{1,m}} \right) \cos\left(\frac{2\pi j' T w_k^\star}{M_{1,m}} \right) \\ - \sin\left(\frac{2\pi j' x^\intercal w^\star}{M_{1,m}} \right) \sin\left(\frac{2\pi j' T w_k^\star}{M_{1,m}} \right) \Bigg) + \epsilon_d \end{aligned} \tag{157}$$

$$\begin{aligned} = \frac{1}{(2\tilde{R}M_{1,m})^d M_{2,m}^2} \sum_{x_1,\ldots,x_d=-\tilde{R}M_{1,m}}^{\tilde{R}M_{1,m}-1} \sum_{j=1}^{D} (\beta_j^\star)^2 \Bigg(\cos^2\left(\frac{2\pi j x^\intercal w^\star}{M_{1,m}} \right) \cos\left(\frac{2\pi j T w_k^\star}{M_{1,m}} \right) \\ - \cos\left(\frac{2\pi j x^\intercal w^\star}{M_{1,m}} \right) \sin\left(\frac{2\pi j x^\intercal w^\star}{M_{1,m}} \right) \sin\left(\frac{2\pi j T w_k^\star}{M_{1,m}} \right) \Bigg) \end{aligned} \tag{158}$$

$$+\frac{1}{(2\tilde{R}M_{1,m})^d M_{2,m}^2}\sum_{x_1,\ldots,x_d=-\tilde{R}M_{1,m}}^{\tilde{R}M_{1,m}-1}\sum_{\substack{j,j'=1\\ j\neq j'}}^{D}\beta_j^\star\beta_{j'}^\star\left(\cos\left(\frac{2\pi j x^\intercal w^\star}{M_{1,m}}\right)\cos\left(\frac{2\pi j' x^\intercal w^\star}{M_{1,m}}\right)\cos\left(\frac{2\pi j' T w_k^\star}{M_{1,m}}\right)\right.$$
$$\left.-\cos\left(\frac{2\pi j x^\intercal w^\star}{M_{1,m}}\right)\sin\left(\frac{2\pi j' x^\intercal w^\star}{M_{1,m}}\right)\sin\left(\frac{2\pi j' T w_k^\star}{M_{1,m}}\right)\right)+\epsilon_d \tag{159}$$

In the second line, we use Claim 1. In the third line, we use the definition of $h_{w^\star,M_{1,m},M_{2,m}}$ from Lemma 6 and Equation (10). Here, recall that $\lfloor\cdot\rceil_{M_{2,m}}$ denotes rounding to the nearest integer multiple of $M_{2,m}$. In the fourth line, we define a discretization error, denoted by ϵ_d, which accounts for the error in getting rid of the rounding. In the fifth line, we use the sum formula for cosine. In the last equality, we split up the sum into the cases when $j = j'$ and $j \neq j'$.

We want to upper and lower bound this expression. To do so, we find it easier to work with integrals over x instead of these discrete sums. We can then bound the integrals, which we relegate to Section IV C. To this end, we first need to bound the error from approximating our summation by an integral.

Claim 2 (Sum-to-integral error)**.** *For $m \in \{1,\ldots,D\}$, consider parameters $M_{1,m}, M_{2,m}$ as defined above. Also consider a parameter $\tilde{R}$ defined above. Then, for an integer $1 \leq j \leq D$,*

$$\frac{1}{(2\tilde{R})^d}\left|\int_{[-\tilde{R},\tilde{R}]^d}\cos^2\left(2\pi j x^\intercal w^\star\right)dx-\frac{1}{M_{1,m}^d}\sum_{x_1,\ldots,x_d=-\tilde{R}M_{1,m}}^{\tilde{R}M_{1,m}-1}\cos^2\left(\frac{2\pi j x^\intercal w^\star}{M_{1,m}}\right)\right|\leq\frac{1}{35D^2}. \tag{160}$$

Proof of Claim 2. We prove this by induction on the dimension d. In particular, denoting $f(x) \triangleq \cos^2(2\pi j x^\intercal w^\star)$, we will prove the following by induction:

$$\frac{1}{(2\tilde{R})^d}\left|\int_{[-\tilde{R},\tilde{R}]^d}f(x_1,\ldots,x_d)\,dx-\frac{1}{M_{1,m}^d}\sum_{x_1,\ldots,x_d=-\tilde{R}M_{1,m}}^{\tilde{R}M_{1,m}-1}f\left(\frac{x_1}{M_{1,m}},\ldots,\frac{x_d}{M_{1,m}}\right)\right|\leq\frac{2\pi dDR_w}{M_{1,m}}. \tag{161}$$

Note that this implies our claim by our choice of $M_{1,m} = mM_1 \geq 70m\pi dD^3R_w \geq 70\pi dD^3R_w$. Thus, it suffices to prove Equation (161). In fact, we will use induction to prove that

$$\frac{1}{(2\tilde{R})^{d-1}}\left|\int_{[-\tilde{R},\tilde{R}]^{d-1}}f(x_1,\ldots,x_{d-1},y)\,dx-\frac{1}{M_{1,m}^{d-1}}\sum_{x_1,\ldots,x_{d-1}=-\tilde{R}M_{1,m}}^{\tilde{R}M_{1,m}-1}f\left(\frac{x_1}{M_{1,m}},\ldots,\frac{x_{d-1}}{M_{1,m}},y\right)\right| \tag{162}$$
$$\leq\frac{2\pi(d-1)DR_w}{M_{1,m}} \tag{163}$$

for some fixed y. In the process, we show that Equation (161) follows from this.

First, consider the base case. Then, we want to prove

$$\frac{1}{2\tilde{R}}\left|\int_{-\tilde{R}}^{+\tilde{R}}f(x)\,dx-\frac{1}{M_{1,m}}\sum_{x=-\tilde{R}M_{1,m}}^{\tilde{R}M_{1,m}-1}f\left(\frac{x}{M_{1,m}}\right)\right|\leq\frac{2\pi DR_w}{M_{1,m}} \tag{164}$$

and

$$\frac{1}{2\tilde{R}}\left|\int_{-\tilde{R}}^{+\tilde{R}}f(x,y)\,dx-\frac{1}{M_{1,m}}\sum_{x=-\tilde{R}M_{1,m}}^{\tilde{R}M_{1,m}-1}f\left(\frac{x}{M_{1,m}},y\right)\right|\leq\frac{2\pi DR_w}{M_{1,m}}, \tag{165}$$

for some fixed y and $f(x,y) \triangleq \cos^2(2\pi j(xw_1^\star + yw_2^\star))$. First, consider Equation (164). Notice that the sum in Equation (164) is just the lefthand Riemann sum for the integral. In particular, we approximate the integral by $2\tilde{R}M_{1,m}$ rectangles of width $2\tilde{R}/(2\tilde{R}M_{1,m}) = 1/M_{1,m}$. Thus, we have

$$\int_{-\tilde{R}}^{+\tilde{R}}f(x)\,dx\approx\frac{1}{M_{1,m}}\sum_{i=0}^{2\tilde{R}M_{1,m}-1}f\left(-\tilde{R}+\frac{i}{M_{1,m}}\right)=\frac{1}{M_{1,m}}\sum_{x=-\tilde{R}M_{1,m}}^{\tilde{R}M_{1,m}-1}f\left(\frac{x}{M_{1,m}}\right). \tag{166}$$

Moreover, the error in this approximation can be bounded by standard results:

$$\left| \int_{-\tilde{R}}^{+\tilde{R}} f(x)\, dx - \frac{1}{M_{1,m}} \sum_{x=-\tilde{R}M_{1,m}}^{\tilde{R}M_{1,m}-1} f\left(\frac{x}{M_{1,m}}\right) \right| \le \frac{L\tilde{R}}{M_{1,m}}, \tag{167}$$

where $L \triangleq \max_{x \in [-\tilde{R},\tilde{R}]} |f'(x)|$. For our choice of $f(x) = \cos^2(2\pi j x w^\star)$, then

$$f'(x) = -4\pi j w^\star \cos(2\pi j x w^\star) \sin(2\pi j x w^\star) \tag{168}$$

so that $|f'(x)| \le 4\pi j R_w \le 4\pi D R_w$. Thus, $L \le 4\pi D R_w$. Dividing both sides by $2\tilde{R}$, we obtain the claim. Note that Equation (165) also follows by the same argument as above for $\tilde{f}(x) \triangleq f(x,y)$ for a fixed y, where $f(x,y) = \cos^2(2\pi j(x w_1^\star + y w_2^\star))$. Namely, the only part of the above argument that relies on properties of the function f was a bound on the derivative. For $\tilde{f}$, we have the same bound:

$$\tilde{f}'(x) = -4\pi j w_1^\star \cos(2\pi j(x w_1^\star + y w_2^\star)) \sin(2\pi j(x w_1^\star + y w_2^\star)) \tag{169}$$

so that $|\tilde{f}'(x)| \le 4\pi D R_w$.

Now, for the inductive step, suppose for ℓ such that $d-1 \ge \ell \ge 1$ that

$$\frac{1}{(2\tilde{R})^\ell} \left| \int_{[-\tilde{R},\tilde{R}]^\ell} f(x_1,\ldots,x_\ell,y)\, dx - \frac{1}{M_{1,m}^\ell} \sum_{x_1,\ldots,x_\ell=-\tilde{R}M_{1,m}}^{\tilde{R}M_{1,m}-1} f\left(\frac{x_1}{M_{1,m}},\ldots,\frac{x_\ell}{M_{1,m}},y\right) \right| \le \frac{4\pi\ell D\tilde{R}_w}{M_{1,m}}, \tag{170}$$

for some fixed y and where $f(x_1,\ldots,x_\ell,y) = \cos^2(2\pi j(x_1 w_1^\star + \cdots + x_\ell w_\ell^\star + y w_{\ell+1}^\star))$. We first show that Equation (161) holds for $\ell+1$.

$$\frac{1}{(2\tilde{R})^{\ell+1}} \int_{[-\tilde{R},\tilde{R}]^{\ell+1}} f(x)\, dx \tag{171}$$

$$= \frac{1}{2\tilde{R}} \int_{-\tilde{R}}^{+\tilde{R}} \left(\frac{1}{(2\tilde{R})^\ell} \int_{[-\tilde{R},\tilde{R}]^\ell} f(x_1,\ldots,x_{\ell+1})\, dx_1 \cdots dx_\ell \right) dx_{\ell+1} \tag{172}$$

$$\le \frac{1}{(2\tilde{R})^{\ell+1} M_{1,m}^\ell} \sum_{x_1,\ldots,x_\ell=-\tilde{R}M_{1,m}}^{\tilde{R}M_{1,m}-1} \int_{-\tilde{R}}^{+\tilde{R}} f\left(\frac{x_1}{M_{1,m}},\ldots,\frac{x_\ell}{M_{1,m}},x_{\ell+1}\right) dx_{\ell+1} + \frac{1}{2\tilde{R}} \int_{-\tilde{R}}^{+\tilde{R}} \frac{2\pi\ell D R_w}{M_{1,m}}\, dx_{\ell+1} \tag{173}$$

$$\le \frac{1}{(2\tilde{R})^{\ell+1} M_{1,m}^\ell} \sum_{x_1,\ldots,x_\ell=-\tilde{R}M_{1,m}}^{\tilde{R}M_{1,m}-1} \left(\frac{1}{M_{1,m}} \sum_{x_{\ell+1}=-\tilde{R}M_{1,m}}^{\tilde{R}M_{1,m}} f\left(\frac{x_1}{M_{1,m}},\ldots,\frac{x_{\ell+1}}{M_{1,m}}\right) + \frac{L'\tilde{R}}{M_{1,m}} \right) + \frac{2\pi\ell D R_w}{M_{1,m}} \tag{174}$$

$$= \frac{1}{(2\tilde{R})^{\ell+1} M_{1,m}^{\ell+1}} \sum_{x_1,\ldots,x_{\ell+1}=-\tilde{R}M_{1,m}}^{\tilde{R}M_{1,m}-1} f\left(\frac{x_1}{M_{1,m}},\ldots,\frac{x_{\ell+1}}{M_{1,m}}\right) + \frac{L'}{2M_{1,m}} + \frac{2\pi\ell D R_w}{M_{1,m}}, \tag{175}$$

where in the third line, we use the inductive hypothesis. In the fourth line, we apply Equation (167) for the function $\tilde{f}(y) \triangleq f(x_1/M_{1,m},\ldots,x_\ell/M_{1,m},y)$. Also, here, $L' \triangleq \max_{y\in[-\tilde{R},\tilde{R}]} |\tilde{f}'(y)|$. For $f(x_1,\ldots,x_{\ell+1}) = \cos^2(2\pi j(x_1 w_1^\star + \cdots x_{\ell+1} w_{\ell+1}^\star))$, then

$$\tilde{f}'(y) = -4\pi j w_{\ell+1}^\star \cos\left(2\pi j \left(y w_{\ell+1}^\star + \sum_{i=1}^\ell \frac{x_i}{M_{1,m}} w_i^\star\right)\right) \sin\left(2\pi j \left(y w_{\ell+1}^\star + \sum_{i=1}^\ell \frac{x_i}{M_{1,m}} w_i^\star\right)\right). \tag{176}$$

Thus, $|\tilde{f}'(y)| \le 4\pi j R_w \le 4\pi D R_w$ so that $L' \le 4\pi D R_w$. Plugging this back into the above, we have

$$\frac{1}{(2\tilde{R})^{\ell+1}} \int_{[-\tilde{R},\tilde{R}]^{\ell+1}} f(x)\, dx \le \frac{1}{(2\tilde{R})^{\ell+1} M_{1,m}^{\ell+1}} \sum_{x_1,\ldots,x_{\ell+1}=-\tilde{R}M_{1,m}}^{\tilde{R}M_{1,m}-1} f\left(\frac{x_1}{M_{1,m}},\ldots,\frac{x_{\ell+1}}{M_{1,m}}\right) + \frac{4\pi(k+1) D R_w}{M_{1,m}}. \tag{177}$$

One can argue similarly for the lower bound. Thus, we have shown that Equation (161) holds for $\ell+1$.

Now, to complete our induction, we need to show that Equation (162) holds for $\ell+1$. Namely, we want to show

$$\frac{1}{(2\tilde{R})^{\ell+1}}\left|\int_{[-\tilde{R},\tilde{R}]^{\ell+1}} f(x_1,\dots,x_{\ell+1},z)\,dx - \frac{1}{M_{1,m}^{\ell+1}}\sum_{x_1,\dots,x_{\ell+1}=-\tilde{R}M_{1,m}}^{\tilde{R}M_{1,m}-1} f\left(\frac{x_1}{M_{1,m}},\dots,\frac{x_{\ell+1}}{M_{1,m}},z\right)\right| \le \frac{4\pi(\ell+1)DR_w}{M_{1,m}} \tag{178}$$

for some fixed z and where $f(x_1,\dots,x_{\ell+1},z)=\cos^2(2\pi j(x_1w_1^\star+\cdots+x_{\ell+1}w_{\ell+1}^\star+zw_{\ell+2}^\star))$. This follows by the same argument as above. Note that the inductive hypothesis can still be applied by taking $y=x_{\ell+1}+z(w_{\ell+2}^\star/w_{\ell+1}^\star)$, which is fixed when integrating with respect to $x_1,\dots,x_\ell$. Moreover, when applying Equation (167), we instead consider the function $\tilde{f}(x_{\ell+1})\triangleq f(x_1/M_{1,m},\dots,x_\ell/M_{1,m},x_{\ell+1},z)$. The bound on the derivative of this function is clearly the same since z is fixed. Thus, the same argument as above applies, completing the induction. □

Note that the same result can be shown for the cross terms $\cos(2\pi jx^\intercal w^\star/M_{1,m})\cos(2\pi j'x^\intercal w^\star)$ and $\cos(2\pi jx^\intercal w^\star/M_{1,m})\sin(2\pi j'x^\intercal w^\star/M_{1,m})$ by the same argument. This is clear because these terms have the same bound on their gradients.

We can also bound the discretization error ϵ_d. Note that this discretization error is defined as

$$\epsilon_d \triangleq \frac{1}{(2\tilde{R}M_{1,m})^d M_{2,m}^2}\sum_{x_1,\dots,x_d=-\tilde{R}M_{1,m}}^{\tilde{R}M_{1,m}-1}\sum_{j,j'=1}^{D}\beta_j^\star\beta_{j'}^\star\left(\cos\left(\frac{2\pi jx^\intercal w^\star}{M_{1,m}}\right)\cos\left(\frac{2\pi j'(x+Te_k)^\intercal w^\star}{M_{1,m}}\right)\right. \tag{179}$$

$$\left. - \left\lfloor\cos\left(\frac{2\pi jx^\intercal w^\star}{M_{1,m}}\right)\right\rfloor_{M_{2,m}}\left\lfloor\cos\left(\frac{2\pi j'(x+Te_k)^\intercal w^\star}{M_{1,m}}\right)\right\rfloor_{M_{2,m}}\right). \tag{180}$$

Claim 3 (Discretization error)**.** *For $m\in\{1,\dots,D\}$, consider parameters $M_{1,m},M_{2,m}$ as defined above. Also, consider a parameter $\tilde{R}$ defined above. Then, we can bound the discretization error ϵ_d defined in Equation (179) as*

$$|\epsilon_d| \le \frac{2}{M_{2,m}^3}. \tag{181}$$

Proof of Claim 3. This follows by a simple calculation. First, we can add and subtract an intermediate term in which $\cos(2\pi jx^\intercal w^\star/M_{1,m})$ is rounded while $\cos(2\pi j'(x+Te_k)^\intercal w^\star/M_{1,m})$.

$$|\epsilon_d| \le \frac{1}{(2\tilde{R}M_{1,m})^d M_{2,m}^2}\sum_{x_1,\dots,x_d=-\tilde{R}M_{1,m}}^{\tilde{R}M_{1,m}-1}\sum_{j,j'=1}^{D}|\beta_j^\star||\beta_{j'}^\star| \tag{182}$$

$$\cdot\left(\left|\cos\left(\frac{2\pi jx^\intercal w^\star}{M_{1,m}}\right)\cos\left(\frac{2\pi j'(x+Te_k)^\intercal w^\star}{M_{1,m}}\right) - \left\lfloor\cos\left(\frac{2\pi jx^\intercal w^\star}{M_{1,m}}\right)\right\rfloor_{M_{2,m}}\cos\left(\frac{2\pi j'(x+Te_k)^\intercal w^\star}{M_{1,m}}\right)\right|\right. \tag{183}$$

$$+\left|\left\lfloor\cos\left(\frac{2\pi jx^\intercal w^\star}{M_{1,m}}\right)\right\rfloor_{M_{2,m}}\cos\left(\frac{2\pi j'(x+Te_k)^\intercal w^\star}{M_{1,m}}\right)\right. \tag{184}$$

$$\left.\left. - \left\lfloor\cos\left(\frac{2\pi jx^\intercal w^\star}{M_{1,m}}\right)\right\rfloor_{M_{2,m}}\left\lfloor\cos\left(\frac{2\pi j'(x+Te_k)^\intercal w^\star}{M_{1,m}}\right)\right\rfloor_{M_{2,m}}\right|\right). \tag{185}$$

Simplifying, we have

$$|\epsilon_d| \tag{186}$$

$$\le \frac{1}{(2\tilde{R}M_{1,m})^d M_{2,m}^2}\sum_{x_1,\dots,x_d=-\tilde{R}M_{1,m}}^{\tilde{R}M_{1,m}-1}\sum_{j,j'=1}^{D}|\beta_j^\star||\beta_{j'}^\star|\left(\left|\cos\left(\frac{2\pi jx^\intercal w^\star}{M_{1,m}}\right) - \left\lfloor\cos\left(\frac{2\pi jx^\intercal w^\star}{M_{1,m}}\right)\right\rfloor_{M_{2,m}}\right|\right.$$

$$\left. + \left|\cos\left(\frac{2\pi j'(x+Te_k)^\intercal w^\star}{M_{1,m}}\right) - \left\lfloor\cos\left(\frac{2\pi j'(x+Te_k)^\intercal w^\star}{M_{1,m}}\right)\right\rfloor_{M_{2,m}}\right|\right) \tag{187}$$

$$\leq \frac{1}{(2\tilde{R}M_{1,m})^d M_{2,m}^2} \sum_{x_1,\ldots,x_d=-\tilde{R}M_{1,m}}^{\tilde{R}M_{1,m}-1} \sum_{j,j'=1}^{D} |\beta_j^\star||\beta_{j'}^\star| \frac{2}{M_{2,m}} \tag{188}$$

$$= \frac{2}{M_{2,m}^3}. \tag{189}$$

In the first inequality, we use that $|\cos(x)| \leq 1$. In the second inequality, we use that $\lfloor\cdot\rceil_{M_{2,m}}$ means rounding to the nearest integer multiple of $M_{2,m}$. Thus, the difference between a rounded and unrounded quantity must be at most $1/M_{2,m}$. Finally, in the last line, we use that $\|\beta^\star\|_1 = 1$. □

With Claim 2 and Claim 3, in Equations (158) and (159), we now have

$$\langle h_{w^\star,M_{1,m},M_{2,m}}|O_{k,m}|h_{w^\star,M_{1,m},M_{2,m}}\rangle \tag{190}$$

$$= \frac{1}{M_{2,m}^2}\int_{x\sim\varphi^2}\sum_{j=1}^{D}(\beta_j^\star)^2\left(\cos^2(2\pi j x^\intercal w^\star)\cos\left(\frac{2\pi jTw_k^\star}{M_1}\right) - \cos(2\pi j x^\intercal w^\star)\sin(2\pi j x^\intercal w^\star)\sin\left(\frac{2\pi jTw_k^\star}{M_{1,m}}\right)\right)dx \tag{191}$$

$$+\frac{1}{M_{2,m}^2}\int_{x\sim\varphi^2}\sum_{\substack{j,j'=1\\ j\neq j'}}^{D}\beta_j^\star\beta_{j'}^\star\left(\cos(2\pi j x^\intercal w^\star)\cos(2\pi j' x^\intercal w^\star)\cos\left(\frac{2\pi j'Tw_k^\star}{M_{1,m}}\right)\right.$$
$$\left. - \cos(2\pi j x^\intercal w^\star)\sin(2\pi j' x^\intercal w^\star)\sin\left(\frac{2\pi j'Tw_k^\star}{M_{1,m}}\right)\right)dx + \epsilon_d + \frac{4}{M_{2,m}^2}\epsilon_{\text{int}}, \tag{192}$$

where $|\epsilon_d| \leq 2/M_{2,m}^3$ and $|\epsilon_{\text{int}}| \leq 1/(35D^2)$. Here, the integrals are with respect to the uniform distribution over $[-\tilde{R},\tilde{R}]^d$. We can simplify this using the fact that an integral of an odd function, e.g., $\sin(x)\cos(x)$, over an even interval is zero:

$$\begin{aligned}
&\langle h_{w^\star,M_{1,m},M_{2,m}}|O_{k,m}|h_{w^\star,M_{1,m},M_{2,m}}\rangle\\
&= \frac{1}{M_{2,m}^2}\sum_{j=1}^{D}(\beta_j^\star)^2\cos\left(\frac{2\pi jTw_k^\star}{M_{1,m}}\right)\int_{x\sim\varphi^2}\cos^2(2\pi j x^\intercal w^\star)\,dx\\
&+\frac{1}{M_{2,m}^2}\sum_{\substack{j,j'=1\\ j\neq j'}}^{D}\beta_j^\star\beta_{j'}^\star\left(\cos\left(\frac{2\pi j'Tw_k^\star}{M_{1,m}}\right)\int_{x\sim\varphi^2}\cos(2\pi j x^\intercal w^\star)\cos(2\pi j' x^\intercal w^\star)\,dx\right.\\
&\left.-\sin\left(\frac{2\pi j'Tw_k^\star}{M_{1,m}}\right)\int_{x\sim\varphi^2}\cos(2\pi j x^\intercal w^\star)\sin(2\pi j' x^\intercal w^\star)\,dx\right) + \epsilon_d + \frac{4}{M_{2,m}^2}\epsilon_{\text{int}}.
\end{aligned} \tag{193}$$

With this, we can finally move on to show that the conditions checked in Steps 3 and 4 of Algorithm 2 are satisfied if and only if $|T - \ell M_1/w_k^\star| \leq 1$. To do so, we leverage integral bounds from Section IV C. The following two claims show this for each direction of the if and only if.

Claim 4 (Correctness of Step 3 in Algorithm 2). *Consider parameters $M_1, M_2, \tilde{R}$ defined above and the observable $O_{k,1}$ defined in Equation (142). Let α_1 denote the result of querying the QSQ oracle with observable $O_{k,1}$ with discretization parameters M_1, M_2, truncation parameter $R \triangleq \tilde{R}M_1$, and tolerance $\tau \leq \frac{1}{M_2^2}\left(\frac{7}{40D} - \frac{1}{M_2}\right)$. If $|T - \ell M_1/w_k^\star| \leq 1$ for some integer ℓ, then*

$$\alpha_1 \geq \frac{1}{M_2^2}\left(\frac{21}{40D} - \frac{3}{M_2}\right). \tag{194}$$

Claim 5 (Correctness of Step 4 in Algorithm 2). *For $m \in \{1,\ldots,D\}$, consider parameters $M_{1,m}, M_{2,m}, \tilde{R}$ defined above and the observables $O_{k,m}$ defined in Equation (142). Let α_m denote the result of querying the QSQ oracle with observable $O_{k,m}$ with discretization parameters $M_{1,m}, M_{2,m}$, truncation parameter $R \triangleq \tilde{R}M_{1,m}$, and tolerance $\tau \leq \frac{1}{2D^2M_2^2}\left(\frac{2}{15} - \frac{1}{8}\left(\frac{2\pi R_w}{M_1}\right)^2 + \frac{2D^2}{M_2}\right)$. If $|T - \ell M_1/w_k^\star|$ is not less than 1 for any integer ℓ, then*

$$\sum_{m=1}^{D}\alpha_m \leq \frac{1}{M_2^2}\left(\frac{20}{39}D + \frac{1}{2D}\left(\frac{2}{15} - \frac{1}{8}\left(\frac{2\pi R_w}{M_1}\right)^2 + \frac{2D^2}{M_2}\right)\right). \tag{195}$$

It suffices to prove these two claims to finish the proof. Our starting point for both proofs is Equation (193).

Proof of Claim 4. We can lower bound $\langle h_{w^\star,M_1,M_2}|O_{k,1}|h_{w^\star,M_1,M_2}\rangle$ using Corollaries 4, 6 and 7 and Equation (193):

$$\langle h_{w^\star,M_1,M_2}|O_{k,1}|h_{w^\star,M_1,M_2}\rangle \geq \frac{1}{M_2^2}\sum_{j=1}^{D}(\beta_j^\star)^2\left(\frac{1}{2}-\frac{\sqrt{d}}{8\pi R_w\tilde{R}}\right)\cos\left(\frac{2\pi jTw_k^\star}{M_1}\right) \tag{196}$$

$$-\frac{1}{M_2^2}\sum_{\substack{j,j'=1\\ j\neq j'}}^{D}\beta_j^\star\beta_{j'}^\star\left(\frac{\sqrt{d}}{\pi R_w\tilde{R}}\right)+\epsilon_d+\frac{4}{M_2^2}\epsilon_{\text{int}} \tag{197}$$

$$\geq \frac{1}{M_2^2}\left(\sum_{j=1}^{D}(\beta_j^\star)^2\left(\frac{1}{2}-\frac{\sqrt{d}}{8\pi R_w\tilde{R}}\right)\cos\left(\frac{2\pi jTw_k^\star}{M_1}\right)-\frac{\sqrt{d}}{\pi R_w\tilde{R}}-\frac{2}{M_2}-\frac{4}{35D^2}\right) \tag{198}$$

$$\geq \frac{1}{M_2^2}\left(\frac{19}{39}\sum_{j=1}^{D}(\beta_j^\star)^2\cos\left(\frac{2\pi jTw_k^\star}{M_1}\right)-\frac{1}{54D^2}-\frac{2}{M_2}-\frac{4}{35D^2}\right). \tag{199}$$

In the second to last line, we use that $\|\beta^\star\|_2^2 \leq 1$ since $\|\beta^\star\|_1 = 1$. We also used that $|\epsilon_d| \leq 2/M_2^3$ by Claim 3 and $|\epsilon_{\text{int}}| \leq 1/(35D^2)$ by Claim 2. In the last line, we use that $\tilde{R} \geq \max(39\sqrt{d}/(4\pi R_w),\ 54D^2\sqrt{d}/(\pi R_w))$ in our choice of $\tilde{R}$.

Here, the key is that the summation over these cosine terms is peaked aroud multiples of $M_1/w_k^\star$. Thus, this sum should be bounded away from 0 when the guess T is close to an integer multiple of the period $M_1/w_k^\star$. The rest of the terms in this expression are error terms. Suppose that $T = \ell M_1/w_k^\star + \epsilon$ for some $|\epsilon| \leq 1$. Then, we have

$$\sum_{j=1}^{D}(\beta_j^\star)^2\cos\left(\frac{2\pi jTw_k^\star}{M_1}\right) = \sum_{j=1}^{D}(\beta_j^\star)^2\cos\left(\frac{2\pi jw_k^\star}{M_1}\left(\frac{\ell M_1}{w_k^\star}+\epsilon\right)\right) \tag{200}$$

$$= \sum_{j=1}^{D}(\beta_j^\star)^2\cos\left(\frac{2\pi jw_k^\star}{M_1}\epsilon\right) \tag{201}$$

$$\geq \sum_{j=1}^{D}(\beta_j^\star)^2\left(1-\frac{1}{2}\left(2\pi j\frac{w_k^\star}{M_1}\epsilon\right)^2\right) \tag{202}$$

$$\geq \sum_{j=1}^{D}(\beta_j^\star)^2\left(1-\frac{1}{2}\left(\frac{2\pi jw_k^\star}{M_1}\right)^2\right) \tag{203}$$

$$\geq \frac{1}{D}-\frac{1}{2}\sum_{j=1}^{D}(\beta_j^\star)^2\left(\frac{2\pi DR_w}{M_1}\right)^2 \tag{204}$$

$$\geq \frac{1}{D}-\frac{1}{2}\left(\frac{2\pi DR_w}{M_1}\right)^2 \tag{205}$$

$$\geq \frac{1}{D}-\frac{1}{2\cdot 35^2D^4} \tag{206}$$

$$\geq \frac{2449}{2450D}. \tag{207}$$

In the second line, we use the periodicity of cosine. In the third line, we use that $\cos(x) \geq 1 - x/2$. In the fourth line, we use that $|\epsilon| \leq 1$. In the fifth line, we use that $\|\beta^\star\|_2^2 \geq 1/D$ since $\|\beta^\star\|_1 = 1$. In the sixth line, we use that $\|\beta^\star\|_2^2 \leq 1$. In the seventh line, we use that $M_1 \geq 70\pi D^3 R_w$. Finally, in the last line, we use that $D \geq 1$ so that $D^4 \geq D$.

Plugging this into Equation (199), we have

$$\langle h_{w^\star,M_1,M_2}|O_{k,1}|h_{w^\star,M_1,M_2}\rangle \geq \frac{1}{M_2^2}\left(\frac{19}{39}\cdot\frac{2449}{2450D}-\frac{1}{54D^2}-\frac{2}{M_2}-\frac{4}{35D^2}\right) \tag{208}$$

$$\geq \frac{1}{M_2^2}\left(\frac{7}{20D} - \frac{2}{M_2}\right). \tag{209}$$

In the second line, we use that $D^2 \geq D$ and simplify.

Thus, we see that if $|T - \ell M_1/w_k^\star| \leq 1$, then this lower bound on the expectation value must be satisfied. Recall that QSQs only approximate the expectation value up to some tolerance τ. By our choice of τ, we have

$$|\alpha_1 - \langle h_{w^\star,M_1,M_2}|O_{k,1}|h_{w^\star,M_1,M_2}\rangle| \leq \frac{1}{M_2^2}\left(\frac{7}{40D} - \frac{1}{M_2}\right). \tag{210}$$

By choosing the condition

$$\alpha_1 \geq \frac{1}{M_2^2}\left(\frac{21}{40D} - \frac{3}{M_2}\right), \tag{211}$$

we can ensure that

$$\langle h_{w^\star,M_1,M_2}|O_{k,1}|h_{w^\star,M_1,M_2}\rangle \geq \alpha_1 - \frac{1}{M_2^2}\left(\frac{7}{40D} - \frac{1}{M_2}\right) \geq \frac{1}{M_2^2}\left(\frac{7}{20D} - \frac{2}{M_2}\right), \tag{212}$$

as required. □

Proof of Claim 5. This time, we can upper bound $\langle h_{w^\star,M_{1,m},M_{2,m}}|O_{k,m}|h_{w^\star,M_{1,m},M_{2,m}}\rangle$ for any $m \in \{1,\dots,D\}$ using Corollaries 5 to 7 and Equation (193):

$$\langle h_{w^\star,M_{1,m},M_{2,m}}|O_{k,m}|h_{w^\star,M_{1,m},M_{2,m}}\rangle \tag{213}$$

$$\leq \frac{1}{M_{2,m}^2}\sum_{j=1}^{D}(\beta_j^\star)^2\left(\frac{1}{2} + \frac{\sqrt{d}}{8\pi R_w \tilde{R}}\right)\cos\left(\frac{2\pi j T w_k^\star}{M_{1,m}}\right) + \frac{1}{M_{2,m}^2}\sum_{\substack{j,j'=1\\ j\neq j'}}^{D}\beta_j^\star\beta_{j'}^\star\left(\frac{\sqrt{d}}{\pi R_w\tilde{R}}\right) + \epsilon_d + \frac{4}{M_{2,m}^2}\epsilon_{\mathrm{int}} \tag{214}$$

$$\leq \frac{1}{M_{2,m}^2}\left(\left(\frac{1}{2} + \frac{\sqrt{d}}{8\pi R_w\tilde{R}}\right)\left((\beta_m^\star)^2\cos\left(\frac{2\pi T w_k^\star}{M_1}\right) + \sum_{\substack{j=1\\ j\neq m}}^{D}(\beta_j^\star)^2\right) + \frac{\sqrt{d}}{\pi R_w\tilde{R}} + \frac{2}{M_{2,m}} + \frac{4}{35D^2}\right) \tag{215}$$

$$\leq \frac{1}{M_{2,m}^2}\left(\frac{20}{39}(\beta_m^\star)^2\cos\left(\frac{2\pi T w_k^\star}{M_1}\right) + \frac{20}{39}\sum_{\substack{j=1\\ j\neq m}}^{D}(\beta_j^\star)^2 + \frac{1}{54D^2} + \frac{2}{M_{2,m}} + \frac{4}{35D^2}\right). \tag{216}$$

In the third line, we split up the sum over j into cases where $j = m$ and $j \neq m$. In the $j = m$ case, we use that $M_{1,m} = mM_1$ by definition. In the $j \neq m$ case, we bound $\cos(x) \leq 1$. We also use that $|\epsilon_d| \leq 2/M_{2,m}^3$ by Claim 3 and $|\epsilon_{\mathrm{int}}| \leq 1/(35D^2)$ by Claim 2 and $\|\beta^\star\|_1 = 1$. In the last line, we use that $\tilde{R} \geq \max(39\sqrt{d}/4\pi R_w, 54D^2\sqrt{d}/\pi R_w)$ by our choice of $\tilde{R}$.

Now, suppose that there does not exist any integer ℓ such that $|T - \ell M_1/w_k^\star| \leq 1$. Then, we can write $T = \ell' M_1/w_k^\star + c$ for some c satisfying $1 < c < M_1/w_k^\star - 1$. Then,

$$\cos\left(\frac{2\pi T w_k^\star}{M_1}\right) = \cos\left(\frac{2\pi w_k^\star}{M_1}\left(\frac{\ell' M_1}{w_k^\star} + c\right)\right) = \cos\left(\frac{2\pi w_k^\star}{M_1}c\right). \tag{217}$$

Without loss of generality, we can assume that $w_k^\star c/M_1 \leq 1/2$. Otherwise, we can write

$$\cos\left(\frac{2\pi w_k^\star}{M_1}c\right) = \cos\left(\frac{2\pi w_k^\star}{M_1}\left(\frac{M_1}{w_k^\star} - c'\right)\right) = \cos\left(\frac{2\pi w_k^\star}{M_1}c'\right) \tag{218}$$

for some c' such that $w_k^\star c'/M_1 \leq 1/2$. Then, we can bound this cosine term:

$$\cos\left(\frac{2\pi T w_k^\star}{M_1}\right) = \cos\left(\frac{2\pi w_k^\star}{M_1}c\right) \tag{219}$$

$$\leq 1 - \frac{1}{8}\left(\frac{2\pi w_k^\star}{M_1}c\right)^2 \tag{220}$$

$$\leq 1 - \frac{1}{8}\left(\frac{2\pi R_w}{M_1}\right)^2. \tag{221}$$

Here, in the second line, we use that $\cos(x) \leq 1 - x/8$ for $x \in [0, \pi]$, which is satisfied because we can assume that $w_k^\star c/M_1 \leq 1/2$ as discussed above. In the last line, we use that $c > 1$ and $w_k^\star \leq R_w$.

Plugging this into Equation (216), we have

$$\langle h_{w^\star,M_{1,m},M_{2,m}} | O_{k,m} | h_{w^\star,M_{1,m},M_{2,m}} \rangle \tag{222}$$

$$\leq \frac{1}{M_{2,m}^2}\left(\frac{20}{39}(\beta_m^\star)^2\left(1 - \frac{1}{8}\left(\frac{2\pi R_w}{M_1}\right)^2\right) + \frac{20}{39}\sum_{\substack{j=1\\ j\neq m}}^{D}(\beta_j^\star)^2 + \frac{1}{54D^2} + \frac{2}{M_{2,m}} + \frac{4}{35D^2}\right) \tag{223}$$

$$\leq \frac{1}{M_{2,m}^2}\left(\frac{20}{39}\left(1 - \frac{1}{8}\left(\frac{2\pi R_w}{M_1}\right)^2(\beta_m^\star)^2\right) + \frac{1}{54D^2} + \frac{2}{M_{2,m}} + \frac{4}{35D^2}\right). \tag{224}$$

In the last line, we use that $\|\beta^\star\|_2^2 \leq 1$. Summing over all $m \in \{1, \ldots, D\}$, then we have

$$\sum_{m=1}^{D} \langle h_{w^\star,M_{1,m},M_{2,m}} | O_{k,m} | h_{w^\star,M_{1,m},M_{2,m}} \rangle \tag{225}$$

$$\leq \sum_{m=1}^{D}\left(\frac{1}{M_{2,m}^2}\left(\frac{20}{39}\left(1 - \frac{1}{8}\left(\frac{2\pi R_w}{M_1}\right)^2(\beta_m^\star)^2\right) + \frac{1}{54D^2} + \frac{2}{M_{2,m}} + \frac{4}{35D^2}\right)\right) \tag{226}$$

$$\leq \frac{1}{M_2^2}\sum_{m=1}^{D}\left(\frac{20}{39}\left(1 - \frac{1}{8}\left(\frac{2\pi R_w}{M_1}\right)^2(\beta_m^\star)^2\right) + \frac{1}{54D^2} + \frac{2}{M_2} + \frac{4}{35D^2}\right) \tag{227}$$

$$\leq \frac{1}{M_2^2}\left(\frac{20}{39}D - \frac{1}{8D}\left(\frac{2\pi R_w}{M_1}\right)^2 + \frac{2}{15D} + \frac{2D}{M_2}\right). \tag{228}$$

In the third line, we use that $M_{2,m} = mM_2$ by definition and $m \geq 1$. In the last line, we use that $D^2 \geq D$ and $\|\beta^\star\|_2^2 \geq 1/D$.

Thus, we see that if $|T - \ell M_1/w_k^\star| \not\leq 1$ for any integer ℓ, then this upper bound on the sum of expectation values must be satisfied. Recall that QSQs only approximate the expectation value up to some tolerance τ. By our choice of τ, we have

$$|\alpha_m - \langle h_{w^\star,M_{1,m},M_{2,m}} | O_{k,m} | h_{w^\star,M_{1,m},M_{2,m}} \rangle| \leq \frac{1}{2D^2M_2^2}\left(\frac{2}{15} - \frac{1}{8}\left(\frac{2\pi R_w}{M_1}\right)^2 + \frac{2D^2}{M_2}\right). \tag{229}$$

By choosing the condition

$$\sum_{m=1}^{D}\alpha_m \leq \frac{1}{M_2^2}\left(\frac{20}{39}D + \frac{1}{2D}\left(\frac{2}{15} - \frac{1}{8}\left(\frac{2\pi R_w}{M_1}\right)^2 + \frac{2D^2}{M_2}\right)\right), \tag{230}$$

we can ensure that

$$\sum_{m=1}^{D} \langle h_{w^\star,M_{1,m},M_{2,m}} | O_{k,m} | h_{w^\star,M_{1,m},M_{2,m}} \rangle \leq \sum_{m=1}^{D}\alpha_m + \frac{1}{2DM_2^2}\left(\frac{2}{15} - \frac{1}{8}\left(\frac{2\pi R_w}{M_1}\right)^2 + \frac{2D^2}{M_2}\right) \tag{231}$$

$$\leq \frac{1}{M_2^2}\left(\frac{20}{39}D + \frac{1}{D}\left(\frac{2}{15} - \frac{1}{8}\left(\frac{2\pi R_w}{M_1}\right)^2\right) + \frac{2D}{M_2}\right), \tag{232}$$

as required. □

□

With each of these parts, we can put everything together to prove Theorem 5.

Proof of Theorem 5. We first consider the case of $d = 1$. Our algorithm is simply to apply Hallgren's algorithm (Section I B) to our setting using QSQs. Choose the discretization parameters to be $M_1 = \max(70\pi d D^3 R_w, R_w^2/\epsilon_1)$ and $M_2 = cM_1$ for some constant c such that $M_2 \in \mathbb{Z}$ and $c < 1/(8\pi D R_w)$. By Lemma 6, we know that there exists a discretization $h_{w^\star,M_1,M_2}$ of the target function $g_{w^\star}$ such that $h_{w^\star,M_1,M_2}$ is (33/35)-pseudoperiodic with period $M_1/w^\star$ by our choice of M_1, M_2.

Recall that by definition of $\mathcal{S}_w$ that $w_j \geq R_w/d^2$. This gives an upper bound on the period, which we denote as $A \triangleq M_1 d^2/R_w$. We carry this d factor through to avoid losing track of it. Choose the truncation parameter $R \geq 6(1/2+\tau)A^2$.

Then, we want to apply period finding to $h_{w^\star,M_1,M_2}$ using our QSQ access for discretization/truncation parameters M_1, M_2, R as chosen above. With Theorem 6, we fulfill all of the conditions to apply the irrational period finding subroutine from Hallgren's algorithm [7] reviewed in Section I B. Note that the main quantum part of the algorithm (Step 2 in Algorithm 1) is the same as standard period finding, i.e., simply quantum Fourier sampling. The classical postprocessing and analysis is mainly what differs. Thus, we can use the same QSQ operator as from Section IV A 1, namely O given in Equation (111), to apply the quantum part of this algorithm. In particular, this applies the QFT over $q = 2R$ and measures.

We can repeat the analysis of Hallgren's algorithm (Algorithm 1) Steps 3-5 to account for the noise $\tau \geq 0$ in the QSQs. From the analysis of Hallgren's algorithm [7], the outputs of the QSQs are some numbers α, β such that

$$|\alpha - b| \leq \tau, \quad \left| b - \frac{kRw^\star}{M_1} \right| \leq \frac{1}{2} \tag{233}$$

$$|\beta - c| \leq \tau, \quad \left| c - \frac{\ell Rw^\star}{M_1} \right| \leq \frac{1}{2} \tag{234}$$

for some integers $k, \ell \geq 1$. We want to show that k/ℓ is a convergent in the continued fraction expansion of α/β. We use the fact that if x is any irrational number, $e/f \in \mathbb{Q}$, and $|x - e/f| \leq 1/(2f^2)$, then e/f is a convergent in the continued fraction expansion of x [11]. We write

$$\alpha = b + \tau_k, \quad b = \frac{kR}{S} + \epsilon_k, \quad |\tau_k| \leq \tau, |\epsilon_k| \leq \frac{1}{2} \tag{235}$$

$$\beta = c + \tau_\ell, \quad c = \frac{\ell R}{S} + \epsilon_\ell, \quad |\tau_\ell| \leq \tau, |\epsilon_k| \leq \frac{1}{2}, \tag{236}$$

where we denote $S \triangleq M_1/w^\star$ as the period of our target function for simplicity. Without loss of generality, suppose that $1 \leq k \leq \ell \leq S$. Then, we have

$$\begin{aligned}
\left| \frac{\alpha}{\beta} - \frac{k}{\ell} \right| &= \left| \frac{kR + S(\epsilon_k + \tau_k)}{\ell R + S(\epsilon_\ell + \tau_\ell)} - \frac{k}{\ell} \right| && (237)\\
&= \left| \frac{S(\ell(\epsilon_k + \tau_k) - k(\epsilon_\ell + \tau_\ell))}{\ell^2 R - S(\epsilon_\ell + \tau_\ell)\ell} \right| && (238)\\
&\leq \left| \frac{S(\ell + k)}{\frac{1}{1/2+\tau}(\ell^2 R - S(1/2+\tau)\ell)} \right| && (239)\\
&\leq \left| \frac{2\ell S}{6\ell^2 S^2 - S\ell} \right| && (240)\\
&= \left| \frac{2\ell S}{2\ell(3\ell S^2 - S/2)} \right| && (241)\\
&= \left| \frac{1}{3\ell S - 1/2} \right| && (242)\\
&\leq \frac{1}{3\ell^2 - 1/2} && (243)\\
&\leq \frac{1}{2\ell^2}. && (244)
\end{aligned}$$

Here, in the third line, we use $|\epsilon_k + \tau_k| \leq 1/2 + \tau$. In the fourth line, we use our choice of $R \geq 6(1/2+\tau)A^2 \geq 6(1/2+\tau)S^2$ since $A \geq S$ and $k \leq \ell$. In the seventh line, we use $\ell \leq S$. Finally, in the

last line, we use that $3\ell^2 - 1/2 \geq 2\ell^2$ for $\ell \geq 1$. This shows that k/ℓ is a convergent in the continued fraction expansion of α/β.

Now, by Step 4 of Algorithm 1, when k/ℓ is a convergent in the continued fraction expansion of α/β, we want to show that either $\lfloor kR/\alpha \rfloor$ or $\lceil kR/\alpha \rceil$ is close to the period S for some k. We denote $\lfloor kR/\alpha \rceil$ to denote rounding to the closest integer. In particular, we will show that $|S - \lfloor kR/\alpha \rceil| \leq 1$. Again, we write α as in Equation (235). Then,

$$\frac{kR}{\alpha} = kR\left(\frac{1}{\frac{kR}{S} + \epsilon_k + \tau_k}\right) = \frac{S}{1 + \frac{(\epsilon_k + \tau_k)S}{kR}} = \frac{S}{1+\gamma}, \tag{245}$$

where in the last equality, we define

$$\gamma \triangleq \frac{(\epsilon_k + \tau_k)S}{kR}. \tag{246}$$

Notice that

$$|\gamma| \leq \frac{(1/2+\tau)S}{kR} \leq \frac{S}{6kS^2} = \frac{1}{6kS} \leq \frac{1}{6S}, \tag{247}$$

where in the first inequality, we use that $|\epsilon_k + \tau_k| \leq 1/2 + \tau$. In the second inequality, we use our choice of $R \geq 6(1/2+\tau)S^2$. In the last inequality, we use $k \geq 1$. Now, we can write

$$\frac{kR}{\alpha} = \frac{S}{1+\gamma} = S - \frac{S\gamma}{1+\gamma}, \quad \left|\frac{S\gamma}{1+\gamma}\right| < \frac{1}{2}. \tag{248}$$

Thus, we see that $|S - \lfloor kR/\alpha \rceil| \leq 1$, as required. Overall, this shows that Hallgren's algorithm correctly recovers the period S even with noise from QSQs, as long as R is chosen large enough.

Now, we analyze the number of QSQs that the algorithm requires. Step 2 of Algorithm 1 requires two QSQs as we are applying quantum Fourier sampling twice. The only other part of the algorithm that requires QSQs is the verification subroutine, which uses D QSQs each time it is called. In Step 4 of Algorithm 1, this verification procedure must be repeated for each convergent in the continued fraction expansion of α/β, where α and β are the outputs from quantum Fourier sampling via the noisy QSQs. Since we assume that the QSQs output rational numbers[4], then α/β is a rational number, which has a finite continued fraction expansion. In fact, it is well known that the continued fraction expansion for rational numbers α/β can be computed via the steps of Euclid's algorithm on the numerator and denominator (see, e.g., the discussion after Theorem 161 in [33]). Moreover, Euclid's algorithm requires a number of steps scaling logarithmically in the numbers it is run on. Thus, in our case, then we must run the verification procedure at most $\mathcal{O}(\log(S)) = \mathcal{O}(\log A) = \mathcal{O}\left(\log\left(M_1 d^2/R_w\right)\right)$ times, which uses $\mathcal{O}(D\log\left(M_1 d^2/R_w\right))$ QSQs in total.

Overall, this shows that we can find an integer a within 1 of $M_1/w^\star$ with some probability using $\mathcal{O}(D\log\left(M_1 d^2/R_w\right))$ QSQs. In particular, a satisfies

$$\frac{a}{M_1} \in \left[\frac{1}{w^\star} \pm \frac{1}{M_1}\right] \tag{249}$$

with probability $\Omega(\eta^2/\log^4(A))$, where $\eta = 33/35$. We want to choose M_1 such that M_1/a is close to $w^\star$. For this, we use the fact that the relative error for $z = 1/x$ is the same as the relative error for x (see, e.g., [34]), i.e., $(\Delta z)/z = (\Delta x)/x$, where Δz and Δx are the uncertainties in z and x, respectively. Thus, taking $z = w^\star, x = 1/w^\star$, we have

$$\frac{\Delta z}{w^\star} = \frac{1/M_1}{1/w^\star}. \tag{250}$$

Solving for Δz, we clearly see that $\Delta z = (w^\star)^2/M_1$. Hence, using the a output from Theorem 1, we can compute $\hat{w} = M_1/a$ satisfying

$$\frac{M_1}{a} \in \left[w^\star \pm \frac{(w^\star)^2}{M_1}\right] \tag{251}$$

[4] As discussed in Section II, the rational numbers are dense in $\mathbb{R}$. Then, if a QSQ outputs an irrational number, we can find a rational number close to it. We can then consider the error in this approximation as a part of the tolerance of the QSQ.

with probability $\Omega(\eta^2/\log^4(A))$, where $\eta = 33/35$. Here, in order to guarantee that $|\hat{w} - w^\star| \leq \epsilon_1$ for some $\epsilon_1 > 0$, we should choose the discretization parameter M_1 as $M_1 \geq R_w^2/\epsilon_1$, which is satisfied by our choice of M_1. Then, the success probability simplifies to

$$p = \Omega\left(\frac{1}{\log^4(A)}\right) = \Omega\left(\frac{1}{\log^4(M_1 d^2/R_w)}\right). \tag{252}$$

To boost the success probability to at least $1-\delta$ (using the verification procedure to check if the period is correct), for some $\delta > 0$, we can repeat this $\mathcal{O}(\log(1/\delta)/p)$ times. In total, this is

$$\mathcal{O}\left(\log\left(\frac{1}{\delta}\right)\log^4\left(\frac{M_1 d^2}{R_w}\right)\right) \tag{253}$$

repetitions, where in each repetition, we use $\mathcal{O}(D\log\bigl(M_1 d^2/R_w\bigr))$ QSQs from the above analysis.

Finally, the generalization to arbitrary $d \geq 1$ is straightforward, using the observable O_j from Equation (114). Here, we only perform quantum Fourier sampling one coordinate at a time. In this case, the function we are Fourier sampling from is effectively

$$g_{w^\star,j}(x_j; x_{-j}) \triangleq \sum_{k=1}^{D} \beta_k^\star \cos\bigl(2\pi k(x_j w_j^\star + x_{-j}^\intercal w_{-j}^\star)\bigr), \tag{254}$$

where x_{-j} denotes the vector x with all coordinates except the j-th one. Here, x_{-j} is a fixed vector because the observable O_j collapses the register storing all but the j-th coordinate of the input. Thus, we can consider the function

$$\tilde{g}_j(z; x_{-j}) \triangleq \sum_{k=1}^{D} \beta_k^\star \cos\bigl(2\pi(z + x_{-j}^\intercal w_{-j}^\star)\bigr). \tag{255}$$

This function clearly satisfies the conditions of Lemma 6. Thus, the resulting discretized function $h_{w^\star,M_1,M_2}$ with the x_{-j} coordinates fixed is also (33/35)-pseudoperiodic with period $M_1/w_j^\star$. Hence, we can apply Hallgren's algorithm one coordinate at a time, learning $M_1/w_j^\star$. It is clear that the argument above still holds for this case as well. For this, we need to repeat the algorithm to learn each entry of the vector $w^\star \in \mathbb{R}^d$ at a time. Altogether, this gives the bound from Theorem 5. □

B. Learning the outer function via gradient methods

From the previous section (in particular, Theorem 5), we have seen that we can obtain an approximation $\hat{w}$ of $w^\star$ such that $\|\hat{w} - w^\star\|_\infty \leq \epsilon_1$ with high probability, for some $\epsilon_1 > 0$. In this section, we complete the algorithm for the uniform case by leveraging this approximation of $w^\star$ to learn the outer periodic function $\tilde{g} : \mathbb{R} \to [-1, 1]$ via classical gradient methods. We emphasize here that this portion of the algorithm is purely classical, where we have classical access to the loss function and its gradients. Recall that we assume that $\tilde{g}$ takes the specific form given in Equation (10), reproduced here for convenience:

$$\tilde{g}(y) = \sum_{j=1}^{D} \beta_j^\star \cos(2\pi j y), \quad \|\beta^\star\|_1 = 1, \tag{256}$$

for some constant $D > 0$. In this way, then our target function can be written as

$$g_{w^\star}(x) = \tilde{g}(x^\intercal w^\star) = \sum_{j=1}^{D} \beta_j^\star \cos(2\pi j x^\intercal w^\star). \tag{257}$$

Also recall that our ultimate goal is to find a good predictor $f_\theta(x)$ that minimizes the objective function given by

$$\mathcal{L}_{w^\star}(\theta) = \mathop{\mathbb{E}}_{x\sim\varphi^2}[(f_\theta(x) - g_{w^\star}(x))^2], \tag{258}$$

where θ are some parameters that we want to learn and φ^2 in this case is a uniform distribution. Here, because we assume this simple form of $\tilde{g}$, then the predictors take a similar form

$$f_\beta(x) = \sum_{j=1}^{D} \beta_j \cos(2\pi j x^\intercal \hat{w}), \tag{259}$$

where $\hat{w}$ is our approximation of $w^\star$ from Theorem 5. Thus, the parameters that we want to learn here are given by the $\beta \in \mathbb{R}^d$. Then, our loss function can be written more explicitly as

$$\mathcal{L}_{w^\star}(\beta) = \int_{x\sim\varphi^2} \left(\sum_{j=1}^{D} \beta_j^\star \cos(2\pi j x^\intercal w^\star) - \sum_{j=1}^{D} \beta_j \cos(2\pi j x^\intercal \hat{w}) \right)^2 dx. \tag{260}$$

As in the classical hardness result [12], our algorithm is given access to this loss function and its gradients. Using this, we design a classical algorithm that can efficiently find a predictor specified by parameters $\hat{\beta}$ such that $\mathcal{L}_{w^\star}(\hat{\beta}) \leq \epsilon$ for a given precision $\epsilon > 0$.

Recall in the previous section that we needed to discretize and truncate our access to the target function $g_{w^\star}$. We no longer require discretization since classically we can perform computations up to arbitrary precision, but we still truncate with truncation parameter R. Namely, we consider φ^2 as the uniform distribution over an ℓ_1-ball of radius R centered at the origin in $\mathbb{R}^d$. To show that $\mathcal{L}_{w^\star}(\hat{\beta}) \leq \epsilon$, we appropriately choose R and ϵ_1 sufficiently large/small enough, respectively.

Theorem 7 (Learning $\tilde{g}$ Guarantee; Uniform Case)**.** *Let $\epsilon > 0$. Let $w^\star \in \mathbb{R}^d$ be unknown with norm $R_w > 0$. Let $g_{w^\star} : \mathbb{R}^d \to [-1,1]$ be defined as $g_{w^\star}(x) = \tilde{g}(x^\intercal w^\star)$ for $\tilde{g}$ given in Equation (10). Choose*

$$R = \tilde{\Omega}\left(\max\left(\frac{D^2}{\epsilon}, \frac{D^2\sqrt{d}}{R_w \epsilon}, \frac{D^{5/2}}{\sqrt{\epsilon}}, \frac{D^{3/2}\sqrt{d}}{R_w\sqrt{\epsilon}} \right) \right), \tag{261}$$

$$\epsilon_1 = \tilde{\mathcal{O}}\left(\min\left(\frac{\epsilon^3}{D^6 d}, \frac{\epsilon^{3/2}}{D^{13/2} d}, \frac{R_w}{D\sqrt{d}} \right) \right). \tag{262}$$

Suppose we have an approximation $\hat{w} \in \mathbb{R}^d$ such that $\|\hat{w} - w^\star\|_\infty \leq \epsilon_1$. Then, there exists a classical algorithm with access to the loss function from Equation (260) and its derivatives that can efficiently find a parameters $\hat{\beta} \in \mathbb{R}^d$ such that $\mathcal{L}_{w^\star}(\hat{\beta}) \leq \epsilon$. Moreover, this algorithm requires at most

$$t = \Theta\left(\log\left(\sqrt{\frac{D}{\epsilon}} \right) \right) \tag{263}$$

iterations of gradient descent.

The rest of this section is dedicated to proving this theorem. The algorithm is simple: just run gradient descent using the loss function to estimate the parameters $\beta^\star$. We prove this using arguments from convex optimization (see, e.g., [35]). Throughout the proof, we require some technical lemmas bounding integrals of exponential functions over our truncated domain, which we relegate to Section IV C.

Proof sketch. The proof of Theorem 7 is fairly technical, but the idea is simple. First, we show that the gradients are informative, i.e., taking the derivative of our loss function with respect to each of the parameters β_k indeed reflects how far β_k is from the true parameter $\beta_k^\star$. Then, we can just apply the standard gradient descent algorithm (see, e.g., [35]). Much of the work then goes into choosing the parameters (e.g., number of iterations to run gradient descent, how accurate we need period finding to be, etc.) to guarantee that the value of the loss function is small. Throughout, we use the following notation: ϵ_1 denotes the error for our estimate of $w^\star$ (in ℓ_∞-norm), ϵ_2 quantifies how informative the gradients are, ϵ_3 denotes the error for our estimate of $\beta^\star$ (in ℓ_2-norm), and ϵ is the desired value of the loss function.

First, we show that the gradients are informative in the following lemma. The idea is that we can choose R sufficiently large and ϵ_1 sufficiently small so that $\partial\mathcal{L}_{w^\star}/\partial\beta_k$ is close to $(\beta_k - \beta_k^\star)$.

Lemma 7 (Informative gradients)**.** *Let $w^\star \in \mathbb{R}^d$ be unknown with norm $R_w > 0$. Let $g_{w^\star} : \mathbb{R}^d \to [-1,1]$ be defined as $g_{w^\star}(x) = \tilde{g}(x^\intercal w^\star)$ for $\tilde{g}$ given in Equation (10). Suppose we have an approximation $\hat{w} \in \mathbb{R}^d$ such that $\|\hat{w} - w^\star\|_\infty \leq \epsilon_1$, for $0 < \epsilon_1 \leq R_w/(D\sqrt{d})$. Then for any $k \in [D]$,*

$$\left| \frac{\partial \mathcal{L}_{w^\star}}{\partial \beta_k} - (\beta_k - \beta_k^\star) \right| \leq \left(\frac{\sqrt{d}}{2\pi R(R_w - \sqrt{d}\epsilon_1)} + \frac{10\pi^2 D^2 d R^2 \epsilon_1}{3} \right) \max(|\beta_k^\star|, |\beta_k|) \tag{264}$$

$$+ \frac{\sqrt{d}}{\pi R(R_w - D\sqrt{d}\epsilon_1)} + \sum_{\substack{j=1 \\ j\neq k}}^{D} |\beta_j| \frac{\sqrt{d}}{\pi R(R_w - \sqrt{d}\epsilon_1)}. \tag{265}$$

Proof. Recall that our loss function is

$$\mathcal{L}_{w^\star}(\beta) = \int\limits_{x\sim\varphi^2} \left(\sum_{j=1}^{D} \beta_j^\star \cos(2\pi j x^\intercal w^\star) - \sum_{j=1}^{D} \beta_j \cos(2\pi j x^\intercal \hat{w})\right)^2 dx. \tag{266}$$

Taking the derivative of this with respect to β_k, we have

$$\frac{\partial \mathcal{L}_{w^\star}}{\partial \beta_k} = -2 \int_{x\sim\varphi^2} \cos(2\pi k x^\intercal \hat{w}) \left(\sum_{j=1}^{D} \beta_j^\star \cos(2\pi j x^\intercal w^\star) - \sum_{j=1}^{D} \beta_j \cos(2\pi j x^\intercal \hat{w})\right) dx. \tag{267}$$

Separating out terms with $k \neq j$, we have

$$\frac{\partial \mathcal{L}_{w^\star}}{\partial \beta_k} = 2\beta_k \int_{x\sim\varphi^2} \cos^2(2\pi k x^\intercal \hat{w})\, dx - 2\beta_k^\star \int_{x\sim\varphi^2} \cos(2\pi k x^\intercal \hat{w}) \cos(2\pi k x^\intercal w^\star)\, dx \tag{268}$$

$$- 2 \int_{x\sim\varphi^2} \sum_{\substack{j=1 \\ j\neq k}}^{D} \beta_j^\star \cos(2\pi k x^\intercal \hat{w}) \cos(2\pi j x^\intercal w^\star)\, dx \tag{269}$$

$$+ 2 \int_{x\sim\varphi^2} \sum_{\substack{j=1 \\ j\neq k}}^{D} \beta_j \cos(2\pi k x^\intercal \hat{w}) \cos(2\pi j x^\intercal \hat{w})\, dx. \tag{270}$$

We can upper and lower bound this expression using the integral bounds from Section IV C. First, to upper bound, we can use Corollary 5, Lemma 12, Corollary 8, and Lemma 14, for each of the terms respectively. Then, we have

$$\frac{\partial \mathcal{L}_{w^\star}}{\partial \beta_k} \leq 2\beta_k \int_{x\sim\varphi^2} \cos^2(2\pi k x^\intercal \hat{w})\, dx - 2\beta_k^\star \int_{x\sim\varphi^2} \cos(2\pi k x^\intercal \hat{w}) \cos(2\pi k x^\intercal w^\star)\, dx \tag{271}$$

$$+ 2 \sum_{\substack{j=1 \\ j\neq k}}^{D} |\beta_k^\star| \left| \int_{x\sim\varphi^2} \cos(2\pi k x^\intercal \hat{w}) \cos(2\pi j x^\intercal w^\star)\, dx \right| + 2 \sum_{\substack{j=1 \\ j\neq k}}^{D} |\beta_k| \left| \int_{x\sim\varphi^2} \cos(2\pi k x^\intercal \hat{w}) \cos(2\pi j x^\intercal \hat{w})\, dx \right| \tag{272}$$

$$\leq \beta_k - \beta_k^\star + \frac{\sqrt{d}}{4\pi R(R_w - \sqrt{d}\epsilon_1)} |\beta_k| + \left(\frac{\sqrt{d}}{4\pi R_w R} + \frac{10\pi^2 D^2 d R^2 \epsilon_1}{3} \right) |\beta_k^\star| \tag{273}$$

$$+ \sum_{\substack{j=1 \\ j\neq k}}^{D} |\beta_j^\star| \frac{\sqrt{d}}{\pi R(R_w - D\sqrt{d}\epsilon_1)} + \sum_{\substack{j=1 \\ j\neq k}}^{D} |\beta_j| \frac{\sqrt{d}}{\pi R(R_w - \sqrt{d}\epsilon_1)} \tag{274}$$

$$\leq (\beta_k - \beta_k^\star) + \left(\frac{\sqrt{d}}{2\pi R(R_w - \sqrt{d}\epsilon_1)} + \frac{10\pi^2 D^2 d R^2 \epsilon_1}{3} \right) \max(|\beta_k^\star|, |\beta_k|) \tag{275}$$

$$+ \frac{\sqrt{d}}{\pi R(R_w - D\sqrt{d}\epsilon_1)} + \sum_{\substack{j=1 \\ j\neq k}}^{D} |\beta_j| \frac{\sqrt{d}}{\pi R(R_w - \sqrt{d}\epsilon_1)} \tag{276}$$

where in the second inequality, we use Corollary 5, Lemma 12, Corollary 8, and Lemma 14 for each term respectively. In the third inequality, we use that $\max(|\beta_k^\star|, |\beta_k|) \geq |\beta_k^\star|, |\beta_k|$ and $\|\beta^\star\|_1 = 1$ so that $\sum_{j\neq k} |\beta_j^\star| \leq 1$. We also use that $R_w \geq R_w - \sqrt{d}\epsilon_1$.

We can also obtain a similar lower bound using Corollary 4, Lemma 13, Corollary 8, and Lemma 14.

$$\frac{\partial \mathcal{L}_{w^\star}}{\partial \beta_k} \geq (\beta_k - \beta_k^\star) - \frac{\sqrt{d}}{4\pi R(R_w - \sqrt{d}\epsilon_1)} |\beta_k| - \left(\frac{\sqrt{d}}{4\pi R_w R} + 2\pi D d \epsilon_1 R \right) |\beta_k^\star| \tag{277}$$

$$- 2 \int_{x\sim\varphi^2} \sum_{\substack{j=1 \\ j\neq k}}^{D} \beta_j^\star \cos(2\pi k x^\intercal \hat{w}) \cos(2\pi j x^\intercal w^\star)\, dx + 2 \int_{x\sim\varphi^2} \sum_{\substack{j=1 \\ j\neq k}}^{D} \beta_j \cos(2\pi k x^\intercal \hat{w}) \cos(2\pi j x^\intercal \hat{w})\, dx \tag{278}$$

$$\geq (\beta_k - \beta_k^\star) - \left(\frac{\sqrt{d}}{2\pi R(R_w - \sqrt{d}\epsilon_1)} + 2\pi D d\epsilon_1 R \right) \max(|\beta_k^\star|, |\beta_k|) \tag{279}$$

$$- \left| -2 \int_{x\sim\varphi^2} \sum_{\substack{j=1 \\ j\neq k}}^{D} \beta_j^\star \cos(2\pi k x^\intercal \hat{w}) \cos(2\pi j x^\intercal w^\star)\, dx \right| - \left| 2 \int_{x\sim\varphi^2} \sum_{\substack{j=1 \\ j\neq k}}^{D} \beta_j \cos(2\pi k x^\intercal \hat{w}) \cos(2\pi j x^\intercal \hat{w})\, dx \right| \tag{280}$$

$$\geq (\beta_k - \beta_k^\star) - \left(\frac{\sqrt{d}}{2\pi R(R_w - \sqrt{d}\epsilon_1)} + 2\pi D d\epsilon_1 R \right) \max(|\beta_k^\star|, |\beta_k|) \tag{281}$$

$$- \frac{\sqrt{d}}{\pi R(R_w - D\sqrt{d}\epsilon_1)} - \sum_{\substack{j=1 \\ j\neq k}}^{D} |\beta_j| \frac{\sqrt{d}}{\pi R(R_w - \sqrt{d}\epsilon_1)} \tag{282}$$

$$\geq (\beta_k - \beta_k^\star) - \left(\frac{\sqrt{d}}{2\pi R(R_w - \sqrt{d}\epsilon_1)} + \frac{10\pi^2 D^2 d R^2 \epsilon_1}{3} \right) \max(|\beta_k^\star|, |\beta_k|) \tag{283}$$

$$- \frac{\sqrt{d}}{\pi R(R_w - D\sqrt{d}\epsilon_1)} - \sum_{\substack{j=1 \\ j\neq k}}^{D} |\beta_j| \frac{\sqrt{d}}{\pi R(R_w - \sqrt{d}\epsilon_1)}. \tag{284}$$

In the first inequality, we use Corollary 4 and Lemma 13. In the second inequality, we use that $\max(|\beta_k^\star|, |\beta_k|) \geq |\beta_k|, |\beta_k^\star|$ and $R_w \geq R_w - \sqrt{d}\epsilon_1$. In the third inequality, we use Lemma 14 and Corollary 8. We also use that $\|\beta^\star\|_1 = 1$ so that $\sum_{j\neq k} |\beta_j^\star| \leq 1$. In the last inequality, we use that $2\pi D d\epsilon_1 R \leq 10\pi^2 D^2 d R^2 \epsilon_1 / 3$.

Combining these two inequalities, we have that

$$\left| \frac{\partial \mathcal{L}_{w^\star}}{\partial \beta_k} - (\beta_k - \beta_k^\star) \right| \leq \left(\frac{\sqrt{d}}{2\pi R(R_w - \sqrt{d}\epsilon_1)} + \frac{10\pi^2 D^2 d R^2 \epsilon_1}{3} \right) \max(|\beta_k^\star|, |\beta_k|) \tag{285}$$

$$+ \frac{\sqrt{d}}{\pi R(R_w - D\sqrt{d}\epsilon_1)} + \sum_{\substack{j=1 \\ j\neq k}}^{D} |\beta_j| \frac{\sqrt{d}}{\pi R(R_w - \sqrt{d}\epsilon_1)}. \tag{286}$$

□

Now, we can use standard gradient descent, which converges as follows.

Lemma 8 (Gradient descent convergence)**.** *Let $\epsilon_1, \epsilon_2 > 0$. Let $w^\star \in \mathbb{R}^d$ be unknown with norm $R_w > 0$. Let $g_{w^\star} : \mathbb{R}^d \to [-1, 1]$ be defined as $g_{w^\star}(x) = \tilde{g}(x^\intercal w^\star)$ for $\tilde{g}$ given in Equation (10). Suppose we have an approximation $\hat{w} \in \mathbb{R}^d$ such that $\|\hat{w} - w^\star\|_\infty \leq \epsilon_1$. Also, suppose that*

$$\left| \frac{\partial \mathcal{L}_{w^\star}}{\partial \beta_k}(\beta_k^{(t)}) - (\beta_k^{(t)} - \beta_k^\star) \right| < \epsilon_2 \tag{287}$$

for all $k \in [D]$. Here, t denotes the step of gradient descent. Then, gradient descent with step size $\eta = \mathcal{O}(1)$ with $0 < \eta < 1$ and initial point $\beta^{(0)} = 0$ converges as follows:

$$|\beta_k^{(t+1)} - \beta_k^\star| \leq (1 - \eta)^t + \eta t \epsilon_2. \tag{288}$$

Proof. This proof is straightforward following the standard gradient descent rule

$$\beta_k^{(t+1)} = \beta_k^{(t)} - \eta \frac{\partial \mathcal{L}_{w^\star}}{\partial \beta_k}(\beta_k^{(t)}). \tag{289}$$

Plugging this in and applying Eq. (287), we have

$$|\beta_k^{(t+1)} - \beta_k^\star| = \left| \beta_k^{(t)} - \eta \frac{\partial \mathcal{L}_{w^\star}}{\partial \beta_k}(\beta_k^{(t)}) - \beta_k^\star \right| \tag{290}$$

$$\leq \left| \beta_k^{(t)} - \beta_k^\star - \eta(\beta_k^{(t)} - \beta_k^\star) + \eta\epsilon_2 \right| \tag{291}$$

$$\leq (1-\eta)|\beta_k^{(t)} - \beta_k^\star| + \eta\epsilon_2. \tag{292}$$

Applying this inequality recursively, we have

$$|\beta_k^{(t+1)} - \beta_k^\star| \leq (1-\eta)^t|\beta_k^{(0)} - \beta_k^\star| + \eta\sum_{i=1}^{t}(1-\eta)^i\epsilon_2 \tag{293}$$

$$\leq (1-\eta)^t|\beta_k^{(0)} - \beta_k^\star| + \eta t\epsilon_2, \tag{294}$$

where the last line follows because $0 < \eta < 1$ so that $0 < 1-\eta < 1$. Now, because we initialize to $\beta^{(0)} = 0$, then

$$|\beta_k^{(0)} - \beta_k^\star| = |\beta_k^\star| \leq 1, \tag{295}$$

where $|\beta_k^\star| \leq 1$ because $\|\beta_k^\star\|_1 = 1$. Thus, we have

$$|\beta_k^{(t+1)} - \beta_k^\star| \leq (1-\eta)^t + \eta t\epsilon_2, \tag{296}$$

as claimed. □

To help us choose parameters such as ϵ_1, ϵ_2, and t properly, we also need to show that the updated parameters via gradient descent do not become too large. In particular, recall from Equation (10) that the true parameters satisfy $|\beta_k^\star| < 1$ because $\|\beta^\star\|_1 = 1$. The following lemma states that the parameters found via gradient descent are not much larger than this.

Lemma 9 (Parameter bound)**.** *Let $w^\star \in \mathbb{R}^d$ be unknown with norm $R_w > 0$. Let $g_{w^\star} : \mathbb{R}^d \to [-1,1]$ be defined as $g_{w^\star}(x) = \tilde{g}(x^\intercal w^\star)$ for $\tilde{g}$ given in Equation (10). Suppose we have an approximation $\hat{w} \in \mathbb{R}^d$ such that $\|\hat{w} - w^\star\|_\infty \leq \epsilon_1$. Suppose*

$$R \geq \max\left(D^2, \frac{16D\sqrt{d}}{\pi R_w}\right), \quad \epsilon_1 \leq \min\left(\frac{3}{40\pi^2 D^6 d}, \frac{R_w}{2D\sqrt{d}}\right). \tag{297}$$

Then,

$$|\beta_k^{(t)}| < 2 \tag{298}$$

for all $k \in [D]$. Here, $\beta_k^{(t)}$ denotes the parameters at the t-th step of gradient descent.

Proof. We prove this by induction on the t steps of gradient descent. For the base case of $t = 0$, this is clearly satisfied by our choice of initialization. Namely, we initialize to $\beta_k^{(0)} = 0$ for all k. Thus, we clearly have $|\beta_k^{(0)}| = 0 < 2$ for all $k \in [D]$.

For the inductive step, suppose that for some step $t > 0$ that $|\beta_k^{(t)}| < 2$ for all $k \in [D]$. We want to prove that $|\beta_k^{(t+1)}| < 2$ for all $k \in [D]$. Let $k \in [D]$. By Lemma 7,

$$\left|\frac{\partial \mathcal{L}_{w^\star}}{\partial \beta_k} - (\beta_k - \beta_k^\star)\right| \leq \left(\frac{\sqrt{d}}{2\pi R(R_w - \sqrt{d}\epsilon_1)} + \frac{10\pi^2 D^2 d R^2 \epsilon_1}{3}\right)\max(|\beta_k^\star|, |\beta_k|) \tag{299}$$

$$+ \frac{\sqrt{d}}{\pi R(R_w - D\sqrt{d}\epsilon_1)} + \sum_{\substack{j=1\\j\neq k}}^{D}|\beta_j|\frac{\sqrt{d}}{\pi R(R_w - \sqrt{d}\epsilon_1)}. \tag{300}$$

Note that the condition needed for Lemma 7 (i.e., $\epsilon_1 \leq R_w/(D\sqrt{d})$) is satisfied for our choice of ϵ_1. Using that $D\epsilon_1 \geq \epsilon_1$ (since $D \geq 1$), we can simplify this:

$$\left|\frac{\partial \mathcal{L}_{w^\star}}{\partial \beta_k} - (\beta_k - \beta_k^\star)\right| \leq \left(\frac{\sqrt{d}}{2\pi R(R_w - D\sqrt{d}\epsilon_1)} + \frac{10\pi^2 D^2 d R^2 \epsilon_1}{3}\right)\max(|\beta_k^\star|, |\beta_k|) \tag{301}$$

$$+ \frac{\sqrt{d}}{\pi R(R_w - D\sqrt{d}\epsilon_1)} + \sum_{\substack{j=1\\j\neq k}}^{D}|\beta_j|\frac{\sqrt{d}}{\pi R(R_w - D\sqrt{d}\epsilon_1)} \tag{302}$$

$$= \left(\frac{\sqrt{d}}{2\pi R(R_w - D\sqrt{d}\epsilon_1)} + \frac{10\pi^2 D^2 d R^2 \epsilon_1}{3} \right) \max(|\beta_k^\star|, |\beta_k|) + \frac{\sqrt{d}\sum_{j=1, j\neq k}^{D} |\beta_j| + \sqrt{d}}{\pi R(R_w - D\sqrt{d}\epsilon_1)}. \tag{303}$$

Evaluating at $\beta_k = \beta_k^{(t)}$, we have

$$\left| \frac{\partial \mathcal{L}_{w^\star}}{\partial \beta_k}(\beta_k^{(t)}) - (\beta_k^{(t)} - \beta_k^\star) \right| \tag{304}$$

$$\leq \left(\frac{\sqrt{d}}{2\pi R(R_w - D\sqrt{d}\epsilon_1)} + \frac{10\pi^2 D^2 d R^2 \epsilon_1}{3} \right) \max(|\beta_k^\star|, |\beta_k^{(t)}|) + \frac{\sqrt{d}\sum_{j=1, j\neq k}^{D} |\beta_j^{(t)}| + \sqrt{d}}{\pi R(R_w - D\sqrt{d}\epsilon_1)} \tag{305}$$

$$\leq \frac{\sqrt{d}}{\pi R(R_w - D\sqrt{d}\epsilon_1)} + \frac{20\pi^2 D^2 d R^2 \epsilon_1}{3} + \frac{2D+1}{\pi R(R_w/\sqrt{d} - D\epsilon_1)} \tag{306}$$

$$= \frac{20\pi^2 D^2 d R^2 \epsilon_1}{3} + \frac{2D+2}{\pi R(R_w/\sqrt{d} - D\epsilon_1)}, \tag{307}$$

where in the second to last line we used the inductive hypothesis. We will use this to bound the parameters after one step of gradient descent. Recall that the update rule for gradient descent is

$$\beta_k^{(t+1)} = \beta_k^{(t)} - \eta \frac{\partial \mathcal{L}_{w^\star}}{\partial \beta_k}(\beta_k^{(t)}) \tag{308}$$

for a step size $\eta = \mathcal{O}(1)$. Then, using the above inequality, we have

$$|\beta_k^{(t+1)}| = \left| \beta_k^{(t)} - \eta \frac{\partial \mathcal{L}_{w^\star}}{\partial \beta_k}(\beta_k^{(t)}) \right| \tag{309}$$

$$\leq \left| \beta_k^{(t)} - \eta(\beta_k^{(t)} - \beta_k^\star) + \eta \left(\frac{20\pi^2 D^2 d R^2 \epsilon_1}{3} + \frac{2D+2}{\pi R(R_w/\sqrt{d} - D\epsilon_1)} \right) \right| \tag{310}$$

$$< 2(1-\eta) + \eta + \eta \left(\frac{20\pi^2 D^2 d R^2 \epsilon_1}{3} + \frac{2D+2}{\pi R(R_w/\sqrt{d} - D\epsilon_1)} \right) \tag{311}$$

$$= 2 - \eta + \eta \left(\frac{20\pi^2 D^2 d R^2 \epsilon_1}{3} + \frac{2D+2}{\pi R(R_w/\sqrt{d} - D\epsilon_1)} \right) \tag{312}$$

$$\leq 2 - \eta + \eta \left(\frac{20\pi^2 D^2 d R^2 \epsilon_1}{3} + \frac{4D}{\pi R(R_w/\sqrt{d} - D\epsilon_1)} \right), \tag{313}$$

where in the second line, we used Equation (307). In the third line, we used triangle inequality, the inductive hypothesis that $|\beta_k^{(t)}| < 2$, and $|\beta_k^\star| < 1$. In the last line, we use $D \geq 1$. In order to achieve the result, we need

$$-\eta + \eta \left(\frac{20\pi^2 D^2 d R^2 \epsilon_1}{3} + \frac{4D}{\pi R(R_w/\sqrt{d} - D\epsilon_1)} \right) \leq 0. \tag{314}$$

Rearranging, we need to show that

$$\frac{20\pi^2 D^2 d R^2 \epsilon_1}{3} + \frac{4D}{\pi R(R_w/\sqrt{d} - D\epsilon_1)} \leq 1. \tag{315}$$

Consider taking

$$R \geq \max\left(D^2, \frac{16D\sqrt{d}}{\pi R_w} \right), \quad \epsilon_1 \leq \min\left(\frac{3}{40\pi^2 D^6 d}, \frac{R_w}{2D\sqrt{d}} \right). \tag{316}$$

We want to show that these choices of R, ϵ_1 allow us to bound each term on the lefthand side by $1/2$ to obtain the required bound. For the first term, consider taking $\epsilon_1 \leq 3/(40\pi^2 R^3 d)$ and R as in the first element in the max of Equation (316), we have

$$\frac{20\pi^2 D^2 d R^2 \epsilon_1}{3} \leq \frac{D^2}{2R} \leq \frac{1}{2}. \tag{317}$$

Finally, for the last term, using $\epsilon_1 \leq R_w/(2D\sqrt{d})$ and $R \geq 16D\sqrt{d}/(\pi R_w)$, we have

$$\frac{4D}{\pi R(R_w/\sqrt{d} - D\epsilon_1)} \leq \frac{8D\sqrt{d}}{\pi R R_w} \leq \frac{1}{2}. \tag{318}$$

□

With the past three lemmas, we can now begin to set the parameters involved to obtain the desired guarantees. As a corollary of Lemma 9, we can obtain the number of steps t and accuracy of the gradient ϵ_2 needed to achieve a desired accuracy for gradient descent.

Corollary 1 (Convergence steps and accuracy)**.** *Let $w^\star \in \mathbb{R}^d$ be unknown with norm $R_w > 0$ and $|w_i^\star| \geq R_w/d^2$ for all $i \in [d]$. Let $g_{w^\star} : \mathbb{R}^d \to [-1, 1]$ be defined as $g_{w^\star}(x) = \tilde{g}(x^\intercal w^\star)$ for $\tilde{g}$ given in Equation (10). Suppose that*

$$\left| \frac{\partial \mathcal{L}_{w^\star}}{\partial \beta_k}(\beta_k^{(t)}) - (\beta_k^{(t)} - \beta_k^\star) \right| < \epsilon_2 \tag{319}$$

for $\epsilon_2 > 0$, for all $k \in [D]$. Here, t denotes the t-th step of gradient descent. Let $\epsilon_3 > 0$. Then, gradient descent with step size $\eta = \mathcal{O}(1)$ with $0 < \eta < 1$ and initial point $\beta^{(0)} = 0$ requires

$$t = \Theta\left(\log\left(\sqrt{D}/\epsilon_3\right)\right) \tag{320}$$

and

$$\epsilon_2 = \mathcal{O}\left(\frac{\epsilon_3}{\sqrt{D}\log\left(\sqrt{D}/\epsilon_3\right)}\right) \tag{321}$$

to converge such that

$$\left\| \beta^{(t+1)} - \beta^\star \right\|_2 \leq \epsilon_3. \tag{322}$$

Proof. By Lemma 8, we have

$$|\beta_k^{(t+1)} - \beta_k^\star| \leq (1-\eta)^t + \eta t \epsilon_2. \tag{323}$$

Then, in order to have $(1-\eta)^t \leq \epsilon_3/(2\sqrt{D})$, we can use

$$t \log(1-\eta) = \log\left(\frac{\epsilon_3}{2\sqrt{D}}\right). \tag{324}$$

Solving for t, we obtain

$$t = \frac{\log\left(2\sqrt{D}/\epsilon_3\right)}{\log(1/c)} \tag{325}$$

for $c = 1 - \eta < 1$. Since η is a constant, then we obtain the claim. It remains to find ϵ_2 such that

$$\eta t \epsilon_2 < \frac{\epsilon_3}{2\sqrt{D}}. \tag{326}$$

Plugging in our previously found t, then we arrive at

$$\epsilon_2 \leq \frac{\log(1/c)\epsilon_3}{2\eta\sqrt{D}\log\left(2\sqrt{D}/\epsilon_3\right)}, \tag{327}$$

where again taking $\eta = \mathcal{O}(1)$ gives the claim. Putting these two pieces together, we have

$$|\beta_k^{(t+1)} - \beta_k^\star| \leq (1-\eta)^t + \eta t \epsilon_2 \leq \frac{\epsilon_3}{\sqrt{D}}. \tag{328}$$

Finally, we obtain the 2-norm bound

$$\left\| \beta^{(t+1)} - \beta^\star \right\|_2 = \sqrt{\sum_{k=1}^{D} |\beta_k^{(t+1)} - \beta_k^\star|^2} \leq \epsilon_3. \tag{329}$$

□

With this, we have set an accuracy ϵ_2, which we need the gradients to satisfy. Using Lemma 7 and Lemma 9, we show that we can achieve this ϵ_2 accuracy from Corollary 1 by setting the parameters R, ϵ_1 appropriately.

Corollary 2 (Achieving required gradient accuracy)**.** *Let $1 > \epsilon_2, \epsilon_3 > 0$. Let $w^\star \in \mathbb{R}^d$ be unknown with norm $R_w > 0$ and $|w_i^\star| \geq R_w/d^2$. Let $g_{w^\star} : \mathbb{R}^d \to [-1, 1]$ be defined as $g_{w^\star}(x) = \tilde{g}(x^\intercal w^\star)$ for $\tilde{g}$ given in Equation (10). Suppose we have an approximation $\hat{w} \in \mathbb{R}^d$ such that $\|\hat{w} - w^\star\|_\infty \leq \epsilon_1$. Suppose that*

$$R \geq \max\left(\frac{D^2}{\epsilon_2}, \frac{16D\sqrt{d}}{\pi R_w \epsilon_2}\right), \quad \epsilon_1 \leq \min\left(\frac{3\epsilon_2^3}{40\pi^2 D^6 d}, \frac{R_w}{2D\sqrt{d}}\right). \tag{330}$$

Then, we can achieve

$$\left|\frac{\partial \mathcal{L}_{w^\star}}{\partial \beta_k}(\beta_k^{(t)}) - (\beta_k^{(t)} - \beta_k^\star)\right| < \epsilon_2 \tag{331}$$

for all $k \in [D]$, where

$$\epsilon_2 = \mathcal{O}\left(\frac{\epsilon_3}{\sqrt{D}\log\left(\sqrt{D}/\epsilon_3\right)}\right). \tag{332}$$

Here, t denotes the t-th step of gradient descent. Writing the bounds on R and ϵ_1 in terms of ϵ_3, we have

$$R = \tilde{\Omega}\left(\max\left(\frac{D^{5/2}}{\epsilon_3}, \frac{D^{3/2}\sqrt{d}}{R_w \epsilon_3}\right)\right), \quad \epsilon_1 = \tilde{\mathcal{O}}\left(\min\left(\frac{\epsilon_3^2}{D^{13/2} d}, \frac{R_w}{D\sqrt{d}}\right)\right). \tag{333}$$

Proof. We need to show that we can indeed achieve this ϵ_2 error for the gradients. This introduces some constraints on R and ϵ_1. By Equation (307) (since we already proved this parameter bound in Lemma 9 and this result holds given our choice of R, ϵ_1), we have

$$\left|\frac{\partial \mathcal{L}_{w^\star}}{\partial \beta_k}(\beta_k^{(t)}) - (\beta_k^{(t)} - \beta_k^\star)\right| \leq \frac{20\pi^2 D^2 d R^2 \epsilon_1}{3} + \frac{2D + 2}{\pi R(R_w/\sqrt{d} - D\epsilon_1)}. \tag{334}$$

In order for gradient descent to converge well, as shown in Corollary 1, we need

$$\left|\frac{\partial \mathcal{L}_{w^\star}}{\partial \beta_k}(\beta_k^{(t)}) - (\beta_k^{(t)} - \beta_k^\star)\right| \leq \epsilon_2 = \mathcal{O}\left(\frac{\epsilon_3}{\sqrt{D}\log\left(\sqrt{D}/\epsilon_3\right)}\right). \tag{335}$$

Thus, we must set R, ϵ_1 such that

$$\frac{20\pi^2 D^2 d R^2 \epsilon_1}{3} + \frac{4D}{\pi R(R_w/\sqrt{d} - D\epsilon_1)} \leq \epsilon_2 = \mathcal{O}\left(\frac{\epsilon_3}{\sqrt{D}\log\left(\sqrt{D}/\epsilon_3\right)}\right). \tag{336}$$

This can be satisfied by taking

$$R \geq \max\left(\frac{D^2}{\epsilon_2}, \frac{16D\sqrt{d}}{\pi R_w \epsilon_2}, D^2, \frac{16D\sqrt{d}}{\pi R_w}\right) = \max\left(\frac{D^2}{\epsilon_2}, \frac{16D\sqrt{d}}{\pi R_w \epsilon_2}\right). \tag{337}$$

$$\epsilon_1 \leq \min\left(\frac{3\epsilon_2^3}{40\pi^2 D^6 d}, \frac{3}{40\pi^2 D^6 d}, \frac{R_w}{2D\sqrt{d}}\right) = \min\left(\frac{3\epsilon_2^3}{40\pi^2 D^6 d}, \frac{R_w}{2D\sqrt{d}}\right). \tag{338}$$

Note that the last two terms in the maximum for R in Equation (337) and in the minimum for ϵ_1 in Equation (338) are from the constraints on R, ϵ_1 in Lemma 9. The equalities follow because $0 < \epsilon_2 < 1$. We can write this in terms of ϵ_3 by using upper bound of ϵ_2 in terms of ϵ_3 (Equation (327))

$$R \geq \max\left(\frac{2\eta D^{5/2}\log\left(2\sqrt{D}/\epsilon_3\right)}{\log(1/c)\epsilon_3}, \frac{32\eta D^{3/2}\sqrt{d}\log\left(2\sqrt{D}/\epsilon_3\right)}{\pi R_w \log(1/c)\epsilon_3}\right) = \tilde{\Omega}\left(\max\left(\frac{D^{5/2}}{\epsilon_3}, \frac{D^{3/2}\sqrt{d}}{R_w \epsilon_3}\right)\right). \tag{339}$$

$$\epsilon_1 \le \min\left(\frac{3\log^3(1/c)\epsilon_3^3}{80\pi^2\eta D^{13/2} d\log\left(2\sqrt{D}/\epsilon_3\right)}, \frac{R_w}{2D\sqrt{d}}\right) = \tilde{\mathcal{O}}\left(\min\left(\frac{\epsilon_3^3}{D^{13/2}d}, \frac{R_w}{D\sqrt{d}}\right)\right). \tag{340}$$

where $\eta = \mathcal{O}(1)$ is the step size of gradient descent and $c = 1-\eta$. We will prove that Equation (336) holds for the ϵ_2 dependence. Writing in terms of ϵ_3 follows simply from the upper bound of ϵ_2 in terms of ϵ_3 in Equation (327). We bound each term on the lefthand side of Equation (336) by $\epsilon_2/2$ to obtain the required bound.

For the first term, using that $R \ge D^2/\epsilon_2$ and $\epsilon_1 \le 3/(40\pi^2 R^3 d) \le 3\epsilon_2^3/(40\pi^2 D^6 d)$, we have

$$\frac{20\pi^2 D^2 d R^2 \epsilon_1}{3} \le \frac{D^2}{2R} \le \frac{\epsilon_2}{2}. \tag{341}$$

Finally, for the second term, using that $\epsilon_1 \le R_w/(2D\sqrt{d})$ and $R \ge 16D\sqrt{d}/(\pi R_w \epsilon_2)$, we have

$$\frac{4D}{\pi R(R_w/\sqrt{d} - D\epsilon_1)} \le \frac{8D\sqrt{d}}{\pi R R_w} \le \frac{\epsilon_2}{2}. \tag{342}$$

This completes the proof. □

With these choices of parameters, we can plug them in to determine the value of the loss function.

Lemma 10 (Loss bound)**.** *Let* $\epsilon_3 > 0$*. Let*

$$R = \tilde{\Omega}\left(\max\left(\frac{D^{5/2}}{\epsilon_3}, \frac{D^{3/2}\sqrt{d}}{R_w \epsilon_3}\right)\right), \quad \epsilon_1 = \tilde{\mathcal{O}}\left(\min\left(\frac{\epsilon_3^2}{D^{13/2}d}, \frac{R_w}{D\sqrt{d}}\right)\right). \tag{343}$$

as in Corollary 2. Let $w^\star \in \mathbb{R}^d$ *be unknown with norm* $R_w > 0$*. Let* $g_{w^\star} : \mathbb{R}^d \to [-1,1]$ *be defined as* $g_{w^\star}(x) = \tilde{g}(x^\intercal w^\star)$ *for* $\tilde{g}$ *given in Equation (10). Suppose we have an approximation* $\hat{w} \in \mathbb{R}^d$ *such that* $\|\hat{w} - w^\star\|_\infty \le \epsilon_1$*. Then, gradient descent can find a predictor* $\hat{\beta}$ *such that*

$$\mathcal{L}_{w^\star}(\hat{\beta}) \le \frac{\epsilon_3^2}{2} + \frac{13\sqrt{d}}{8\pi R_w R} + \frac{32\pi^2 D^2 d R^2 \epsilon_1}{3} + \frac{9D^2\sqrt{d}}{2\pi R(R_w - D\sqrt{d}\epsilon_1)}. \tag{344}$$

Proof. This proof will be somewhat similar to Lemma 7. First, let us expand the loss function:

$$\mathcal{L}_{w^\star}(\beta) = \int\limits_{x\sim\varphi^2} \left(\sum_{j=1}^D \beta_j^\star \cos(2\pi j x^\intercal w^\star) - \sum_{j=1}^D \beta_j \cos(2\pi j x^\intercal \hat{w})\right)^2 dx \tag{345}$$

$$\begin{aligned} &= \int\limits_{x\sim\varphi^2} \sum_{j,j'=1}^D \beta_j^\star \beta_{j'}^\star \cos(2\pi j x^\intercal w^\star)\cos(2\pi j' x^\intercal w^\star) + \beta_j\beta_{j'}\cos(2\pi j x^\intercal \hat{w})\cos(2\pi j' x^\intercal \hat{w}) \\ &\qquad - 2\beta_j^\star \beta_{j'} \cos(2\pi j x^\intercal w^\star)\cos(2\pi j' x^\intercal \hat{w})\, dx \end{aligned} \tag{346}$$

Separating out terms with $j \ne j'$, we have

$$\mathcal{L}_{w^\star}(\beta) = \sum_{j=1}^D \left((\beta_j^\star)^2 \int_{x\sim\varphi^2} \cos^2(2\pi j x^\intercal w^\star)\, dx + \beta_j^2 \int_{x\sim\varphi^2} \cos^2(2\pi j x^\intercal \hat{w})\, dx \right. \tag{347}$$

$$\left. -2\beta_j^\star \beta_j \int_{x\sim\varphi^2} \cos(2\pi j x^\intercal w^\star)\cos(2\pi j x^\intercal \hat{w})\, dx\right) \tag{348}$$

$$+ \sum_{\substack{j,j'=1 \\ j\ne j'}}^D \beta_j^\star \beta_{j'}^\star \int_{x\sim\varphi^2} \cos(2\pi j x^\intercal w^\star)\cos(2\pi j' x^\intercal w^\star)\, dx \tag{349}$$

$$+ \sum_{\substack{j,j'=1 \\ j\ne j'}}^D \beta_j \beta_{j'} \int_{x\sim\varphi^2} \cos(2\pi j x^\intercal \hat{w})\cos(2\pi j' x^\intercal \hat{w})\, dx \tag{350}$$

$$-2\sum_{\substack{j,j'=1\\j\neq j'}}^{D}\beta_j^\star\beta_{j'}\int_{x\sim\varphi^2}\cos(2\pi j x^\intercal w^\star)\cos(2\pi j' x^\intercal \hat{w})\,dx \tag{351}$$

We can upper bound the absolute values of the last three terms. For the term in Equation (349), by Corollary 6, we have

$$\left|\sum_{\substack{j,j'=1\\j\neq j'}}^{D}\beta_j^\star\beta_{j'}^\star\int_{x\sim\varphi^2}\cos(2\pi j x^\intercal w^\star)\cos(2\pi j' x^\intercal w^\star)\,dx\right| \tag{352}$$

$$\leq \sum_{\substack{j,j'=1\\j\neq j'}}^{D}|\beta_j^\star||\beta_{j'}^\star|\left|\int_{x\sim\varphi^2}\cos(2\pi j x^\intercal w^\star)\cos(2\pi j' x^\intercal w^\star)\,dx\right| \tag{353}$$

$$\leq \sum_{\substack{j,j'=1\\j\neq j'}}^{D}|\beta_j^\star||\beta_{j'}^\star|\frac{\sqrt{d}}{2\pi R R_w} \tag{354}$$

$$\leq \frac{\sqrt{d}}{2\pi R R_w}. \tag{355}$$

In the last line, we use that $\|\beta^\star\|_1 = 1$. Similarly, we can upper bound the absolute value of Equation (350):

$$\left|\sum_{\substack{j,j'=1\\j\neq j'}}^{D}\beta_j\beta_{j'}\int_{x\sim\varphi^2}\cos(2\pi j x^\intercal \hat{w})\cos(2\pi j' x^\intercal \hat{w})\,dx\right| \leq \sum_{\substack{j,j'=1\\j\neq j'}}^{D}|\beta_j||\beta_{j'}|\frac{\sqrt{d}}{2\pi R(R_w-\sqrt{d}\epsilon_1)} \tag{356}$$

$$\leq \frac{2D^2\sqrt{d}}{\pi R(R_w-\sqrt{d}\epsilon_1)}. \tag{357}$$

In the first inequality, we use Lemma 14, and in the second line we use Lemma 9 and our choice of R,ϵ_1. We can also upper bound the absolute value of Equation (351):

$$\left|2\sum_{\substack{j,j'=1\\j\neq j'}}^{D}\beta_j^\star\beta_{j'}\int_{x\sim\varphi^2}\cos(2\pi j x^\intercal w^\star)\cos(2\pi j' x^\intercal \hat{w})\,dx\right| \leq 2\sum_{\substack{j,j'=1\\j\neq j'}}^{D}|\beta_j^\star||\beta_{j'}|\frac{\sqrt{d}}{2\pi R(R_w-D\sqrt{d}\epsilon_1)} \tag{358}$$

$$\leq \frac{2D\sqrt{d}}{\pi R(R_w-D\sqrt{d}\epsilon_1)}. \tag{359}$$

In the first inequality, we use Corollary 8, and in the second line, we use Lemma 9 and our choice of R,ϵ_1 as well as $\|\beta^\star\|_1 = 1$. Combining Equations (355), (357) and (359), we have

$$\mathcal{L}_{w^\star}(\beta) \leq \sum_{j=1}^{D}\Bigg((\beta_j^\star)^2\int_{x\sim\varphi^2}\cos^2(2\pi j x^\intercal w^\star)\,dx + \beta_j^2\int_{x\sim\varphi^2}\cos^2(2\pi j x^\intercal \hat{w})\,dx \tag{360}$$

$$-2\beta_j^\star\beta_j\int_{x\sim\varphi^2}\cos(2\pi j x^\intercal w^\star)\cos(2\pi j x^\intercal \hat{w})\,dx\Bigg) \tag{361}$$

$$+\frac{\sqrt{d}}{2\pi R R_w}+\frac{2D^2\sqrt{d}}{\pi R(R_w-\sqrt{d}\epsilon_1)}+\frac{2D\sqrt{d}}{\pi R(R_w-D\sqrt{d}\epsilon_1)}. \tag{362}$$

It remains to bound the terms involving the integral of cosine. By the proof of Lemma 13 and Corollary 5, then

$$\mathcal{L}_{w^\star}(\beta) \leq \left(\frac{1}{2}+\frac{\sqrt{d}}{8\pi R_w R}\right)\|\beta^\star\|_2^2 + \left(\frac{1}{2}+\frac{\sqrt{d}}{8\pi R(R_w-\sqrt{d}\epsilon_1)}\right)\|\beta\|_2^2 \tag{363}$$

$$- 2\beta_j^\star \beta_j \int_{x\sim\varphi^2} \cos(2\pi j x^\intercal w^\star)\cos(2\pi j x^\intercal \hat{w})\,dx \tag{364}$$

$$+ \frac{\sqrt{d}}{2\pi R R_w} + \frac{2D^2\sqrt{d}}{\pi R(R_w - \sqrt{d}\epsilon_1)} + \frac{2D\sqrt{d}}{\pi R(R_w - D\sqrt{d}\epsilon_1)} \tag{365}$$

$$\leq \frac{1}{2}\|\beta^\star\|_2^2 + \frac{1}{2}\|\beta\|_2^2 - 2\beta_j^\star\beta_j \int_{x\sim\varphi^2} \cos(2\pi j x^\intercal w^\star)\cos(2\pi j x^\intercal \hat{w})\,dx \tag{366}$$

$$+ \frac{5\sqrt{d}}{8\pi R_w R} + \frac{(4D^2 + D)\sqrt{d}}{2\pi R(R_w - \sqrt{d}\epsilon_1)} + \frac{2D\sqrt{d}}{\pi R(R_w - D\sqrt{d}\epsilon_1)}. \tag{367}$$

In the inequality, we use that $\|\beta^\star\|_1 = 1$ and Lemma 9. For the last remaining integral term, we have the following

$$2\beta_j^\star\beta_j \int_{x\sim\varphi^2} \cos(2\pi j x^\intercal w^\star)\cos(2\pi j x^\intercal \hat{w})\,dx \tag{368}$$

$$= -2 \sum_{\substack{j=1 \\ \operatorname{sign}(\beta_j)=\operatorname{sign}(\beta_j^\star)}}^{D} \beta_j^\star\beta_j \int_{x\sim\varphi^2} \cos(2\pi j x^\intercal w^\star)\cos(2\pi j x^\intercal \hat{w})\,dx \tag{369}$$

$$- 2 \sum_{\substack{j=1 \\ \operatorname{sign}(\beta_j)\neq\operatorname{sign}(\beta_j^\star)}}^{D} \beta_j^\star\beta_j \int_{x\sim\varphi^2} \cos(2\pi j x^\intercal w^\star)\cos(2\pi j x^\intercal \hat{w})\,dx \tag{370}$$

$$\leq -2 \sum_{\substack{j=1 \\ \operatorname{sign}(\beta_j)=\operatorname{sign}(\beta_j^\star)}}^{D} \beta_j^\star\beta_j \left(\frac{1}{2} - \frac{\sqrt{d}}{8\pi R_w R} - \frac{5\pi^2 D^2 d R^2 \epsilon_1}{3}\right) \tag{371}$$

$$- 2 \sum_{\substack{j=1 \\ \operatorname{sign}(\beta_j)\neq\operatorname{sign}(\beta_j^\star)}}^{D} \beta_j^\star\beta_j \left(\frac{1}{2} + \frac{\sqrt{d}}{8\pi R_w R} + \pi D d \epsilon_1 R\right) \tag{372}$$

$$= -\sum_{j=1}^{D} \beta_j^\star\beta_j + \left(\frac{\sqrt{d}}{4\pi R_w R} + \frac{10\pi^2 D^2 d R^2 \epsilon_1}{3}\right) \sum_{\substack{j=1 \\ \operatorname{sign}(\beta_j)=\operatorname{sign}(\beta_j^\star)}}^{D} \beta_j^\star\beta_j \tag{373}$$

$$- \left(\frac{\sqrt{d}}{4\pi R_w R} + 2\pi D d \epsilon_1 R\right) \sum_{\substack{j=1 \\ \operatorname{sign}(\beta_j)\neq\operatorname{sign}(\beta_j^\star)}}^{D} \beta_j^\star\beta_j. \tag{374}$$

Here, in the second line, we split the sum depending on if the signs of the $\beta_j, \beta_j^\star$ match. In the fourth line, since $\operatorname{sign}(\beta_j) \neq \operatorname{sign}(\beta_j^\star)$, then $\beta_j\beta_j^\star \leq 0$ so that the last term has a positive coefficient overall. Thus, we can use an upper bound on the integral, where we use Lemma 13. Also, since $\operatorname{sign}(\beta_j) = \operatorname{sign}(\beta_j^\star)$, then $\beta_j\beta_j^\star \geq 0$ so that the first term has a negative coefficient overall. Thus, we can use a lower bound on the integral, where we use Lemma 12. In the last equality, we combined the summations over j again. Plugging this into the expression we had before, we have

$$\mathcal{L}_{w^\star}(\beta) \leq \frac{1}{2}\|\beta - \beta^\star\|_2^2 + \left(\frac{\sqrt{d}}{4\pi R_w R} + \frac{10\pi^2 D^2 d R^2 \epsilon_1}{3}\right) \sum_{\substack{j=1 \\ \operatorname{sign}(\beta_j)=\operatorname{sign}(\beta_j^\star)}}^{D} \beta_j^\star\beta_j \tag{375}$$

$$- \left(\frac{\sqrt{d}}{4\pi R_w R} + 2\pi D d \epsilon_1 R\right) \sum_{\substack{j=1 \\ \operatorname{sign}(\beta_j)\neq\operatorname{sign}(\beta_j^\star)}}^{D} \beta_j^\star\beta_j + \frac{5\sqrt{d}}{8\pi R_w R} + \frac{(4D^2 + D)\sqrt{d}}{2\pi R(R_w - \sqrt{d}\epsilon_1)} + \frac{2D\sqrt{d}}{\pi R(R_w - D\sqrt{d}\epsilon_1)} \tag{376}$$

We can further bound this by taking the absolute value to get

$$\mathcal{L}_{w^\star}(\beta) \leq \frac{1}{2}\|\beta - \beta^\star\|_2^2 + \left(\frac{\sqrt{d}}{4\pi R_w R} + \frac{10\pi^2 D^2 d R^2 \epsilon_1}{3}\right) \sum_{\substack{j=1 \\ \operatorname{sign}(\beta_j)=\operatorname{sign}(\beta_j^\star)}}^{D} |\beta_j^\star||\beta_j| \tag{377}$$

$$+\left(\frac{\sqrt{d}}{4\pi R_w R}+2\pi Dd\epsilon_1 R\right)\sum_{\substack{j=1\\ \mathrm{sign}(\beta_j)\neq\mathrm{sign}(\beta_j^\star)}}^{D}|\beta_j^\star||\beta_j|+\frac{5\sqrt{d}}{8\pi R_w R}+\frac{(4D^2+D)\sqrt{d}}{2\pi R(R_w-\sqrt{d}\epsilon_1)}+\frac{2D\sqrt{d}}{\pi R(R_w-D\sqrt{d}\epsilon_1)}. \tag{378}$$

Using Lemma 9 with our choice of R, ϵ_1 and $\|\beta^\star\|_1 = 1$, then we have

$$\mathcal{L}_{w^\star}(\beta) \le \frac{1}{2}\|\beta-\beta^\star\|_2^2+\frac{\sqrt{d}}{2\pi R_w R}+\frac{20\pi^2D^2dR^2\epsilon_1}{3}+\frac{\sqrt{d}}{2\pi R_w R}+4\pi Dd\epsilon_1 R \tag{379}$$

$$+\frac{5\sqrt{d}}{8\pi R_w R}+\frac{(4D^2+D)\sqrt{d}}{2\pi R(R_w-\sqrt{d}\epsilon_1)}+\frac{2D\sqrt{d}}{\pi R(R_w-D\sqrt{d}\epsilon_1)} \tag{380}$$

$$=\frac{1}{2}\|\beta-\beta^\star\|_2^2+\frac{13\sqrt{d}}{8\pi R_w R}+\frac{20\pi^2D^2dR^2\epsilon_1}{3}+4\pi Dd\epsilon_1 R+\frac{(4D^2+D)\sqrt{d}}{2\pi R(R_w-\sqrt{d}\epsilon_1)}+\frac{2D\sqrt{d}}{\pi R(R_w-D\sqrt{d}\epsilon_1)} \tag{381}$$

$$\le\frac{1}{2}\|\beta-\beta^\star\|_2^2+\frac{13\sqrt{d}}{8\pi R_w R}+\frac{32\pi^2D^2dR^2\epsilon_1}{3}+\frac{(4D^2+D)\sqrt{d}}{2\pi R(R_w-\sqrt{d}\epsilon_1)}+\frac{2D\sqrt{d}}{\pi R(R_w-D\sqrt{d}\epsilon_1)} \tag{382}$$

$$\le\frac{1}{2}\|\beta-\beta^\star\|_2^2+\frac{13\sqrt{d}}{8\pi R_w R}+\frac{32\pi^2D^2dR^2\epsilon_1}{3}+\frac{9D^2\sqrt{d}}{2\pi R(R_w-D\sqrt{d}\epsilon_1)}. \tag{383}$$

Here, in the first inequality, we use Lemma 9 and $\|\beta^\star\|_1 = 1$. In the second inequality, we use that $D \ge 1$ so that $D^2 \ge D$ and $R^2 \ge R$. In the last line, we use that $D\epsilon_1 \ge \epsilon_1$ and $D^2 \ge D$. The claim then follows from Lemma 8 and Corollary 2, which says that using gradient descent, after a sufficient number of steps, we reach $\hat{\beta} = \beta^{(t+1)}$ such that $\left\|\beta^{(t+1)} - \beta^\star\right\|_2 \le \epsilon_3$. □

Finally, we can choose ϵ_3 and adjust our choices for R, ϵ_1 to show that the loss function is indeed bounded by ϵ for our predictor $\hat{\beta}$ found via gradient descent.

Proof of Theorem 7. Let $\epsilon > 0$. By Lemma 10, taking $\epsilon_3 = \sqrt{\epsilon}$, we have that

$$\mathcal{L}_{w^\star}(\hat{\beta}) \le \frac{\epsilon}{2}+\frac{13\sqrt{d}}{8\pi R_w R}+\frac{32\pi^2D^2dR^2\epsilon_1}{3}+\frac{9D^2}{2\pi R(R_w/\sqrt{d}-D\epsilon_1)}. \tag{384}$$

for our choice of R, ϵ_1. Here, recall that ϵ_1 is the accuracy with which we can estimate $w^\star$, i.e., $|\hat{w}_i - w_i^\star| \le \epsilon_1$. We want to show that $\mathcal{L}_{w^\star}(\hat{\beta}) \le \epsilon$. This can be satisfied by taking

$$R \ge \max\left(\frac{39\sqrt{d}}{4\pi R_w\epsilon},\frac{D^2}{\epsilon},\frac{54D^2\sqrt{d}}{\pi R_w\epsilon},\frac{2\eta D^{5/2}\log\left(2\sqrt{D}/\sqrt{\epsilon}\right)}{\log(1/c)\sqrt{\epsilon}},\frac{32\eta D^{3/2}\sqrt{d}\log\left(2\sqrt{D}/\sqrt{\epsilon}\right)}{\pi R_w\log(1/c)\sqrt{\epsilon}}\right) \tag{385}$$

$$=\max\left(\frac{D^2}{\epsilon},\frac{54D^2\sqrt{d}}{\pi R_w\epsilon},\frac{2\eta D^{5/2}\log\left(2\sqrt{D}/\sqrt{\epsilon}\right)}{\log(1/c)\sqrt{\epsilon}},\frac{32\eta D^{3/2}\sqrt{d}\log\left(2\sqrt{D}/\sqrt{\epsilon}\right)}{\pi R_w\log(1/c)\sqrt{\epsilon}}\right) \tag{386}$$

$$\epsilon_1 \le \min\left(\frac{\epsilon^3}{64\pi^2D^6d},\frac{3\log^3(1/c)\epsilon^{3/2}}{80\pi^2\eta D^{13/2}d\log\left(2\sqrt{D}/\sqrt{\epsilon}\right)},\frac{R_w}{2D\sqrt{d}}\right), \tag{387}$$

where $\eta = \mathcal{O}(1)$ is the step size of gradient descent and $c = 1-\eta$. Note that the last two terms in the maximum for R come from Corollary 2 and similarly for the last two terms in the minimum for ϵ_1.

For the second term in Equation (384), since $R \ge 39\sqrt{d}/(4\pi R_w\epsilon)$, we have

$$\frac{13\sqrt{d}}{8\pi R_w R} \le \frac{\epsilon}{6}. \tag{388}$$

For the third term in Equation (384), using $R \ge D^2/\epsilon$ and $\epsilon_1 \le 1/(64\pi^2R^3d) \le \epsilon^3/(64\pi^2D^6d)$, then

$$\frac{32\pi^2D^2dR^2\epsilon_1}{3} \le \frac{D^2}{6R} \le \frac{\epsilon}{6}. \tag{389}$$

Finally, for the last term in Equation (384), using $\epsilon_1 \leq R_w/(2D\sqrt{d})$ and $R \geq 54D^2\sqrt{d}/(\pi R_w \epsilon)$, we have

$$\frac{9D^2}{2\pi R(R_w/\sqrt{d} - D\epsilon_1)} \leq \frac{9D^2\sqrt{d}}{\pi R R_w} \leq \frac{\epsilon}{6}. \tag{390}$$

Thus, we have shown that

$$\mathcal{L}_{w^\star}(\hat{\beta}) \leq \frac{\epsilon}{2} + \frac{\epsilon}{6} + \frac{\epsilon}{6} + \frac{\epsilon}{6} = \epsilon, \tag{391}$$

proving the claim. Moreover, we have the following simplified scaling of R, ϵ_1 by hiding the constants and logarithmic factors:

$$R = \tilde{\Omega}\left(\max\left(\frac{D^2}{\epsilon}, \frac{D^2\sqrt{d}}{R_w\epsilon}, \frac{D^{5/2}}{\sqrt{\epsilon}}, \frac{D^{3/2}\sqrt{d}}{R_w\sqrt{\epsilon}}\right)\right), \tag{392}$$

$$\epsilon_1 = \tilde{\mathcal{O}}\left(\min\left(\frac{\epsilon^3}{D^6 d}, \frac{\epsilon^{3/2}}{D^{13/2}d}, \frac{R_w}{D\sqrt{d}}\right)\right). \tag{393}$$

The bound on the number of iterations of gradient descent used simply comes from

$$t = \Theta\left(\log\left(\sqrt{D}/\epsilon_3\right)\right) \tag{394}$$

from Corollary 1 and the choice $\epsilon_3 = \sqrt{\epsilon}$. □

C. Integral bounds

The following technical lemmas for bounding integrals will be useful in the proofs of Theorem 6 and Theorem 7.

First, we have a bound on a complex exponential that will be useful in several of the other lemmas in this section.

Lemma 11. *Let φ^2 be the uniform density over $[-R, R]^d \subseteq \mathbb{R}^d$. Let $w^\star \in \mathbb{R}^d$ be unknown with norm $R_w > 0$, and let $\hat{w} \in \mathbb{R}^d$ be an approximation of $w^\star$ with $\|\hat{w} - w^\star\|_\infty \leq \epsilon_1$. Let $1 \leq j, j' \leq D$ be integers with $j \neq j'$, for $D \in \mathbb{N}$ from Equation (10). Then,*

$$\left|\int_{x\sim\varphi^2} e^{2\pi i x^\intercal \hat{w}(j-j')}\,dx\right| \leq \frac{1}{2\pi R}\frac{\sqrt{d}}{R_w - \sqrt{d}\epsilon_1}. \tag{395}$$

Proof. Using that φ^2 is the uniform density:

$$\left|\int_{x\sim\varphi^2} e^{2\pi i x^\intercal \hat{w}(j-j')}\,dx\right| = \left|\frac{1}{(2R)^d}\int_{x_1=-R}^{+R}\cdots\int_{x_d=-R}^{+R} e^{2\pi i\sum_{k=1}^d x_k\hat{w}_k(j-j')}\,dx_d\cdots dx_1\right| \tag{396}$$

$$= \left|\frac{1}{(2R)^d}\prod_{k=1}^d\int_{x_k=-R}^{+R} e^{2\pi i x_k\hat{w}_k(j-j')}\,dx_k\right|. \tag{397}$$

Here, notice that we can bound each of these integrals by $2R$:

$$\left|\int_{x_k=-R}^{+R} e^{2\pi i x_k\hat{w}_k(j-j')}\,dx_k\right| \leq \int_{x_k=-R}^{+R}\left|e^{2\pi i x_k\hat{w}_k(j-j')}\right|dx_k \leq \int_{x_k=-R}^{+R} dx_k = 2R. \tag{398}$$

We also notice that because $\|w^\star\|_2^2 = \sum_{i=1}^d |w_i^\star|^2 = R_w^2$, then there must exist some $k \in [d]$ such that $|w_k^\star| \geq R_w/\sqrt{d}$. Here, equality is satisfied for the case when $w_i = R_w/\sqrt{d}$ for all $i \in [d]$. We will bound each integral in the product in Equation (397) using Equation (398) except for this k such that $|w_k^\star| \geq R_w/\sqrt{d}$:

$$\left|\int_{x\sim\varphi^2} e^{2\pi i x^\intercal \hat{w}(j-j')}\,dx\right| = \left|\frac{1}{(2R)^d}\prod_{k=1}^d\int_{x_k=-R}^{+R} e^{2\pi i x_k\hat{w}_k(j-j')}\,dx_k\right| \tag{399}$$

$$\leq \frac{1}{2R}\left|\int_{x_k=-R}^{+R} e^{2\pi i x_k \hat{w}_k (j-j')}\,dx_k\right| \tag{400}$$

$$= \frac{1}{2R}\left|\int_{x_k=-R}^{+R} \cos(2\pi x_k \hat{w}_k (j-j'))\,dx_k\right| \tag{401}$$

$$= \frac{1}{2R}\left|\frac{\sin(2\pi(j-j')R\hat{w}_k)}{\pi(j-j')\hat{w}_k}\right| \tag{402}$$

$$\leq \frac{1}{2R}\frac{1}{\pi|j-j'||\hat{w}_k|} \tag{403}$$

$$\leq \frac{1}{2R}\frac{1}{\pi|\hat{w}_k|}. \tag{404}$$

Here, in the second line, we use Equation (398). In the third line, because we are integrating over a symmetric interval, the sine contribution vanishes. In the fifth line, we use that $|\sin(x)| \leq 1$, and in the last line we used that $j \neq j'$ so that $|j - j'| \geq 1$. Now, because we chose k such that $|w_k^\star| \geq R_w/\sqrt{d}$ and $|\hat{w}_i - w_i^\star| \leq \epsilon_1$ for all i, we have

$$\epsilon_1 \geq |\hat{w}_k - w_k^\star| \geq ||\hat{w}_k| - |w_k^\star|| \geq \left||\hat{w}_k| - \frac{R_w}{\sqrt{d}}\right| \tag{405}$$

so that rearranging, we have

$$|\hat{w}_k| \geq \frac{R_w}{\sqrt{d}} - \epsilon_1. \tag{406}$$

Plugging this back into the above, we have

$$\left|\int_{x\sim\varphi^2} e^{2\pi i x^\intercal \hat{w}(j-j')}\,dx\right| \leq \frac{1}{2\pi R}\frac{\sqrt{d}}{R_w - \sqrt{d}\epsilon_1}. \tag{407}$$

□

Corollary 3. *Let φ^2 be the uniform density over $[-R, R]^d \subseteq \mathbb{R}^d$. Let $w^\star \in \mathbb{R}^d$ be unknown with norm $R_w > 0$, and let $\hat{w} \in \mathbb{R}^d$ be an approximation of $w^\star$ with $\|\hat{w} - w^\star\|_\infty \leq \epsilon_1$. Let $1 \leq j, j' \leq D$ be integers with $j \neq j'$, for $D \in \mathbb{N}$ from Equation (10). Then,*

$$\left|\int_{x\sim\varphi^2} e^{2\pi i x^\intercal w^\star (j-j')}\,dx\right| \leq \frac{1}{2\pi R}\frac{\sqrt{d}}{R_w}. \tag{408}$$

Proof. This is true by the same proof as Lemma 14. Because this is for $w^\star$ instead of $\hat{w}$, we no longer have the ϵ_1 term. □

Now, we can use this to obtain a lower bound for an integral of a product of cosines.

Lemma 12. *Let φ^2 be the uniform density over $[-R, R]^d \subseteq \mathbb{R}^d$. Let $w^\star \in \mathbb{R}^d$ be unknown with norm $R_w > 0$, and let $\hat{w} \in \mathbb{R}^d$ be an approximation of $w^\star$ with $\|\hat{w} - w^\star\|_\infty \leq \epsilon_1$. Let $1 \leq j \leq D$ be an integer, for $D \in \mathbb{N}$ from Equation (10). Then,*

$$\int_{x\sim\varphi^2} \cos(2\pi j x^\intercal \hat{w})\cos(2\pi j x^\intercal w^\star)\,dx \geq \frac{1}{2} - \frac{\sqrt{d}}{8\pi R_w R} - \frac{5\pi^2 D^2 d R^2 \epsilon_1}{3}. \tag{409}$$

Proof. Using the sum formulas for cosine, we have

$$\int_{x\sim\varphi^2} \cos(2\pi j x^\intercal \hat{w})\cos(2\pi j x^\intercal w^\star)\,dx \tag{410}$$

$$= \int_{x\sim\varphi^2} \cos(2\pi j x^\intercal (w^\star + (\hat{w} - w^\star)))\cos(2\pi j x^\intercal w^\star)\,dx \tag{411}$$

$$= \int_{x\sim\varphi^2} (\cos(2\pi j x^\intercal w^\star)\cos(2\pi j x^\intercal(\hat{w} - w^\star)) - \sin(2\pi j x^\intercal w^\star)\sin(2\pi j x^\intercal(\hat{w} - w^\star)))\cos(2\pi j x^\intercal w^\star)\,dx \tag{412}$$

$$\geq \int_{x\sim\varphi^2} \cos^2(2\pi j x^\intercal w^\star)\left(1-\frac{1}{2}(2\pi j x^\intercal(\hat{w}-w^\star))^2\right) - \sin(2\pi j x^\intercal w^\star)\sin(2\pi j x^\intercal(\hat{w}-w^\star))\cos(2\pi j x^\intercal w^\star)\,dx \tag{413}$$

$$\geq \int_{x\sim\varphi^2} \cos^2(2\pi j x^\intercal w^\star)\,dx - 2\pi^2 j^2 \int_{x\sim\varphi^2} (x^\intercal(\hat{w}-w^\star))^2\,dx - 2\pi j \int_{x\sim\varphi^2} |x^\intercal(\hat{w}-w^\star)|\,dx. \tag{414}$$

In the third line, we use the sum formula for cosines. In the fourth line, we use that $\cos(y) \geq 1 - y^2/2$. In the fifth line, we use that $\sin(y), \cos(y) \leq 1$ and $\sin(y) \leq |y|$. We want to lower bound the first term and upper bound the second two.

First, we will lower bound the first term in Equation (414). We can expand the first term in terms of complex exponentials:

$$\int_{x\sim\varphi^2} \cos^2(2\pi j x^\intercal w^\star)\,dx = \frac{1}{4}\int_{x\sim\varphi^2}\left(e^{2\pi i j x^\intercal w^\star} + e^{-2\pi i j x^\intercal w^\star}\right)^2 dx \tag{415}$$

$$= \frac{1}{2} + \frac{1}{4}\int_{x\sim\varphi^2} e^{4\pi i j x^\intercal w^\star}\,dx + \frac{1}{4}\int_{x\sim\varphi^2} e^{-4\pi i j x^\intercal w^\star}\,dx. \tag{416}$$

Now, we can bound the absolute value of these complex exponentials via Corollary 3. Note that Corollary 3 applies because we only needed to use that $j \neq j'$ to lower bound $|j - j'| \geq 1$. This already clearly holds for $j \geq 1$. Thus, we have

$$\left|\int_{x\sim\varphi^2} \cos^2(2\pi j x^\intercal w^\star)\,dx - \frac{1}{2}\right| \leq \frac{1}{2}\left|\int_{x\sim\varphi^2} e^{4\pi i j x^\intercal w^\star}\,dx\right| \leq \frac{1}{8\pi R}\frac{\sqrt{d}}{R_w}. \tag{417}$$

Rearranging, we have

$$\int_{x\sim\varphi^2} \cos^2(2\pi j x^\intercal w^\star)\,dx \geq \frac{1}{2} - \frac{\sqrt{d}}{8\pi R_w R}. \tag{418}$$

This gives a lower bound on the first term in Equation (414). We still need to upper bound the other terms in Equation (414). For the second term, we can first directly evaluate the integral.

$$\int_{x\sim\varphi^2} (x^\intercal(\hat{w}-w^\star))^2\,dx \tag{419}$$

$$= \frac{1}{(2R)^d}\int_{x_1=-R}^{+R}\cdots\int_{x_d=-R}^{+R}\left(\sum_{i=1}^d x_i\hat{w}_i - x_i w_i^\star\right)^2 dx_d\cdots dx_1 \tag{420}$$

$$= \frac{1}{(2R)^d}\int_{x_1=-R}^{+R}\cdots\int_{x_d=-R}^{+R}\left(\sum_{i,i'=1}^d x_i x_{i'}\hat{w}_i\hat{w}_{i'} + x_i x_{i'} w_i^\star w_{i'}^\star - x_i x_{i'}\hat{w}_i w_{i'}^\star - x_i x_{i'} w_i^\star \hat{w}_{i'}\right) dx_d\cdots dx_1. \tag{421}$$

Here, notice that

$$\frac{1}{(2R)^d}\int_{x_1=-R}^{+R}\cdots\int_{x_d=-R}^{+R} x_i x_{i'}\,dx_d\cdots dx_1 = \frac{1}{(2R)^2}\int_{x_i=-R}^{+R}\int_{x_{i'}=-R}^{+R} x_i x_{i'}\,dx_{i'}\,dx_i \tag{422}$$

$$= \frac{\delta_{ii'}}{2R}\int_{x=-R}^{+R} x^2\,dx \tag{423}$$

$$= \frac{R^2}{3}\delta_{ii'}, \tag{424}$$

where the second line follows because if $i \neq i'$, we are integrating an odd function $x_{i'}$ over a symmetric interval. Plugging this into our previous expression, we have

$$\int_{x\sim\varphi^2} (x^\intercal(\hat{w}-w^\star))^2\,dx = \frac{R^2}{3}\sum_{i=1}^d (\hat{w}_i)^2 + (w_i^\star)^2 - 2\hat{w}_i w_i^\star \tag{425}$$

$$= \frac{R^2}{3}\|\hat{w} - w^\star\|_2^2 \tag{426}$$

$$\leq \frac{R^2}{3} d\epsilon_1^2. \tag{427}$$

In the last line, we used that $|\hat{w}_i - w_i^\star| \leq \epsilon_1$ for all $i \in [d]$.

Finally, we can similarly upper bound the last term in Equation (414).

$$\int_{x\sim\varphi^2} |x^\intercal(\hat{w} - w^\star)|\, dx = \frac{1}{(2R)^d} \int_{x_1=-R}^{+R} \cdots \int_{x_d=-R}^{+R} \left| \sum_{i=1}^{d} x_i(\hat{w}_i - w_i^\star) \right| dx_d \cdots dx_1 \tag{428}$$

$$\leq \frac{1}{(2R)^d} \int_{x_1=-R}^{+R} \cdots \int_{x_d=-R}^{+R} \sum_{i=1}^{d} |x_i(\hat{w}_i - w_i^\star)|\, dx_d \cdots dx_1 \tag{429}$$

$$= \frac{1}{2R} \left(\sum_{i=1}^{d} |\hat{w}_i - w_i^\star| \int_{x_i=-R}^{+R} |x_i|\, dx_i \right) \tag{430}$$

$$\leq \frac{\epsilon_1}{2R} \sum_{i=1}^{d} \int_{x_i=-R}^{+R} |x_i|\, dx_i \tag{431}$$

$$= \frac{\epsilon_1 d}{2R} R^2 \tag{432}$$

$$= \frac{\epsilon_1 d R}{2}. \tag{433}$$

In the second line, we use the triangle inequality. In the fourth line, we use that $|\hat{w}_i - w_i^\star| \leq \epsilon_1$ for all $i \in [d]$. Now, combining Equations (418), (427) and (433) in Equation (414), we have

$$\int\limits_{x\sim\varphi^2} \cos(2\pi j x^\intercal \hat{w}) \cos(2\pi j x^\intercal w^\star)\, dx \geq \frac{1}{2} - \frac{\sqrt{d}}{8\pi R_w R} - \frac{2\pi^2 j^2 R^2 d\epsilon_1^2}{3} - \pi j \epsilon_1 d R \tag{434}$$

$$\geq \frac{1}{2} - \frac{\sqrt{d}}{8\pi R_w R} - \frac{2\pi^2 j^2 R^2 d\epsilon_1}{3} - \pi^2 j^2 \epsilon_1 d R^2 \tag{435}$$

$$\geq \frac{1}{2} - \frac{\sqrt{d}}{8\pi R_w R} - \frac{5\pi^2 D^2 R^2 d\epsilon_1}{3}, \tag{436}$$

where in the second line, we use that $j, R \geq 1$ so that $j^2 \geq j$ and $R^2 \geq R$ and $\epsilon_1 < 1$ so that $\epsilon_1^2 \leq \epsilon_1$. In the last line, we use that $j \leq D$. □

Corollary 4. *Let φ^2 be the uniform density over $[-R, R]^d \subseteq \mathbb{R}^d$. Let $w^\star \in \mathbb{R}^d$ be unknown with norm $R_w > 0$, and let $\hat{w} \in \mathbb{R}^d$ be an approximation of $w^\star$ with $\|\hat{w} - w^\star\|_\infty \leq \epsilon_1$. Let $1 \leq j \leq D$ be an integer, for $D \in \mathbb{N}$ from Equation (10). Then,*

$$\int_{x\sim\varphi^2} \cos^2(2\pi j x^\intercal \hat{w})\, dx \geq \frac{1}{2} - \frac{\sqrt{d}}{8\pi R(R_w - \sqrt{d}\epsilon_1)}. \tag{437}$$

Proof. The proof follows from the lower bound of the first term in Equation (414) in the proof of Lemma 12. We can expand the first term in terms of complex exponentials:

$$\int_{x\sim\varphi^2} \cos^2(2\pi j x^\intercal \hat{w})\, dx = \frac{1}{4} \int_{x\sim\varphi^2} \left(e^{2\pi i j x^\intercal \hat{w}} + e^{-2\pi i j x^\intercal \hat{w}} \right)^2 dx \tag{438}$$

$$= \frac{1}{2} + \frac{1}{4} \int_{x\sim\varphi^2} e^{4\pi i j x^\intercal \hat{w}}\, dx + \frac{1}{4} \int_{x\sim\varphi^2} e^{-4\pi i j x^\intercal \hat{w}}\, dx. \tag{439}$$

Now, we can bound the absolute value of these complex exponentials via Lemma 11 (instead of Corollary 3). Note that Lemma 11 applies because we only needed to use that $j \neq j'$ to lower bound $|j - j'| \geq 1$. This already clearly holds for $j \geq 1$. Thus, we have

$$\left| \int_{x\sim\varphi^2} \cos^2(2\pi j x^\intercal \hat{w})\, dx - \frac{1}{2} \right| \leq \frac{1}{2} \left| \int_{x\sim\varphi^2} e^{4\pi i j x^\intercal \hat{w}}\, dx \right| \leq \frac{1}{8\pi R} \frac{\sqrt{d}}{R_w - \sqrt{d}\epsilon_1}. \tag{440}$$

Rearranging, we have

$$\int_{x\sim\varphi^2} \cos^2(2\pi j x^\intercal \hat{w})\, dx \geq \frac{1}{2} - \frac{\sqrt{d}}{8\pi R(R_w - \sqrt{d}\epsilon_1)}. \tag{441}$$

□

Lemma 13. *Let φ^2 be the uniform density over $[-R, R]^d \subseteq \mathbb{R}^d$. Let $w^\star \in \mathbb{R}^d$ be unknown with norm $R_w > 0$, and let $\hat{w} \in \mathbb{R}^d$ be an approximation of $w^\star$ with $\|\hat{w} - w^\star\|_\infty \leq \epsilon_1$. Let $1 \leq j \leq D$ be an integer, for $D \in \mathbb{N}$ from Equation (10). Then,*

$$\int\limits_{x\sim\varphi^2} \cos(2\pi j x^\intercal \hat{w}) \cos(2\pi j x^\intercal w^\star)\, dx \leq \frac{1}{2} + \frac{\sqrt{d}}{8\pi R_w R} + \pi D d \epsilon_1 R. \tag{442}$$

Proof. The proof of this is similar to that of Lemma 12. Using the sum formulas for cosine, we have

$$\int_{x\sim\varphi^2} \cos(2\pi j x^\intercal \hat{w}) \cos(2\pi j x^\intercal w^\star)\, dx \tag{443}$$
$$= \int_{x\sim\varphi^2} \cos(2\pi j x^\intercal (w^\star + (\hat{w} - w^\star))) \cos(2\pi j x^\intercal w^\star)\, dx \tag{444}$$
$$= \int_{x\sim\varphi^2} (\cos(2\pi j x^\intercal w^\star) \cos(2\pi j x^\intercal (\hat{w} - w^\star)) - \sin(2\pi j x^\intercal w^\star) \sin(2\pi j x^\intercal (\hat{w} - w^\star))) \cos(2\pi j x^\intercal w^\star)\, dx \tag{445}$$
$$\leq \int_{x\sim\varphi^2} \cos^2(2\pi j x^\intercal w^\star) - \sin(2\pi j x^\intercal w^\star) \sin(2\pi j x^\intercal (\hat{w} - w^\star)) \cos(2\pi j x^\intercal w^\star)\, dx \tag{446}$$
$$\leq \int_{x\sim\varphi^2} \cos^2(2\pi j x^\intercal w^\star) + \sin(2\pi j x^\intercal (\hat{w} - w^\star))\, dx \tag{447}$$
$$\leq \int_{x\sim\varphi^2} \cos^2(2\pi j x^\intercal w^\star)\, dx + 2\pi j \int_{x\sim\varphi^2} |x^\intercal (\hat{w} - w^\star)|\, dx. \tag{448}$$

In the fourth line, we use that $\cos(y) \leq 1$. In the fifth line, we use that $-\sin(y)\cos(y) \leq 1$. In the last line, we use that $\sin(y) \leq |y|$. We want to upper bound both of these terms, which is simple given the proof of Lemma 12.

Namely, in Equation (417), we showed that

$$\left| \int_{x\sim\varphi^2} \cos^2(2\pi j x^\intercal w^\star)\, dx - \frac{1}{2} \right| \leq \frac{1}{8\pi R} \frac{\sqrt{d}}{R_w}. \tag{449}$$

Thus, we can upper bound

$$\int_{x\sim\varphi^2} \cos^2(2\pi j x^\intercal w^\star)\, dx \leq \frac{1}{2} + \frac{\sqrt{d}}{8\pi R_w R} \tag{450}$$

Note that we have already upper bounded the third term in Equation (433):

$$2\pi j \int_{x\sim\varphi^2} |x^\intercal (\hat{w} - w^\star)|\, dx \leq \pi j d \epsilon_1 R \leq \pi D d \epsilon_1 R. \tag{451}$$

Combining Equation (450) and Equation (451) in Equation (448), we have

$$\int_{x\sim\varphi^2} \cos(2\pi j x^\intercal \hat{w}) \cos(2\pi j x^\intercal w^\star)\, dx \leq \frac{1}{2} + \frac{\sqrt{d}}{8\pi R_w R} + \pi D d \epsilon_1 R. \tag{452}$$

□

Corollary 5. *Let φ^2 be the uniform density over $[-R, R]^d \subseteq \mathbb{R}^d$. Let $w^\star \in \mathbb{R}^d$ be unknown with norm $R_w > 0$, and let $\hat{w} \in \mathbb{R}^d$ be an approximation of $w^\star$ with $\|\hat{w} - w^\star\|_\infty \leq \epsilon_1$. Let $1 \leq j \leq D$ be an integer, for $D \in \mathbb{N}$ from Equation (10). Then,*

$$\int\limits_{x\sim\varphi^2} \cos^2(2\pi j x^\intercal \hat{w})\, dx \leq \frac{1}{2} + \frac{\sqrt{d}}{8\pi R(R_w - \sqrt{d}\epsilon_1)}. \tag{453}$$

Proof. This follows directly from Equation (440). □

Lemma 14. *Let φ^2 be the uniform density over $[-R,R]^d \subseteq \mathbb{R}^d$. Let $w^\star \in \mathbb{R}^d$ be unknown with norm $R_w > 0$, and let $\hat{w} \in \mathbb{R}^d$ be an approximation of $w^\star$ with $\|\hat{w} - w^\star\|_\infty \leq \epsilon_1$. Let $1 \leq j, j' \leq D$ be integers with $j \neq j'$, for $D \in \mathbb{N}$ from Equation (10). Then,*

$$\left| \int\limits_{x\sim\varphi^2} \cos(2\pi j x^\intercal \hat{w}) \cos(2\pi j' x^\intercal \hat{w})\, dx \right| \leq \frac{1}{2\pi R} \frac{\sqrt{d}}{R_w - \sqrt{d}\epsilon_1}. \tag{454}$$

Proof. Using the product formulas for cosine, we can write the integral as

$$\left| \int\limits_{x\sim\varphi^2} \cos(2\pi j x^\intercal \hat{w}) \cos(2\pi j' x^\intercal \hat{w})\, dx \right| = \left| \frac{1}{2} \int_{x\sim\varphi^2} \cos(2\pi x^\intercal \hat{w}(j-j')) + \cos(2\pi x^\intercal \hat{w}(j+j'))\, dx \right|. \tag{455}$$

We can bound each of the integrals on the right hand side similarly. Starting with the first term, we can write it in terms of complex exponentials

$$\left| \int_{x\sim\varphi^2} \cos(2\pi x^\intercal \hat{w}(j-j'))\, dx \right| \leq \frac{1}{2} \left| \int_{x\sim\varphi^2} e^{2\pi i x^\intercal \hat{w}(j-j')}\, dx \right| + \frac{1}{2} \left| \int_{x\sim\varphi^2} e^{2\pi i x^\intercal \hat{w}(j'-j)}\, dx \right| \tag{456}$$

Both terms in Equation (49) can be bounded via Lemma 11. Thus, this bounds the first term in Equation (455) as

$$\left| \int_{x\sim\varphi^2} \cos(2\pi x^\intercal \hat{w}(j-j'))\, dx \right| \leq \frac{1}{2\pi R} \frac{\sqrt{d}}{R_w - \sqrt{d}\epsilon_1}. \tag{457}$$

We can similarly bound the second term in Equation (455). Namely, the argument is the same as in Lemma 11, but in Equation (403), we have

$$\left| \int_{x\sim\varphi^2} e^{2\pi i x^\intercal \hat{w}(j+j')}\, dx \right| \leq \frac{1}{2R} \frac{1}{\pi |j+j'| |\hat{w}_k|} \tag{458}$$

$$\leq \frac{1}{6R} \frac{1}{\pi |\hat{w}_k|}, \tag{459}$$

where since $j \neq j'$ and $j, j' \geq 1$, then $|j+j'| \geq 3$. The rest of the bound follows the same argument. Then, we obtain

$$\left| \int_{x\sim\varphi^2} \cos(2\pi x^\intercal \hat{w}(j+j'))\, dx \right| \leq \frac{1}{6\pi R} \frac{\sqrt{d}}{R_w - \sqrt{d}\epsilon_1} \leq \frac{1}{2\pi R} \frac{\sqrt{d}}{R_w - \sqrt{d}\epsilon_1}. \tag{460}$$

Thus, combined with Equation (457) in Equation (455), we have

$$\left| \int\limits_{x\sim\varphi^2} \cos(2\pi j x^\intercal \hat{w}) \cos(2\pi j' x^\intercal \hat{w})\, dx \right| \leq \frac{1}{2\pi R} \frac{\sqrt{d}}{R_w - \sqrt{d}\epsilon_1}. \tag{461}$$

□

By essentially the same proof, we can obtain a similar upper bound replacing $\hat{w}$ with $w^\star$. This follows by applying Corollary 3 instead of Lemma 11.

Corollary 6. *Let φ^2 be the uniform density over $[-R,R]^d \subseteq \mathbb{R}^d$. Let $w^\star \in \mathbb{R}^d$ be unknown with norm $R_w > 0$, and let $\hat{w} \in \mathbb{R}^d$ be an approximation of $w^\star$ with $\|\hat{w} - w^\star\|_\infty \leq \epsilon_1$. Let $1 \leq j, j' \leq D$ be integers with $j \neq j'$, for $D \in \mathbb{N}$ from Equation (10). Then,*

$$\left| \int\limits_{x\sim\varphi^2} \cos(2\pi j x^\intercal w^\star) \cos(2\pi j' x^\intercal w^\star)\, dx \right| \leq \frac{1}{2\pi R} \frac{\sqrt{d}}{R_w}. \tag{462}$$

Corollary 7. *Let φ^2 be the uniform density over $[-R,R]^d \subseteq \mathbb{R}^d$. Let $w^\star \in \mathbb{R}^d$ be unknown with norm $R_w > 0$, and let $\hat{w} \in \mathbb{R}^d$ be an approximation of $w^\star$ with $\|\hat{w} - w^\star\|_\infty \le \epsilon_1$. Let $1 \le j, j' \le D$ be integers with $j \ne j'$, for $D \in \mathbb{N}$ from Equation (10). Then,*

$$\left| \int\limits_{x\sim\varphi^2} \cos(2\pi j x^\intercal w^\star)\sin(2\pi j' x^\intercal w^\star)\, dx \right| \le \frac{1}{2\pi R}\frac{\sqrt{d}}{R_w}. \tag{463}$$

Proof. This follows by the same proof as Lemma 14 and Corollary 6. In particular, using the sum-product formulas for sine and cosine, we have

$$\left| \int_{x\sim\varphi^2} \cos(2\pi j x^\intercal w^\star)\sin(2\pi j' x^\intercal w^\star)\, dx \right| = \left| \frac{1}{2}\int_{x\sim\varphi^2} \sin(2\pi(j+j')x^\intercal w^\star) + \sin(2\pi(j'-j)x^\intercal w^\star)\, dx \right|. \tag{464}$$

Then, writing in terms of complex exponentials, we have

$$\left| \int_{x\sim\varphi^2} \sin(2\pi(j'-j)x^\intercal w^\star)\, dx \right| \le \frac{1}{|2i|}\left| \int_{x\sim\varphi^2} e^{2\pi i x^\intercal w^\star (j'-j)}\, dx \right| + \frac{1}{|2i|}\left| \int_{x\sim\varphi^2} e^{2\pi i x^\intercal w^\star (j-j')}\, dx \right|. \tag{465}$$

The rest of the proof is the same as Lemma 14, using Corollary 3 instead of Lemma 11 to bound the complex exponential terms. □

Finally, we need another integral bound similar to Lemma 14.

Corollary 8. *Let φ^2 be the uniform density over $[-R,R]^d \subseteq \mathbb{R}^d$. Let $w^\star \in \mathbb{R}^d$ be unknown with norm $R_w > 0$, and let $\hat{w} \in \mathbb{R}^d$ be an approximation of $w^\star$ with $\|\hat{w} - w^\star\|_\infty \le \epsilon_1$, where $\epsilon_1 \le R_w/(D\sqrt{d})$. Let $1 \le j, j' \le D$ be integers with $j \ne j'$, for $D \in \mathbb{N}$ from Equation (10). Then,*

$$\left| \int\limits_{x\sim\varphi^2} \cos(2\pi j x^\intercal w^\star)\cos(2\pi j' x^\intercal \hat{w})\, dx \right| \le \frac{1}{2\pi R}\frac{\sqrt{d}}{R_w - D\sqrt{d}\epsilon_1}. \tag{466}$$

Proof. The proof is similar to that of Lemma 14, but we write it out fully to keep track of the differences. Using the product formulas for cosine, we can write the integral as

$$\left| \int\limits_{x\sim\varphi^2} \cos(2\pi j x^\intercal w^\star)\cos(2\pi j' x^\intercal \hat{w})\, dx \right| = \left| \frac{1}{2}\int_{x\sim\varphi^2} \cos(2\pi(j x^\intercal w^\star - j' x^\intercal \hat{w})) + \cos(2\pi(j x^\intercal w^\star + j' x^\intercal \hat{w}))\, dx \right|. \tag{467}$$

We can bound each of the integrals on the right hand side similarly. Starting with the first term, we can write it in terms of complex exponentials

$$\left| \int_{x\sim\varphi^2} \cos(2\pi(j x^\intercal w^\star - j' x^\intercal \hat{w}))\, dx \right| \le \frac{1}{2}\left| \int_{x\sim\varphi^2} e^{2\pi i (j x^\intercal w^\star - j' x^\intercal \hat{w})}\, dx \right| + \frac{1}{2}\left| \int_{x\sim\varphi^2} e^{2\pi i (j' x^\intercal \hat{w} - j x^\intercal w^\star)}\, dx \right| \tag{468}$$

Each of these complex exponentials can be bounded by an argument similar to Lemma 11. Using that φ^2 is the uniform density:

$$\left| \int_{x\sim\varphi^2} e^{2\pi i (j' x^\intercal \hat{w} - j x^\intercal w^\star)}\, dx \right| = \left| \frac{1}{(2R)^d} \prod_{k=1}^{d} \int_{x_k=-R}^{+R} e^{2\pi i (j' x_k \hat{w}_k - j x_k w_k^\star)}\, dx_k \right|. \tag{469}$$

Again, we can bound each of these integrals by $2R$ as in Equation (398). Notice that because $\|w^\star\|_2^2 = \sum_{i=1}^d |w_i^\star|^2 = R_w^2$, then there must exist some $k \in [d]$ such that $|w_k^\star| \ge \sqrt{R_w/d}$. Then, we will bound each integral in the product above using Equation (398) except for this k such that $|w_k^\star| \ge R_w/\sqrt{d}$:

$$\left| \int_{x\sim\varphi^2} e^{2\pi i (j' x^\intercal \hat{w} - j x^\intercal w^\star)}\, dx \right| = \left| \frac{1}{(2R)^d} \prod_{k=1}^{d} \int_{x_k=-R}^{+R} e^{2\pi i (j' x_k \hat{w}_k - j x_k w_k^\star)}\, dx_k \right| \tag{470}$$

$$\leq \frac{1}{2R} \left| \int_{x_k=-R}^{+R} e^{2\pi i (j' x_k \hat{w}_k - j x_k w_k^\star)} \, dx_k \right| \tag{471}$$

$$= \frac{1}{2R} \left| \int_{x_k=-R}^{+R} \cos(2\pi (j' x_k \hat{w}_k - j x_k w_k^\star)) \, dx_k \right| \tag{472}$$

$$= \frac{1}{2R} \left| \frac{\sin(2\pi R (j' \hat{w}_k - j w_k^\star))}{\pi (j' \hat{w}_k - j w_k^\star)} \right| \tag{473}$$

$$\leq \frac{1}{2\pi R} \frac{1}{|j' \hat{w}_k - j w_k^\star|}. \tag{474}$$

Here, in the second line, we used Equation (398). In the third line, because we are integrating over a symmetric interval, the sine contribution vanishes. In the fifth line, we use that $|\sin(x)| \leq 1$. We wish to lower bound $|j' \hat{w}_k - j w_k^\star|$. Recall that we chose k such that $|w_k^\star| \geq R_w/\sqrt{d}$ and $|\hat{w}_i - w_i^\star| \leq \epsilon_1$ for all $i \in [d]$ with $\epsilon_1 \leq R_w/(D\sqrt{d})$. Without loss of generality, for $D \geq j' > j \geq 1$, then note that

$$j' \hat{w}_k - j w_k^\star \geq (j' - j) w_k^\star - j' \epsilon_1 \geq \frac{R_w}{\sqrt{d}} - D\epsilon_1 \geq 0. \tag{475}$$

Here, in the first inequality, we use that $\hat{w}_k \geq w_k^\star - \epsilon_1$. In the second inequality, we use that $w_k^\star \geq R_w/\sqrt{d}$ for our choice of k, $j' - j \geq 1$ (since $j' > j$ in this case), and $j' \leq D$. The last inequality holds due to our choice of $\epsilon \leq R_w/(D\sqrt{d})$. Thus, since these terms are nonnegative, then taking the absolute value, we have

$$|j' \hat{w}_k - j w_k^\star| \geq |(j' - j) w_k^\star - j' \epsilon_1|. \tag{476}$$

We can further lower bound this using the reverse triangle inequality:

$$|j' \hat{w}_k - j w_k^\star| \geq ||j' - j||w_k^\star| - j' \epsilon_1|. \tag{477}$$

One can arrive at the same inequality for $j' \leq j$ as well by a similar argument. Thus, from here, we can simply consider any $j' \neq j$. Since $j \neq j'$, then $|j' - j| \geq 1$, and we also know that $j' \leq D$. Thus, we have

$$|j' - j||w_k^\star| - j' \epsilon_1 \geq |w_k^\star| - D\epsilon_1 \geq 0, \tag{478}$$

where the last inequality follows again by our choice of k with $|w_k^\star| \geq R_w/\sqrt{d}$ and the choice of ϵ_1. Thus, taking the absolute value, we have

$$|j' \hat{w}_k - j w_k^\star| \geq ||w_k^\star| - D\epsilon_1|. \tag{479}$$

Finally, using that $|w_k^\star| \geq R_w/\sqrt{d}$, we have

$$|j' \hat{w}_k - j w_k^\star| \geq \left| \frac{R_w}{\sqrt{d}} - D\epsilon_1 \right| \geq \frac{R_w}{\sqrt{d}} - D\epsilon_1. \tag{480}$$

Plugging this back into Equation (474), then we have

$$\left| \int_{x \sim \varphi^2} e^{2\pi i (j' x^\intercal \hat{w} - j x^\intercal w^\star)} \, dx \right| \leq \frac{1}{2\pi R} \frac{\sqrt{d}}{R_w - D\sqrt{d}\epsilon_1}. \tag{481}$$

Putting this together with Equation (468), we can bound the first term in Equation (467)

$$\left| \int_{x \sim \varphi^2} \cos(2\pi (j x^\intercal w^\star - j' x^\intercal \hat{w})) \, dx \right| \leq \frac{1}{2\pi R} \frac{\sqrt{d}}{R_w - D\sqrt{d}\epsilon_1}. \tag{482}$$

We can similarly bound the second term in Equation (467) using the same approach. Thus, with Equation (467), we have the desired bound. □

V. SUPPLEMENTARY NOTE 5 - NON-UNIFORM DISTRIBUTIONS

In this section, we repeat the steps of Section IV when instead given QSQ access to quantum example states with respect to a non-uniform distribution satisfying some technical assumptions.

Recall that we want to learn the target function $g_{w^\star}(x) = \tilde{g}(x^\intercal w^\star)$ for some unknown $w^\star \in \mathbb{R}^d$ and $\tilde{g}$ a function given in Equation (10). We refer to the definitions in Section II for the precise problem statement. Again, the overall idea of the algorithm is to apply period finding to find the unknown vector $w^\star$ one component at a time. Then, given the form of $\tilde{g}$ from Equation (10), we can find the unknown parameters $\beta_j^\star$ via gradient methods.

As in Section IV, we need to suitable discretize and truncate our target function. In addition, we also need to introduce a suitable discretization of our distribution. For the discretization of the target function, the results from Section IV A carry over. Thus, we refer to the discretized function as $h_{w^\star,M_1,M_2}$, which has discretization parameters $M_1, M_2 \in \mathbb{Z}$.

Now, we define our discretized distribution and state our assumptions. Consider a nonnegative function $p : \mathbb{R}^d \to [0,1]$ that can be written as

$$p(x) \triangleq \prod_{j=1}^{d} p_j(x_j) \tag{483}$$

for some nonnegative function $p_j : \mathbb{R} \to [0,1]$. Let φ^2 denote the probability distribution defined by p^2, suitably normalized. In particular, we consider the quantum example state

$$|h_{w^\star,M_1,M_2}\rangle = \frac{1}{\tilde{G}} \sum_{x_1,\ldots,x_d=-R}^{R-1} p(x)\,|x\rangle\,|h_{w^\star,M_1,M_2}(x)\rangle\,, \quad \tilde{G} \triangleq \sum_{x_1,\ldots,x_d=-R}^{R-1} p^2(x), \tag{484}$$

where $\tilde{G}$ is a normalization constant and $x = x_1 \cdots x_d$. Throughout the rest of this section, we suppose that we are given access to quantum statistical queries with respect to this example state and discretization/truncation parameters M_1, M_2, R. We note that one can consider preparing example states by first preparing $\sum_x p_\Sigma(x)\,|x\rangle$ and then evaluating the function $h_{w^\star,M_1,M_2}$ coherently. to Algorithms to prepare this superposition over all inputs x for, e.g., discrete Gaussian distributions, has been well-studied [36–42].

We consider the following additional assumptions on the probability distributions:

1. φ^2 is Fourier concentrated (Definition 3).
2. For a chosen truncation parameter R, for all $x \in [-R, R]$ and $j \in [d]$, then $|1 - p_j^2(x)| \leq 1/10$.
3. The functions $p_j : \mathbb{R} \to [0,1]$ are nonnegative and bounded by 1.
4. p^2 is an even function.
5. Let M_1 be a chosen discretization parameter, and let the truncation parameter be $R \triangleq \tilde{R}M_1$ for some suitably chosen $\tilde{R} \geq 1$. Then, the derivative of p_j' is bounded: $|p_j'(M_1 x)| \leq \frac{\pi D R_w}{2M_1}$ and $|p_j'(M_1 x + T)| \leq \frac{\pi D R_w}{2M_1}$ for all $x \in [-\tilde{R}, \tilde{R}]$ and $j \in [d]$, where T is a guess for the period from Hallgren's algorithm (Section I B).
6. p^2 has a constant number of critical points.

Note that Assumption 1 is necessary in order for classical hardness to hold [12]. Also, one can think of Assumption 5 as just needing this bound on the absolute value of the derivative for all inputs. We state it more specifically in the form we require for the proofs. While these assumptions may seem restrictive at first, we show later in this section that they are satisfied by several natural distributions when taking the scale parameter large enough, such as Gaussians, generalized Gaussians [43], and logistic distributions. With these assumptions on the input distribution, we can efficiently learn the target functions $g_{w^\star}$ using QSQs.

Theorem 8 (Guarantee; Non-Uniform Case)**.** *Let $\epsilon, \delta > 0, \tau \geq 0$. Let $\varphi^2 \propto \prod_{j=1}^d p_j^2$ be a probability distribution over $[-R,R]^d$ satisfying Assumptions 1-6 for the parameters specified shortly. Let $w^\star \in \mathbb{R}^d$ be unknown with norm $R_w > 0$ and $w_j^\star \geq R_w/d^2$, for all $j \in [d]$. Let $g_{w^\star} : \mathbb{R}^d \to [-1,1]$ be defined as $g_{w^\star}(x) = \tilde{g}(x^\intercal w^\star)$, where $\tilde{g} : \mathbb{R} \to [-1,1]$ is a function defined in Equation (10). Consider parameters $M_1 = \max(70\pi d^2 D^3 R_w, R_w^2/\epsilon_1)$, $M_2 = cM_1$, where c is any constant such that M_2 is an integer and $c < 1/(8\pi D R_w)$, and*

$$\tilde{R} = \tilde{\Omega}\left(\max\left(\frac{\tau M_1^2 d^4}{R_w^2}, \frac{D^2}{\epsilon}, \frac{D^2\sqrt{d}}{R_w \epsilon}, \frac{D^{5/2}}{\sqrt{\epsilon}}, \frac{D^{3/2}\sqrt{d}}{R_w\sqrt{\epsilon}}, \frac{d^2 D}{R_w^2}\right)\right), \quad \epsilon_1 = \tilde{\mathcal{O}}\left(\min\left(\frac{\epsilon^3}{D^6 d}, \frac{\epsilon^{3/2}}{D^{13/2} d}, \frac{R_w}{D\sqrt{d}}\right)\right). \tag{485}$$

Suppose we have QSQ access (see Definition 1) with respect to discretization parameters $M_{1,m} \triangleq mM_1$, $M_{2,m} \triangleq mM_2$ *and a truncation parameter* $R \geq \tilde{R}$, *for* $m \in \{1, \ldots, D\}$. *Then, there exists a quantum algorithm with this QSQ access that can efficiently find parameters* $\hat{\beta} \in \mathbb{R}^D$ *such that* $\mathcal{L}_{w^\star}(\hat{\beta}) \leq \epsilon$ *with probability at least* $1 - \delta$. *Moreover, this algorithm uses*

$$N = \mathcal{O}\left(dD \log\left(\frac{1}{\delta}\right) \log^5\left(\frac{M_1 d^2}{R_w}\right)\right) \tag{486}$$

quantum statistical queries with tolerance $\tau \leq \min\left(\frac{1}{M_2^2}\left(\frac{5}{42} - \frac{3}{2M_2}\right), \frac{1}{2D^2 M_2^2}\left(\frac{2}{9} - \frac{1}{8}\left(\frac{2\pi R_w}{M_1}\right)^2 + \frac{3D^2}{M_2}\right)\right)$ *and*

$$t = \Theta\left(\log\left(\sqrt{\frac{D}{\epsilon}}\right)\right) \tag{487}$$

iterations of gradient descent.

As in the uniform case, our algorithm uses QSQs with different choices of discretization/truncation parameters for the two subroutines of Hallgren's algorithm (Section I B): quantum Fourier sampling and the verification procedure. In the quantum Fourier sampling part, we use QSQs with respect to discretization parameters M_1, M_2 and truncation parameter $\tilde{R} = R$. For verification, we use discretization parameters $M_{1,m} \triangleq mM_1, M_{2,m} \triangleq mM_2$ and truncation parameter $\tilde{R} = RM_{1,m}$ for $m \in \{1, \ldots, D\}$.

Note that for non-uniform distributions, Hallgren's algorithm does not immediately apply. To remedy this, we give a new analysis for Hallgren's algorithm for non-uniform distributions satisfying our assumptions. In particular, the quantum Fourier sampling part of this algorithm only requires Assumptions 2 and 3.

While it may seem like we have many (potentially restrictive) assumptions, the next few results show that it still captures natural classes of distributions. In particular, the next proposition shows that generalized Gaussian distributions [43] with a large enough scale parameter satisfy all of them. As a corollary, Gaussians with large enough variance also satisfy the assumptions.

Proposition 2 (Generalized Gaussians satisfy assumptions)**.** *Let* $\tau \geq 0$. *Let* $\alpha_j \geq 2$ *be even shape parameters, and let* $s_j > 0$ *be scale parameters specified later, for* $j \in [d]$. *Let* $p^2(x) = \prod_{j=1}^d \exp(-(x_j/s_j)^{\alpha_j})$ *and* $\varphi^2 \propto p^2$ *suitably normalized so that* φ^2 *is a generalized Gaussian distribution. Let* M_1, M_2 *be discretization parameters with* $M_1 \geq R_w$. *Let*

$$\tilde{R} = \tilde{\Omega}\left(\max\left(\frac{\tau M_1^2 d^4}{R_w^2}, \frac{D^2\sqrt{d}}{R_w}, \max_j \left(\frac{d^2}{R_w}\right)^{a_j - 1} \frac{D}{R_w}\right)\right) \tag{488}$$

and let $R \geq \tilde{R}$ *be the truncation parameter. Then, if* $s_j \geq 2R\sqrt{\pi}$ *for all* $j \in [d]$, *Assumptions 1-6 are satisfied for* φ^2 *for truncation parameter* R. *In particular,* φ^2 *is* $\epsilon(r)$*-Fourier-concentrated with* $\epsilon(r)$ *decaying superpolynomially in* r.

The corollary follows easily because the Gaussian distribution is a special case of the generalized Gaussian for shape parameter $\alpha_j = 2$. Fourier concentration follows by standard Gaussian concentration arguments.

Corollary 9 (Gaussians satisfy assumptions)**.** *Let* $\tau \geq 0$. *Let* $\sigma_j > 0$ *be standard deviations to be specified later. Let* $p(x) \triangleq \exp\left(-x^\intercal \Sigma^{-1} x/2\right) = \prod_{j=1}^d \exp\left(-x_j^2/(2\sigma_j^2)\right)$. *Let* $\varphi^2 \propto p^2$ *suitably normalized so that* φ^2 *is a Gaussian distribution with a diagonal covariance matrix* $\Sigma = \mathrm{diag}(\sigma_1^2, \ldots, \sigma_d^2)$. *Let* M_1, M_2 *be discretization parameters, with* $M_1 \geq R_w$. *Let*

$$\tilde{R} = \tilde{\Omega}\left(\max\left(\frac{\tau M_1^2 d^4}{R_w^2}, \frac{D^2\sqrt{d}}{R_w}, \frac{d^2 D}{R_w^2}\right)\right) \tag{489}$$

and let $R \geq \tilde{R}$ *be the truncation parameter. Then, if* $\sigma_j \geq 2R\sqrt{\pi}$ *for all* $j \in [d]$, *Assumptions 1-6 are satisfied for* φ^2 *for truncation parameter* R. *In particular,* φ^2 *is* $\epsilon(r)$*-Fourier-concentrated with* $\epsilon(r) = \exp\left(-\Omega(r^2)\right)$.

Note here that we distinguish between R and $\tilde{R}$. This is because our algorithm, as explained above, uses different discretization/truncation parameters for different subroutines, and some assumptions are

only relevant for particular subroutines. Assumption 5, in particular, is used in the verification subroutine from Hallgren's algorithm (Section I B), which is why we consider the truncation parameter $R = \tilde{R}M_1$. Note that for these different choices of truncation parameters, the required lower bound on the scale parameter s_j also changes. To satisfy all conditions simultaneously, one may take $s_j \geq RDM_1\sqrt{5\pi\sqrt{18}}$.

Proof of Proposition 2. We consider the functions $p_j^2(x) = \exp(-(x/s_j)^{\alpha_j})$. For even $\alpha_j \geq 2$, then p_j is a Schwartz function. Moreover, since the product of Schwartz functions is still a Schwartz function, then φ is also a Schwartz function. The Fourier transform of a Schwartz function is also a Schwartz function (see, e.g., Proposition 11.25 of [44]). Thus, φ^2 is $\epsilon(r)$-Fourier-concentrated with superpolynomially decaying $\epsilon(r)$, satisfying Assumption 1.

Moreover, Assumptions 3, 4, and 6 are clearly satisfied. For Assumption 2, we have

$$|1 - p_j^2(x)| = \left|1 - e^{-\left(\frac{x}{s_j}\right)^{\alpha_j}}\right| \leq \left|\left(\frac{x}{s_j}\right)^{\alpha_j}\right| \leq \frac{R^{\alpha_j}}{s_j^{\alpha_j}} \leq \frac{1}{(4\pi)^{\alpha_j/2}} \leq \frac{1}{4\pi} \leq \frac{1}{10}, \tag{490}$$

where in the first inequality, we use that $|e^{-x^{\alpha}} - 1| \leq x^{\alpha}$ for all x. In the second inequality, we use that $x \in [-R, R]$. In the third inequality, we use that $s_j \geq 2R\sqrt{\pi}$. In the last inequality, we use that $\alpha_j \geq 2$. Thus, Assumption 2 is satisfied.

Finally, we need to show that Assumption 5 is satisfied as well. Consider truncation parameter $R = \tilde{R}M_1$ for $\tilde{R}$ and M_1 defined in the proposition statement. First, we show that $|p_j'(M_1x)| \leq \pi DR_w/(2M_1)$ for all $x \in [-\tilde{R}, \tilde{R}]$. Taking the derivative, we have

$$p_j'(x) = -\frac{\alpha_j}{2}\frac{x^{\alpha_j - 1}}{s_j^{\alpha_j}} e^{-\frac{1}{2}\left(\frac{x}{s_j}\right)^{\alpha_j}}. \tag{491}$$

Plugging in M_1x, we have

$$|p_j'(M_1x)| \leq \frac{\alpha_j}{2s_j^{\alpha_j}}|M_1x|^{\alpha_j - 1} \leq \frac{\alpha_j}{2s_j^{\alpha_j}}(M_1\tilde{R})^{\alpha_j - 1} \leq \frac{\alpha_j}{2(4\pi)^{\alpha_j/2}}\frac{1}{M_1\tilde{R}} \leq \frac{\pi DR_w}{360M_1}, \tag{492}$$

where in the first inequality, we use that $e^{-\pi z} \leq 1$. In the second inequality, we use that $x \in [-\tilde{R}, \tilde{R}]$. In the third inequality, we use that $s_j \geq 2R\sqrt{\pi} = 2\tilde{R}M_1\sqrt{\pi}$. In the last inequality, we use $\tilde{R} \geq 54D^2\sqrt{d}/(\pi R_w) \geq 54/(\pi DR_w)$ by our choice of $\tilde{R}$ and the maximum of the function $x/(2(4\pi)^{x/2})$. Thus, $|p_j'(M_1x)|$ satisfies the required bound. For $|p_j'(M_1x + T)|$, we have

$$|p_j'(M_1x + T)| \leq \left|\frac{\alpha_j}{2}\frac{(M_1x + T)^{\alpha_j - 1}}{s_j^{\alpha_j}}\right| \leq \frac{\alpha_j}{2s_j^{\alpha_j}}(M_1\tilde{R} + |T|)^{\alpha_j - 1}. \tag{493}$$

In the last inequality, we use triangle inequality and $x \in [-\tilde{R}, \tilde{R}]$. To bound T, we need to appeal to the specifics of the problem, namely how the guess T is produced from Hallgren's algorithm (Algorithm 1). Note we will later show that Hallgren's algorithm works for non-uniform distributions, but the following analysis is the same regardless so it suffices to recall the analysis for the uniform case from Theorem 5. In particular, consider Step 4 of Algorithm 1. Here, T is either $\lfloor \alpha_i\tilde{R}/\alpha \rfloor$ or $\lceil \alpha_i\tilde{R}/\alpha \rceil$, where α, β are the outputs of running quantum Fourier sampling using QSQs and α_i/β_i are the convergents of the continued fraction expansion of α/β. In the proof of Theorem 5, we show that e/f for $1 \leq e, f \leq M_1/w_k^\star$ are convergents of the continued fraction expansion of α/β. We also showed that

$$|\alpha - b| \leq \tau, \qquad \left|b - \frac{e\tilde{R}w_k^\star}{M_1}\right| \leq \frac{1}{2}, \tag{494}$$

for an integer $e \geq 1$, see, e.g., Equation (233). Then, we have

$$\alpha \geq b - \tau \geq \frac{\tilde{R}w_k^\star}{M_1} - \tau - \frac{1}{2} \geq \frac{R_w\tilde{R}}{M_1d^2} - \left(\tau - \frac{1}{2}\right) \geq 6\left(\frac{1}{2} + \tau\right)\frac{M_1d^2}{R_w} - \left(\tau + \frac{1}{2}\right) \geq 5\left(\frac{1}{2} + \tau\right) \geq 1. \tag{495}$$

Here, in the third inequality, we use that $w_k^\star \geq R_w/d^2$. In the fourth inequality, we use that $\tilde{R} \geq 6(1/2 + \tau)M_1^2d^4/R_w^2$. In the fifth inequality, we use that $M_1/w_k^\star \geq R_w/w_k^\star \geq 1$ by our choice of M_1 so that $M_1d^2/R_w \geq M_1/w_k^\star \geq 1$ as well. Finally, in the last inequality, we use that the QSQ tolerance is $\tau \geq 0$. We can use this to bound $|T|$. Let $\lfloor \cdot \rceil$ denote either $\lfloor \cdot \rfloor$ or $\lceil \cdot \rceil$.

$$|T| = \left|\left\lfloor \frac{e\tilde{R}}{\alpha} \right\rceil\right| \leq \frac{e\tilde{R}}{\alpha} + 1 \leq \frac{M_1\tilde{R}}{w_k^\star\alpha} + 1 \leq \frac{M_1d^2\tilde{R}}{R_w} + 1 \leq \frac{2M_1d^2\tilde{R}}{R_w}. \tag{496}$$

In the second inequality, we use that $e \leq M_1/w_k^\star$. In the third inequality, we use that $w_k^\star \geq R_w/d^2$ and $\alpha \geq 1$. Putting everything together with Equation (493), then we have

$$|p_j'(M_1 x + T)| \leq \frac{\alpha_j}{2s_j^{\alpha_j}} \left(M_1 \tilde{R} + \frac{2M_1 d^2 \tilde{R}}{R_w} \right)^{\alpha_j - 1} \tag{497}$$

$$= \frac{\alpha_j}{2s_j^{\alpha_j}} \left(1 + \frac{2d^2}{R_w} \right)^{\alpha_j - 1} (M_1 \tilde{R})^{\alpha_j - 1} \tag{498}$$

$$\leq \frac{\alpha_j}{2(4\pi)^{\alpha_j/2}} \left(1 + \frac{2d^2}{R_w} \right)^{\alpha_j - 1} \frac{1}{M_1 \tilde{R}} \tag{499}$$

$$\leq \frac{3}{20} \frac{\pi D R_w}{M_1}, \tag{500}$$

where in the third line, we use $s_j \geq 2R\sqrt{\pi} = 2\tilde{R}M_1\sqrt{\pi}$. In the last line, we use that $\tilde{R} \geq (d^2/R_w)^{\alpha_j - 1} D/(\pi R_w) \geq (d^2/R_w)^{\alpha_j - 1}/(\pi D R_w)$. This gives the desired bound on $|p_j'(M_1 x + T)|$ as well, completing the proof. □

As another example, our assumptions are also satisfied by logistic distributions.

Proposition 3 (Logistic distributions satisfy assumptions)**.** *Let $s_j > 0$ be scale parameters specified later for $j \in [d]$. Let $p^2(x) = \prod_{j=1}^d \operatorname{sech}^2(x_j/(2s_j))$ and $\varphi^2 \propto p^2$ suitably normalized so that φ^2 is a logistic distribution. Let M_1, M_2 be discretization parameters. Let $\tilde{R} = \tilde{\Omega}(\tau M_1^2 d^4/R_w^2, \sqrt{d})$ and let $R \geq \tilde{R}$ be the truncation parameter. Then, if $s_j \geq \max(4\pi R, M_1/(\pi D R_w))$ for all $j \in [d]$, Assumptions 1-6 are satisfied for φ^2 for truncation parameter R. In particular, φ^2 is $\epsilon(r)$-Fourier-concentrated with $\epsilon(r) = \exp(-\Omega(rd))$.*

Proof. We consider the functions $p_j(x) = \operatorname{sech}(x/(2s_j))$. Assumptions 3, 4, and 6 are clearly satisfied.

Consider Assumption 1. Properly normalized, we have $\varphi_j^2(x) = \operatorname{sech}^2(x/(2s_j))/(4s_j)$, where $\varphi^2(x) = \prod_{j=1}^d \varphi_j^2(x_j)$. Thus, $\varphi_j(x) = \operatorname{sech}(x/(2s_j))/(2\sqrt{s_j})$. Then, the Fourier transform of φ_j is

$$\hat{\varphi}_j(y) = \frac{1}{2\sqrt{s_j}} \int_{-\infty}^{+\infty} e^{-2\pi i x y} \operatorname{sech}\left(\frac{x}{2s_j}\right) dx = \pi\sqrt{s_j} \operatorname{sech}\big(2s_j \pi^2 y\big). \tag{501}$$

We can use this to compute the Fourier transform of φ:

$$\hat{\varphi}(y) = \int_{\mathbb{R}^d} e^{-2\pi i x^\intercal y} \varphi(x)\, dx = \prod_{j=1}^d \int_{-\infty}^{+\infty} e^{-2\pi i x_j y_j} \varphi_j(x_j) = \prod_{j=1}^d \hat{\varphi}_j(y_j) = \pi^d \prod_{j=1}^d \sqrt{s_j} \operatorname{sech}\big(2s_j\pi^2 y_j\big) \tag{502}$$

Because $\|\varphi\|_2 = \|\hat{\varphi}\|_2$, then $\|\hat{\varphi}\|_2 = 1$. Then, to show Fourier concentration (Definition 3), we want to show that $\|\hat{\varphi} \cdot \mathbb{1}_{\geq r}\|_2 \leq \epsilon(r)$ for some function $\epsilon(r)$ and $\mathbb{1}_{\geq r}$ is the indicator function for $\{x : \|x\|_2 \geq r\}$. We have

$$\|\hat{\varphi} \cdot \mathbb{1}_{\geq r}\|_2^2 = \int_{\|y\|_2 \geq r} \hat{\varphi}^2(y)\, dy = \pi^{2d} \int_{\|y\|_2 \geq r} \prod_{j=1}^d s_j \operatorname{sech}^2(2s_j\pi^2 y_j)\, dy \tag{503}$$

Consider the hypercube inscribed in the hypersphere $\|y\|_2 \leq r$:

$$S \triangleq \left\{ y \in \mathbb{R}^d : -\frac{r}{\sqrt{d}} \leq y_1, \ldots, y_d \leq \frac{r}{\sqrt{d}} \right\} \subseteq \{y \in \mathbb{R}^d : \|y\|_2 \leq r\}. \tag{504}$$

Thus, $\{y : \|y\|_2 \geq r\} \subseteq \mathbb{R}^d \setminus S$ so that we can bound the integral over this domain

$$\|\hat{\varphi} \cdot \mathbb{1}_{\geq r}\|_2^2 \leq \pi^{2d} \int_{\mathbb{R}^d \setminus S} \prod_{j=1}^d s_j \operatorname{sech}^2(2s_j\pi^2 y_j)\, dy = \prod_{j=1}^d \left(2\pi^2 s_j \int_{r/\sqrt{d}}^{+\infty} \operatorname{sech}^2(2s_j\pi^2 y_j)\, dy_j \right), \tag{505}$$

where we also used that sech^2 is an even function. Evaluating the integral, we have

$$\|\hat{\varphi} \cdot \mathbb{1}_{\geq r}\|_2^2 \leq \prod_{j=1}^d \left(\int_{2s_j\pi^2 r/\sqrt{d}}^{+\infty} \operatorname{sech}^2(u_j)\, du_j \right) \tag{506}$$

$$= \prod_{j=1}^{d} \left(1 - \tanh\left(\frac{2s_j\pi^2 r}{\sqrt{d}}\right)\right) \tag{507}$$

$$= \prod_{j=1}^{d} \left(1 - \frac{e^{4s_j\pi^2 r/\sqrt{d}} - 1}{e^{4s_j\pi^2 r/\sqrt{d}} + 1}\right) \tag{508}$$

$$= \prod_{j=1}^{d} \left(\frac{2}{e^{4s_j\pi^2 r/\sqrt{d}} + 1}\right) \tag{509}$$

$$\leq \prod_{j=1}^{d} \left(2e^{-4s_j\pi^2 r/\sqrt{d}}\right) \tag{510}$$

$$\leq 2^d e^{-4\pi^2 rd} \tag{511}$$

$$= e^{-\Omega(rd)}. \tag{512}$$

In the first line, we use the change of variables $u_j = 2s_j\pi^2 y_j$. In the second to last line, we use that $s_j \geq 4\pi R \geq 4\pi\sqrt{d} \geq \sqrt{d}$. Thus, Assumption 1 is satisfied with $\epsilon(r) = e^{-\Omega(rd)}$.

For Assumption 2, we have

$$|1 - p_j^2(x)| = \left|1 - \operatorname{sech}^2\left(\frac{x}{2s_j}\right)\right| = 1 - \frac{4e^{x/s_j}}{(e^{x/s_j}+1)^2}. \tag{513}$$

For $x \in [0, R]$, $e^x \leq e^R$ and $e^x \geq 1$ so that

$$|1 - p_j^2(x)| \leq 1 - \frac{4}{(e^{R/s_j}+1)^2} \leq 1 - \frac{4}{(e^{1/(4\pi)}+1)^2} \leq \frac{1}{10}, \tag{514}$$

where in the second inequality, we used that $s_j \geq 4\pi R$. Since p_j^2 is symmetric, the same holds for $x \in [-R, 0]$.

Finally, we need to check Assumption 5. Taking the derivative, we have

$$p_j'(x) = -\frac{1}{2s_j} \tanh\left(\frac{x}{2s_j}\right) \operatorname{sech}\left(\frac{x}{2s_j}\right). \tag{515}$$

Plugging in $M_1 x$ for $x \in [-\tilde{R}, \tilde{R}]$, then

$$|p_j'(M_1 x)| = \left|\frac{1}{2s_j} \tanh\left(\frac{M_1 x}{2s_j}\right) \operatorname{sech}\left(\frac{M_1 x}{2s_j}\right)\right| \leq \frac{1}{2s_j} \leq \frac{\pi D R_w}{2M_1}, \tag{516}$$

where the last inequality comes from $s_j \geq M_1/(\pi D R_w)$. A similar calculation holds for $p_j'(M_1 x + T)$ so that Assumption 5 holds. □

As a corollary of Theorem 8, we obtain the same complexity for learning over generalized Gaussians, Gaussians, and logistic distributions with large enough scale parameters. Note that in the generalized Gaussian case, one may need to take a larger truncation parameter (as specified in Proposition 2), but the sample complexity remains the same. Thus, using the Fourier-concentration in Propositions 2 and 3 and Corollary 9, we see that Gaussian, generalized Gaussian, and logistic distributions achieve an exponential sample complexity quantum advantage.

The next sections are dedicated to proving Theorem 8. In Section V A, we discuss the non-uniform period finding algorithm. In Section V B, similarly to Section IV B, we show how one can use gradient descent to learn the outer function $\tilde{g}$ given knowledge of $w^\star$. In Section V C, we prove some integral bounds which are useful for both Sections V A and V B.

A. Learning the linear function

In this section, we discuss how to use period finding to learn the inner linear function, i.e., how to find the vector of coefficients $w^\star \in \mathbb{R}^d$, when given QSQ access to an example state with non-uniform amplitudes, when the non-uniform distribution satisfies Assumptions 1-6. We also utilize the results regarding pseudoperiodicity from Section IV A.

In Section V A 1, we consider a simple special case for pedagogical purposes to demonstrate how our non-uniform period finding algorithm works. In Section V A 2, we prove the general case.

1. *Warmup*

As a warmup, let us consider the simple case where $1/w^\star_j \in \mathbb{Z}$ for all $j \in [d]$. In fact, we prove a general guarantee on period finding for states with non-uniform amplitudes. Then, the result in our setting, i.e., for learning $w^\star$ from access to $g_{w^\star}$, is a special case. Note that in this simple case, we only need Assumptions 1-3 to hold for our non-uniform distributions. Moreover, Assumption 1 is only needed for classical hardness and is not required for the correctness/complexity of our quantum algorithm.

Proposition 4 (Non-Uniform Period Finding; Simple Case)**.** *Let $\varphi^2 \propto \prod_{j=1}^d p_j^2$ be a probability distribution over $[-R,R]^d$ satisfying Assumptions 1-3 for the truncation parameter R specified shortly and discretization parameter 1. Let $\tau \geq 0$. Let $f : \mathbb{Z}^d \to \mathbb{Z}$ be a periodic function with period S_j in each component. Suppose that we know an upper bound A_j on the period S_j. Let $A = \max_j A_j$. Let $R \geq (1+2\tau)A^2$ be the truncation parameter. Then, there exists an algorithm that can learn each $S_1, \ldots, S_d$ exactly with constant probability using*

$$N = d \tag{517}$$

quantum statistical queries with tolerance τ (with respect to the truncated example state).

In our case, by Lemma 5, our target function can be suitably discretized to be periodic with period $S = M/w^\star$. Moreover, note that we know an upper bound on the period $A = Md^2/R_w$ due to Equation (9). Thus, the previous proposition readily applies, giving us the following corollary.

Corollary 10 (Linear Function Guarantee; Simple Non-Uniform Case)**.** *Let $\varphi^2 \propto \prod_{j=1}^d p_j^2$ be a probability distribution over $[-R,R]^d$ satisfying Assumptions 1-3 for the parameters specified shortly. Let $\tau \geq 0$. Let $w^\star \in \mathbb{R}^d$ be unknown with $1/w^\star_j \in \mathbb{Z}$ for all $j \in [d]$. Suppose also that $w^\star_j \geq R_w/d^2$, for all $j \in [d]$. Let $g_{w^\star} : \mathbb{R}^d \to [-1,1]$ be defined as $g_{w^\star}(x) = \tilde{g}(x^\intercal w^\star)$, where $\tilde{g} : \mathbb{R} \to [-1,1]$ is a function with period 1 which has bounded variation on every finite interval. Let $M \geq 1$ be any choice of discretization parameter and let $R \geq (1+2\tau)M^2 d^4/R_w^2$ be the truncation parameter. Then, there exists an algorithm that can learn $w^\star$ exactly with constant probability using*

$$N = d \tag{518}$$

quantum statistical queries with tolerance τ (with respect to the discretized and truncated example state).

Again, because a discrete Gaussian distribution with large enough variance satisfies the assumptions on our non-uniform distribution (Corollary 9), then this corollary holds for discrete Gaussian distributions as a special case. It is instructive to note that one could obtain a similar guarantee for discrete Gaussian distributions by leveraging a discrete Gaussian phase estimation subroutine from [37] (Theorem 4.2). However, one drawback of this approach is that it does not generalize as easily to the pseudoperiodic case when the period is irrational. This seems to stem from the issue that the Fourier transform of a pseudoperiodic function does not have a simple exact form as is the case for periodic functions. Our result in Proposition 4 holds for more general distributions than just discrete Gaussians and generalizes to the pseudoperiodic case, as we will see in the next section. First, we prove Proposition 4.

Proof of Proposition 4. Because φ^2 is a product distribution, we can write the quantum example state as

$$|f\rangle = \bigotimes_{j=1}^{d} \left(\frac{1}{\tilde{G}_j} \sum_{x_j=-R}^{R-1} p_j(x_j) |x_j\rangle \right) |f(x)\rangle, \quad \tilde{G}_j \triangleq \sum_{x_j=-R}^{R-1} p_j^2(x_j), \tag{519}$$

where $\tilde{G}_j$ are normalization constants, and we define $\tilde{G} \triangleq \prod_{j=1}^d \tilde{G}_j$. Because of this factorization, by the same argument as in Section IV A 1, it suffices to consider $d = 1$, as we can perform period finding one component at a time to find each S_j. Thus, from now on, we consider the case of $d = 1$, where we are given QSQ access to the example state

$$|f\rangle = \frac{1}{\sqrt{\tilde{G}}} \sum_{x=-R}^{R-1} p(x) |x\rangle |f(x)\rangle, \tag{520}$$

where R is chosen such that $R \geq (1+2\tau)A^2$ and p satisfies Assumptions 1-3.

Our algorithm is as before: apply the QFT over $q = 2R$ and measure. This can be encoded into a QSQ by querying the observable

$$O = \left(\mathsf{QFT}_q \sum_{\ell \in [M]} \frac{\ell}{M} |\ell\rangle\langle\ell| \, \mathsf{QFT}_q^{-1} \right) \otimes I, \tag{521}$$

where QFT_q denotes the QFT in dimension $q = 2R$, and I is the identity operator acting on the qubits encoding the output $f(x)$. This is exactly the same observable as in Section IV A 1. However, because of the non-uniform amplitudes, a standard analysis of this algorithm does not apply, so we analyze it in the following.

First, notice that by periodicity we can rewrite our example state as

$$|f\rangle = \frac{1}{\sqrt{\tilde{G}}} \sum_{x=-R}^{R-1} p(x) |x\rangle |f(x)\rangle = \frac{1}{\sqrt{\tilde{G}}} \sum_{x=0}^{S-1} \sum_{k=-B}^{B-1} p(x+kS) |x+kS\rangle |f(x)\rangle, \tag{522}$$

where we denote the period of f as S and we write $B \triangleq \lfloor R/S \rfloor$. Applying the QFT over $q = 2R$ to this state, we have

$$\mathsf{QFT}_q |f\rangle = \frac{1}{\sqrt{2R\tilde{G}}} \sum_{x=0}^{S-1} \sum_{y=0}^{2R-1} \sum_{k=-B}^{B-1} e^{2\pi i(x+kS)y/(2R)} p(x+kS) |y\rangle |f(x)\rangle. \tag{523}$$

The probability of measuring some outcome y is then

$$\Pr(\text{measure } y) = \frac{1}{2R\tilde{G}} \left\| \sum_{x=0}^{S-1} \sum_{k=-B}^{B-1} e^{2\pi i(x+kS)y/(2R)} p(x+kS) |f(x)\rangle \right\|^2 \tag{524}$$

$$= \frac{1}{2R\tilde{G}} \sum_{x,z=0}^{S-1} \sum_{k,\ell=-B}^{B-1} e^{2\pi i(x+kS)y/(2R)} e^{-2\pi i(z+\ell S)y/(2R)} p(x+kS) p(z+\ell S) \langle f(z)|f(x)\rangle \tag{525}$$

$$= \frac{1}{2R\tilde{G}} \sum_{x=0}^{S-1} \sum_{k,\ell=-B}^{B-1} e^{2\pi i(x+kS)y/(2R)} e^{-2\pi i(x+\ell S)y/(2R)} p(x+kS) p(x+\ell S) \tag{526}$$

$$= \frac{1}{2R\tilde{G}} \sum_{x=0}^{S-1} \left| \sum_{k=-B}^{B-1} e^{2\pi i(x+kS)y/(2R)} p(x+kS) \right|^2 \tag{527}$$

$$= \frac{1}{2R\tilde{G}} \sum_{x=0}^{S-1} \left| \sum_{k=-B}^{B-1} e^{2\pi ikSy/(2R)} p(x+kS) \right|^2. \tag{528}$$

We want to lower bound this probability for y satisfying

$$\left| y - \frac{aR}{S} \right| \leq \frac{1}{2} \tag{529}$$

for $a \in \mathbb{Z}$, i.e., $y = \lfloor aR/S \rceil$, where $\lfloor x \rceil$ denotes the nearest integer above or below x. We start by lower bounding the term in absolute value via the reverse triangle inequality:

$$\left| \sum_{k=-B}^{B-1} e^{2\pi ikSy/(2R)} p(x+kS) \right| \tag{530}$$

$$= \left| \sum_{k=-B}^{B-1} e^{2\pi ikSy/(2R)} + \sum_{k=-B}^{B-1} e^{2\pi ikSy/(2R)} p(x+kS) - \sum_{k=-B}^{B-1} e^{2\pi ikSy/(2R)} \right| \tag{531}$$

$$\geq \left| \left| \sum_{k=-B}^{B-1} e^{2\pi ikSy/(2R)} \right| - \left| \sum_{k=-B}^{B-1} e^{2\pi ikSy/(2R)} p(x+kS) - \sum_{k=-B}^{B-1} e^{2\pi ikSy/(2R)} \right| \right|. \tag{532}$$

To lower bound this further, we lower bound the first term and upper bound the second. First, to lower bound the first term, we can change the index of summation to see that

$$\left|\sum_{k=-B}^{B-1} e^{2\pi i k S y/(2R)}\right| = \left|\sum_{\ell=0}^{2B-1} e^{2\pi i(\ell-B)Sy/(2R)}\right| = \left|\sum_{\ell=0}^{2B-1} e^{2\pi i \ell S y/(2R)}\right|, \tag{533}$$

where we set $\ell = k + B$. Then, for $y = aR/S + \epsilon$, where $|\epsilon| \leq 1/2$, then this is equal to

$$\left|\sum_{\ell=0}^{2B-1} e^{2\pi i \ell S \epsilon/(2R)}\right| = \left|\sum_{\ell=0}^{2B-1} e^{2\pi i C \ell/(2B)}\right|. \tag{534}$$

Here, we define $C \triangleq BS\epsilon/R$. Because $B = \lfloor R/S \rfloor$, then $SB \leq R$ and hence $|C| \leq |\epsilon| \leq 1/2$. Then, by Lemma 3 in [8] (or Claim 3.1 in [7]), we obtain the desired lower bound:

$$\left|\sum_{k=-B}^{B-1} e^{2\pi i k S y/(2R)}\right| \geq \frac{2}{\sqrt{18}} B. \tag{535}$$

Now, we consider the other term.

$$\begin{aligned}
\left|\sum_{k=-B}^{B-1} e^{2\pi i k S y/2R} p(x+kS) - \sum_{k=-B}^{B-1} e^{2\pi i k S y/2R}\right| &\leq \sum_{k=-B}^{B-1} |p(x+kS) - 1| && (536)\\
&= \sum_{k=-B}^{B-1} (1 - p(x+kS)) && (537)\\
&\leq \frac{B}{5} && (538)\\
&\leq \frac{B}{\sqrt{18}}. && (539)
\end{aligned}$$

Here, in the second line, we use Assumption 3. In the third line, we use Assumption 2. Namely, because $p(x) \leq 1$ by Assumption 3, then $1 - p(x+kS) \leq 1 - p^2(x+kS)$, which is in turn less than $1/10$ by Assumption 2. Note that Assumption 2 applies because for the range of x, k considered, then $x + kS \in [-R, R]$. Putting everything together, we thus see that

$$\left|\sum_{k=-B}^{B-1} e^{2\pi i k S y/2R} p(x+kS)\right| \geq \frac{2}{\sqrt{18}} B - \frac{1}{\sqrt{18}} B = \frac{1}{\sqrt{18}} B. \tag{540}$$

Then, plugging this back into our original expression, the probability that we obtain some output $y = \lfloor aR/S \rceil$ is

$$\begin{aligned}
\Pr\left(y = \left\lfloor \frac{aR}{S} \right\rceil\right) &= \frac{1}{2R\tilde{G}} \sum_{x=0}^{S-1} \left|\sum_{k=-B}^{B-1} e^{2\pi i k S y/2R} p(x+kS)\right| && (541)\\
&\geq \frac{1}{2R\tilde{G}} \sum_{x=0}^{S-1} \frac{1}{18} B^2 && (542)\\
&\geq \frac{1}{72} \frac{1}{R^2} S B^2 && (543)\\
&= \Omega\left(\frac{SB^2}{R^2}\right) && (544)\\
&= \Omega\left(\frac{1}{S}\right). && (545)
\end{aligned}$$

The third line follows because

$$\tilde{G} = \sum_{x=-R}^{R-1} p(x)^2 \leq 2R, \tag{546}$$

using Assumption 3. The last line follows because $B = \lfloor R/S \rfloor = \Theta(R/S)$. Instead of measuring $y = \lfloor aR/S \rfloor$ exactly, we obtain some estimate due to the noisy QSQs. From here, the analysis is the same as that of Proposition 1. In particular, by the choice of $R \geq (1+2\tau)A^2$, so by the same analysis as Proposition 1, we can recover S with constant probability. This only required one QSQ. To learn each period $S_1, \ldots, S_d$, it thus requires d QSQs. □

2. General case

In the previous section, we showed that $w^\star$ can be recovered exactly in a simple case when $1/w^\star \in \mathbb{Z}$ and our example state has amplitudes distributed according to a non-uniform distribution satisfying Assumptions 1-3. In general, $1/w^\star$ may not be an integer, but nevertheless we can again prove a general guarantee on period finding given an example state with non-uniform amplitudes. This is in contrast to standard period finding guarantees which only hold for uniform amplitudes. This then implies that we can find the period of $g_{w^\star}$ as a simple corollary.

We require that our non-uniform distributions satisfy Assumptions 1-6. As in Section V A 1, Assumption 1 is only needed to ensure classical hardness and is not required for the correctness/complexity of our quantum algorithm. Our algorithm is the same as Hallgren's algorithm [7] (see Section I B) but requires a new analysis due to the non-uniform amplitudes. This analysis is similar to that of Section V A 1.

Theorem 9 (Non-Uniform Period Finding)**.** *Let $\varphi^2 \propto \prod_{k=1}^d p_k^2$ be a probability distribution over $[-R,R]^d$ satisfying Assumptions 1-3 for a truncation parameter R specified shortly and discretization parameter 1. Let $\tau, \eta \geq 0$. Let $f : \mathbb{Z}^d \to \mathbb{Z}$ be an η-pseudoperiodic function with period $S_j \geq 1$ in each component. Suppose that, given an integer T, we can efficiently check (in time* $\mathrm{polylog}(S)$*) whether or not $|kS-T| < 1$ for some $k \in \mathbb{Z}$. Suppose that we know an upper bound A_j on the period S_j. Let $A = \max_j A_j$. Let $R \geq 6(1/2+\tau)A^2$ be the truncation parameter. Then, there exists an algorithm that outputs integers a_j such that $|S_j - a_j| \leq 1$ with probability $\Omega(\eta^2/\log^4 A)$ for all $j \in [d]$ using*

$$N = 2d \tag{547}$$

quantum statistical queries with tolerance τ (with respect to the truncated example state).

In the case of $d = 1$, our algorithm is the same as Hallgren's algorithm, which we present in Algorithm 3.

Algorithm 3: Non-Uniform Period Finding

1: Choose a truncation parameter $R \geq 6(1/2+\tau)A^2$.
2: Apply quantum Fourier sampling to the function f over $\mathbb{Z}_q$, $q = 2R$ twice. Let α, β be the outputs.
3: Compute the continued fraction expansion of α/β.
4: For each convergent α_i/β_i in the continued fractions expansion, use the verification procedure to check whether $\lfloor \alpha_i R/\alpha \rfloor$ or $\lceil \alpha_i R/\alpha \rceil$ is an integer multiple of the period S.
5: **return** the smallest value that passed the test from the previous step.

As a corollary, we can apply Theorem 9 to our particular setting to obtain guarantees.

Corollary 11 (Linear Function Guarantee; Non-Uniform Case)**.** *Let $1 > \epsilon_1 > 0, \delta > 0, \tau \geq 0$. Let $\varphi^2 \propto \prod_{k=1}^d p_k^2$ be a probability distribution over $[-R,R]^d$ satisfying Assumptions 1-6 for the parameters specified shortly. Let $w^\star \in \mathbb{R}^d$ be unknown with norm $R_w > 0$ and $w_j^\star \geq R_w/d^2$, for all $j \in [d]$. Let $g_{w^\star} : \mathbb{R}^d \to [-1,1]$ be defined as $g_{w^\star}(x) = \tilde{g}(x^\intercal w^\star)$, where $\tilde{g} : \mathbb{R} \to [-1,1]$ is given in Equation (10). Consider parameters $M_1 = \max(70\pi d^2 D^3 R_w, R_w^2/\epsilon_1), M_2 = cM_1$, where c is any constant such that M_2 is an integer and $c < 1/(8\pi D R_w)$, and*

$$\tilde{R} = \tilde{\Omega}\left(\max\left(\frac{\tau M_1^2 d^4}{R_w^2}, \frac{D^2}{\epsilon}, \frac{D^2\sqrt{d}}{R_w \epsilon}, \frac{D^{5/2}}{\sqrt{\epsilon}}, \frac{D^{3/2}\sqrt{d}}{R_w\sqrt{\epsilon}}, \frac{d^2 D}{R_w^2}\right)\right). \tag{548}$$

Suppose we have QSQ access (see Definition 1) with respect to discretization parameters $M_{1,m} \triangleq mM_1$, $M_{2,m} \triangleq mM_2$ and a truncation parameter $R \geq \tilde{R}$, for $m \in \{1, \ldots, D\}$. Then, there exists a quantum algorithm with this QSQ access that can learn an approximation $\hat{w}$ of $w^\star$ such that $\|\hat{w} - w^\star\|_\infty \leq \epsilon_1$ with probability at least $1-\delta$ using

$$N = \mathcal{O}\left(dD \log\left(\frac{1}{\delta}\right) \log^5\left(\frac{M_1 d^2}{R_w}\right)\right) \tag{549}$$

quantum statistical queries with tolerance $\tau \leq \min\left(\frac{1}{M_2^2}\left(\frac{5}{42} - \frac{3}{2M_2}\right), \frac{1}{2D^2M_2^2}\left(\frac{2}{9} - \frac{1}{8}\left(\frac{2\pi R_w}{M_1}\right)^2 + \frac{3D^2}{M_2}\right)\right)$ *(with respect to the discretized and truncated state).*

As stated before, our algorithm has two subroutines. In one, we use QSQs with respect to discretization parameters M_1, M_2 and truncation parameter $R = \tilde{R}$. In the other, we use discretization parameters $M_{1,m} \triangleq mM_1, M_{2,m} \triangleq mM_2$ and truncation parameter $R = \tilde{R}M_{1,m}$ for $m \in \{1, \ldots, D\}$.

Also, notice that Theorem 9 requires the distribution to satisfy only Assumptions 1-3 while Corollary 11 requires Assumptions 1-6. This is because Assumptions 4-6 are only needed to instantiate the verification procedure for checking if a given integer is close to an integer multiple of the true period. This is assumed in Theorem 9 whereas we need to construct this algorithm in order to obtain Corollary 11. Again, we remark that Assumption 1 is only needed for classical hardness and does not affect the correctness/complexity of our quantum algorithm.

We first prove the correctness of Algorithm 3 as stated in Theorem 9. Then, we will prove Corollary 11 using this.

Proof of Theorem 9. Because φ^2 is a product distribution, we can write the quantum example state as

$$|f\rangle = \bigotimes_{j=1}^{d}\left(\frac{1}{\sqrt{\tilde{G}_j}}\sum_{x_j=-R}^{R-1} p_j(x_j)\,|x_j\rangle\right)|f(x)\rangle\,, \quad \tilde{G}_j \triangleq \sum_{x_j=-R}^{R-1} p_j^2(x_j), \tag{550}$$

where $\tilde{G}_j$ are normalization constants with $\tilde{G} = \prod_j \tilde{G}_j$. Because of this factorization, by the same argument as in Section IV A 1, it suffices to consider $d = 1$, as we can perform period finding one component at a time to find each S_j. Thus, from now on, we consider the case of $d = 1$, where we are given QSQ access to the example state

$$|f\rangle = \frac{1}{\sqrt{\tilde{G}}}\sum_{x=-R}^{R-1} p(x)\,|x\rangle\,|f(x)\rangle\,, \tag{551}$$

where R is chosen such that $R \geq 6(1/2+\tau)A^2$ and p satisfies Assumptions 1-3. To apply quantum Fourier sampling in Step 2 of Algorithm 3, we can encode this into a QSQ by querying the observable

$$O = \left(\mathsf{QFT}_q \sum_{\ell\in[M]} \frac{\ell}{M}\,|\ell\rangle\langle\ell|\,\mathsf{QFT}_q^{-1}\right)\otimes I, \tag{552}$$

where QFT_q denotes the QFT in dimension $q = 2R$, and I is the identity operator acting on the qubits encoding the output $f(x)$. This is exactly the same observable as in Section IV A 1. However, because of the non-uniform amplitudes, a standard analysis of this algorithm does not apply, so we analyze it in the following. The analysis is similar to Proposition 4 but is a bit more complicated due to pseudoperiodicity.

For simplicity, suppose that f is pseudoperiodic on the whole domain rather than an η-fraction. This only affects the probability of success of the algorithm, which we will reintroduce in at the end. Our argument is still valid for only an η-fraction but would lead to unnecessary complications. This is also how the proofs of [7, 8] proceed.

First, notice that by pseudoperiodicity, we can rewrite our example state as

$$|f\rangle = \frac{1}{\sqrt{\tilde{G}}}\sum_{x=-R}^{R-1} p(x)\,|x\rangle\,|f(x)\rangle = \frac{1}{\sqrt{\tilde{G}}}\sum_{x=0}^{S-1}\sum_{k=-B}^{B-1} p(x+[kS])\,|x+[kS]\rangle\,|f(x)\rangle\,, \tag{553}$$

where we denote the period of f as S and we write $B \triangleq \lfloor R/S\rfloor$. We also use $[x]$ to denote a chosen one of the two values $\lfloor x\rfloor$ or $\lceil x\rceil$. Note that this is different from $\lfloor x\rceil$, which denotes rounding to the nearest integer above or below x. Applying the QFT over $q = 2R$ to this state as in Step 2 of the algorithm, we have

$$\mathsf{QFT}_q\,|f\rangle = \frac{1}{\sqrt{2R\tilde{G}}}\sum_{x=0}^{S-1}\sum_{y=0}^{2R-1}\sum_{k=-B}^{B-1} e^{2\pi i(x+[kS])y/(2R)} p(x+[kS])\,|y\rangle\,|f(x)\rangle\,. \tag{554}$$

The probability of measuring some outcome y is then

$$\Pr(\text{measure } y) = \frac{1}{2R\tilde{G}}\left\|\sum_{x=0}^{S-1}\sum_{k=-B}^{B-1} e^{2\pi i(x+[kS])y/(2R)} p(x+[kS])\,|f(x)\rangle\right\|^2 \tag{555}$$

$$= \frac{1}{2R\tilde{G}} \sum_{x,z=0}^{S-1} \sum_{k,\ell=-B}^{B-1} e^{2\pi i(x+[kS])y/(2R)} e^{-2\pi i(z+[\ell S])y/(2R)} p(x+[kS])p(z+[\ell S]) \langle f(z)|f(x)\rangle \tag{556}$$

$$= \frac{1}{2R\tilde{G}} \sum_{x=0}^{S-1} \sum_{k,\ell=-B}^{B-1} e^{2\pi i(x+[kS])y/(2R)} e^{-2\pi i(x+[\ell S])y/(2R)} p(x+[kS])p(x+[\ell S]) \tag{557}$$

$$= \frac{1}{2R\tilde{G}} \sum_{x=0}^{S-1} \left| \sum_{k=-B}^{B-1} e^{2\pi i(x+[kS])y/(2R)} p(x+[kS]) \right|^2 \tag{558}$$

$$= \frac{1}{2R\tilde{G}} \sum_{x=0}^{S-1} \left| \sum_{k=-B}^{B-1} e^{2\pi i[kS]y/(2R)} p(x+[kS]) \right|^2. \tag{559}$$

We want to lower bound this probability for $y = \lfloor aR/S \rceil$ for $a \in \mathbb{Z}$ and $y < R/\log A$. We start by lower bounding the term in absolute value via the reverse triangle inequality:

$$\left| \sum_{k=-B}^{B-1} e^{2\pi i[kS]y/(2R)} p(x+[kS]) \right| \tag{560}$$

$$= \left| \sum_{k=-B}^{B-1} e^{2\pi i[kS]y/(2R)} + \sum_{k=-B}^{B-1} e^{2\pi i[kS]y/(2R)} p(x+[kS]) - \sum_{k=-B}^{B-1} e^{2\pi i[kS]y/(2R)} \right| \tag{561}$$

$$\geq \left| \left| \sum_{k=-B}^{B-1} e^{2\pi i[kS]y/(2R)} \right| - \left| \sum_{k=-B}^{B-1} e^{2\pi i[kS]y/(2R)} p(x+[kS]) - \sum_{k=-B}^{B-1} e^{2\pi i[kS]y/(2R)} \right| \right|. \tag{562}$$

To lower bound this further, we lower bound the first term and upper bound the second. First, to lower bound the first term, we can change the index of summation to see that

$$\left| \sum_{k=-B}^{B-1} e^{2\pi i[kS]y/(2R)} \right| = \left| \sum_{k=-B}^{B-1} e^{2\pi i(kS+\delta_k)y/(2R)} \right| = \left| \sum_{\ell=0}^{2B-1} e^{\frac{2\pi i(\ell-B)Sy+2\pi i\delta_{\ell-B}y}{2R}} \right| = \left| \sum_{\ell=0}^{2B-1} e^{2\pi i(\ell S+\delta_{\ell-B})y/(2R)} \right|. \tag{563}$$

Here, we wrote $[kS] = kS = \delta_k$, where $|\delta_k| < 1$. Then, for $y = aR/S + \epsilon$, where $|\epsilon| \leq 1/2$, then this is equal to

$$\left| \sum_{\ell=0}^{2B-1} e^{2\pi i(\ell S+\delta_{\ell-B})(aR/S+\epsilon)/2R} \right| = \left| \sum_{\ell=0}^{2B-1} e^{2\pi i\left(\frac{\epsilon\ell S}{2R} + \frac{a\delta_{\ell-B}}{S} + \frac{\epsilon\delta_{\ell-B}}{2R}\right)} \right|. \tag{564}$$

Now, define $C \triangleq BS\epsilon/R$. Because $B = \lfloor R/S \rfloor$, then $SB \leq R$ and hence $|C| \leq |\epsilon| \leq 1/2$. Also, note that because we are considering $y = aR/S + \epsilon < R/\log S$ and $|\epsilon| \leq 1/2$, then $a/S < 1/\log S + 1/(2R)$. Since $|\delta_k| < 1$ and $R \geq 6(1/2+\tau)A^2 \geq 3S^2$, then

$$\left| \frac{a\delta_{\ell-B}}{S} + \frac{\epsilon\delta_{\ell-B}}{2R} \right| < \frac{1}{\log S} + \frac{1}{2R} + \frac{1}{4R} \leq \frac{2}{\log S}. \tag{565}$$

Thus, we can write our summation as

$$\left| \sum_{\ell=0}^{2B-1} e^{2\pi i(C\ell/(2B)+\xi(\ell))} \right|, \tag{566}$$

where $|\xi(\ell)| \leq 2/\log S$. By Lemma 3 in [8] (or Claim 3.1 in [7]), we obtain the desired lower bound:

$$\left| \sum_{k=-B}^{B-1} e^{2\pi i[kS]y/(2R)} \right| \geq \frac{2}{\sqrt{18}} B \tag{567}$$

if $y = \lfloor aR/S \rceil$ and $y < R/\log S$. Now, we consider the other term.

$$\left| \sum_{k=-B}^{B-1} e^{2\pi i[kS]y/(2R)} p(x+[kS]) - \sum_{k=-B}^{B-1} e^{2\pi i[kS]y/(2R)} \right| \leq \sum_{k=-B}^{B-1} |p(x+[kS]) - 1| \tag{568}$$

$$= \sum_{k=-B}^{B-1} (1 - p(x + [kS])) \tag{569}$$

$$\leq \frac{B}{5} \tag{570}$$

$$\leq \frac{B}{\sqrt{18}}. \tag{571}$$

Here, in the second line, we use Assumption 3. In the third line, we use Assumption 2. Namely, because $p(x) \leq 1$ by Assumption 3, then $1 - p(x + [kS]) \leq 1 - p^2(x + [kS])$, which is in turn less than $1/10$ by Assumption 2. Note that Assumption 2 applies because for the range of x, k considered, then $x + [kS] \in [-R, R]$.

Putting everything together, we thus see that

$$\left| \sum_{k=-B}^{B-1} e^{2\pi i[kS]y/(2R)} p(x + [kS]) \right| \geq \frac{2}{\sqrt{18}} B - \frac{1}{\sqrt{18}} B = \frac{1}{\sqrt{18}} B \tag{572}$$

for $y = \lfloor aR/S \rceil$ and $y < R/\log S$. Then, plugging this back into our original expression, the probability that we obtain some output $y = \lfloor aR/S \rceil$ and $y < R/\log S$ is

$$\Pr\left(y = \left\lfloor \frac{aR}{S} \text{ and } y < R/\log S \right\rceil \right) = \frac{1}{2R\tilde{G}} \sum_{x=0}^{S-1} \left| \sum_{k=-B}^{B-1} e^{2\pi i[kS]y/2R} p(x + [kS]) \right|^2 \tag{573}$$

$$\geq \frac{1}{2R\tilde{G}} \sum_{x=0}^{S-1} \frac{1}{18} B^2 \tag{574}$$

$$\geq \frac{1}{72} \frac{1}{R^2} SB^2 \tag{575}$$

$$= \Omega\left(\frac{SB^2}{R^2} \right) \tag{576}$$

$$= \Omega\left(\frac{1}{S} \right). \tag{577}$$

Here, the second line follows from our above argument. The third line follows because

$$\tilde{G} = \sum_{x=-R}^{R-1} p^2(x) \leq 2R, \tag{578}$$

where we used Assumption 3. The last line follows because $B = \lfloor R/S \rfloor = \Theta(R/S)$.

There are $S/\log A$ integer multiples of R/S less than $R/\log A$ (and hence less than $R/\log S$). Thus, the probability of measuring two values less than $R/\log A$ (as in Step 2 of Algorithm 3) is $\Omega(1/\log^2 A)$. Furthermore, the probability that the two values are relatively prime is at least $\Omega(1/\log(S/\log A))^2$ by the prime number theorem. The probability of measuring two such values satisfying all the conditions (including pseudoperiodicity) is $\Omega(\eta^2/\log^4 A)$.

Steps 3-5 of Algorithm 3 are analyzed in the case that we obtain a noisy estimate with tolerance τ in the same way as Theorem 5. Thus, we obtain the claim. □

Now, we can prove Corollary 11 using Theorem 9. We need to show that the condition about checking whether a guess for the period is close or not is satisfied. We design such a verification procedure in Algorithm 4 and analyze it in Theorem 10. This is analogous to Theorem 6. As before, in Algorithm 4, we must restrict the noise tolerance of our QSQs to be inverse polynomial in some of our parameters. Classically, the hardness results have access to gradients that are exponentially accurate, so requiring the tolerance parameter to scale inverse polynomially is not particularly strong.

Theorem 10 (Verification Procedure; Non-Uniform Case)**.** *Let $\varphi^2 \propto \prod_{k=1}^d p_k^2$ be a probability distribution over $[-R, R]^d$ satisfying Assumptions 1-6 for a truncation parameter R specified later. Let $1 > \epsilon_1 > 0$. Let $w^\star \in \mathbb{R}^d$ be unknown with norm $R_w > 0$ and $w_j^\star \geq R_w/d^2$ for all $j \in [d]$. Let $g_{w^\star} : \mathbb{R}^d \to [-1, 1]$ be defined as $g_{w^\star}(x) = \tilde{g}(x^\intercal w^\star)$ for $\tilde{g}$ given in Equation (10). Consider parameters $M_1 = \max(70\pi d^2 D^3 R_w, R_w^2/\epsilon_1)$, $M_2 = cM_1$, where c is any constant such that $M_2 \in \mathbb{Z}$ and*

Algorithm 4: Verification Procedure; Non-Uniform Case

1: Choose parameters $M_1 = \max(70\pi d^2 D^3 R_w, R_w^2/\epsilon_1)$, $M_2 = cM_1$ for c any constant such that $M_2 \in \mathbb{Z}$ and $c < 1/(8\pi D R_w)$, and $\tilde{R} = \tilde{\Omega}\left(\max\left(\frac{\tau M_1^2 d^4}{R_w^2}, \frac{D^2}{\epsilon}, \frac{D^2\sqrt{d}}{R_w \epsilon}, \frac{D^{5/2}}{\sqrt{\epsilon}}, \frac{D^{3/2}\sqrt{d}}{R_w\sqrt{\epsilon}}, \frac{d^2 D}{R_w^2}\right)\right)$.

2: For $m \in \{1, \ldots, D\}$, query the QSQ oracle with observable $O_{k,m}$ (defined in Equation (142)), discretization parameters $M_{1,m} \triangleq mM_1$, $M_{2,m} \triangleq mM_2$, truncation parameter $R \triangleq \tilde{R}M_{1,m}$, and tolerance $\tau \leq \min\left(\frac{1}{M_2^2}\left(\frac{5}{42} - \frac{3}{2M_2}\right), \frac{1}{2D^2M_2^2}\left(\frac{2}{9} - \frac{1}{8}\left(\frac{2\pi R_w}{M_1}\right)^2 + \frac{3D^2}{M_2}\right)\right)$ to obtain values α_m.

3: Check if $\alpha_1 \geq \frac{1}{M_2^2}\left(\frac{5}{14} - \frac{9}{2M_2}\right)$.

4: Check if $\sum_{m=1}^{D} \alpha_m \leq \frac{1}{M_2^2}\left(\frac{13}{25}D + \frac{1}{2D}\left(\frac{2}{9} - \frac{1}{8}\left(\frac{2\pi R_w}{M_1}\right)^2 + \frac{3D^2}{M_2}\right)\right)$.

5: **return** "yes" iff both conditions in Steps 3 and 4 are satisfied.

$c < 1/(8\pi D R_w)$, and

$$\tilde{R} = \tilde{\Omega}\left(\max\left(\frac{\tau M_1^2 d^4}{R_w^2}, \frac{D^2}{\epsilon}, \frac{D^2\sqrt{d}}{R_w \epsilon}, \frac{D^{5/2}}{\sqrt{\epsilon}}, \frac{D^{3/2}\sqrt{d}}{R_w\sqrt{\epsilon}}, \frac{d^2 D}{R_w^2}\right)\right). \tag{579}$$

Suppose we have QSQ access (see Definition 1) with respect to discretization parameters $M_{1,m} \triangleq mM_1$, $M_{2,m} \triangleq mM_2$ and a truncation parameter $R \triangleq \tilde{R}M_{1,m}$, for $m \in \{1, \ldots, D\}$. Then, given an integer T found as in Algorithm 3 and $k \in [d]$, Algorithm 4 can check whether or not $|T - \frac{\ell M_1}{w_k^\star}| \leq 1$ for some integer ℓ using D QSQs with tolerance $\tau \leq \min\left(\frac{1}{M_2^2}\left(\frac{5}{42} - \frac{3}{2M_2}\right), \frac{1}{2D^2M_2^2}\left(\frac{2}{9} - \frac{1}{8}\left(\frac{2\pi R_w}{M_1}\right)^2 + \frac{3D^2}{M_2}\right)\right)$.

Proof. This proof is similar to Theorem 6, so we omit some details when they follow straightforwardly from Theorem 6. Explicitly, the example state for our QSQ access is

$$\left|h_{w^\star,M_{1,m},M_{2,m}}\right\rangle = \frac{1}{\sqrt{\tilde{G}_d}} \sum_{x_1,\ldots,x_d=-\tilde{R}M_{1,m}}^{\tilde{R}M_{1,m}-1} p_1(x_1)\cdots p_d(x_d)\left|x\right\rangle\left|h_{w^\star,M_{1,m},M_{2,m}}(x)\right\rangle, \tag{580}$$

where

$$\tilde{G}_d \triangleq \sum_{x_1,\ldots,x_d=-\tilde{R}M_{1,m}}^{\tilde{R}M_{1,m}-1} p_1^2(x_1)\cdots p_d^2(x_d) \tag{581}$$

is a normalization constant. Also, $h_{w^\star,M_{1,m},M_{2,m}}$ is a discretization of $g_{w^\star}$ from Lemma 6. Note that p_j satisfies Assumptions 1-6 for the truncation parameter $R = \tilde{R}M_{1,m}$, not $\tilde{R}$. We query D QSQs, each with the different parameters indexed by m as specified previously.

As in Theorem 6, the main idea behind our verification procedure is to compute the inner product between $h_{w^\star,M_{1,m},M_{2,m}}$ and this function with its input shifted by the guess T for the period. This inner product should be large for a good guess. We again consider the observable $O_{k,m}$ defined in Equation (142) which computes the inner product between $h_{w^\star,M_{1,m},M_{2,m}}$ and this function with its input shifted by T.

Claim 6 (Approximating inner product; Non-uniform case)**.** *For $m \in \{1, \ldots, D\}$, consider parameters $M_{1,m}, M_{2,m}$ as defined above. Also consider a parameter $\tilde{R}$ and an observable $O_{k,m}$ as defined above. Then, the expectation value of O_m with respect to the example state in Equation (580) is given by*

$$\left\langle h_{w^\star,M_{1,m},M_{2,m}}\middle|O_{k,m}\middle|h_{w^\star,M_{1,m},M_{2,m}}\right\rangle \tag{582}$$

$$= \frac{1}{\tilde{G}_d M_{2,m}^2} \sum_{x_1,\ldots,x_d=-\tilde{R}M_{1,m}}^{\tilde{R}M_{1,m}-1} p_1^2(x_1)\cdots p_k(x_k)p_k(x_k+T)\cdots p_d^2(x_d)h_{w^\star,M_{1,m},M_{2,m}}(x)h_{w^\star,M_{1,m},M_{2,m}}(x+Te_k), \tag{583}$$

where e_k denotes the unit vector with a single one in the kth coordinate.

We omit the proof of this claim, as it follows in the same way as Claim 1. Now, we want to show that the conditions checked in Steps 3 and 4 in Algorithm 4 are satisfied if and only if $|T - \ell M_1/w_k^\star| \leq 1$. To do so, we first simplify our approximate inner product from Claim 6 further using the particular form of $h_{w^\star,M_{1,m},M_{2,m}}$ from Lemma 6 and $\tilde{g}$ from Equation (10). This follows in the same way as Theorem 6, just carrying along the extra discrete Gaussian terms. For simplicity, denote

$$p_T^2(x) \triangleq p_1^2(x_1)\cdots p_k(x_k)p_k(x_k+T)\cdots p_d^2(x_d). \tag{584}$$

Then, we have

$$\left\langle h_{w^\star,M_{1,m},M_{2,m}} \middle| O_{k,m} \middle| h_{w^\star,M_{1,m},M_{2,m}} \right\rangle \tag{585}$$

$$= \frac{1}{\tilde{G}_d M_{2,m}^2} \sum_{x_1,\ldots,x_d=-\tilde{R}M_{1,m}}^{\tilde{R}M_{1,m}-1} \sum_{j=1}^{D} p_T^2(x)\,(\beta_j^\star)^2 \left(\cos^2\left(\frac{2\pi j x^\intercal w^\star}{M_{1,m}}\right)\cos\left(\frac{2\pi j T w_k^\star}{M_{1,m}}\right)\right.$$
$$\left. - \cos\left(\frac{2\pi j x^\intercal w^\star}{M_{1,m}}\right)\sin\left(\frac{2\pi j x^\intercal w^\star}{M_{1,m}}\right)\sin\left(\frac{2\pi j T w_k^\star}{M_{1,m}}\right)\right) \tag{586}$$

$$+\frac{1}{\tilde{G}_d M_{2,m}^2} \sum_{x_1,\ldots,x_d=-\tilde{R}M_{1,m}}^{\tilde{R}M_{1,m}-1} \sum_{\substack{j,j'=1\\ j\neq j'}}^{D} p_T^2(x)\beta_j^\star\beta_{j'}^\star \left(\cos\left(\frac{2\pi j x^\intercal w^\star}{M_{1,m}}\right)\cos\left(\frac{2\pi j' x^\intercal w^\star}{M_{1,m}}\right)\cos\left(\frac{2\pi j' T w_k^\star}{M_{1,m}}\right)\right.$$
$$\left. - \cos\left(\frac{2\pi j x^\intercal w^\star}{M_{1,m}}\right)\sin\left(\frac{2\pi j' x^\intercal w^\star}{M_{1,m}}\right)\sin\left(\frac{2\pi j' T w_k^\star}{M_{1,m}}\right)\right) + \epsilon_d \tag{587}$$

We want to upper and lower bound this expression. To do so, we find it easier to work with integrals over x instead of these discrete sums. We can then bound the integrals, which we relegate to Section V C. To this end, we first need to bound the error from approximating our summation by an integral.

Claim 7 (Sum-to-integral error; Non-uniform case). *For $m \in \{1, \ldots, D\}$, consider parameters $M_{1,m}, M_{2,m}$ as defined above. Also consider a parameter R defined above. Then, for an integer $1 \leq j \leq D$,*

$$\frac{1}{G_d}\left|\int_{[-\tilde{R},\tilde{R}]^d} p_T^2(M_{1,m}x)\cos^2\left(2\pi j x^\intercal w^\star\right)\,dx - \frac{1}{M_{1,m}^d}\sum_{x_1,\ldots,x_d=-\tilde{R}M_{1,m}}^{\tilde{R}M_{1,m}-1} p_T^2(x)\cos^2\left(\frac{2\pi j x^\intercal w^\star}{M_{1,m}}\right)\right| \tag{588}$$

$$\leq \frac{6805}{6804}\frac{1}{21D^2}, \tag{589}$$

where

$$G_d \triangleq \prod_{i=1}^{d}\left(\int_{-\tilde{R}}^{+\tilde{R}} p_j^2(M_{1,m}x_j)\,dx_j\right). \tag{590}$$

Proof of Claim 7. The proof is similar to that of Claim 2, so we omit some details. As in Claim 2, we prove this by induction on the dimension d. Denoting $f(x) \triangleq \cos^2(2\pi j x^\intercal w^\star)$, we will prove

$$\frac{1}{G_d}\left|\int_{[-\tilde{R},\tilde{R}]^d} p_T^2(M_{1,m}x)f(x)\,dx - \frac{1}{M_{1,m}^d}\sum_{x_1,\ldots,x_d=-\tilde{R}M_{1,m}}^{\tilde{R}M_{1,m}-1} p_T^2(x)\,f\left(\frac{x}{M_{1,m}}\right)\right| \leq \frac{6805}{6804}\frac{10\pi d D R_w}{3M_{1,m}}. \tag{591}$$

Note that this implies our claim by our choice of $M_{1,m} = mM_1 \geq 70m\pi d^2D^3R_w \geq 70\pi dD^3R_w$. Thus, it suffices to prove Equation (591). In fact, we will use induction to prove that

$$\frac{1}{G_{d-1}}\left|\int_{[-\tilde{R},\tilde{R}]^{d-1}} p_T^2(M_{1,m}x)f(x,y)\,dx - \frac{1}{M_{1,m}^{d-1}}\sum_{x_1,\ldots,x_{d-1}=-\tilde{R}M_{1,m}}^{\tilde{R}M_{1,m}-1} p_T^2(x)\,f\left(\frac{x}{M_{1,m}},y\right)\right| \tag{592}$$

$$\leq \frac{6805}{6804}\frac{10\pi(d-1)DR_w}{3M_{1,m}} \tag{593}$$

for some fixed y. In the process, we show that Equation (591) follows from this. First, consider the base case. We want to prove

$$\frac{1}{G_1}\left|\int_{-\tilde{R}}^{+\tilde{R}} p(M_{1,m}x)p(M_{1,m}x+T)f(x)\,dx - \frac{1}{M_{1,m}}\sum_{x=-\tilde{R}M_{1,m}}^{\tilde{R}M_{1,m}-1} p(x)p(x+T)f\left(\frac{x}{M_{1,m}}\right)\,dx\right| \leq \frac{6805}{6804}\frac{10\pi DR_w}{3M_{1,m}} \tag{594}$$

and

$$\frac{1}{G_1}\left|\int_{-\tilde{R}}^{+\tilde{R}} p(M_{1,m}x)p(M_{1,m}x+T)f(x,y)\,dx - \frac{1}{M_{1,m}}\sum_{x=-\tilde{R}M_{1,m}}^{\tilde{R}M_{1,m}-1} p(x)p(x+T)f\left(\frac{x}{M_{1,m}},y\right)\,dx\right| \tag{595}$$

$$\leq \frac{6805}{6804}\frac{10\pi DR_w}{3M_{1,m}} \tag{596}$$

for some fixed y. First, for Equation (594), the error can be bounded by standard results in approximating integrals by Riemann sums:

$$\left|\int_{-\tilde{R}}^{+\tilde{R}} p(M_{1,m}x)p(M_{1,m}x+T)f(x)\,dx - \frac{1}{M_{1,m}}\sum_{x=-\tilde{R}M_{1,m}}^{\tilde{R}M_{1,m}-1} p(x)p(x+T)f\left(\frac{x}{M_{1,m}}\right)\,dx\right| \leq \frac{L\tilde{R}}{M_{1,m}}, \tag{597}$$

where $L \triangleq \max_{x\in[-\tilde{R},\tilde{R}]} |\tilde{f}'(x)|$ and $\tilde{f}(x) \triangleq p(M_{1,m}x)p(M_{1,m}x+T)f(x)$. By definition, $f(x) = \cos^2(2\pi jxw^\star)$, so

$$\tilde{f}'(x) = M_{1,m}p'(M_{1,m}x)p(M_{1,m}x+T)\cos^2(2\pi jxw^\star) + M_{1,m}p(M_{1,m}x)p'(M_{1,m}x+T)\cos^2(2\pi jxw^\star) \tag{598}$$

$$- 2p(M_{1,m}x)p(M_{1,m}x+T)\cos(2\pi jxw^\star)\sin(2\pi jxw^\star)\cdot 2\pi jw^\star. \tag{599}$$

Then,

$$|\tilde{f}'(x)| \leq M_{1,m}|p'(M_{1,m}x)| + M_{1,m}|p'(M_{1,m}x+T)| + 4\pi DR_w \leq 5\pi DR_w. \tag{600}$$

In the first inequality, we used Assumption 3 so that $p(x) \leq 1$. In the second inequality, we used Assumption 5, which bounds the derivative of p by $\pi DR_w/(2M_{1,m})$ since we chose our discretization parameter as $M_{1,m}$. Thus, we can conclude that

$$\left|\int_{-\tilde{R}}^{+\tilde{R}} p(M_{1,m}x)p(M_{1,m}x+T)f(x)\,dx - \frac{1}{M_{1,m}}\sum_{x=-\tilde{R}M_{1,m}}^{\tilde{R}M_{1,m}-1} p(x)p(x+T)f\left(\frac{x}{M_{1,m}}\right)\,dx\right| \leq \frac{5\pi DR_w\tilde{R}}{M_{1,m}}. \tag{601}$$

When dividing both sides by G_1, note that

$$G_1 = \int_{-\tilde{R}}^{+\tilde{R}} p^2(M_{1,m}z)\,dz \geq \frac{9\tilde{R}}{5} \geq \frac{5\tilde{R}}{3}, \tag{602}$$

where we used Assumption 2. In particular, by Assumption 2, we have

$$\int_{-\tilde{R}}^{+\tilde{R}} p^2(M_{1,m}z)\,dz - 2\tilde{R} = \int_{-\tilde{R}}^{+\tilde{R}} (p^2(M_{1,m}z) - 1)\,dz \geq -\frac{\tilde{R}}{5}. \tag{603}$$

This implies that $\int_{-\tilde{R}}^{+\tilde{R}} p^2(M_{1,m}z)\,dz \geq 2\tilde{R} - \tilde{R}/5 \geq 9\tilde{R}/5 \geq 5\tilde{R}/3$. Note that Assumption 2 applies here because the truncation parameter is $R = \tilde{R}M_{1,m}$ so that $M_{1,m}z \in [-\tilde{R}M_{1,m}, \tilde{R}M_{1,m}] = [-R, R]$. Thus, we have that $\tilde{R}/G_1 \leq 3/5$ so that

$$\frac{1}{G_1}\left|\int_{-\tilde{R}}^{+\tilde{R}} p(M_{1,m}x)p(M_{1,m}x+T)f(x)\,dx - \frac{1}{M_{1,m}}\sum_{x=-\tilde{R}M_{1,m}}^{\tilde{R}M_{1,m}-1} p(x)p(x+T)f\left(\frac{x}{M_{1,m}}\right)\,dx\right| \tag{604}$$

$$\leq \frac{3\pi DR_w}{M_{1,m}} \tag{605}$$

$$\leq \frac{6805}{6804}\frac{10\pi D R_w}{3M_{1,m}}. \tag{606}$$

The proof of Equation (595) follows similarly.

Now, for the inductive step, suppose for ℓ such that $d-1 \geq \ell \geq 1$ that

$$\frac{1}{G_\ell}\left|\int_{[-\tilde{R},\tilde{R}]^\ell} p_T^2(M_{1,m}x)f(x,y)\,dx - \frac{1}{M_{1,m}^\ell}\sum_{x_1,\ldots,x_\ell=-\tilde{R}M_{1,m}}^{\tilde{R}M_{1,m}-1} p_T^2(x)f\left(\frac{x}{M_{1,m}},y\right)\right| \leq \frac{6805}{6804}\frac{10\pi\ell D R_w}{3M_{1,m}} \tag{607}$$

for some fixed y and where $f(x_1,\ldots,x_\ell,y) = \cos^2(2\pi j(x_1 w_1^\star + \cdots + x_\ell w_\ell^\star + y w_{\ell+1}^\star))$. We first show that Equation (591) holds for $\ell+1$. Suppose that $\ell+1 > k$ for now.

$$\frac{1}{G_{\ell+1}}\int_{[-\tilde{R},\tilde{R}]^{\ell+1}} p_T^2(M_{1,m}x)f(x)\,dx \tag{608}$$

$$= \frac{1}{\int_{-\tilde{R}}^{+\tilde{R}} p_{\ell+1}^2(M_{1,m}z)\,dz}\int_{-\tilde{R}}^{+\tilde{R}}\left(\frac{1}{G_\ell}\int_{[-\tilde{R},\tilde{R}]^\ell} p_T^2(M_{1,m}x_1,\ldots,M_{1,m}x_\ell)f(x_1,\ldots,x_{\ell+1})\,dx_1\cdots dx_\ell\right) \cdot p_{\ell+1}^2(M_{1,m}x_{\ell+1})\,dx_{\ell+1} \tag{609}$$

$$\leq \frac{1}{M_{1,m}^\ell G_{\ell+1}}\sum_{x_1,\ldots,x_\ell=-\tilde{R}M_{1,m}}^{\tilde{R}M_{1,m}-1}\int_{-\tilde{R}}^{+\tilde{R}} p_T^2\left(x_1,\ldots,x_\ell,M_{1,m}x_{\ell+1}\right) f\left(\frac{x_1}{M_{1,m}},\ldots,\frac{x_\ell}{M_{1,m}},x_{\ell+1}\right)dx_{\ell+1} \tag{610}$$

$$+ \frac{1}{\int_{-\tilde{R}}^{+\tilde{R}} p_{\ell+1}^2(M_{1,m}z)\,dz}\int_{-\tilde{R}}^{+\tilde{R}} p_{\ell+1}^2(M_{1,m}x_{\ell+1})\frac{6805}{6804}\frac{10\pi\ell D R_w}{3M_{1,m}}\,dx_{\ell+1} \tag{611}$$

$$= \frac{1}{M_{1,m}^\ell G_{\ell+1}}\sum_{x_1,\ldots,x_\ell=-\tilde{R}M_{1,m}}^{\tilde{R}M_{1,m}-1} p_T^2\left(x_1,\ldots,x_\ell\right)\int_{-\tilde{R}}^{+\tilde{R}} p_{\ell+1}^2(M_{1,m}x_{\ell+1})f\left(\frac{x_1}{M_{1,m}},\ldots,\frac{x_\ell}{M_{1,m}},x_{\ell+1}\right)dx_{\ell+1} \tag{612}$$

$$+ \frac{6805}{6804}\frac{10\pi\ell D R_w}{3M_{1,m}}. \tag{613}$$

In the inequality, we use the inductive hypothesis. In the last equality, we rearrange and simplify. Now, we can approximate this last integral by a Riemann sum for the function $\tilde{f}(y) \triangleq p_{\ell+1}^2(M_{1,m}y)f(x_1/M_{1,m},\ldots,x_\ell/M_{1,m},y)$, with error bounded similarly to Equation (597):

$$\frac{1}{G_{\ell+1}}\int_{[-\tilde{R},\tilde{R}]^{\ell+1}} p_T^2(M_{1,m}x)f(x)\,dx \tag{614}$$

$$\leq \frac{1}{M_{1,m}^\ell G_{\ell+1}}\sum_{x_1,\ldots,x_\ell=-\tilde{R}M_{1,m}}^{\tilde{R}M_{1,m}-1} p_T^2\left(x_1,\ldots,x_\ell\right)\left(\frac{1}{M_{1,m}}\sum_{x_{\ell+1}=-\tilde{R}M_{1,m}}^{\tilde{R}M_{1,m}-1} p_{\ell+1}^2(x_{\ell+1})f\left(\frac{x}{M_{1,m}}\right) + \frac{L'\tilde{R}}{M_{1,m}}\right) \tag{615}$$

$$+ \frac{6805}{6804}\frac{10\pi\ell D R_w}{3M_{1,m}}. \tag{616}$$

Here, $L' \triangleq \max_{y\in[-\tilde{R},\tilde{R}]}|\tilde{f}'(y)|$. Since $f(x) = \cos^2(2\pi j x^\intercal w^\star)$, then

$$\tilde{f}'(y) = 2M_{1,m}p_{\ell+1}(M_{1,m}y)p_{\ell+1}'(M_{1,m}y)\cos^2\left(2\pi j\left(y w_{\ell+1}^\star + \sum_{i=1}^{\ell}\frac{x_i w_i^\star}{M_{1,m}}\right)\right) \tag{617}$$

$$- 2p_{\ell+1}^2(M_{1,m}y)\cos\left(2\pi j\left(y w_{\ell+1}^\star + \sum_{i=1}^{\ell}\frac{x_i w_i^\star}{M_{1,m}}\right)\right)\sin\left(2\pi j\left(y w_{\ell+1}^\star + \sum_{i=1}^{\ell}\frac{x_i w_i^\star}{M_{1,m}}\right)\right)\cdot 2\pi j w_{\ell+1}^\star. \tag{618}$$

Thus, for $y \in [-\tilde{R},\tilde{R}]$, then

$$|\tilde{f}'(y)| \leq 2M_{1,m}|p_{\ell+1}'(M_{1,m}y)| + 4\pi D R_w \leq 5\pi D R_w. \tag{619}$$

In the first inequality, we use Assumption 3 so that $p(z) \leq 1$ and $j \leq D$. In the second inequality, we use Assumption 5 so that $|p'_{\ell+1}(M_{1,m}y)| \leq \pi D R_w/(2M_{1,m})$ since we used discretization parameter $M_{1,m}$. Also, note that this applies because we chose our truncation parameter as $R = \tilde{R}M_{1,m}$ so that $M_{1,m}y \in [-\tilde{R}M_{1,m}, \tilde{R}M_{1,m}] = [-R, R]$. Then, $L' \leq 5\pi D R_w$. Plugging this back in,

$$\frac{1}{G_{\ell+1}} \int_{[-\tilde{R},\tilde{R}]^{\ell+1}} p_T^2(M_{1,m}x) f(x)\, dx \leq \frac{1}{M_{1,m}^{\ell+1} G_{\ell+1}} \sum_{x_1,\ldots,x_{\ell+1}=-\tilde{R}M_{1,m}}^{\tilde{R}M_{1,m}-1} p_T^2(x) f\left(\frac{x}{M_{1,m}}\right) \tag{620}$$

$$+ \frac{5\pi D R_w}{M_{1,m}} \frac{\tilde{R}}{\int_{-\tilde{R}}^{+\tilde{R}} p_{\ell+1}^2(M_{1,m}z)\, dz} \frac{1}{M_{1,m}^{\ell} G_\ell} \sum_{x_1,\ldots,x_\ell=-\tilde{R}M_{1,m}}^{\tilde{R}M_{1,m}-1} p_T^2(x) \tag{621}$$

$$+ \frac{6805}{6804} \frac{10\pi\ell D R_w}{3M_{1,m}}. \tag{622}$$

We previously showed that $\tilde{R}/G_1 \leq 3/5$ (see around Equation (602)). By the same argument here, then we can bound

$$\frac{1}{G_{\ell+1}} \int_{[-\tilde{R},\tilde{R}]^{\ell+1}} p_T^2(M_{1,m}x) f(x)\, dx \leq \frac{1}{M_{1,m}^{\ell+1} G_{\ell+1}} \sum_{x_1,\ldots,x_{\ell+1}=-\tilde{R}M_{1,m}}^{\tilde{R}M_{1,m}-1} p_T^2(x) f\left(\frac{x}{M_{1,m}}\right) \tag{623}$$

$$+ \frac{3\pi D R_w}{M_{1,m}} \frac{1}{M_{1,m}^{\ell} G_\ell} \sum_{x_1,\ldots,x_\ell=-\tilde{R}M_{1,m}}^{\tilde{R}M_{1,m}-1} p_T^2(x) + \frac{6805}{6804} \frac{10\pi\ell D R_w}{3M_{1,m}}. \tag{624}$$

Thus, it is clear to that to complete our argument, we need to show that

$$\frac{3\pi D R_w}{M_{1,m}} \frac{1}{M_{1,m}^{\ell} G_\ell} \sum_{x_1,\ldots,x_\ell=-\tilde{R}M_{1,m}}^{\tilde{R}M_{1,m}-1} p_T^2(x) \leq \frac{6805}{6804} \frac{10\pi D R_w}{3M_{1,m}}. \tag{625}$$

To see this, first note that

$$M_{1,m}^{\ell} G_\ell \geq M_{1,m}^{\ell} \prod_{i=1}^{\ell} \left(\frac{9\tilde{R}}{5}\right) = \left(\frac{9}{5} M_{1,m}\tilde{R}\right)^{\ell} \geq \left(\frac{9}{5} \cdot 54 \cdot 70\right)^{\ell} \geq 6804. \tag{626}$$

In the first inequality, we use the same argument as Equation (602), which relies on Assumption 2. In the second inequality, we use that $M_{1,m} \geq M_1 \geq 70\pi d^2 D^3 R_w \geq 70\pi R_w$ and $\tilde{R} \geq 54D^2\sqrt{d}/(\pi R_w) \geq 54/(\pi R_w)$. In the last inequality, we use that $\ell \geq 1$ and simplify.

Suppose for now that p^2 has at most one critical point at $a \in (-\tilde{R}M_{1,m}, \tilde{R}M_{1,m})^{\ell}$. Without loss of generality, since p^2 is even by Assumption 4, then we can assume that the critical point occurs at $a = 0$. Also suppose without loss of generality that p^2 is nondecreasing for $x \leq 0$ and nonincreasing for $x \geq 0$. The argument is the same for other cases. By the above argument, we have

$$\frac{1}{M_{1,m}^{\ell} G_\ell} = \frac{M_{1,m}^{\ell} G_\ell + 1}{M_{1,m}^{\ell} G_\ell} \cdot \frac{1}{M_{1,m}^{\ell} G_\ell + 1} \leq \frac{6805}{6804} \frac{1}{M_{1,m}^{\ell} G_\ell + 1}. \tag{627}$$

Moreover, by standard results bounding sums in terms of integrals for monotone functions,

$$\tilde{G}_\ell = \sum_{x_1,\ldots,x_\ell=-\tilde{R}M_{1,m}}^{\tilde{R}M_{1,m}-1} p_1^2(x_1)\cdots p_\ell^2(x_\ell) \tag{628}$$

$$\leq \sum_{x_1,\ldots,x_\ell=-\tilde{R}M_{1,m}}^{-1} p_1^2(x_1)\cdots p_\ell^2(x_\ell) + \sum_{x_1,\ldots,x_\ell=1}^{\tilde{R}M_{1,m}} p_1^2(x_1)\cdots p_\ell^2(x_\ell) + 1 \tag{629}$$

$$\leq \int_{[-\tilde{R}M_{1,m},0]^{\ell}} p_1^2(x_1)\cdots p_\ell^2(x_\ell)\, dx + \int_{[0,\tilde{R}M_{1,m}]^{d}} p_1^2(x_1)\cdots p_\ell^2(x_\ell)\, dx + 1 \tag{630}$$

$$= \prod_{i=1}^{\ell}\left(\int_{-\tilde{R}M_{1,m}}^{+\tilde{R}M_{1,m}} p_i^2(x_i)\,dx_i\right) + 1 \tag{631}$$

$$= M_{1,m}^{\ell} G_\ell + 1, \tag{632}$$

where in the second line, we use Assumption 3 that $p_j \leq 1$. In the last line, we use a change of variables. Combining this with the above, we have

$$\frac{1}{M_{1,m}^{\ell} G_\ell} \leq \frac{6805}{6804}\frac{1}{M_{1,m}^{\ell} G_\ell + 1} \leq \frac{6805}{6804}\frac{1}{\tilde{G}_\ell}. \tag{633}$$

Earlier, we considered the case when p^2 has at most one critical point. If we instead consider p^2 with a constant number of critical points, as in Assumption 6, the above argument only changes the constant factor 6805/6804. We carry the factor of 6805/6804 through the analysis, but changing this only affects some of the constants in the overall verification procedure and not the sample complexity.

Putting everything together,

$$\frac{3\pi D R_w}{M_{1,m}}\frac{1}{M_{1,m}^{\ell} G_\ell}\sum_{x_1,\ldots,x_\ell=-\tilde{R}M_{1,m}}^{\tilde{R}M_{1,m}-1} p_T^2(x) \leq \frac{6805}{6804}\frac{3\pi D R_w}{M_{1,m}}\frac{1}{\tilde{G}_\ell}\sum_{x_1,\ldots,x_\ell=-\tilde{R}M_{1,m}}^{\tilde{R}M_{1,m}-1} p_T^2(x) \tag{634}$$

$$= \frac{6805}{6804}\frac{3\pi D R_w}{M_{1,m}}\frac{1}{\sum_{x_k=-\tilde{R}M_{1,m}}^{\tilde{R}M_{1,m}-1} p_k^2(x_k)}\sum_{x_k=-\tilde{R}M_{1,m}}^{\tilde{R}M_{1,m}-1} p_k(x_k)p_k(x_k+T) \tag{635}$$

$$\leq \frac{6805}{6804}\frac{3\pi D R_w}{M_{1,m}}\frac{1}{\sum_{x_k=-\tilde{R}M_{1,m}}^{\tilde{R}M_{1,m}-1} p_k^2(x_k)}(2\tilde{R}M_{1,m}) \tag{636}$$

$$\leq \frac{6805}{6804}\frac{3\pi D R_w}{M_{1,m}}\frac{5}{9\tilde{R}M_{1,m}} \cdot 2\tilde{R}M_{1,m} \tag{637}$$

$$= \frac{6805}{6804}\frac{10\pi D R_w}{3M_{1,m}}, \tag{638}$$

as required. In the first line, we use Equation (633). In the third line, we use Assumption 3 that $p_k \leq 1$. In the fourth line, we use Assumption 2. In particular, by Assumption 2, we have

$$\sum_{x_k=-\tilde{R}M_{1,m}}^{\tilde{R}M_{1,m}-1} p_k^2(x_k) - 2\tilde{R}M_{1,m} = \sum_{x_k=-\tilde{R}M_{1,m}}^{\tilde{R}M_{1,m}-1} (p_k^2(x_k) - 1) \geq -\frac{\tilde{R}}{5}. \tag{639}$$

Thus, this implies that $\sum_{x_k=-\tilde{R}M_{1,m}}^{\tilde{R}M_{1,m}-1} p_k^2(x_k) \geq 2\tilde{R} - \tilde{R}/5 = 9\tilde{R}/5$. Note that we assumed throughout this analysis that $\ell + 1 > k$. If $\ell + 1 = k$, the only part affected is when we bound L', which would instead be a bound on the derivative of $\tilde{f}(y) = p_{\ell+1}(M_{1,m}y)p_{\ell+1}(M_{1,m}y + T)f(x_1/M_{1,m}, \ldots, x_\ell/M_{1,m}, y)$. The derivative of $\tilde{f}(y)$ now has a term depending on $|T|$, which can be bounded again using Assumption 5, as we did in the base case, resulting in the same bound $L' \leq 5\pi D R_w$. One can do the same argument for the lower bound, so this concludes the proof that Equation (591) holds for $\ell + 1$.

To complete the induction, one should also show that Equation (592) holds for $\ell + 1$. This follows by the same argument as above, and we refer to Claim 2 for a sketch of how the argument is modified. This completes the proof. □

The same result can be shown for the cross terms $\cos(2\pi j x^\intercal w^\star/M_{1,m})\cos(2\pi j' x^\intercal w^\star)$ and $\cos(2\pi j x^\intercal w^\star/M_{1,m})\sin(2\pi j' x^\intercal w^\star/M_{1,m})$ by the same argument. This is clear because these terms have the same bound on their gradients.

We can also bound the discretization error ϵ_d. Note that this discretization error is defined as

$$\epsilon_d \triangleq \frac{1}{\tilde{G}_d M_{2,m}^2}\sum_{x_1,\ldots,x_d=-\tilde{R}M_{1,m}}^{\tilde{R}M_{1,m}-1}\sum_{j,j'=1}^{D} p_T^2(x)\,\beta_j^\star\beta_{j'}^\star\left(\cos\left(\frac{2\pi j x^\intercal w^\star}{M_{1,m}}\right)\cos\left(\frac{2\pi j'(x+Te_k)^\intercal w^\star}{M_{1,m}}\right)\right. \tag{640}$$

$$\left. - \left\lfloor\cos\left(\frac{2\pi j x^\intercal w^\star}{M_{1,m}}\right)\right\rfloor_{M_{2,m}}\left\lfloor\cos\left(\frac{2\pi j'(x+Te_k)^\intercal w^\star}{M_{1,m}}\right)\right\rfloor_{M_{2,m}}\right). \tag{641}$$

Claim 8 (Discretization error; Non-uniform case)**.** *For $m \in \{1, \ldots, D\}$, consider parameters $M_{1,m}, M_{2,m}$ as defined above. Also, consider a parameter $\tilde{R}$ defined above. Then, we can bound the discretization error ϵ_d defined in Equation (640) as*

$$|\epsilon_d| \leq \frac{3}{M_{2,m}^3}. \tag{642}$$

Proof of Claim 8. This follows by a simple calculation and is similar to Claim 3. Following the same steps as the proof of Claim 3, we can arrive at

$$|\epsilon_d| \leq \frac{1}{\tilde{G}_d M_{2,m}^2} \sum_{x_1,\ldots,x_d=-\tilde{R}M_{1,m}}^{\tilde{R}M_{1,m}-1} \sum_{j,j'=1}^{D} p_T^2\left(x\right) |\beta_j^\star||\beta_{j'}^\star| \frac{2}{M_{2,m}} \tag{643}$$

$$= \frac{2}{\tilde{G}_d M_{2,m}^3} \sum_{x_1,\ldots,x_d=-\tilde{R}M_{1,m}}^{\tilde{R}M_{1,m}-1} p_T^2\left(x\right), \tag{644}$$

where in the second line, we use $\left\|\beta^\star\right\|_1 = 1$. We can simplify this further using the definition of $p_T^2(x)$:

$$|\epsilon_d| \leq \frac{2}{M_{2,m}^3} \frac{1}{\tilde{G}_d} \left(\sum_{x_k=-\tilde{R}M_{1,m}}^{\tilde{R}M_{1,m}-1} p_k(x_k) p_k(x_k+T) \right) \prod_{\substack{i=1\\ i\neq k}}^{d} \left(\sum_{x_i=-\tilde{R}M_{1,m}}^{\tilde{R}M_{1,m}-1} p_i^2(x_i) \right) \tag{645}$$

$$= \frac{2}{M_{2,m}^3} \frac{1}{\sum_{x_k=-\tilde{R}M_{1,m}}^{\tilde{R}M_{1,m}-1} p_k^2(x_k)} \sum_{x_k=-\tilde{R}M_{1,m}}^{\tilde{R}M_{1,m}-1} p_k(x_k) p_k(x_k+T) \tag{646}$$

$$\leq \frac{2}{M_{2,m}^3} \frac{1}{\sum_{x_k=-\tilde{R}M_{1,m}}^{\tilde{R}M_{1,m}-1} p_k^2(x_k)} (2\tilde{R}M_{1,m}) \tag{647}$$

$$\leq \frac{2}{M_{2,m}^3} \frac{5}{9\tilde{R}M_{1,m}} \cdot 2\tilde{R}M_{1,m} \tag{648}$$

$$\leq \frac{3}{M_{2,m}^3}. \tag{649}$$

In the third line, we use Assumption 3 that $p_k \leq 1$. In the fourth line, we use Assumption 2. In particular, by Assumption 2, we have

$$\sum_{x_k=-\tilde{R}M_{1,m}}^{\tilde{R}M_{1,m}-1} p_k^2(x_k) - 2\tilde{R}M_{1,m} = \sum_{x_k=-\tilde{R}M_{1,m}}^{\tilde{R}M_{1,m}-1} (p_k^2(x_k) - 1) \geq -\frac{\tilde{R}}{5}. \tag{650}$$

Thus, this implies that $\sum_{x_k=-\tilde{R}M_{1,m}}^{\tilde{R}M_{1,m}-1} p_k^2(x_k) \geq 2\tilde{R} - \tilde{R}/5 = 9\tilde{R}/5$. □

With this, we can finally move on to show that the conditions checked in Steps 3 and 4 of Algorithm 4 are satisfied if and only if $|T - \ell M_1/w_k^\star| \leq 1$. To do so, we use Claim 7 and Claim 8 in Equations (586) and (587) and leverage integral bounds from Section V C. The following two claims show this for each direction of the if and only if.

Claim 9 (Correctness of Step 3 in Algorithm 4)**.** *Consider parameters M_1, M_2, R defined above and the observable $O_{k,1}$ defined in Equation (142). Let α_1 denote the result of querying the QSQ oracle with observable $O_{k,1}$ with discretization parameters M_1, M_2, truncation parameter $R = \tilde{R}M_1$, and tolerance $\tau \leq \frac{1}{M_2^2}\left(\frac{5}{42D} - \frac{3}{2M_2}\right)$. If $|T - \ell M_1/w_k^\star| \leq 1$ for some integer ℓ, then*

$$\alpha_1 \geq \frac{1}{M_2^2}\left(\frac{5}{14D} - \frac{9}{2M_2}\right). \tag{651}$$

Claim 10 (Correctness of Step 4 in Algorithm 4)**.** *For $m \in \{1, \ldots, D\}$, consider parameters $M_{1,m}, M_{2,m}, R$ defined above and the observables $O_{k,m}$ defined in Equation (142). Let α_m denote the result of querying the QSQ oracle with observable $O_{k,m}$ with discretization parameters $M_{1,m}, M_{2,m}$, truncation parameter*

$R = \tilde{R}M_{1,m}$, and tolerance $\tau \leq \frac{1}{2D^2M_2^2}\left(\frac{2}{9} - \frac{1}{8}\left(\frac{2\pi R_w}{M_1}\right)^2 + \frac{3D^2}{M_2}\right)$. If $|T - \ell M_1/w_k^\star|$ is not less than 1 for any integer ℓ, then

$$\sum_{m=1}^{D} \alpha_m \leq \frac{1}{M_2^2}\left(\frac{13}{25}D + \frac{1}{2D}\left(\frac{2}{9} - \frac{1}{8}\left(\frac{2\pi R_w}{M_1}\right)^2 + \frac{3D^2}{M_2}\right)\right). \tag{652}$$

It suffices to prove these two claims to finish the proof. Our starting point for both proofs is Equations (586) and (587).

Proof of Claim 9. We want to lower bound $\langle h_{w^\star,M_1,M_2}|O_{k,1}|h_{w^\star,M_1,M_2}\rangle$. As in Equation (633), one can show that $\tilde{G}_d \leq M_1^d G_d + 1$. Recall that this uses Assumption 6. Using this along with Equation (626), we have

$$\frac{1}{\tilde{G}_d} \geq \frac{1}{M_1^d G_d + 1} = \frac{M_1^d G_d}{M_1^d G_d + 1}\frac{1}{M_1^d G_d} \geq \frac{6804}{6805}\frac{1}{M_1^d G_d}. \tag{653}$$

Plugging this into Equations (586) and (587), we have

$$\begin{aligned}
&\langle h_{w^\star,M_1,M_2}|O_{k,1}|h_{w^\star,M_1,M_2}\rangle && (654)\\
&\geq \frac{6804}{6805}\frac{1}{M_2^2}\frac{1}{M_1^d G_d}\sum_{x_1,\ldots,x_d=-\tilde{R}M_1}^{\tilde{R}M_1-1}\sum_{j=1}^{D} p_T^2(x)(\beta_j^\star)^2\left(\cos^2\left(\frac{2\pi j x^\intercal w^\star}{M_1}\right)\cos\left(\frac{2\pi j T w_k^\star}{M_1}\right)\right. && (655)\\
&\qquad\left. - \cos\left(\frac{2\pi j x^\intercal w^\star}{M_1}\right)\sin\left(\frac{2\pi j x^\intercal w^\star}{M_1}\right)\sin\left(\frac{2\pi j T w_k^\star}{M_1}\right)\right)\\
&+\frac{6804}{6805}\frac{1}{M_2^2}\frac{1}{M_1^d G_d}\sum_{x_1,\ldots,x_d=-\tilde{R}M_1}^{\tilde{R}M_1-1}\sum_{\substack{j,j'=1\\ j\neq j'}}^{D} p_T^2(x)\,\beta_j^\star\beta_{j'}^\star\left(\cos\left(\frac{2\pi j x^\intercal w^\star}{M_1}\right)\cos\left(\frac{2\pi j' x^\intercal w^\star}{M_1}\right)\cos\left(\frac{2\pi j' T w_k^\star}{M_1}\right)\right.\\
&\qquad\left. - \cos\left(\frac{2\pi j x^\intercal w^\star}{M_1}\right)\sin\left(\frac{2\pi j' x^\intercal w^\star}{M_1}\right)\sin\left(\frac{2\pi j' T w_k^\star}{M_1}\right)\right) + \epsilon_d. && (656)
\end{aligned}$$

Applying Claim 7, then

$$\begin{aligned}
&\langle h_{w^\star,M_1,M_2}|O_{k,1}|h_{w^\star,M_1,M_2}\rangle && (657)\\
&\geq \frac{6804}{6805}\frac{1}{M_2^2}\frac{1}{G_d}\sum_{j=1}^{D}(\beta_j^\star)^2\int_{[-\tilde{R},\tilde{R}]^d} p_T^2(M_{1,m}x)\left(\cos^2(2\pi j x^\intercal w^\star)\cos\left(\frac{2\pi j T w_k^\star}{M_1}\right)\right. && (658)\\
&\qquad\left. - \cos(2\pi j x^\intercal w^\star)\sin(2\pi j x^\intercal w^\star)\sin\left(\frac{2\pi j T w_k^\star}{M_1}\right)\right) dx\\
&+\frac{6804}{6805}\frac{1}{M_2^2}\frac{1}{G_d}\sum_{\substack{j,j'=1\\ j\neq j'}}^{D}\beta_j^\star\beta_{j'}^\star\int_{[-\tilde{R},\tilde{R}]^d} p_T^2(M_{1,m}x)\left(\cos(2\pi j x^\intercal w^\star)\cos(2\pi j' x^\intercal w^\star)\cos\left(\frac{2\pi j' T w_k^\star}{M_1}\right)\right.\\
&\qquad\left. - \cos(2\pi j x^\intercal w^\star)\sin(2\pi j' x^\intercal w^\star)\sin\left(\frac{2\pi j' T w_k^\star}{M_1}\right)\right) + \epsilon_d + \frac{6804}{6805}\frac{4}{M_2^2}\epsilon_{\text{int}}. && (659)
\end{aligned}$$

We can simplify this using the fact that an integral of an odd function, e.g., $\sin(x)\cos(x)$, over an even interval is zero. This also uses Assumption 4 that p^2 is an even function.

$$\begin{aligned}
&\langle h_{w^\star,M_1,M_2}|O_{k,1}|h_{w^\star,M_1,M_2}\rangle && (660)\\
&\geq \frac{6804}{6805}\frac{1}{M_2^2}\sum_{j=1}^{D}(\beta_j^\star)^2\cos\left(\frac{2\pi j T w_k^\star}{M_1}\right)\frac{1}{G_d}\int_{[-\tilde{R},\tilde{R}]^d} p_T^2(M_{1,m}x)\left(\cos^2(2\pi j x^\intercal w^\star)\right) dx + \epsilon_d + \frac{6804}{6805}\frac{4}{M_2^2}\epsilon_{\text{int}} && (661)
\end{aligned}$$

$$+\frac{6804}{6805}\frac{1}{M_2^2}\sum_{\substack{j,j'=1\\j\neq j'}}^{D}\beta_j^\star\beta_{j'}^\star\left(\cos\left(\frac{2\pi j' T w_k^\star}{M_1}\right)\frac{1}{G_d}\int_{[-\tilde{R},\tilde{R}]^d}p_T^2(M_{1,m}x)\cos(2\pi j x^\intercal w^\star)\cos(2\pi j' x^\intercal w^\star)\,dx\right.$$
$$\left.-\sin\left(\frac{2\pi j' T w_k^\star}{M_1}\right)\frac{1}{G_d}\int_{[-\tilde{R},\tilde{R}]^d}p_T^2(M_{1,m}x)\cos(2\pi j x^\intercal w^\star)\sin(2\pi j' x^\intercal w^\star)\,dx\right). \tag{662}$$

Using Corollaries 14, 16 and 17,

$$\langle h_{w^\star,M_1,M_2}|O_{k,1}|h_{w^\star,M_1,M_2}\rangle \geq \frac{6804}{6805}\frac{1}{M_2^2}\sum_{j=1}^{D}(\beta_j^\star)^2\left(\frac{1}{2}-\frac{3\sqrt{d}}{16\pi R_w\tilde{R}}\right)\cos\left(\frac{2\pi j T w_k^\star}{M_1}\right) \tag{663}$$
$$-\frac{6804}{6805}\frac{1}{M_2^2}\sum_{\substack{j,j'=1\\j\neq j'}}^{D}\beta_j^\star\beta_{j'}^\star\left(\frac{3\sqrt{d}}{2\pi R_w\tilde{R}}\right)+\epsilon_d+\frac{6804}{6805}\frac{4}{M_2^2}\epsilon_{\text{int}}. \tag{664}$$

Note that Corollaries 14, 16 and 17 apply when integrating with respect to the non-uniform density, which we don't quite have here. However, using Corollary 13 instead of Corollary 12 in their proofs, we see that the results still hold for integrating with respect to p_T^2. Using our choice of $\tilde{R} \geq \max(39\sqrt{d}/(4\pi R_w), 54D^2\sqrt{d}/(\pi R_w))$, we have

$$\langle h_{w^\star,M_1,M_2}|O_{k,1}|h_{w^\star,M_1,M_2}\rangle \geq \frac{1}{M_2^2}\left(\frac{6804}{6805}\frac{25}{52}\sum_{j=1}^{D}(\beta_j^\star)^2\cos\left(\frac{2\pi j T w_k^\star}{M_1}\right)-\frac{6804}{6805}\frac{1}{36D^2}-\frac{3}{M_2}-\frac{4}{21D^2}\right) \tag{665}$$

We also use that $\|\beta^\star\|_2^2 \leq 1$ since $\|\beta^\star\|_1 = 1$, $|\epsilon_d| \leq 3/M_2^3$ by Claim 8, and $|\epsilon_{\text{int}}| \leq 6805/(21\cdot 6804D^2)$ by Claim 7. We can lower bound the summation term by Equation (207) to obtain

$$\langle h_{w^\star,M_1,M_2}|O_{k,1}|h_{w^\star,M_1,M_2}\rangle \geq \frac{1}{M_2^2}\left(\frac{6804}{6805}\frac{25}{52}\frac{2449}{2550D}-\frac{6879}{6880}\frac{1}{36D^2}-\frac{3}{M_2}-\frac{4}{21D^2}\right) \tag{666}$$
$$\geq \frac{1}{M_2^2}\left(\frac{5}{21D}-\frac{3}{M_2}\right). \tag{667}$$

Thus, we see that if $|T-\ell M_1/w_k^\star| \leq 1$, then this lower bound on the expectation value must be satisfied. Finally, our choice of τ and the condition on α_1 guarantees that this the lower bound on the expectation value also holds, as required. □

Proof of Claim 10. This time, we want to upper bound $\langle h_{w^\star,M_{1,m},M_{2,m}}|O_{k,m}|h_{w^\star,M_{1,m},M_{2,m}}\rangle$ for any $m \in \{1,\ldots,D\}$. Similarly to Equation (633), we can show that $\tilde{G}_d \geq M_{1,m}^d G_d$.

Suppose for now that p^2 has at most one critical point at $a \in (-\tilde{R}M_{1,m}, \tilde{R}M_{1,m})^d$. Without loss of generality, since p^2 is even by Assumption 4, then we can assume that the critical point occurs at $a = 0$. Also, suppose without loss of generality that p^2 is nondecreasing for $x \leq 0$ and nonincreasing for $x \geq 0$. The argument is the same for other cases. By Equation (626), we have

$$\frac{1}{M_{1,m}^d G_d} = \frac{M_{1,m}^d G_d - 1}{M_{1,m}^d G_d}\cdot\frac{1}{M_{1,m}^d G_d - 1} \geq \frac{6803}{6804}\frac{1}{M_{1,m}^d G_d - 1}. \tag{668}$$

Moreover, by standard results bounding sums in terms of integrals for monotone functions, we have

$$\tilde{G}_d = \sum_{x_1,\ldots,x_d=-\tilde{R}M_{1,m}}^{\tilde{R}M_{1,m}-1} p_1^2(x_1)\cdots p_d^2(x_d) \tag{669}$$
$$= \sum_{x_1,\ldots,x_d=-\tilde{R}M_{1,m}}^{0} p_1^2(x_1)\cdots p_d^2(x_d) + \sum_{x_1,\ldots,x_d=0}^{\tilde{R}M_{1,m}-1} p_1^2(x_1)\cdots p_d^2(x_d) - p^2(0) \tag{670}$$
$$\geq \int_{[-\tilde{R}M_{1,m}-1,0]^d} p^2(x)\,dx + \int_{[0,\tilde{R}M_{1,m}]^d} p^2(x)\,dx - 1 \tag{671}$$

$$\geq \int_{[-\tilde{R}M_{1,m},\tilde{R}M_{1,m}]^d} p^2(x)\,dx - 1 \tag{672}$$

$$= M_{1,m}^d G_d - 1. \tag{673}$$

In the third line, we use Assumption 3 tahat $p_j^2 \leq 1$. In the last line, we use a change of variables. Combining this with the above, we have

$$\frac{1}{M_{1,m}^d G_d} \geq \frac{6803}{6804}\frac{1}{M_{1,m}^d G_d - 1} \geq \frac{6803}{6804}\frac{1}{\tilde{G}_d}. \tag{674}$$

Earlier, we considered the case when p^2 has at most one critical point. If we instead consider p^2 with a constant number of critical points, as in Assumption 6, the above argument only changes the constant factor 6803/6804. We carry the factor of 6803/6804 through the analysis, but changing this only affects some of the constants in the overall verification procedure and not the sample complexity.

Using this along with Claim 7, plugging into Equations (586) and (587), we have

$$\langle h_{w^\star,M_{1,m},M_{2,m}} | O_{k,m} | h_{w^\star,M_{1,m},M_{2,m}} \rangle \tag{675}$$

$$\leq \frac{6804}{6803}\frac{1}{M_{2,m}^2}\frac{1}{G_d}\sum_{j=1}^D (\beta_j^\star)^2 \int_{[-\tilde{R},\tilde{R}]^d} p_T^2(M_{1,m}x)\left(\cos^2\left(2\pi j x^\intercal w^\star\right)\cos\left(\frac{2\pi j T w_k^\star}{M_{1,m}}\right)\right.$$
$$\left. - \cos\left(2\pi j x^\intercal w^\star\right)\sin\left(2\pi j x^\intercal w^\star\right)\sin\left(\frac{2\pi j T w_k^\star}{M_{1,m}}\right)\right) dx \tag{676}$$

$$+\frac{6804}{6803}\frac{1}{M_{2,m}^2}\frac{1}{G_d}\sum_{\substack{j,j'=1\\ j\neq j'}}^D \beta_j^\star\beta_{j'}^\star \int_{[-\tilde{R},\tilde{R}]^d} p_T^2\left(M_{1,m}x\right)\left(\cos\left(2\pi j x^\intercal w^\star\right)\cos\left(2\pi j' x^\intercal w^\star\right)\cos\left(\frac{2\pi j' T w_k^\star}{M_{1,m}}\right)\right.$$
$$\left. - \cos\left(2\pi j x^\intercal w^\star\right)\sin\left(2\pi j' x^\intercal w^\star\right)\sin\left(\frac{2\pi j' T w_k^\star}{M_{1,m}}\right)\right) + \epsilon_d + \frac{6804}{6803}\frac{4}{M_{2,m}^2}\epsilon_{\mathrm{int}}. \tag{677}$$

Now, we use that an integral of an odd function, e.g., $\sin(x)\cos(x)$, over an even interval is zero (also using Assumption 4 that p^2 is even). We also use Corollaries 15 to 17 so that we have

$$\langle h_{w^\star,M_{1,m},M_{2,m}} | O_{k,m} | h_{w^\star,M_{1,m},M_{2,m}} \rangle \leq \frac{6804}{6803}\frac{1}{M_{2,m}^2}\sum_{j=1}^D (\beta_j^\star)^2\left(\frac{1}{2} + \frac{3\sqrt{d}}{16\pi R_w \tilde{R}}\right)\cos\left(\frac{2\pi j T w_k^\star}{M_{1,m}}\right) \tag{678}$$

$$+\frac{6804}{6803}\frac{1}{M_{2,m}^2}\sum_{\substack{j,j'=1\\ j\neq j'}}^D \beta_j^\star\beta_{j'}^\star\left(\frac{3\sqrt{d}}{2\pi R_w \tilde{R}}\right) + \epsilon_d + \frac{6804}{6803}\frac{4}{M_{2,m}^2}\epsilon_{\mathrm{int}}. \tag{679}$$

Note that Corollaries 15 to 17 apply when integrating with respect to the Gaussian density. Using Corollary 13 instead of Corollary 12 in their proofs, we see the results still hold when integrating with respect to p_T^2. Using our choice of $\tilde{R} \geq \max(39\sqrt{d}/(4\pi R_w), 54D^2\sqrt{d}/(\pi R_w))$, we have

$$\langle h_{w^\star,M_{1,m},M_{2,m}} | O_{k,m} | h_{w^\star,M_{1,m},M_{2,m}} \rangle \tag{680}$$

$$\leq \frac{1}{M_{2,m}^2}\left(\frac{6804}{6803}\frac{27}{52}\sum_{j=1}^D (\beta_j^\star)^2\cos\left(\frac{2\pi j T w_k^\star}{M_{1,m}}\right) + \frac{6804}{6803}\frac{1}{36D^2} + \frac{3}{M_{2,m}} + \frac{6805}{6803}\frac{4}{21D^2}\right) \tag{681}$$

$$\leq \frac{1}{M_{2,m}^2}\left(\frac{6804}{6803}\frac{27}{52}(\beta_m^\star)^2\cos\left(\frac{2\pi T w_k^\star}{M_1}\right) + \frac{6804}{6803}\frac{27}{52}\sum_{\substack{j=1\\ j\neq m}}^D (\beta_j^\star)^2 + \frac{6804}{6803}\frac{1}{36D^2} + \frac{3}{M_{2,m}} + \frac{6805}{6803}\frac{4}{21D^2}\right) \tag{682}$$

In the first line, we also use $\|\beta^\star\|_2^2 \leq 1$ since $\|\beta^\star\|_1 = 1$. In addition, we use $|\epsilon_d| \leq 3/M_{2,m}^3$ by Claim 8, and $|\epsilon_{\mathrm{int}}| \leq 6805/(21\cdot 6804D^2)$ by Claim 7. In the second line, we use $M_{1,m} = mM_1$. We can further bound the cosine term using Equation (221):

$$\langle h_{w^\star,M_{1,m},M_{2,m}} | O_{k,m} | h_{w^\star,M_{1,m},M_{2,m}} \rangle \tag{683}$$

$$\leq \frac{1}{M_{2,m}^2}\left(\frac{6804}{6803}\frac{27}{52}(\beta_m^\star)^2\left(1-\frac{1}{8}\left(\frac{2\pi R_w}{M_1}\right)^2\right)+\frac{6804}{6803}\frac{27}{52}\sum_{\substack{j=1\\ j\neq m}}^{D}(\beta_j^\star)^2+\frac{6804}{6803}\frac{1}{36D^2}+\frac{3}{M_{2,m}}+\frac{6805}{6803}\frac{4}{21D^2}\right) \tag{684}$$

$$\leq \frac{1}{M_{2,m}^2}\left(\frac{13}{25}\left(1-\frac{1}{8}\left(\frac{2\pi R_w}{M_1}\right)^2(\beta_m^\star)^2\right)+\frac{2}{9D^2}+\frac{3}{M_{2,m}}\right). \tag{685}$$

In the last line, we use that $\|\beta^\star\|_2^2 \leq 1$ since $\|\beta^\star\|_1 = 1$. Summing over all $m \in \{1,\ldots,D\}$, then we have

$$\sum_{m=1}^{D}\langle h_{w^\star,M_{1,m},M_{2,m}}|O_{k,m}|h_{w^\star,M_{1,m},M_{2,m}}\rangle \leq \sum_{m=1}^{D}\frac{1}{M_{2,m}^2}\left(\frac{13}{25}\left(1-\frac{1}{8}\left(\frac{2\pi R_w}{M_1}\right)^2(\beta_m^\star)^2\right)+\frac{2}{9D^2}+\frac{3}{M_{2,m}}\right) \tag{686}$$

$$\leq \frac{1}{M_2^2}\sum_{m=1}^{D}\left(\frac{13}{25}\left(1-\frac{1}{8}\left(\frac{2\pi R_w}{M_1}\right)^2(\beta_m^\star)^2\right)+\frac{2}{9D^2}+\frac{3}{M_2}\right) \tag{687}$$

$$\leq \frac{1}{M_2^2}\left(\frac{13}{25}D-\frac{1}{8D}\left(\frac{2\pi R_w}{M_1}\right)^2+\frac{2}{9D}+\frac{3D}{M_2}\right). \tag{688}$$

In the second line, we use $M_{2,m} = mM_2$ by definition and $m \geq 1$. In the last line, we use $\|\beta^\star\|_2^2 \geq 1/D$. Thus, we see that if $|T - \ell M_1/w_k^\star| \not\lesssim 1$ for any integer ℓ, then this upper bound on the sum of expectation values must be satisfied. Finally, our choice of τ and the condition on $\sum_{m=1}^{D}\alpha_m$ guarantees that this upper bound on also holds, as required. □

□

Finally, using Theorem 10 and Theorem 9, we can prove Corollary 11.

Proof of Corollary 11. Choose the discretization parameter to be $M_1 = \max(70\pi d D^3 R_w, R_w^2/\epsilon_1)$. By Lemma 6, we know that there exists a discretization $h_{w^\star,M_1,M_2}$ of the target function $g_{w^\star}$ such that $h_{w^\star,M_1,M_2}$ is (33/35)-pseudoperiodic with period $S_j = M_1/w_j^\star$ in each component. Note that $S_j \geq 1$ by our choice of discretization parameter. Moreover, we know an upper bound on the period $A = M_1 d^2/R_w$ by Equation (9). Finally, we have an efficient verification procedure by Theorem 10. Thus, we satisfy all of the conditions of Theorem 9, so applying its result, we can find integers a_j such that $|a_j - S_j| \leq 1$ with probability $\Omega(1/\log^4(M_1 d^2/R_w))$. The rest of the proof then follows in the same way as Theorem 5 by our choice of $M_1 \geq R_w^2/\epsilon_1$. □

B. Learning the outer function via gradient methods

As in Section IV B, now that we have an approximation $\hat{w}$ of $w^\star$ such that $\|\hat{w} - w^\star\|_\infty \leq \epsilon_1$, we want to learn the outer periodic function $\tilde{g} : \mathbb{R} \to [-1,1]$ via classical gradient methods. Again, this portion of the algorithm is purely classical. The difference with Section IV B is that the density φ^2 is now not a uniform density. In particular, we consider a probability distribution $\varphi^2 \propto \prod_{k=1}^{d} p_k^2$ over $[-R,R]^d$, where R is the truncation parameter. We also consider that φ^2 satisfies Assumptions 1-6. In particular for this part of the algorithm, we only need φ^2 to satisfy Assumptions 2-4.

Explicitly, we consider a density function

$$\varphi^2(x) = \frac{1}{\prod_{j=1}^{d}\left(\int_{-R}^{+R} p_j^2(z)\,dz\right)}\prod_{j=1}^{d} p_j^2(x_j). \tag{689}$$

Recall that our target function is

$$g_{w^\star}(x) = \tilde{g}(x^\intercal w^\star) = \sum_{j=1}^{D}\beta_j^\star \cos(2\pi j x^\intercal w^\star), \tag{690}$$

and we want to find a good predictor

$$f_\beta(x) = \sum_{j=1}^{D} \beta_j \cos(2\pi j x^\intercal \hat{w}), \tag{691}$$

that minimizes the objective function

$$\mathcal{L}_{w^\star}(\beta) = \mathop{\mathbb{E}}_{x\sim\varphi^2}[(f_\beta(x) - g_{w^\star}(x))^2] = \int\limits_{x\sim\varphi^2} \left(\sum_{j=1}^{D} \beta_j^\star \cos(2\pi j x^\intercal w^\star) - \sum_{j=1}^{D} \beta_j \cos(2\pi j x^\intercal \hat{w}) \right)^2 dx, \tag{692}$$

where $\hat{w}$ is our approximation of $w^\star$ from Corollary 11. As in the classical hardness result [12], our algorithm is given access to this loss function and its gradients. Using this, we design a classical algorithm that can effiicently find a predictor specified by parameters $\hat{\beta}$ such that $\mathcal{L}_{w^\star}(\hat{\beta}) \leq \epsilon$ for a given precision $\epsilon > 0$.

In Section IV B, we proved that for an appropriate choice of truncation parameter R and accuracy ϵ_1 such that $\|\hat{w} - w^\star\|_\infty \leq \epsilon_1$, then we can achieve this small loss (Theorem 7). In fact, we can achieve the same guarantee for non-uniform distributions.

Theorem 11 (Learning $\tilde{g}$ Guarantee; Non-Uniform Case)**.** *Let $\varphi^2 \propto \prod_{k=1}^d p_k^2$ be a probability distribution over $[-R, R]^d$ satisfying Assumptions 2-4 for R defined shortly Let $\epsilon > 0$. Let $w^\star \in \mathbb{R}^d$ be unknown with norm $R_w > 0$, and let $g_{w^\star} : \mathbb{R}^d \to [-1, 1]$ be defined as $g_{w^\star}(x) = \tilde{g}(x^\intercal w^\star)$ for $\tilde{g}$ given in Equation (10). Choose*

$$R = \tilde{\Omega}\left(\max\left(\frac{D^2}{\epsilon}, \frac{D^2\sqrt{d}}{R_w \epsilon}, \frac{D^{5/2}}{\sqrt{\epsilon}}, \frac{D^{3/2}\sqrt{d}}{R_w\sqrt{\epsilon}} \right) \right), \tag{693}$$

$$\epsilon_1 = \tilde{\mathcal{O}}\left(\min\left(\frac{\epsilon^3}{D^6 d}, \frac{\epsilon^{3/2}}{D^{13/2} d}, \frac{R_w}{D\sqrt{d}} \right) \right). \tag{694}$$

Suppose we have an approximation $\hat{w} \in \mathbb{R}^d$ such that $\|\hat{w} - w^\star\|_\infty \leq \epsilon_1$. Then, there exists a classical algorithm with access to the loss function from Equation (260) and its derivatives that can efficiently find a parameters $\hat{\beta} \in \mathbb{R}^d$ such that $\mathcal{L}_{w^\star}(\hat{\beta}) \leq \epsilon$. Moreover, this algorithm requires at most

$$t = \Theta\left(\log\left(\sqrt{\frac{D}{\epsilon}} \right) \right) \tag{695}$$

iterations of gradient descent.

The proof of this theorem is simple given what we have already proven in Section IV B. There, notice that the proof only depends on the distribution φ^2 through Lemmas 7 and 10. In fact, notice that in these lemmas, their proofs only depend on φ^2 via the integral bounds in Section IV C. Thus, to prove Theorem 11, we only need to obtain similar integral bounds when φ^2 is a non-uniform distribution satisfying Assumptions 2-4. We achieve this in Section V C. These integral bounds differ from the uniform case only in constant factors, thus immediately giving the result.

C. Integral bounds

Similarly to Section IV C, we need the following technical lemmas for bounding integrals when the integral is taken with respect to a non-uniform distribution instead. We require that the distribution satisfies Assumptions 2-4 in order for all of the bounds to hold. Some bounds only require Assumptions 2 and 3.

Lemma 15. *Let $\varphi^2 \propto \prod_{k=1}^d p_k^2$ be a probability distribution over $[-R, R]^d$ satisfying Assumptions 2 and 3 for a truncation parameter R. Let $w^\star \in \mathbb{R}^d$ be unknown with norm $R_w > 0$, and let $\hat{w} \in \mathbb{R}^d$ be an approximation of $w^\star$ with $\|\hat{w} - w^\star\|_\infty \leq \epsilon_1$. Let $1 \leq j, j' \leq D$ be integers with $j \neq j'$, for $D \in \mathbb{N}$ from Equation (10). Then,*

$$\left| \int\limits_{x\sim\varphi^2} e^{2\pi i x^\intercal \hat{w}(j-j')}\, dx \right| \leq \frac{3}{4\pi R} \frac{\sqrt{d}}{R_w - \sqrt{d}\epsilon_1}. \tag{696}$$

Proof. The proof follows similarly to that of Lemma 11. Denote the normalization constant by

$$G \triangleq \prod_{k=1}^{d}\left(\int_{-R}^{+R} p_k^2(z)\,dz\right). \tag{697}$$

We can bound this integral using

$$\left|\int_{x\sim\varphi^2} e^{2\pi i x^\intercal \hat{w}(j-j')}\,dx\right| \tag{698}$$

$$= \left|\frac{1}{G}\int_{x_1=-R}^{+R}\cdots\int_{x_d=-R}^{+R} e^{2\pi i\sum_{k=1}^{d} x_k\hat{w}_k(j-j')}p_1^2(x_1)\cdots p_d^2(x_d)\,dx_d\cdots dx_1\right| \tag{699}$$

$$= \left|\frac{1}{G}\prod_{k=1}^{d}\int_{x_k=-R}^{+R} e^{2\pi i x_k\hat{w}_k(j-j')}p_k^2(x_k)\,dx_k\right|. \tag{700}$$

Here, notice that we can bound each of these integrals trivially

$$\left|\int_{x_k=-R}^{+R} e^{2\pi i x_k\hat{w}_k(j-j')}p_k^2(x_k)\,dx_k\right| \le \int_{x_k=-R}^{+R} p_k^2(x_k)\,dx_k, \tag{701}$$

where we use that $|e^{2\pi i z}| \le 1$. We also notice that because $\|w^\star\|_2^2 = \sum_{i=1}^{d}|w_i^\star|^2 = R_w^2$, then there must exist some $k\in[d]$ such that $|w_k^\star| \ge R_w/\sqrt{d}$. Here, equality is satisfied for the case when $w_i = R_w/\sqrt{d}$ for all $i\in[d]$. We will bound each integral in the product in Equation (700) using Equation (701) except for this k such that $|w_k^\star| \ge R_w/\sqrt{d}$:

$$\left|\int_{x\sim\varphi^2} e^{2\pi i x^\intercal \hat{w}(j-j')}\,dx\right| = \left|\frac{1}{G}\prod_{k=1}^{d}\int_{x_k=-R}^{+R} e^{2\pi i x_k\hat{w}_k(j-j')}p_k^2(x_k)\,dx_k\right| \tag{702}$$

$$\le \frac{1}{\left(\int_{-R}^{+R} p_k^2(z)\,dz\right)}\left|\int_{x_k=-R}^{+R} e^{2\pi i x_k\hat{w}_k(j-j')}p_k^2(x_k)\,dx_k\right| \tag{703}$$

$$\le \frac{1}{\left(\int_{-R}^{+R} p_k^2(z)\,dz\right)}\left|\int_{x_k=-R}^{+R} e^{2\pi i x_k\hat{w}_k(j-j')}\,dx_k\right| \tag{704}$$

$$\le \frac{3}{4R}\left|\int_{x_k=-R}^{+R} e^{2\pi i x_k\hat{w}_k(j-j')}\,dx_k\right|, \tag{705}$$

where in the second line, we use Equation (701). In the third line, we use that $p_k^2(x) \le 1$ by Assumption 3. In the fourth line, we use Assumption 2. In particular, by Assumption 2, we have

$$\int_{-R}^{+R} p_k^2(z)\,dz - 2R = \int_{-R}^{+R}(p_k^2(z)-1)\,dz \ge -\frac{R}{5}. \tag{706}$$

Thus, this implies that $\int_{-R}^{+R} p_k^2(z)\,dz \ge 2R - R/5 \ge 4R/3$. From here, the proof is the same as that of Lemma 11, just carrying through a constant factor of 3/4 instead of 1/2. □

By essentially the same proof, we can obtain a similar upper bound replacing $\hat{w}$ with $w^\star$.

Corollary 12. *Let $\varphi^2 \propto \prod_{k=1}^{d} p_k^2$ be a probability distribution over $[-R,R]^d$ satisfying Assumptions 2 and 3 for a truncation parameter R. Let $w^\star \in \mathbb{R}^d$ be unknown with norm $R_w > 0$, and let $\hat{w}\in\mathbb{R}^d$ be an approximation of $w^\star$ with $\|\hat{w}-w^\star\|_\infty \le \epsilon_1$. Let $1 \le j, j' \le D$ be integers with $j \ne j'$, for $D\in\mathbb{N}$ from Equation (10). Then,*

$$\left|\int_{x\sim\varphi^2} e^{2\pi i x^\intercal w^\star(j-j')}\,dx\right| \le \frac{3}{4\pi R}\frac{\sqrt{d}}{R_w}. \tag{707}$$

We also have a similar corollary, where the integral is taken over a slightly different distribution. This is useful in Section V A 2.

Corollary 13. *Consider the space $[-R,R]^d \subseteq \mathbb{R}^d$ and nonnegative functions $p_i : \mathbb{R} \to [0,1]$ satisfying Assumptions 2 and 3 for $i \in [d]$. Let $w^\star \in \mathbb{R}^d$ be unknown with norm $R_w > 0$, and let $\hat{w} \in \mathbb{R}^d$ be an approximation of $w^\star$ with $\|\hat{w} - w^\star\|_\infty \leq \epsilon_1$. Let $1 \leq j, j' \leq D$ be integers with $j \neq j'$, for $D \in \mathbb{N}$ from Equation (10). Let M_1, T and $k \in [d]$ be integers. Then,*

$$\left| \frac{1}{G} \int_{[-R,R]^d} p_1^2(x_1) \cdots p_k(x_k) p_k(M_1 x_k + T) \cdots p_d^2(x_d) e^{2\pi i x^\intercal w^\star (j-j')} \, dx \right| \leq \frac{3}{4\pi R} \frac{\sqrt{d}}{R_w}, \tag{708}$$

where

$$G \triangleq \prod_{i=1}^{d} \left(\int_{-R}^{+R} p^2(x_j) \, dx_j \right). \tag{709}$$

Proof. We can rewrite the integral as

$$\left| \frac{1}{G} \int_{[-R,R]^d} p_1^2(x_1) \cdots p_k(x_k) p_k(M_1 x_k + T) \cdots p_d^2(x_d) e^{2\pi i x^\intercal w^\star (j-j')} \, dx \right| \tag{710}$$

$$= \left| \frac{1}{G} \left(\prod_{\substack{\ell=1 \\ \ell \neq k}}^{d} \int_{-R}^{+R} p_\ell^2(x_\ell) e^{2\pi i x_\ell w_\ell^\star (j-j')} \, dx_\ell \right) \left(\int_{-R}^{+R} p_k(x_k) p_k(M_1 x_k + T) e^{2\pi i x_k w_k^\star (j-j')} \, dx_k \right) \right|. \tag{711}$$

Notice that we can bound each of these integrals trivially as in Equation (701). Also, notice that because $\|w^\star\|_2^2 = R_w^2$, then there must exist some $k' \in [d]$ such that $|w_k^\star| \geq R_w/\sqrt{d}$. We will bound each integral in the product in Equation (711) using Equation (701) except for this k' such that $|w_{k'}^\star| \geq R_w/\sqrt{d}$. If $k = k'$, then

$$\left| \frac{1}{G} \int_{[-R,R]^d} p_1^2(x_1) \cdots p_k(x_k) p_k(M_1 x_k + T) \cdots p_d^2(x_d) e^{2\pi i x^\intercal w^\star (j-j')} \, dx \right| \tag{712}$$

$$\leq \frac{1}{\int_{-R}^{+R} p_k^2(z) \, dz} \left| \int_{x_k=-R}^{+R} p_k(x_k) p_k(M_1 x_k + T) e^{2\pi x_k w_k^\star (j-j')} \, dx_k \right| \tag{713}$$

$$\leq \frac{1}{\int_{-R}^{+R} p_k^2(z) \, dz} \left| \int_{x_k=-R}^{+R} e^{2\pi i x_k w_k^\star (j-j')} \, dx_k \right|. \tag{714}$$

Here, in the last line, we used Assumption 3 that $p_k(z) \leq 1$. From here, the proof is the same as Lemma 15 and Corollary 12. If $k \neq k'$, then

$$\left| \frac{1}{G} \int_{[-R,R]^d} p_1^2(x_1) \cdots p_k(x_k) p_k(M_1 x_k + T) \cdots p_d^2(x_d) e^{2\pi i x^\intercal w^\star (j-j')} \, dx \right| \tag{715}$$

$$\leq \frac{1}{\left(\int_{-R}^{+R} p_k^2(x_k) \, dx_k \right) \left(\int_{-R}^{+R} p_{k'}^2(x_{k'}) \, dx_{k'} \right)} \left| \int_{-R}^{+R} p_k(x_k) p_k(M_1 x_k + T) e^{2\pi i x_k w_k^\star (j-j')} \, dx_k \right| \tag{716}$$

$$\cdot \left| \int_{-R}^{+R} p_{k'}^2(x_{k'}) e^{2\pi i x_{k'} w_{k'}^\star (j-j')} \, dx_{k'} \right| \tag{717}$$

$$\leq \frac{2R}{\left(\int_{-R}^{+R} p_k^2(x_k) \, dx_k \right) \left(\int_{-R}^{+R} p_{k'}^2(x_{k'}) \, dx_{k'} \right)} \left| \int_{-R}^{+R} e^{2\pi i x_{k'} w_{k'}^\star (j-j')} \, dx_{k'} \right| \tag{718}$$

$$\leq \frac{50}{81R} \left| \int_{-R}^{+R} e^{2\pi i x_{k'} w_{k'}^\star (j-j')} \, dx_{k'} \right|. \tag{719}$$

$$\leq \frac{3}{4R} \left| \int_{-R}^{+R} e^{2\pi i x_{k'} w_{k'}^\star (j-j')} \, dx_{k'} \right| \tag{720}$$

In the second inequality, we use Assumption 3 that $p_k(z) \leq 1$. In the next to last inequality, we use Assumption 2. In particular, by Assumption 2, we have

$$\int_{-R}^{+R} p_k^2(z) \, dz - 2R = \int_{-R}^{+R} (p_k^2(z) - 1) \, dz \geq -\frac{R}{5}. \tag{721}$$

Thus, this implies that $\int_{-R}^{+R} p_k^2(z)\,dz \geq 2R - R/5 = 9R/5$. From here, again, the proof is the same as Lemma 15 and Corollary 12. □

Now, we can use this to obtain a lower bound for an integral of a product of cosines, as in Lemma 12. In this next integral bound, we also require Assumption 4.

Lemma 16. *Let $\varphi^2 \propto \prod_{k=1}^d p_k^2$ be a probability distribution over $[-R, R]^d$ satisfying Assumptions 2-4 for a truncation parameter R. Let $w^\star \in \mathbb{R}^d$ be unknown with norm $R_w > 0$, and let $\hat{w} \in \mathbb{R}^d$ be an approximation of $w^\star$ with $\|\hat{w} - w^\star\|_\infty \leq \epsilon_1$. Let $1 \leq j \leq D$ be an integer, for $D \in \mathbb{N}$ from Equation (10). Then,*

$$\int_{x\sim\varphi^2} \cos(2\pi j x^\intercal \hat{w}) \cos(2\pi j x^\intercal w^\star)\,dx \geq \frac{1}{2} - \frac{3\sqrt{d}}{16\pi R_w R} - \frac{5\pi^2 D^2 R^2 d\epsilon_1}{2}. \tag{722}$$

Proof. The proof follows similarly to that of Lemma 12. Using the sum formulas for cosine, we have

$$\int_{x\sim\varphi^2} \cos(2\pi j x^\intercal \hat{w}) \cos(2\pi j x^\intercal w^\star)\,dx \tag{723}$$

$$= \int_{x\sim\varphi^2} \cos(2\pi j x^\intercal (w^\star + (\hat{w} - w^\star))) \cos(2\pi j x^\intercal w^\star)\,dx \tag{724}$$

$$= \int_{x\sim\varphi^2} (\cos(2\pi j x^\intercal w^\star)\cos(2\pi j x^\intercal(\hat{w} - w^\star)) - \sin(2\pi j x^\intercal w^\star)\sin(2\pi j x^\intercal(\hat{w} - w^\star)))\cos(2\pi j x^\intercal w^\star)\,dx \tag{725}$$

$$\geq \int_{x\sim\varphi^2} \cos^2(2\pi j x^\intercal w^\star)\left(1 - \frac{1}{2}(2\pi j x^\intercal(\hat{w} - w^\star))^2\right) - \sin(2\pi j x^\intercal w^\star)\sin(2\pi j x^\intercal(\hat{w} - w^\star))\cos(2\pi j x^\intercal w^\star)\,dx \tag{726}$$

$$\geq \int_{x\sim\varphi^2} \cos^2(2\pi j x^\intercal w^\star)\,dx - 2\pi^2 j^2 \int_{x\sim\varphi^2} (x^\intercal(\hat{w} - w^\star))^2\,dx - 2\pi j \int_{x\sim\varphi^2} |x^\intercal(\hat{w} - w^\star)|\,dx. \tag{727}$$

In the third line, we use the sum formula for cosines. In the fourth line, we use that $\cos(y) \geq 1 - y^2/2$. In the fifth line, we use that $\sin(y), \cos(y) \leq 1$ and $\sin(y) \leq |y|$. We want to lower bound the first term and upper bound the second two.

First, we will lower bound the first term in Equation (727). We can expand the first term in terms of complex exponentials:

$$\int_{x\sim\varphi^2} \cos^2(2\pi j x^\intercal w^\star)\,dx = \frac{1}{4}\int_{x\sim\varphi^2} \left(e^{2\pi i j x^\intercal w^\star} + e^{-2\pi i j x^\intercal w^\star}\right)^2 dx \tag{728}$$

$$= \frac{1}{2} + \frac{1}{4}\int_{x\sim\varphi^2} e^{4\pi i j x^\intercal w^\star}\,dx + \frac{1}{4}\int_{x\sim\varphi^2} e^{-4\pi i j x^\intercal w^\star}\,dx. \tag{729}$$

Now, we can bound the absolute value of these complex exponentials via Corollary 12. Note that Corollary 12 applies because we only needed to use that $j \neq j'$ to lower bound $|j - j'| \geq 1$. This already clearly holds for $j \geq 1$. Thus, we have

$$\left|\int_{x\sim\varphi^2} \cos^2(2\pi j x^\intercal w^\star)\,dx - \frac{1}{2}\right| \leq \frac{1}{2}\left|\int_{x\sim\varphi^2} e^{4\pi i j x^\intercal w^\star}\,dx\right| \leq \frac{3}{16\pi R}\frac{\sqrt{d}}{R_w}. \tag{730}$$

Rearranging, we have

$$\int_{x\sim\varphi^2} \cos^2(2\pi j x^\intercal w^\star)\,dx \geq \frac{1}{2} - \frac{3\sqrt{d}}{16\pi R_w R}. \tag{731}$$

This gives a lower bound on the first term in Equation (727). We still need to upper bound the other terms in Equation (727). For the second term, we can first directly evaluate the integral.

For $\varphi^2 \propto \prod_{k=1}^d p_k^2$ over $[-R, R]^d$, we have

$$\int_{x\sim\varphi^2} dx = \frac{1}{\prod_{k=1}^d \left(\int_{-R}^{+R} p_k^2(z)\,dz\right)} \int_{x_1=-R}^{+R} \cdots \int_{x_d=-R}^{+R} p_1^2(x_1)\cdots p_d^2(x_d)\,dx_d \cdots dx_1 = 1. \tag{732}$$

For simplicity, from here on, we denote the normalizing factor by G. Then,

$$\int_{x\sim\varphi^2} (x^\intercal(\hat{w} - w^\star))^2 \, dx \tag{733}$$

$$= \frac{1}{G} \int_{x_1=-R}^{+R} \cdots \int_{x_d=-R}^{+R} \left(\sum_{i=1}^{d} x_i \hat{w}_i - x_i w_i^\star \right)^2 p_1^2(x_1) \cdots p_d^2(x_d) \, dx_d \cdots dx_1 \tag{734}$$

$$= \frac{1}{G} \int_{x_1=-R}^{+R} \cdots \int_{x_d=-R}^{+R} \left(\sum_{i,i'=1}^{d} x_i x_{i'} \hat{w}_i \hat{w}_{i'} + x_i x_{i'} w_i^\star w_{i'}^\star - x_i x_{i'} \hat{w}_i w_{i'}^\star - x_i x_{i'} w_i^\star \hat{w}_{i'} \right) \tag{735}$$

$$\cdot \, p_1^2(x_1) \cdots p_d^2(x_d) \, dx_d \cdots dx_1. \tag{736}$$

Here, notice that

$$\frac{1}{G} \int_{x_1=-R}^{+R} \cdots \int_{x_d=-R}^{+R} x_i x_{i'} p_1^2(x_1) \cdots p_d^2(x_d) \, dx_d \cdots dx_1 \tag{737}$$

$$= \frac{1}{\left(\int_{-R}^{+R} p_i^2(z) \, dz \right) \left(\int_{-R}^{+R} p_{i'}^2(z) \, dz \right)} \int_{x_i=-R}^{+R} \int_{x_{i'}=-R}^{+R} x_i x_{i'} p_i^2(x_i) p_{i'}^2(x_{i'}) \, dx_{i'} \, dx_i \tag{738}$$

$$= \frac{\delta_{ii'}}{\left(\int_{-R}^{+R} p_i^2(z) \, dz \right)} \int_{x=-R}^{+R} x^2 p_i^4(x) \, dx \tag{739}$$

$$\leq \frac{\delta_{ii'}}{\left(\int_{-R}^{+R} p_i^2(z) \, dz \right)} \int_{x=-R}^{+R} x^2 \, dx \tag{740}$$

$$= \frac{\delta_{ii'}}{\left(\int_{-R}^{+R} p_i^2(z) \, dz \right)} \frac{2R^3}{3} \tag{741}$$

$$\leq \frac{R^2}{2} \delta_{ii'}, \tag{742}$$

where the third line follows because if $i \neq i'$, we are integrating an odd function over a symmetric interval since p^2 is even by Assumption 4. The fourth line follows by Assumption 3 that $p_i(z) \leq 1$. The last line follows by Assumption 2. In particular, by Assumption 2, we have

$$\int_{-R}^{+R} p_k^2(z) \, dz - 2R = \int_{-R}^{+R} (p_k^2(z) - 1) \, dz \geq -\frac{R}{5}. \tag{743}$$

Thus, this implies that $\int_{-R}^{+R} p_k^2(z) \, dz \geq 2R - R/5 \geq 4R/3$. Plugging this into our previous expression, we have

$$\int_{x\sim\varphi^2} (x^\intercal(\hat{w} - w^\star))^2 \, dx \leq \frac{R^2}{2} \left(\sum_{i=1}^{d} (\hat{w}_i)^2 + (w_i^\star)^2 - 2\hat{w}_i w_i^\star \right) \tag{744}$$

$$= \frac{R^2}{2} \|\hat{w} - w^\star\|_2^2 \tag{745}$$

$$\leq \frac{R^2}{2} d\epsilon_1^2, \tag{746}$$

where in the last line, we used $|\hat{w}_i - w_i^\star| \leq \epsilon_1$ for all $i \in [d]$. Finally, we can similarly upper bound the last term in Equation (727).

$$\int_{x\sim\varphi^2} |x^\intercal(\hat{w} - w^\star)| \, dx \tag{747}$$

$$= \frac{1}{G} \int_{x_1=-R}^{+R} \cdots \int_{x_d=-R}^{+R} \left| \sum_{i=1}^{d} x_i (\hat{w}_i - w_i^\star) \right| p_1^2(x_1) \cdots p_d^2(x_d) \, dx_d \cdots dx_1 \tag{748}$$

$$\leq \frac{1}{G} \int_{x_1=-R}^{+R} \cdots \int_{x_d=-R}^{+R} \sum_{i=1}^{d} |x_i (\hat{w}_i - w_i^\star)| p_1^2(x_1) \cdots p_d^2(x_d) \, dx_d \cdots dx_1 \tag{749}$$

$$= \sum_{i=1}^{d} \frac{1}{\int_{-R}^{+R} p_i^2(z)\, dz} |\hat{w}_i - w_i^\star| \int_{x_i=-R}^{+R} |x_i| p_i^2(x_i)\, dx_i \tag{750}$$

$$\leq \sum_{i=1}^{d} \frac{1}{\int_{-R}^{+R} p_i^2(z)\, dz} |\hat{w}_i - w_i^\star| \int_{x_i=-R}^{+R} |x_i|\, dx_i \tag{751}$$

$$\leq R^2 \epsilon_1 \sum_{i=1}^{d} \frac{1}{\int_{-R}^{+R} p_i^2(z)\, dz} \tag{752}$$

$$\leq \frac{3\epsilon_1 dR}{4}. \tag{753}$$

In the third line, we use triangle inequality. In the fifth line, we use Assumption 3 that $p_k(z) \leq 1$. In the sixth line, we use that $|\hat{w}_i - w_i^\star| \leq \epsilon_1$ for all $i \in [d]$ and evaluate the integral. In the last line, we use Assumption 2.

Combining Equations (731), (746) and (753) in Equation (727), we have

$$\int_{x\sim\varphi^2} \cos(2\pi j x^\intercal \hat{w}) \cos(2\pi j x^\intercal w^\star)\, dx \geq \frac{1}{2} - \frac{3\sqrt{d}}{16\pi R_w R} - \pi^2 j^2 R^2 d\epsilon_1^2 - \frac{3\pi j \epsilon_1 dR}{2} \tag{754}$$

$$\geq \frac{1}{2} - \frac{3\sqrt{d}}{16\pi R_w R} - \pi^2 j^2 R^2 d\epsilon_1 - \frac{3\pi^2 j^2 \epsilon_1 dR^2}{2} \tag{755}$$

$$\geq \frac{1}{2} - \frac{3\sqrt{d}}{16\pi R_w R} - \frac{5\pi^2 D^2 R^2 d\epsilon_1}{2}, \tag{756}$$

where in the second line we use that $j, R \geq 1$ so that $j^2 \geq j$ and $R^2 \geq R$ and $\epsilon_1 < 1$ so that $\epsilon_1^2 \leq \epsilon_1$. In the last line, we use that $j \leq D$. □

Corollary 14. *Let $\varphi^2 \propto \prod_{k=1}^{d} p_k^2$ be a probability distribution over $[-R, R]^d$ satisfying Assumptions 2 and 3 for a truncation parameter R. Let $w^\star \in \mathbb{R}^d$ be unknown with norm $R_w > 0$, and let $\hat{w} \in \mathbb{R}^d$ be an approximation of $w^\star$ with $\|\hat{w} - w^\star\|_\infty \leq \epsilon_1$. Let $1 \leq j \leq D$ be an integer, for $D \in \mathbb{N}$ from Equation (10). Then,*

$$\int_{x\sim\varphi^2} \cos^2(2\pi j x^\intercal \hat{w})\, dx \geq \frac{1}{2} - \frac{3\sqrt{d}}{16\pi R(R_w - \sqrt{d}\epsilon_1)}. \tag{757}$$

Proof. The proof follows from the lower bound of the first term in Equation (727) in the proof of Lemma 16. We can expand the first term in terms of complex exponentials:

$$\int_{x\sim\varphi^2} \cos^2(2\pi j x^\intercal \hat{w})\, dx = \frac{1}{4} \int_{x\sim\varphi^2} \left(e^{2\pi i j x^\intercal \hat{w}} + e^{-2\pi i j x^\intercal \hat{w}}\right)^2 dx \tag{758}$$

$$= \frac{1}{2} + \frac{1}{4} \int_{x\sim\varphi^2} e^{4\pi i j x^\intercal \hat{w}}\, dx + \frac{1}{4} \int_{x\sim\varphi^2} e^{-4\pi i j x^\intercal \hat{w}}\, dx. \tag{759}$$

Now, we can bound the absolute value of these complex exponentials via Lemma 15 (instead of Corollary 12). Note that Lemma 15 applies because we only needed to use that $j \neq j'$ to lower bound $|j - j'| \geq 1$. This already clearly holds for $j \geq 1$. Thus, we have

$$\left| \int_{x\sim\varphi^2} \cos^2(2\pi j x^\intercal \hat{w})\, dx - \frac{1}{2} \right| \leq \frac{1}{2} \left| \int_{x\sim\varphi^2} e^{4\pi i j x^\intercal \hat{w}}\, dx \right| \leq \frac{3}{16\pi R} \frac{\sqrt{d}}{R_w - \sqrt{d}\epsilon_1}. \tag{760}$$

Rearranging, we have

$$\int_{x\sim\varphi^2} \cos^2(2\pi j x^\intercal \hat{w})\, dx \geq \frac{1}{2} - \frac{3\sqrt{d}}{16\pi R(R_w - \sqrt{d}\epsilon_1)}. \tag{761}$$

□

Lemma 17. *Let $\varphi^2 \propto \prod_{k=1}^{d} p_k^2$ be a probability distribution over $[-R, R]^d$ satisfying Assumptions 2 and 3 for a truncation parameter R. Let $w^\star \in \mathbb{R}^d$ be unknown with norm $R_w > 0$, and let $\hat{w} \in \mathbb{R}^d$ be an*

approximation of $w^\star$ with $\|\hat{w} - w^\star\|_\infty \le \epsilon_1$. Let $1 \le j \le D$ be an integer, for $D \in \mathbb{N}$ from Equation (10). Then,

$$\int\limits_{x\sim\varphi^2} \cos(2\pi j x^\intercal \hat{w}) \cos(2\pi j x^\intercal w^\star)\, dx \le \frac{1}{2} + \frac{3\sqrt{d}}{16\pi R_w R} + \frac{3\pi D d\epsilon_1 R}{2}. \tag{762}$$

Proof. The proof of this is similar to that of Lemmas 12 and 16. Using the sum formulas for cosine, we have

$$\int_{x\sim\varphi^2} \cos(2\pi j x^\intercal \hat{w}) \cos(2\pi j x^\intercal w^\star)\, dx \tag{763}$$

$$= \int_{x\sim\varphi^2} \cos(2\pi j x^\intercal (w^\star + (\hat{w} - w^\star))) \cos(2\pi j x^\intercal w^\star)\, dx \tag{764}$$

$$= \int_{x\sim\varphi^2} (\cos(2\pi j x^\intercal w^\star) \cos(2\pi j x^\intercal (\hat{w} - w^\star)) - \sin(2\pi j x^\intercal w^\star) \sin(2\pi j x^\intercal (\hat{w} - w^\star))) \cos(2\pi j x^\intercal w^\star)\, dx \tag{765}$$

$$\le \int_{x\sim\varphi^2} \cos^2(2\pi j x^\intercal w^\star) - \sin(2\pi j x^\intercal w^\star) \sin(2\pi j x^\intercal (\hat{w} - w^\star)) \cos(2\pi j x^\intercal w^\star)\, dx \tag{766}$$

$$\le \int_{x\sim\varphi^2} \cos^2(2\pi j x^\intercal w^\star) + \sin(2\pi j x^\intercal (\hat{w} - w^\star))\, dx \tag{767}$$

$$\le \int_{x\sim\varphi^2} \cos^2(2\pi j x^\intercal w^\star)\, dx + 2\pi j \int_{x\sim\varphi^2} |x^\intercal (\hat{w} - w^\star)|\, dx. \tag{768}$$

In the fourth line, we use that $\cos(y) \le 1$. In the fifth line, we use that $-\sin(y)\cos(y) \le 1$. In the last line, we use that $\sin(y) \le |y|$. We want to upper bound both of these terms, which is simple given the proof of Lemma 16.

Namely, in Equation (730), we showed that

$$\left| \int_{x\sim\varphi^2} \cos^2(2\pi j x^\intercal w^\star)\, dx - \frac{1}{2} \right| \le \frac{3}{16\pi R} \frac{\sqrt{d}}{R_w}. \tag{769}$$

Thus, we can upper bound

$$\int_{x\sim\varphi^2} \cos^2(2\pi j x^\intercal w^\star)\, dx \le \frac{1}{2} + \frac{3\sqrt{d}}{16\pi R_w R} \tag{770}$$

Note that we have already upper bounded the third term in Equation (753):

$$2\pi j \int_{x\sim\varphi^2} |x^\intercal (\hat{w} - w^\star)|\, dx \le \frac{3\pi j d\epsilon_1 R}{2} \le \frac{3\pi D d\epsilon_1 R}{2}. \tag{771}$$

Note that this part of the proof did not require Assumption 4. Combining Equation (770) and Equation (771) in Equation (768), we have

$$\int_{x\sim\varphi^2} \cos(2\pi j x^\intercal \hat{w}) \cos(2\pi j x^\intercal w^\star)\, dx \le \frac{1}{2} + \frac{3\sqrt{d}}{16\pi R_w R} + \frac{3\pi D d\epsilon_1 R}{2}. \tag{772}$$

□

Corollary 15. *Let $\varphi^2 \propto \prod_{k=1}^d p_k^2$ be a probability distribution over $[-R, R]^d$ satisfying Assumptions 2 and 3 for a truncation parameter R. Let $w^\star \in \mathbb{R}^d$ be unknown with norm $R_w > 0$, and let $\hat{w} \in \mathbb{R}^d$ be an approximation of $w^\star$ with $\|\hat{w} - w^\star\|_\infty \le \epsilon_1$. Let $1 \le j \le D$ be an integer, for $D \in \mathbb{N}$ from Equation (10). Then,*

$$\int\limits_{x\sim\varphi^2} \cos^2(2\pi j x^\intercal \hat{w})\, dx \le \frac{1}{2} + \frac{3\sqrt{d}}{16\pi R(R_w - \sqrt{d}\epsilon_1)}. \tag{773}$$

Proof. This follows directly from Equation (760). □

We also have a non-uniform analogue of Lemma 14. This is similar in spirit to the previous lemmas.

Lemma 18. *Let $\varphi^2 \propto \prod_{k=1}^d p_k^2$ be a probability distribution over $[-R, R]^d$ satisfying Assumptions 2 and 3 for a truncation parameter R. Let $w^\star \in \mathbb{R}^d$ be unknown with norm $R_w > 0$, and let $\hat{w} \in \mathbb{R}^d$ be an approximation of $w^\star$ with $\|\hat{w} - w^\star\|_\infty \leq \epsilon_1$. Let $1 \leq j, j' \leq D$ be integers with $j \neq j'$, for $D \in \mathbb{N}$ from Equation (10). Then,*

$$\left| \int_{x\sim\varphi^2} \cos(2\pi j x^\intercal \hat{w}) \cos(2\pi j' x^\intercal \hat{w})\, dx \right| \leq \frac{3}{4\pi R} \frac{\sqrt{d}}{R_w - \sqrt{d}\epsilon_1}. \tag{774}$$

Proof. The proof follows similarly to that of Lemma 14. Using the product formulas for cosine, we can write the integral as

$$\left| \int_{x\sim\varphi^2} \cos(2\pi j x^\intercal \hat{w}) \cos(2\pi j' x^\intercal \hat{w})\, dx \right| = \left| \frac{1}{2} \int_{x\sim\varphi^2} \cos(2\pi x^\intercal \hat{w}(j - j')) + \cos(2\pi x^\intercal \hat{w}(j + j'))\, dx \right|. \tag{775}$$

We can bound each of the integrals on the right hand side similarly. Starting with the first term, we can write it in terms of complex exponentials

$$\left| \int_{x\sim\varphi^2} \cos(2\pi x^\intercal \hat{w}(j - j'))\, dx \right| \leq \frac{1}{2} \left| \int_{x\sim\varphi^2} e^{2\pi i x^\intercal \hat{w}(j-j')}\, dx \right| + \frac{1}{2} \left| \int_{x\sim\varphi^2} e^{2\pi i x^\intercal \hat{w}(j'-j)}\, dx \right| \tag{776}$$

Both terms in Equation (776) can be bounded via Lemma 15. Thus, this bounds the first term in Equation (775) as

$$\left| \int_{x\sim\varphi^2} \cos(2\pi x^\intercal \hat{w}(j - j'))\, dx \right| \leq \frac{3}{4\pi R} \frac{\sqrt{d}}{R_w - \sqrt{d}\epsilon_1}. \tag{777}$$

We can similarly bound the second term in Equation (775). Namely, the argument is the same as the above and Lemma 14 so that we have

$$\left| \int_{x\sim\varphi^2} e^{2\pi i x^\intercal \hat{w}(j+j')}\, dx \right| \leq \frac{3}{4R} \frac{1}{\pi |j + j'| |\hat{w}_k|} \tag{778}$$

$$\leq \frac{1}{4R} \frac{1}{\pi |\hat{w}_k|}, \tag{779}$$

where since $j \neq j'$ and $j, j' \geq 1$, then $|j + j'| \geq 3$. The rest of the bound follows the same argument. Then, we obtain

$$\left| \int_{x\sim\varphi^2} \cos(2\pi x^\intercal \hat{w}(j + j'))\, dx \right| \leq \frac{1}{4\pi R} \frac{\sqrt{d}}{R_w - \sqrt{d}\epsilon_1} \leq \frac{3}{4\pi R} \frac{\sqrt{d}}{R_w - \sqrt{d}\epsilon_1}. \tag{780}$$

Thus, combined with Equation (777) in Equation (775), we have

$$\left| \int_{x\sim\varphi^2} \cos(2\pi j x^\intercal \hat{w}) \cos(2\pi j' x^\intercal \hat{w})\, dx \right| \leq \frac{3}{4\pi R} \frac{\sqrt{d}}{R_w - \sqrt{d}\epsilon_1}. \tag{781}$$

□

By essentially the same proof, we can obtain a similar upper bound replacing $\hat{w}$ with $w^\star$.

Corollary 16. *Let $\varphi^2 \propto \prod_{k=1}^d p_k^2$ be a probability distribution over $[-R, R]^d$ satisfying Assumptions 2 and 3 for a truncation parameter R. Let $w^\star \in \mathbb{R}^d$ be unknown with norm $R_w > 0$, and let $\hat{w} \in \mathbb{R}^d$ be an approximation of $w^\star$ with $\|\hat{w} - w^\star\|_\infty \leq \epsilon_1$. Let $1 \leq j, j' \leq D$ be integers with $j \neq j'$, for $D \in \mathbb{N}$ from Equation (10). Then,*

$$\left| \int_{x\sim\varphi^2} \cos(2\pi j x^\intercal w^\star) \cos(2\pi j' x^\intercal w^\star)\, dx \right| \leq \frac{3}{4\pi R} \frac{\sqrt{d}}{R_w}. \tag{782}$$

Corollary 17. *Let $\varphi^2 \propto \prod_{k=1}^{d} p_k^2$ be a probability distribution over $[-R,R]^d$ satisfying Assumptions 2 and 3 for a truncation parameter R. Let $w^\star \in \mathbb{R}^d$ be unknown with norm $R_w > 0$, and let $\hat{w} \in \mathbb{R}^d$ be an approximation of $w^\star$ with $\|\hat{w} - w^\star\|_\infty \le \epsilon_1$. Let $1 \le j, j' \le D$ be integers with $j \ne j'$, for $D \in \mathbb{N}$ from Equation (10). Then,*

$$\left| \int_{x\sim\varphi^2} \cos(2\pi j x^\intercal w^\star) \sin(2\pi j' x^\intercal w^\star)\, dx \right| \le \frac{3}{4\pi R} \frac{\sqrt{d}}{R_w}. \tag{783}$$

Proof. This follows by the same proof as Lemma 18 and Corollary 16. In particular, using the sum-product formulas for sine and cosine, we have

$$\left| \int_{x\sim\varphi^2} \cos(2\pi j x^\intercal w^\star) \sin(2\pi j' x^\intercal w^\star)\, dx \right| = \left| \frac{1}{2} \int_{x\sim\varphi^2} \sin(2\pi(j+j')x^\intercal w^\star) + \sin(2\pi(j'-j)x^\intercal w^\star)\, dx \right|. \tag{784}$$

Then, writing in terms of complex exponentials, we have

$$\left| \int_{x\sim\varphi^2} \sin(2\pi(j'-j)x^\intercal w^\star)\, dx \right| \le \frac{1}{|2i|} \left| \int_{x\sim\varphi^2} e^{2\pi i x^\intercal w^\star (j'-j)}\, dx \right| + \frac{1}{|2i|} \left| \int_{x\sim\varphi^2} e^{2\pi i x^\intercal w^\star (j-j')}\, dx \right|. \tag{785}$$

The rest of the proof is the same as Lemma 18, using Corollary 12 instead of Lemma 15 to bound the complex exponential terms. □

Finally, we need another integral bound that is also similar to Lemma 18. This is the non-uniform analogue of Corollary 8. The proof of this result follows easily following the steps of Corollary 16 and Corollary 8.

Corollary 18. *Let $\varphi^2 \propto \prod_{k=1}^{d} p_k^2$ be a probability distribution over $[-R,R]^d$ satisfying Assumptions 2 and 3 for a truncation parameter R. Let $w^\star \in \mathbb{R}^d$ be unknown with norm $R_w > 0$, and let $\hat{w} \in \mathbb{R}^d$ be an approximation of $w^\star$ with $\|\hat{w} - w^\star\|_\infty \le \epsilon_1$, where $\epsilon_1 \le R_w/(D\sqrt{d})$. Let $1 \le j, j' \le D$ be integers with $j \ne j'$, for $D \in \mathbb{N}$ from Equation (10). Then,*

$$\left| \int_{x\sim\varphi^2} \cos(2\pi j x^\intercal w^\star) \cos(2\pi j' x^\intercal \hat{w})\, dx \right| \le \frac{3}{4\pi R} \frac{\sqrt{d}}{R_w - D\sqrt{d}\epsilon_1}. \tag{786}$$

---

[1] Leslie G Valiant. A theory of the learnable. *Communications of the ACM*, 27(11):1134–1142, 1984.

[2] Michael Kearns. Efficient noise-tolerant learning from statistical queries. *Journal of the ACM (JACM)*, 45(6):983–1006, 1998.

[3] Nader H Bshouty and Jeffrey C Jackson. Learning dnf over the uniform distribution using a quantum example oracle. In *Proceedings of the eighth annual conference on Computational learning theory*, pages 118–127, 1995.

[4] Srinivasan Arunachalam, Alex B Grilo, and Henry Yuen. Quantum statistical query learning. *arXiv preprint arXiv:2002.08240*, 2020.

[5] Sebastien Bubeck, Sitan Chen, and Jerry Li. Entanglement is necessary for optimal quantum property testing. In *2020 IEEE 61st Annual Symposium on Foundations of Computer Science (FOCS)*, pages 692–703. IEEE, 2020.

[6] Srinivasan Arunachalam, Vojtech Havlicek, and Louis Schatzki. On the role of entanglement and statistics in learning. *Advances in Neural Information Processing Systems*, 36, 2024.

[7] Sean Hallgren. Polynomial-time quantum algorithms for pell's equation and the principal ideal problem. *Journal of the ACM (JACM)*, 54(1):1–19, 2007.

[8] Richard Jozsa. Notes on hallgren's efficient quantum algorithm for solving pell's equation. *arXiv preprint quant-ph/0302134*, 2003.

[9] Andrew M Childs and Wim Van Dam. Quantum algorithms for algebraic problems. *Reviews of Modern Physics*, 82(1):1–52, 2010.

[10] Peter W Shor. Algorithms for quantum computation: discrete logarithms and factoring. In *Proceedings 35th annual symposium on foundations of computer science*, pages 124–134. Ieee, 1994.

[11] Alexander Schrijver. *Theory of linear and integer programming.* John Wiley & Sons, 1998.

[12] Ohad Shamir. Distribution-specific hardness of learning neural networks. *Journal of Machine Learning Research*, 19(32):1–29, 2018.

[13] Shai Shalev-Shwartz, Ohad Shamir, and Shaked Shammah. Failures of gradient-based deep learning. In *International Conference on Machine Learning*, pages 3067–3075. PMLR, 2017.

[14] Le Song, Santosh Vempala, John Wilmes, and Bo Xie. On the complexity of learning neural networks. *Advances in neural information processing systems*, 30, 2017.

[15] Min Jae Song, Ilias Zadik, and Joan Bruna. On the cryptographic hardness of learning single periodic neurons. *Advances in neural information processing systems*, 34:29602–29615, 2021.

[16] Avrim Blum, Merrick Furst, Jeffrey Jackson, Michael Kearns, Yishay Mansour, and Steven Rudich. Weakly learning dnf and characterizing statistical query learning using fourier analysis. In *Proceedings of the twenty-sixth annual ACM symposium on Theory of computing*, pages 253–262, 1994.

[17] Shengqiao Li. Concise formulas for the area and volume of a hyperspherical cap. *Asian Journal of Mathematics & Statistics*, 4(1):66–70, 2010.

[18] James G Wendel. Note on the gamma function. *The American Mathematical Monthly*, 55(9):563, 1948.

[19] Roman Vershynin. *High-dimensional probability: An introduction with applications in data science*, volume 47. Cambridge university press, 2018.

[20] Nader H Bshouty and Vitaly Feldman. On using extended statistical queries to avoid membership queries. *Journal of Machine Learning Research*, 2(Feb):359–395, 2002.

[21] Shai Bendavid, Alon Itai, and Eyal Kushilevitz. Learning by distances. *Information and Computation*, 117(2):240–250, 1995.

[22] Alexandr Andoni, Rina Panigrahy, Gregory Valiant, and Li Zhang. Learning sparse polynomial functions. In *Proceedings of the twenty-fifth annual ACM-SIAM symposium on Discrete algorithms*, pages 500–510. SIAM, 2014.

[23] Alexandr Andoni, Rishabh Dudeja, Daniel Hsu, and Kiran Vodrahalli. Attribute-efficient learning of monomials over highly-correlated variables. In *Algorithmic Learning Theory*, pages 127–161. PMLR, 2019.

[24] Sitan Chen, Adam R Klivans, and Raghu Meka. Learning deep relu networks is fixed-parameter tractable. In *2021 IEEE 62nd Annual Symposium on Foundations of Computer Science (FOCS)*, pages 696–707. IEEE, 2022.

[25] Surbhi Goel, Aravind Gollakota, Zhihan Jin, Sushrut Karmalkar, and Adam Klivans. Superpolynomial lower bounds for learning one-layer neural networks using gradient descent. In *International Conference on Machine Learning*, pages 3587–3596. PMLR, 2020.

[26] Ilias Diakonikolas, Daniel M Kane, Vasilis Kontonis, and Nikos Zarifis. Algorithms and sq lower bounds for pac learning one-hidden-layer relu networks. In *Conference on Learning Theory*, pages 1514–1539. PMLR, 2020.

[27] Ke Yang. New lower bounds for statistical query learning. *Journal of Computer and System Sciences*, 70(4):485–509, 2005.

[28] Vitaly Feldman, Elena Grigorescu, Lev Reyzin, Santosh S Vempala, and Ying Xiao. Statistical algorithms and a lower bound for detecting planted cliques. *Journal of the ACM (JACM)*, 64(2):1–37, 2017.

[29] Balázs Szörényi. Characterizing statistical query learning: simplified notions and proofs. In *International Conference on Algorithmic Learning Theory*, pages 186–200. Springer, 2009.

[30] Vitaly Feldman. A complete characterization of statistical query learning with applications to evolvability. *Journal of Computer and System Sciences*, 78(5):1444–1459, 2012.

[31] Michael A Nielsen and Isaac L Chuang. *Quantum computation and quantum information*. Cambridge university press, 2010.

[32] Michael James David Powell. *Approximation theory and methods*. Cambridge university press, 1981.

[33] Godfrey Harold Hardy and Edward Maitland Wright. *An introduction to the theory of numbers*. Oxford university press, 1979.

[34] John Robert Taylor and William Thompson. *An introduction to error analysis: the study of uncertainties in physical measurements*, volume 2. Springer, 1982.

[35] Yurii Nesterov et al. *Lectures on convex optimization*, volume 137. Springer, 2018.

[36] Yanlin Chen and Ronald de Wolf. Quantum algorithms and lower bounds for linear regression with norm constraints. *arXiv preprint arXiv:2110.13086*, 2021.

[37] Yanlin Chen, András Gilyén, and Ronald de Wolf. A quantum speed-up for approximating the top eigenvectors of a matrix. *arXiv preprint arXiv:2405.14765*, 2024.

[38] Lov Grover and Terry Rudolph. Creating superpositions that correspond to efficiently integrable probability distributions. *arXiv preprint quant-ph/0208112*, 2002.

[39] Arthur G Rattew, Yue Sun, Pierre Minssen, and Marco Pistoia. The efficient preparation of normal distributions in quantum registers. *Quantum*, 5:609, 2021.

[40] Sam McArdle, András Gilyén, and Mario Berta. Quantum state preparation without coherent arithmetic. *arXiv preprint arXiv:2210.14892*, 2022.

[41] Adam Holmes and Anne Y Matsuura. Efficient quantum circuits for accurate state preparation of smooth, differentiable functions. In *2020 IEEE International Conference on Quantum Computing and Engineering (QCE)*, pages 169–179. IEEE, 2020.

[42] Jason Iaconis, Sonika Johri, and Elton Yechao Zhu. Quantum state preparation of normal distributions using matrix product states. *npj Quantum Information*, 10(1):15, 2024.

[43] M Th Subbotin. On the law of frequency of error. *Matematicheskii*, 31(2):296–301, 1923.

[44] John K Hunter and Bruno Nachtergaele. *Applied analysis*. World Scientific Publishing Company, 2001.
